# Casdatifan shows durable response linked to HIF-2α biology in kidney cancer

**DOI:** 10.1038/s41586-026-10718-x

**Published:** 2026-07-01

**Authors:** Toni K. Choueiri, Jaime Merchán, Amita Patnaik, Alexandra Drakaki, Brian I. Rini, Sun Young Rha, Jae Lyun Lee, Moshe C. Ornstein, Rohit Kumar, Clara Hwang, Yusra Shao, Se Hoon Park, Pedro C. Barata, Bradley A. McGregor, Paul Foster, Jianfen Chen, Melissa Eisen, Hunter Cole, Ben Weeder, Yinghui Guan, Jaskirat Singh, Angelo Kaplan, Soonweng Cho, Richard Markus, Omar Kabbarah, Rana R. McKay

**Affiliations:** 1 Dana-Farber Cancer Institute, Harvard Medical School, Boston, MA USA; 2 University of Miami Sylvester Comprehensive Cancer Center, Miami, FL USA; 3 The START Center for Cancer Research, San Antonio, TX USA; 4 Division of Hematology/Oncology, Department of Medicine, David Geffen School of Medicine, University of California, Los Angeles, Los Angeles, CA USA; 5 Vanderbilt-Ingram Cancer Center, Nashville, TN USA; 6 Yonsei Cancer Center, Yonsei University College of Medicine, Seoul, South Korea; 7 Asan Medical Center, University of Ulsan College of Medicine, Seoul, South Korea; 8 Department of Hematology and Medical Oncology, Cleveland Clinic Taussig Cancer Institute, Cleveland, OH USA; 9 Division of Hematology Oncology, James Graham Brown Cancer Center, University of Louisville, Louisville, KY USA; 10 Department of Internal Medicine, Henry Ford Health System, Henry Ford Cancer Institute, Detroit, MI USA; 11 Karmanos Cancer Center, Detroit, MI USA; 12 Department of Medicine, Samsung Medical Center, Sungkyunkwan University School of Medicine, Seoul, Korea; 13 University Hospitals Seidman Cancer Center, Cleveland, OH USA; 14 Arcus Biosciences, Inc., Hayward, CA USA; 15 Division of Hematology Oncology, University of California, San Diego, San Diego, CA USA

**Keywords:** Targeted therapies, Renal cell carcinoma

## Abstract

Clear cell renal cell carcinoma (ccRCC) is largely driven by the transcription factor hypoxia-inducible factor 2α (HIF-2α)^1^. Here we show that monotherapy with casdatifan—an orally bioavailable, potent and selective HIF-2α inhibitor^2^—produces meaningful, durable antitumour activity with manageable safety in individuals with refractory metastatic ccRCC. Dose-expansion data from the ARC-20 study (NCT05536141) are presented, including for the 100 mg once daily (QD) cohort (*n* = 32) and the total cohort (*n* = 127). Treatment discontinuation from casdatifan-related adverse events was infrequent (3%), and class-effect toxicities included anaemia and hypoxia. The confirmed objective response rates (ORRs) were 35% (95% confidence intervals (CI) = 19–55%; 100 mg QD) and 31% (95% CI = 23–40%; total); median progression-free survival (PFS) was not estimable (95% CI = 5.7–not estimable; 100 mg QD) and 12.2 months (9.4–20.6; total). Greater maximal reductions in serum erythropoietin were associated with improved clinical outcomes, including a higher ORR (*P* = 0.001), lower rates of progressive disease (*P* = 0.003) and longer PFS (*P* = 0.006). Erythropoietin expression was restricted to cancer cells and was significantly higher at the mRNA level in patients with clinical benefit. Concordantly, HIF-2α protein expression and HIF-2α expression signature were associated with prolonged PFS. Overall, our findings show that casdatifan achieves meaningful, durable responses with manageable safety. These data establish a link between on-target HIF-2α pathway modulation, tumour biology and clinical efficacy.

## Abstract (web summary)

Monotherapy with the HIF-2α inhibitor casdatifan achieves meaningful, durable responses linked to HIF-2α biology with manageable safety in the treatment of individuals with clear cell renal cell carcinoma.

## Main

RCC accounts for most kidney cancers and is among the top ten cancers in the USA, with ccRCC representing approximately 75% of all RCC cases^3,4^. Although the introduction of immune checkpoint inhibitors and antiangiogenic therapies has improved survival in ccRCC, there remains a need to improve clinical outcomes in patients who are refractory to first-line treatment^5–7^.

Most ccRCC tumours are characterized by inactivation of the *VHL* tumour suppressor gene, resulting in impaired degradation of HIF-α subunits^1,8^. Accumulation of HIF-2α leads to upregulation of genes that promote tumour growth, angiogenesis and metastasis^9^. The first-in-class HIF-2α inhibitor belzutifan clinically validated this target and is approved for the treatment of adults with advanced ccRCC who were previously treated with a PD-1 or PD-L1 inhibitor and a VEGF tyrosine kinase inhibitor (TKI)^10^. However, clinical studies showed that fewer than 25% of patients with previously treated advanced ccRCC responded to treatment with single-agent belzutifan^11,12^. Pharmacokinetic analyses indicate that the clinical activity of belzutifan may be constrained by saturable absorption, as exposure in humans does not increase beyond the approved 120 mg dose^11,13,14^.

Casdatifan is a potent, selective, orally bioavailable HIF-2α inhibitor that was designed to enhance tissue penetration, reflected in its improved pharmacodynamic profile relative to other members of the therapeutic class^2,15^. In the phase I ARC-14 study, casdatifan exhibited manageable toxicity and dose-proportional and time-invariant pharmacokinetics across doses of 3–100 mg in healthy participants^2^. Importantly, the findings of the ARC-14 study indicate that higher doses of casdatifan lead to deeper suppression of serum erythropoietin (sEPO)^2^, a well-established pharmacodynamic biomarker of HIF-2α inhibition used to assess on-target activity^13,16^. Pharmacokinetic and pharmacodynamic modelling predicted that casdatifan at a dose of 20 mg QD would produce a more than 70% reduction in EPO levels from baseline, which is higher than the effect observed with the approved dose of belzutifan (120 mg QD)^2,15^. Notably, casdatifan doses above 20 mg QD showed mean maximal reductions in EPO levels of up to 85% in ARC-14 (ref. ^2^). In summary, casdatifan achieves stronger and more consistent HIF-2α inhibition in tumours.

Here we report findings from ARC-20, a first-in-patient study investigating casdatifan in metastatic ccRCC. The objectives of this report were to evaluate the safety and efficacy of casdatifan monotherapy in heavily pretreated ccRCC and to examine the biomarker–outcome relationships.

## Dose-escalation stage

Between 26 October 2022 and 7 January 2025, in total, 25 patients with advanced solid tumour malignancies were screened and enrolled across casdatifan total daily doses of 20–200 mg. Baseline characteristics and safety data from the dose-escalation stage are presented in Extended Data Table 1 and Extended Data Table 2. None of the patients had previously received treatment with a HIF-2α inhibitor, except for one patient in the 50 mg twice daily (BID) cohort who had received previous belzutifan treatment for 6 months as a fifth-line treatment (discontinued because of progressive disease (PD)). No dose-limiting toxicities (DLTs) were observed up to the 150 mg QD dose. One DLT (grade 3 hypoxia that resolved after treatment interruption) occurred among the first three patients enrolled in the 200 mg QD cohort. According to the 3 + 3 design, three additional patients were required to complete DLT evaluation at this dose. However, among the additional patients enrolled, two experienced non-DLT adverse events resulting in brief on-treatment holds during the DLT window and were therefore DLT-unevaluable, and one patient failed screening. As a result, a maximum tolerated dose could not be determined. Casdatifan at doses of 50 mg QD (capsule), 50 mg BID (capsule), 100 mg QD (tablet) and 150 mg QD (tablet) were selected for further evaluation in the dose-expansion stage.

## Dose-expansion stage

### Patients

**Extended Data Fig. 1:**
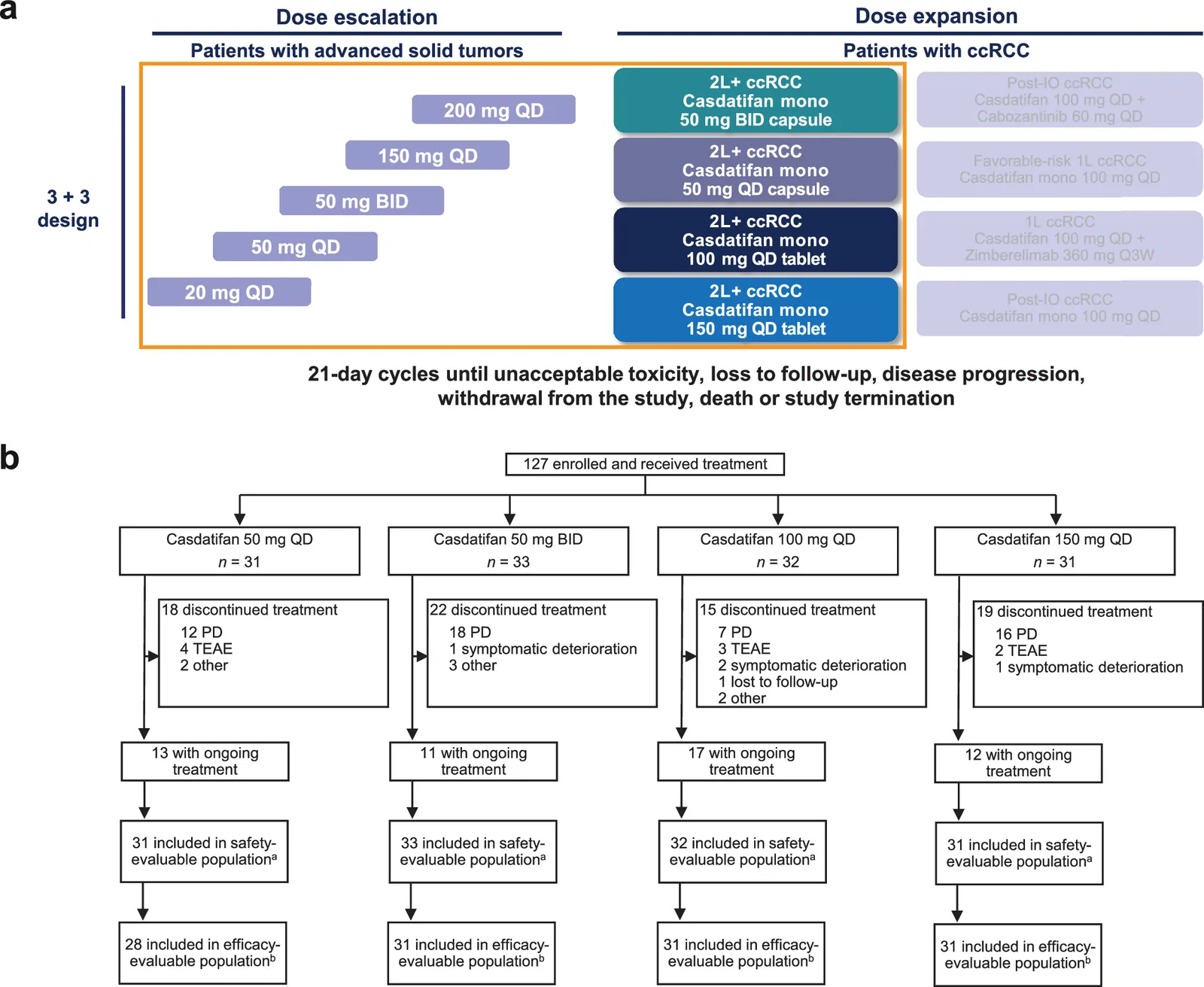
Study design and patient disposition. **a**, Overall study design. **b**, Patient disposition during the dose-expansion stage. Data cutoff date: August 15, 2025. ^a^The safety-evaluable population included all enrolled patients who received ≥1 dose of casdatifan. ^b^The efficacy-evaluable population included all enrolled patients who received ≥1 dose of casdatifan and either had ≥1 post-baseline efficacy assessment or discontinued casdatifan due to PD or death (excluding patients with major protocol deviations impacting efficacy interpretation). 1 L, first-line treatment setting; 2 L+, second-line treatment setting or greater; BID, twice daily; ccRCC, clear cell renal cell carcinoma; IO, immunotherapy; mono, monotherapy; PD, progressive disease; Q3W, every 3 weeks; QD, once daily; TEAE, treatment-emergent adverse event.

Patients were screened and enrolled into casdatifan monotherapy cohorts of the dose-expansion stage between 7 June 2023 and 8 January 2025. As of 15 August 2025, in total, 127 patients with refractory metastatic ccRCC were enrolled, including 31 in the 50 mg QD cohort, 33 in the 50 mg BID cohort, 32 in the 100 mg QD cohort and 31 in the 150 mg QD cohort (Extended Data Fig. 1). We report data from the 100 mg QD cohort (recommended phase III dose (RP3D)) and the total casdatifan monotherapy population, which pooled the 50 mg QD, 50 mg BID, 100 mg QD and 150 mg QD cohorts. At data cut-off, the median (range) study follow-up was 12.4 months (6.3–13.5 months) in the 100 mg QD cohort and 15.5 months (6.3–26.0 months) in the total population; 17 out of 32 patients (53%) in the 100 mg QD cohort and 53 out of 127 patients (42%) in the total population had ongoing casdatifan treatment. Patients most commonly discontinued casdatifan due to progression (7 out of 32 (22%) and 53 out of 127 (42%), respectively) and treatment-emergent adverse events (TEAEs; 3 out of 32 (9%) and 9 out of 127 (7%), respectively).

The median (range) age was 61 years (45–77 years) in the casdatifan 100 mg QD cohort and 63 years (41–82 years) in the total population (Table 1). Most patients had an Eastern Cooperative Oncology Group (ECOG) performance status of 1 (53% and 51%) and had either an intermediate or poor International Metastatic RCC Database Consortium (IMDC) risk score (72% and 71%). In total, 41% of patients in the 100 mg QD cohort and 31% in the total population had liver metastases at baseline. The median (range) number of previous systemic anticancer therapies was 3 (1–6) in the 100 mg QD cohort and 3 (1–11) in the total population; all patients previously received treatment with VEGFR TKIs and anti-PD-L1/anti-PD-1 therapy

**Table 1:** Baseline characteristics (dose-expansion stage)

Characteristic	Casdatifan 100 mg QD (*n* = 32)	Total casdatifan monotherapy population (*n* = 127)
Age (years), median (range)	61 (45–77)	63 (41–82)
Sex, *n* (%)		
Male	27 (84)	96 (76)
Female	5 (16)	31 (24)
Race, *n* (%)		
White	23 (72)	78 (61)
Asian	8 (25)	39 (31)
Black	0	2 (2)
American Indian or Alaska Native	1 (3)	1 (<1)
Unknown or missing	0	7 (6)
ECOG PS, *n* (%)		
0	15 (47)	62 (49)
1	17 (53)	65 (51)
IMDC risk score, *n* (%)		
Favourable	7 (22)	34 (27)
Intermediate	20 (63)	77 (61)
Poor	3 (9)	13 (10)
Unknown or missing	2 (6)	3 (2)
Liver metastases, *n* (%)	13 (41)	39 (31)
Time (years) since initial diagnosis, median (range)	5 (<1–19)	5 (<1–30)
Previous radiotherapy, *n* (%)	22 (69)	65 (51)
Previous systemic anticancer therapies (all settings)		
Number of previous therapies, median (range)	3 (1–6)	3 (1–11)
Patients receiving previous therapies, *n* (%)		
1	6 (19)	20 (16)
2	8 (25)	37 (29)
3	10 (31)	33 (26)
>3	8 (25)	37 (29)
Previous VEGFR TKI/IO therapy^a^, *n* (%)	32 (100)	127 (100)

Data cut-off date, 15 August 2025. PS, performance status; IO, immunotherapy.

^a^Therapies could have been administered in combination or sequentially.

### Safety

The safety-evaluable population included 32 patients in the casdatifan 100 mg QD cohort and 127 patients in the total population, with a median (range) treatment duration of 9.0 months (0.1–13.3 months) and 9.7 months (<0.1–24.5 months), respectively, at the time of data extraction. The most commonly reported TEAEs included anaemia, fatigue, headache and dyspnoea (Table 2). Treatment-related adverse events leading to dose reduction were reported in 22% of patients in the 100 mg QD cohort and 24% in the total population. Most dose reductions due to treatment-related adverse events were from anaemia (9% in the 100 mg QD cohort and 14% in the total population), hypoxia (3% and 7%) and fatigue (6% and 4%). TEAEs leading to death were reported in 6% of patients in the 100 mg QD cohort (*n* = 2; haemoptysis and septic shock) and 2% in the total population (*n* = 3; haemoptysis, septic shock and COVID-19); none were considered to be related to casdatifan.

**Table 2:** Safety overview (dose-expansion stage)

Parameter	Casdatifan 100 mg QD (*n* = 32)	Total casdatifan monotherapy population (*n* = 127)
Treatment duration (months), median (range)	9.0 (0.1–13.3)	9.7 (<0.1–24.5)
Any TEAE, *n* (%)	31 (97)	124 (98)
Occurred in ≥20% of patients		
Anaemia	29 (91)	117 (92)
Fatigue	17 (53)	51 (40)
Headache	10 (31)	32 (25)
Dyspnoea	9 (28)	25 (20)
Grade 3 or higher	16 (50)	76 (60)
Serious	10 (31)	39 (31)
Leading to dose reduction	8 (25)	36 (28)
Leading to treatment discontinuation	3 (9)	11 (9)
Leading to death	2 (6)	3 (2)
Treatment related, *n* (%)	29 (91)	120 (95)
Occurred in ≥10% of patients		
Anaemia	27 (84)	113 (89)
Fatigue	13 (41)	42 (33)
Headache	8 (25)	22 (17)
Dyspnoea	7 (22)	20 (16)
Hypoxia	4 (13)	20 (16)
Dizziness	4 (13)	17 (13)
Grade 3 or higher	12 (38)	62 (49)
Serious	2 (6)	10 (8)
Leading to dose reduction	7 (22)	31 (24)
Leading to treatment discontinuation	1 (3)	4 (3)
Leading to death	0	0
Anaemia		
All grades, *n* (%)	29 (91)	117 (92)
Time (days) to onset of first event of any-grade anaemia, median (range)	22 (8–105)^a^	23 (3–135)^b^
Treatment related, *n* (%)	27 (84)	113 (89)
Grade 3 or higher	8 (25)	52 (41)
Leading to interruption	9 (28)	45 (35)
Leading to dose reduction	3 (9)	18 (14)
Leading to treatment discontinuation	0	0
Hypoxia, *n* (%)		
All grades	5 (16)	23 (18)
Treatment related	4 (13)	20 (16)
Grade 3 or higher	3 (9)	14 (11)
Leading to interruption	3 (9)	18 (14)
Leading to dose reduction	1 (3)	9 (7)
Leading to treatment discontinuation	1 (3)	3 (2)

Data cut-off date, 15 August 2025.

^a^*n* = 29.

^b^*n* = 117.

A total of 84% of patients in the 100 mg QD cohort and 89% in the total population experienced treatment-related anaemia, which led to interruption of treatment in 28% and 35%, and a dose reduction in 9% and 14% of individuals, respectively; no incidences of treatment-related anaemia led to treatment discontinuation. Treatment-related grade ≥3 anaemia occurred in 25% of patients in the 100 mg QD cohort and 41% in the total population and occurred most commonly in the 150 mg QD cohort (52%). Details about timing and use of blood transfusions and EPO-stimulating products are provided in Extended Data Table 3.

A total of 13% of patients in the 100 mg QD cohort and 16% in the total population experienced treatment-related hypoxia, which led to interruption of treatment in 9% and 14%, dose reduction in 3% and 7%, and treatment discontinuation in 3% and 2% of individuals, respectively. Among patients experiencing hypoxia from any cause (100 mg QD cohort, *n* = 5; total population, *n* = 23), 80% and 70%, respectively, received supplemental oxygen therapy, with a median (range) duration of 31 days (3–288 days) and 25 days (1–288 days). The median (range) time to resolution of hypoxia was 12 days (7–32 days) in the 100 mg QD cohort and 11 days (2–689 days) in the total population.

### Efficacy

**Extended Data Fig. 2:**
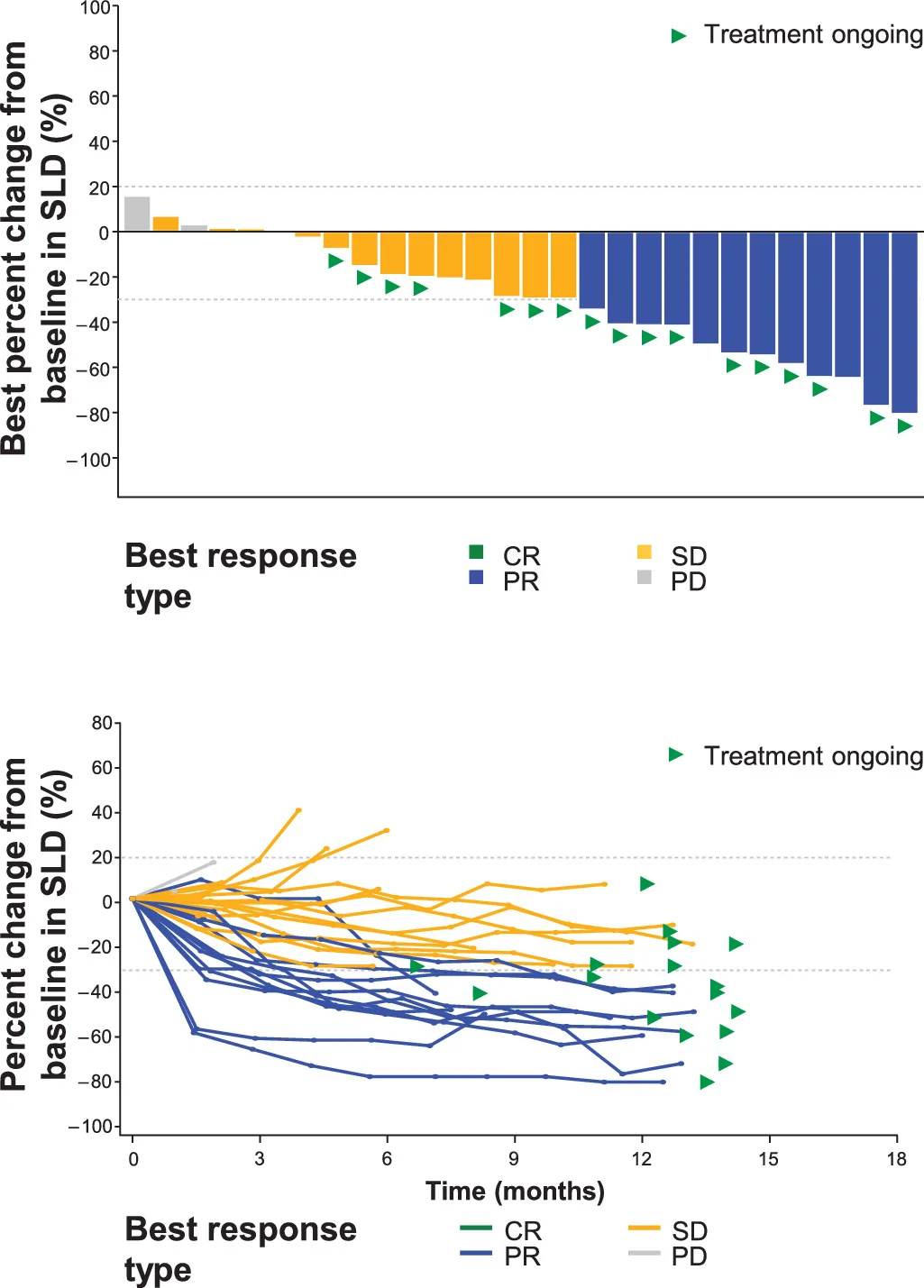
Waterfall plot of confirmed best overall response per RECIST v1.1 and spider plot among patients receiving casdatifan 100 mg QD (dose-expansion stage). Data cutoff date: August 15, 2025. Dashed reference lines indicate a 20% increase or 30% decrease from baseline in the sum of target lesions. CR, complete response; PD, progressive disease; PR, partial response; QD, once daily; SD, stable disease; SLD, sum of lesion diameter.

**Extended Data Fig. 3:**
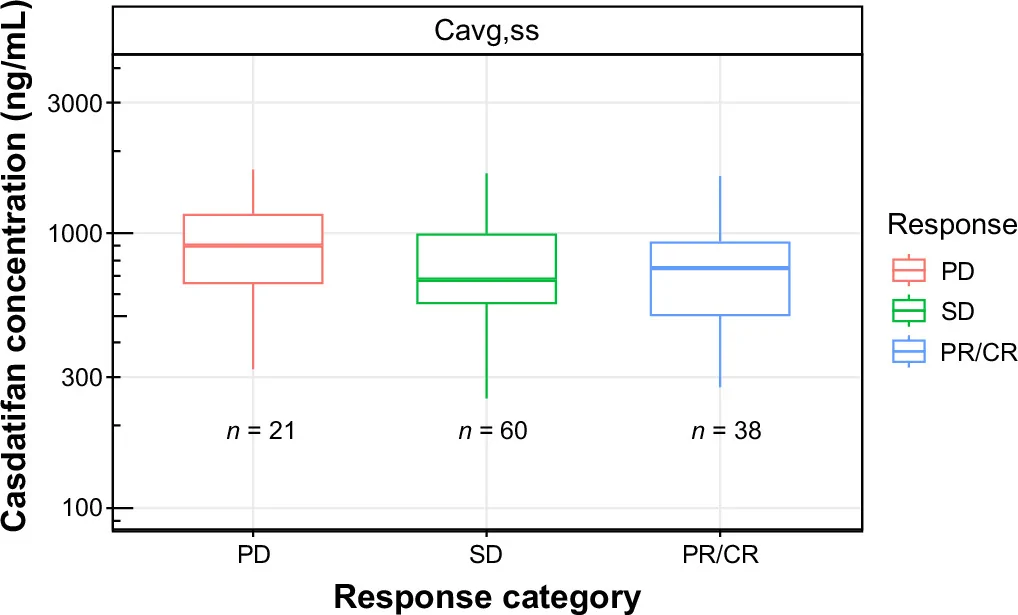
Steady-state plasma concentration in patients with different tumour responses. Boxes represent the interquartile range (25th–75th percentiles), centre lines indicate the median and whiskers extend to the most extreme non-outlier values (defined as 1.5 times the interquartile range). The sample size of each group indicates the number of patients in the ARC-20 study with the specified tumour response. avg, average; CR, complete response; PD, progressive disease; PR, partial response; SD, stable disease; SS, steady state.

The efficacy-evaluable population included 31 patients in the 100 mg QD cohort and 121 patients in the total population. As of 15 August 2025, the confirmed ORR was 35% (95% CI = 19–55%) in the 100 mg QD cohort (all partial responses (PRs)) and 31% (95% CI = 23–40%) in the total population, including one complete response (CR; <1%) and 37 PRs (31%; Extended Data Table 4 and Extended Data Fig. 2). The median (range) time to response was 2.6 months (1.2–9.5 months) in the 100 mg QD cohort and 2.8 months (1.2–13.0 months) in the total population; the disease control rate was 84% (95% CI = 66–95%) and 81% (95% CI = 73–88%), respectively. Continued reductions in tumour size were observed beyond 12 months of treatment (Extended Data Fig. 2). Among patients with IMDC favourable risk, confirmed ORR was 29% (95% CI = 4–71%) in the 100 mg QD cohort and 32% (95% CI = 17–51%) in the total population; those with IMDC intermediate or poor risk had a confirmed ORR of 41% (95% CI = 21–64%) and 31% (95% CI = 22–42%), respectively (Extended Data Table 5). Patients with CR or PR, stable disease (SD) and PD exhibited similar steady-state plasma concentrations of casdatifan (Extended Data Fig. 3).

**Fig. 1:**
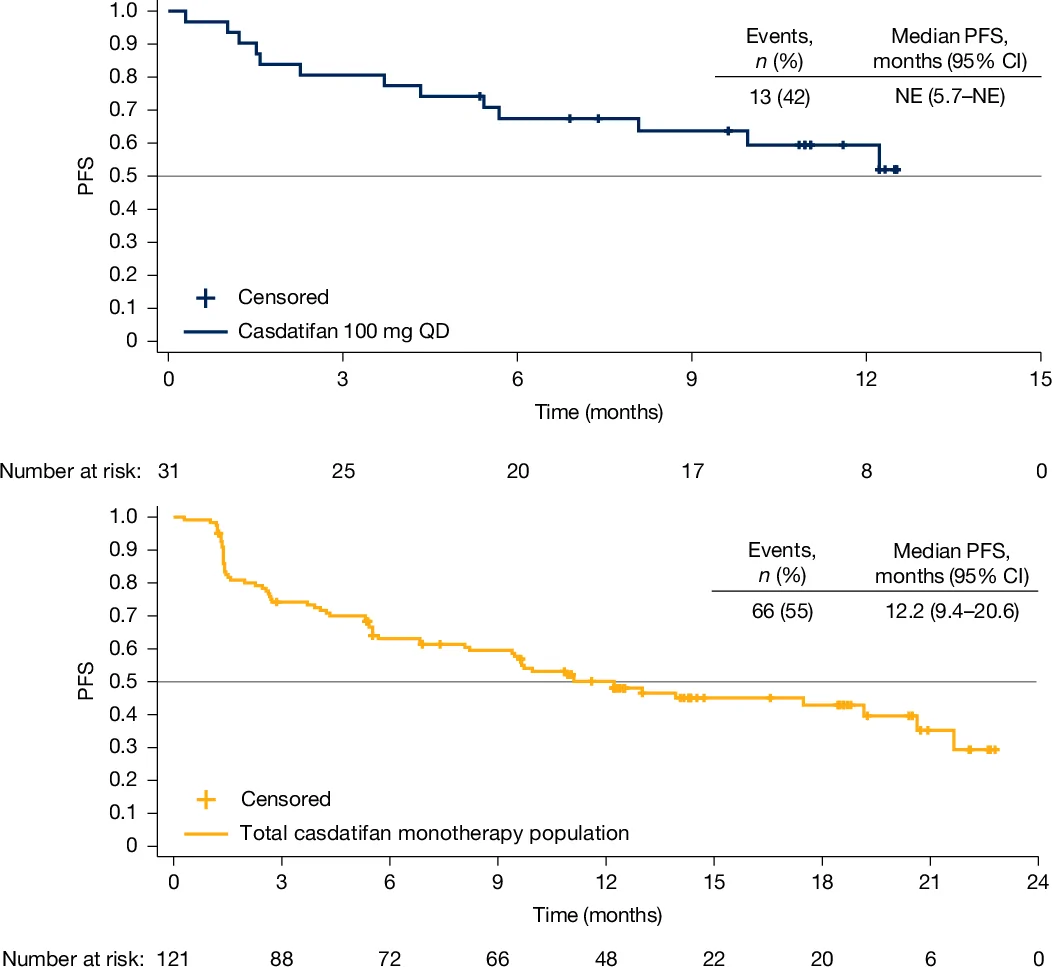
PFS for the casdatifan 100 mg QD cohort and the total casdatifan monotherapy population (dose-expansion stage). Data cut-off date: 15 August 2025. NE, not estimable.

With a median (range) follow-up of 12.4 months (6.3–13.5 months) in the 100 mg QD cohort and 15.5 months (6.3–26.0 months) in the total population, the median PFS was not estimable (95% CI = 5.7%–not estimable) in the 100 mg QD cohort and 12.2 months (95% CI = 9.4–20.6%) in the total population (Fig. 1). The landmark PFS rates in the 100 mg QD cohort and total population were 67% (95% CI = 48–81%) and 63% (95% CI = 54–71%), respectively, at 6 months and 60% (95% CI = 40–75%) and 50% (95% CI = 41–59%), respectively, at 12 months. Among patients with IMDC favourable risk, the median PFS was not estimable in either the 100 mg QD cohort (95% CI = 0.3%–not estimable) or the total population (95% CI = 10.9–not estimable). For those with IMDC intermediate or poor risk, median PFS was not estimable in the 100 mg QD cohort (95% CI = 5.4%–not estimable) and was 9.7 months (95% CI = 5.5–17.5%) in the total population.

## Biomarker analyses

### Reduction in HIF-2α-regulated sEPO

**Fig. 2:**
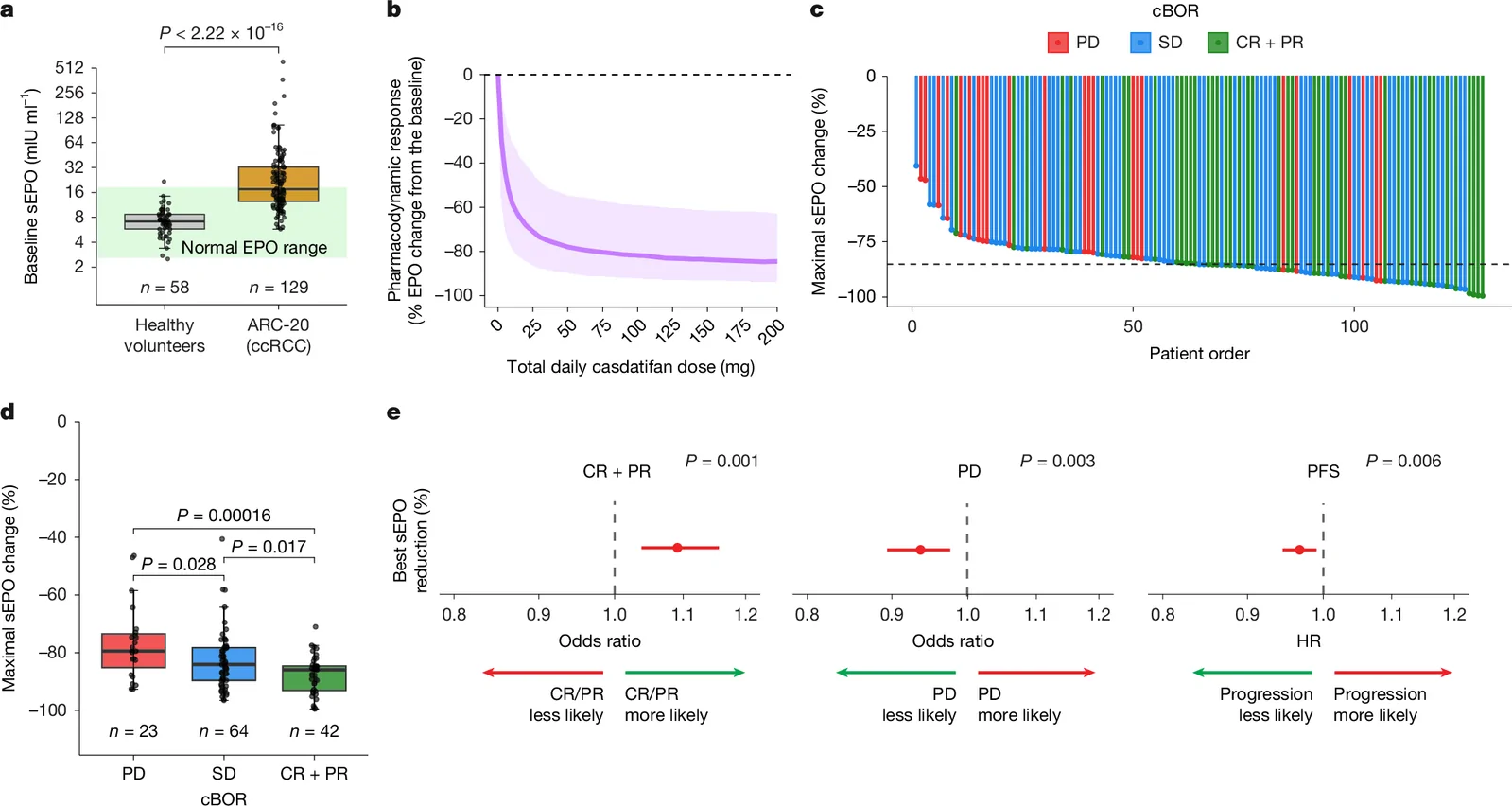
Systemic suppression of HIF-2α-regulated sEPO is associated with clinical benefit with casdatifan. **a**, Baseline sEPO levels in patients with ccRCC from ARC-20 and healthy volunteers from ARC-14. Statistical analysis was performed using the two-sided Wilcoxon ranked-sum test; *P* < 2.22 × 10^−16^. **b**, Pharmacokinetic/pharmacodynamic modelling of sEPO change on day 15 after treatment across casdatifan doses. Data are mean ± 95% CI. **c**, The maximal sEPO change from baseline of 129 patients with ccRCC treated with casdatifan monotherapy, ranked from least to deepest change. Each bar represents data from one patient coloured according to confirmed best overall response (cBOR) status. **d**, The percentage maximal sEPO change in patients with CR/PR, SD and PD. *P* values were calculated using two-sided Wilcoxon ranked-sum tests with no adjustment for multiple comparisons. **e**, The odds ratios (circles), 95% CIs (horizontal lines) and two-sided *P* values from the logistic regression model for PR or CR (left) and PD (middle); and HR (circles), 95% CI (horizontal lines) and two-sided *P* values from the Cox regression model for PFS (right); expressed per 1% increase in maximal sEPO reduction from baseline (*n* = 129 patients from the ARC-20 study). For the box plots, the box limits represent the interquartile range (25th–75th percentiles), the centre lines indicate the median and the whiskers extend to the most extreme non-outlier values (defined as 1.5× the interquartile range).

**Extended Data Fig. 4:**
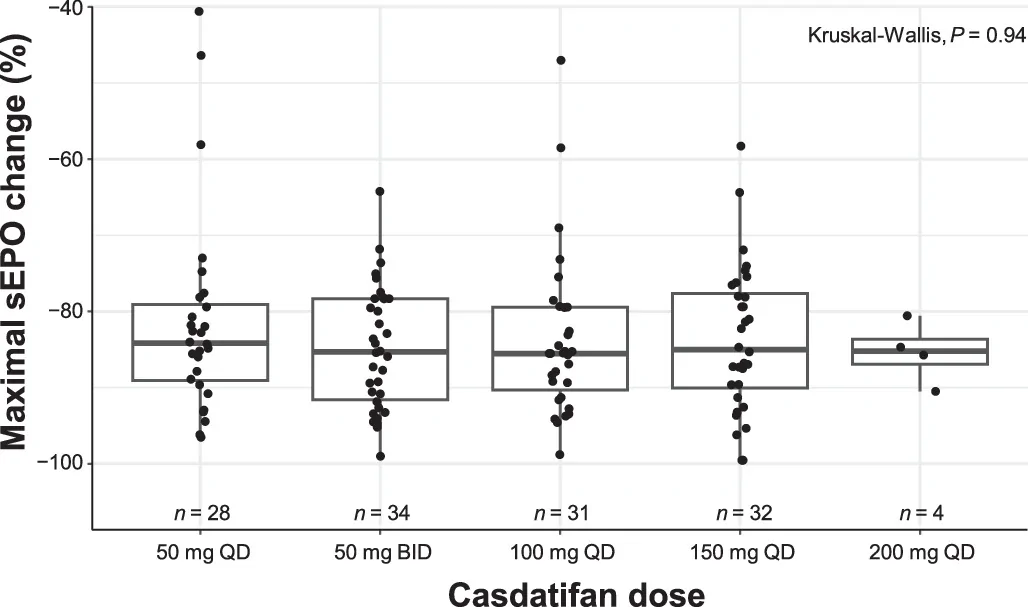
Best sEPO reduction across all doses of casdatifan monotherapy in the ARC-20 ccRCC biomarker-evaluable population. Boxes represent the interquartile range (25th–75th percentiles), centre lines indicate the median and whiskers extend to the most extreme non-outlier values (defined as 1.5 times the interquartile range). BID, twice daily; ccRCC, clear cell renal cell carcinoma; QD, once daily; sEPO, serum erythropoietin.

To understand how sEPO levels in patients with ccRCC differ from physiological ranges, we compared patients from the ARC-20 study (*n* = 129; dose-escalation (*n* = 12) and dose-expansion stages (*n* = 117); casdatifan daily doses ranging from 50 mg to 200 mg) with healthy volunteers from the ARC-14 study^2^. Patients with ccRCC had significantly higher baseline levels of sEPO than healthy volunteers (*P* < 2.22 × 10^−16^), suggestive of tumour-derived increases in circulating EPO (Fig. 2a). sEPO levels dropped substantially on treatment with casdatifan (Fig. 2b), with 71% (92 out of 129) of patients reaching their maximal sEPO reduction (defined as the lowest level achieved on treatment versus baseline levels) during the first cycle of treatment. Across all doses, most patients (88 out of 129, 68%) experienced a maximal sEPO reduction of over 80% (Fig. 2c). Notably, maximal sEPO reduction was comparable across casdatifan daily doses ranging from 50 mg to 200 mg (Extended Data Fig. 4). Deeper sEPO reductions were more frequently indicative of better objective responses (Fig. 2c). In fact, the degree of maximal sEPO reduction (measured as the percentage of decrease on treatment compared with baseline levels) was significantly greater in patients who achieved CR, PR or SD than in those who had PD (PD versus SD, *P* = 0.028; PD versus CR + PR, *P* = 0.00016; SD versus CR + PR, *P* = 0.017; Fig. 2d). When modelled by logistic regression, patients with greater maximal reductions in sEPO had a higher likelihood of CR or PR (odds ratio = 1.09, 95% CI = 1.04–1.16, per per cent difference; *P* = 0.001) and a lower likelihood of PD (odds ratio = 0.94, 95% CI = 0.89–0.98, per per cent difference; *P* = 0.003). Consistently, longer PFS was also associated with a greater maximal sEPO reduction (hazard ratio (HR) = 0.97, 95% CI = 0.95–0.99, per per cent difference; Cox regression, *P* = 0.006; Fig. 2e). Accordingly, each 10% deeper reduction in maximal sEPO would predict a 140% increase in the relative odds of tumour response and a 28% decrease in the relative risk of tumour progression or death.

The association between maximal sEPO reduction and clinical outcome also remained significant after adjustment for known ccRCC prognostic risk factors such as sex, age, IMDC risk (favourable versus intermediate/poor) and ECOG performance status. The *P* values for logistic regression multivariable models were *P* = 0.004 and *P* = 0.007 for likelihood of CR/PR and PD, respectively, and *P* = 0.004 for likelihood of PFS in the Cox regression multivariable model. These data suggest that the magnitude of sEPO reduction was associated with the degree of clinical benefit observed with casdatifan treatment in the ARC-20 study.

### t*EPO* mRNA expression

**Fig. 3:**
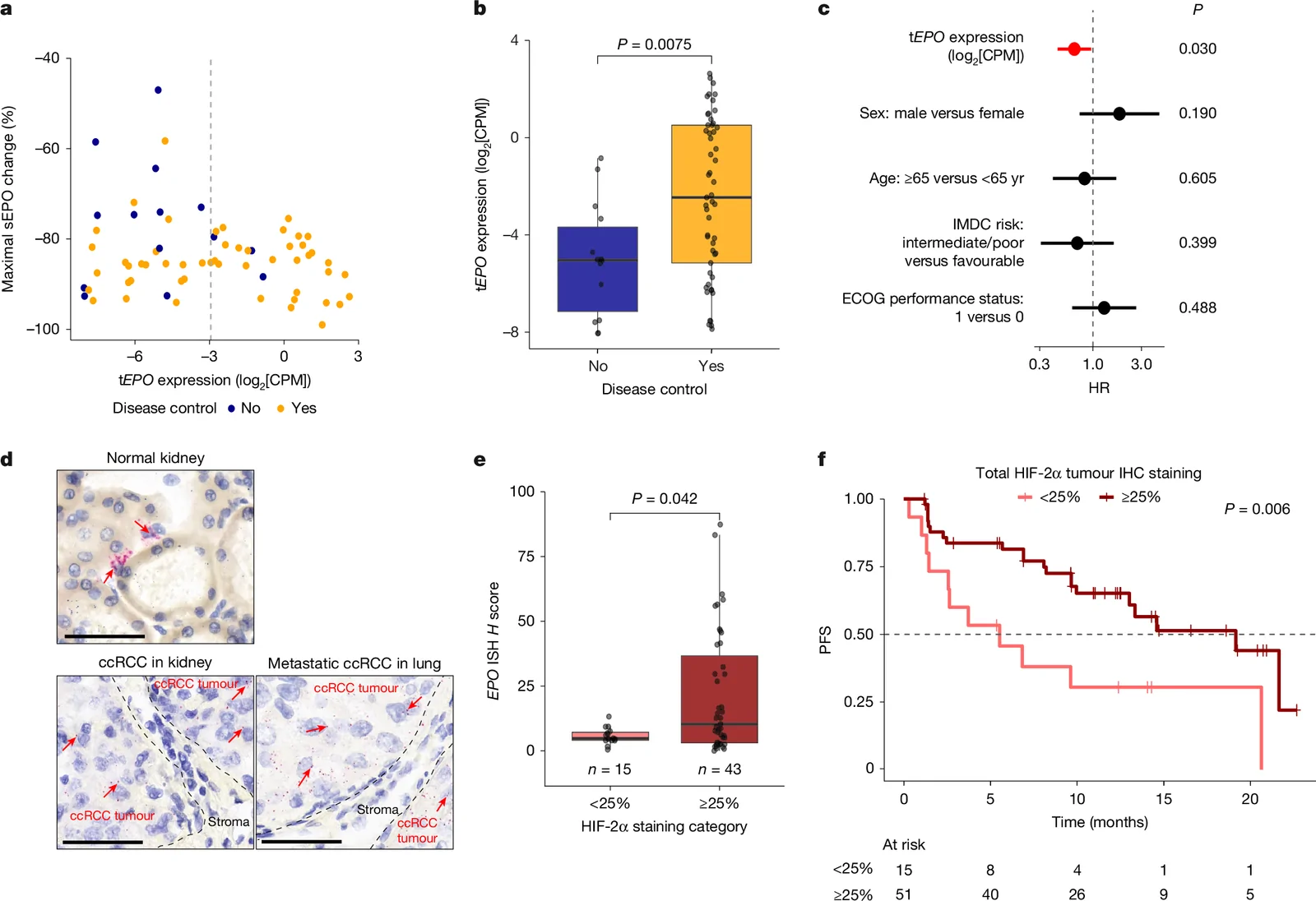
t*EPO* mRNA is linked to HIF-2α dependency and clinical benefit with casdatifan. **a**, The correlation between maximal sEPO change and baseline t*EPO* mRNA expression. *n* = 66. The dashed line represents the median t*EPO* expression. **b**, t*EPO* expression in patients who had disease control (*n* = 53) versus those who did not (*n* = 14). *P* values were calculated using two-sided Wilcoxon ranked-sum tests. **c**, The HRs (circles), 95% CIs (horizontal lines) and two-sided *P* values from the multivariable analysis using a Cox regression model to evaluate the association of t*EPO* expression, sex, age, IMDC risk groups and ECOG performance status against PFS. *n* = 64. **d**, Representative *EPO* RNA ISH images from ccRCC tumours in the kidney (bottom left), ccRCC lung metastasis (bottom right) and normal kidney (top left). Scale bar, 50 µm. **e**, *EPO* RNA ISH scores in patients with at least 25% HIF-2α IHC staining versus patients with less than 25% IHC staining. *P* values were calculated using two-sided Wilcoxon ranked-sum tests. **f**, Kaplan–Meier survival curves comparing the PFS of patients with HIF-2α tumour IHC staining positivity ≥25% versus <25%. The log-rank *P* value is shown on the graph. For the box plots, the box limits represent the interquartile range (25th–75th percentiles), the centre lines indicate the median and whiskers extend to the most extreme non-outlier values (defined as 1.5× the interquartile range). CPM, counts per million.

We next examined whether baseline tumour mRNA levels of *EPO* (t*EPO*), a direct target of HIF-2α, were associated with sEPO decreases on treatment and subsequently with clinical benefit. Patients with high t*EPO* mRNA expression (at least median levels; *n* = 34) consistently showed maximal sEPO reduction of over 75% with casdatifan treatment (Fig. 3a), with only three patients (9%) having primary PD. By contrast, of those with low t*EPO* (*n* = 33), 11 patients had primary PD (33%). Similarly, t*EPO* mRNA expression was significantly elevated in patients with disease control (*n* = 53) compared with patients who experienced PD (*n* = 14) (median log_2_-transformed fold change = 2.58, *P* = 0.0075; Fig. 3b). In a multivariable analysis examining multiple baseline characteristics (sex, age, ECOG performance status and IMDC risk score), t*EPO* mRNA expression was predictive of significantly improved PFS with casdatifan treatment (*P* = 0.030; Fig. 3c).

To assess the cellular origin of t*EPO*, we performed *EPO* mRNA in situ hybridization (ISH) analysis of 59 available samples. These results showed that carcinoma cells, but not adjacent stromal cells, were the primary source of t*EPO* mRNA, regardless of whether the carcinoma cells were obtained from primary kidney or metastatic tumour sites (Fig. 3d). This indicates that t*EPO* levels and their potential contribution to circulating EPO may reflect dysregulated HIF-2α activity in metastatic ccRCC cells.

To further examine the relationship between the clinical benefit of casdatifan and HIF-2α biology in the tumour, we investigated whether t*EPO* mRNA expression was associated with tumour HIF-2α protein levels using a proprietary HIF-2α immunohistochemistry (IHC) assay. Of patients with both HIF-2α IHC and *EPO* mRNA ISH available, we observed that those with at least 25% tumour HIF-2α IHC staining (the cut-off was selected on the basis of a natural breakpoint distinguishing low versus high staining) had significantly higher levels of t*EPO* mRNA in tumour cells (ISH tumour *H*-score) than those with less than 25% tumour HIF-2α IHC staining (*n* = 43 and *n* = 15, respectively; *P* = 0.042; Fig. 3e). Notably, patients with at least 25% tumour HIF-2α IHC staining had significantly longer PFS than those with less than 25% tumour HIF-2α IHC staining (*P* = 0.006; Fig. 3f). Moreover, we investigated the relationship between HIF-1α IHC staining in a similar manner and observed no association between HIF-1α expression and primary PD rate or PFS (data not shown). Overall, these results point to a link between HIF-2α biology in the tumours of patients with ccRCC and clinical benefit with casdatifan treatment.

### Efficacy in HIF-2α-dependent ccRCC

To better understand genomic and transcriptomic features associated with clinical outcomes with casdatifan treatment, we investigated mutations in genes that are commonly altered in ccRCC (*VHL*, *PBRM1*, *SETD2* and *BAP1*) using whole-exome sequencing (WES) and transcriptome analysis. Of 54 patients with available WES data, potential *VHL* impact (single-nucleotide variant and/or copy loss) was identified in 85% (46 out of 54). Moreover, mutations in *PBRM1* were identified in 28% of patients (15 out of 54), *SETD2* in 19% of patients (10 out of 54) and *BAP1* in 9% of patients (5 out of 54). *VHL* mutations, biallelic *VHL* loss and *PBRM1* mutations were not associated with responses to casdatifan. Notably, 4 out of 54 (7%) patients had no identifiable mutations in *VHL*, *PBRM1*, *SETD2* or *BAP1* and did not experience primary PD. Taken together, these findings suggest that mutation status alone is not a sufficient indicator of response to casdatifan.

**Fig. 4:**
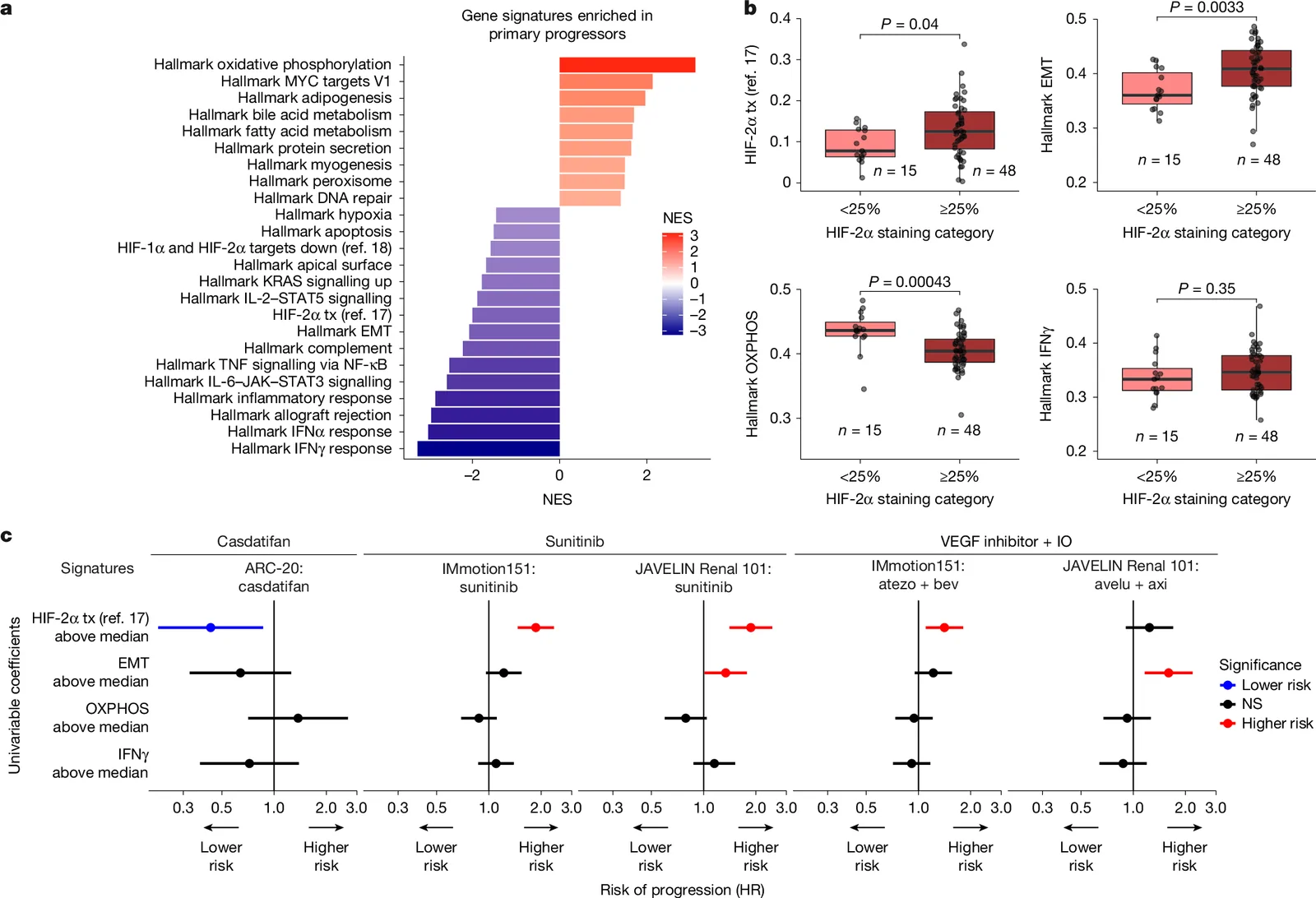
HIF-2α-dependent ccRCC tumours underperform on current therapies, supporting exploration of casdatifan-based combination strategies. **a**, The normalized enrichment score (NES) of gene sets significantly enriched in patients with rapid PD (positive scores) versus patients with disease control (negative scores). **b**, Selected gene set ssGSEA scores in patients with at least 25% HIF-2α IHC staining versus patients with less than 25% IHC staining. For the box plots, the box limits represent the interquartile range (25th–75th percentiles), the centre lines indicate the median value and the whiskers extend to the most extreme non-outlier values (defined as 1.5× the interquartile range). *P* values were calculated using two-sided Wilcoxon ranked-sum tests. **c**, The HRs (circles) and 95% CIs (horizontal lines) of a univariable Cox proportional hazard model with a false-discovery rate threshold of 0.05, comparing the PFS of patients with high versus low ssGSEA scores (by median) of select gene sets across clinical trials of different treatments: ARC-20 (casdatifan), IMmotion151 (sunitinib (*n* = 416) and atezolizumab (atezo) plus bevacizumab (bev) (*n* = 407) arms) and JAVELIN Renal 101 (sunitinib (*n* = 372) and avelumab (avelu) plus axitinib (axi) (*n* = 354) arms). Blue indicates that a higher signature score is associated with lower risk of progression (longer PFS). Red indicates that a higher signature score is associated with higher risk of progression (shorter PFS). NS, not significant; OXPHOS, oxidative phosphorylation; tx, transcriptional.

Gene set enrichment analysis (GSEA) was performed on bulk transcriptomic data from 67 patients using published Hallmark and HIF-2α-related gene signatures^17,18^ (Fig. 4a). Patients with disease control had high expression of transcriptional signatures consistent with engagement of multiple HIF-2α-regulated biological pathways^17–20^, including hypoxia and previously reported HIF-2α-regulation signatures, as well as Hallmark inflammation and epithelial–mesenchymal transition (EMT)-related gene signatures (Fig. 4a). By contrast, patients with disease progression had higher expression of the Hallmark oxidative phosphorylation signature^21^, which is known to be suppressed by HIF activation and hypoxia^17,21,22^ (Fig. 4a).

Patients with at least 25% tumour HIF-2α IHC staining expressed significantly higher levels of the HIF-2α transcriptional signature^17^ (*P* = 0.04) and the Hallmark EMT signature (*P* = 0.0033) and lower levels of the oxidative phosphorylation signature (*P* = 0.00043; Fig. 4b). Although the IFNγ pathway was enriched in tumours from patients exhibiting disease control, expression did not appear to be associated with HIF-2α protein levels (Fig. 4b) or predictive of PFS (Fig. 4c).

**Extended Data Fig. 5:**
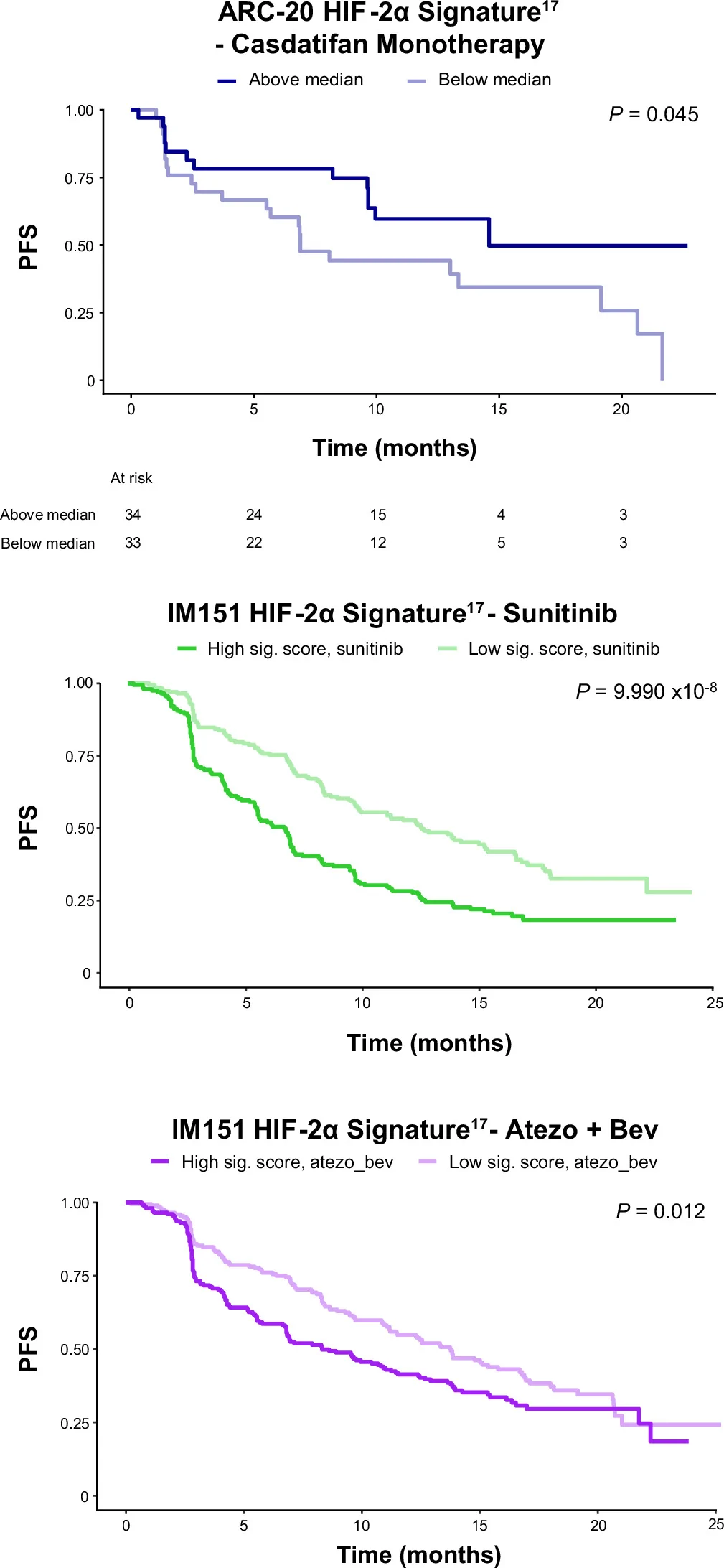
Kaplan-Meier PFS probability analysis by HIF-2α signature expression in ARC-20 and IM151. *P* values were calculated using log-rank test. atezo, atezolizumab; bev, bevacizumab; HIF, hypoxia-inducible factor; IM151, IMmotion151; PFS, progression-free survival; sig, signature.

Finally, we explored the predictive value for PFS of the HIF-2α transcriptional signature^17^ in the ARC-20 study testing the HIF-2α inhibitor casdatifan and available transcriptomic datasets from the phase III IMmotion151 (ref. ^23^) and JAVELIN Renal 101 (ref. ^24^) studies in ccRCC. Each patient was assigned a signature score using single-sample GSEA (ssGSEA), and we compared patients who were above and below the median for each gene expression signature using the univariable Cox proportional hazard models. In the ARC-20 study, high expression of the HIF-2α transcriptional signature was associated with improved PFS with casdatifan monotherapy (HR for risk of progression, 0.43, 95% CI = 0.21–0.86, *P* = 0.018; Fig. 4c and Extended Data Fig. 5). By contrast, high expression of the same HIF-2α signature was associated with decreased PFS among patients receiving sunitinib in IMmotion151 (HR for risk of progression, 1.86, 95% CI = 1.47–2.37, *P* = 3.52 × 10^−7^; Extended Data Fig. 5) and JAVELIN Renal 101 (HR for risk of progression, 1.87, 95% CI = 1.41–2.48, *P* = 0.012) and among patients receiving atezolizumab + bevacizumab in IMmotion151 (HR for risk of progression, 1.41, 95% CI = 1.10–1.81, *P* = 0.006; Extended Data Fig. 5). Moreover, patients with high Hallmark EMT expression (enriched in HIF-2α-dependent tumours) experienced decreased PFS in both the sunitinib (HR = 1.34, 95% CI = 1.01–1.77, *P* = 0.041) and avelumab + axitinib (HR = 1.60, 95% CI = 1.16–2.20, *P* = 0.004) arms of JAVELIN Renal 101, whereas patients with EMT-high tumours in ARC-20 trended towards improved PFS on casdatifan (Fig. 4c). Consistent with its mechanism of action, these findings suggest that casdatifan has the potential to improve outcomes in combination with current treatment regimens that have diminished clinical benefit in the subgroup of patients with highest HIF-2α activity.

## Discussion

Here we report the results from the ARC-20 study evaluating casdatifan monotherapy in patients with heavily pretreated metastatic ccRCC, a population with limited therapeutic options and high unmet need^5,25^. In the dose-escalation stage, no DLTs were observed up to a dose of 150 mg QD, with a single DLT reported in the 200 mg QD cohort. In the dose-expansion stage, casdatifan monotherapy showed encouraging clinical activity and durable responses across cohorts. Findings from the ARC-20 study, along with those from the ARC-14 study^26^, support the selection of casdatifan 100 mg QD as the RP3D. To further characterize the dose–response relationship, we evaluated doses both above (150 mg QD) and below (50 mg QD) the RP3D, as well as a BID regimen (50 mg BID). The analysis of these patients as a total pooled population allowed a more comprehensive evaluation of safety and clinical activity in a sample size larger (*n* = 127) than that observed in typical phase I studies. Notably, compared with previous HIF-2α inhibitors, casdatifan exhibited a deeper on-target effect at lower or equivalent exposures^13,26^, which is consistent with its preclinical potency and favourable pharmacokinetics and pharmacodynamics^2,27,28^.

The clinical activity observed with casdatifan monotherapy in patients with previous immunotherapy and TKI treatments was encouraging, with an observed ORR of 31–35%. The median PFS was not estimable at over 12 months median follow-up in the 100 mg QD cohort and was over 12 months with 15 months median follow-up in the total population. For context, belzutifan monotherapy in similarly pretreated patient populations has shown an ORR of 21% in the phase I LITESPARK-001 study^29^, an ORR of 19% in the phase II LITESPARK-013 study^30^ and an ORR of 23% with a median PFS of 5.6 months in the phase III LITESPARK-005 study^12^. Taken together, clinical outcomes observed with casdatifan in the ARC-20 study are encouraging for this patient population and may suggest an enhanced clinical effect compared with previous HIF-2α inhibitors, potentially reflecting a more favourable pharmacodynamic profile. However, cross-trial comparisons should be interpreted cautiously owing to differences in study design, patient populations, treatment history and follow-up durations.

IMDC risk category is an important prognostic tool for assessing outcomes in ccRCC^31^. The majority of patients in ARC-20 had intermediate or poor IMDC risk; among these patients, the confirmed ORR was 41% in the 100 mg QD cohort and 31% in the overall population. These response rates are numerically higher than those reported in a recent real-world, retrospective analysis^32^, which showed ORRs of 23–32% for second-line ccRCC in these IMDC risk categories. Given that the patients in the ARC-20 study received a median of 3 previous lines of therapy (with a maximum of 11), these findings suggest that casdatifan delivers clinically meaningful activity even in a high-risk, refractory population.

This is one of the first systematic and comprehensive studies linking patient-level tumour biology and clinical activity for a HIF-2α inhibitor. We investigated sEPO dynamics, t*EPO* expression, HIF-2α dependency and HIF-2α inhibitor clinical activity in a population of advanced refractory ccRCC. We showed that the magnitude of sEPO reduction while receiving treatment is not only a robust pharmacodynamic biomarker but is also strongly associated with clinical benefit. These findings provide additional evidence on the mechanism of action of casdatifan as a potent HIF-2α inhibitor^33^ and highlight the importance of sEPO changes as a peripheral pharmacodynamic end point and a potential marker of tumour HIF-2α biology. Consistent with observations in the periphery, baseline t*EPO* mRNA expression was higher in patients who experienced better outcomes from casdatifan. Patients with higher levels of tumour HIF-2α protein expressed higher levels of t*EPO* as measured by ISH and experienced longer PFS, linking sEPO, t*EPO* and HIF-2α biology to clinical benefit with casdatifan. This is consistent with results from a biomarker analysis of LITESPARK-013, in which a high HIF-2α tumour positivity score was associated with improved PFS with belzutifan treatment^34^. Transcriptomic GSEA analysis evaluating signalling pathways differentially enriched in patients with divergent clinical outcomes provided further insights into HIF-2α dependency and the clinical activity of casdatifan. Notably, expression of a HIF-2α gene signature^17^ was associated with significantly longer PFS in ARC-20 but shorter PFS in external datasets across multiple other non-HIF-2α regimens. The expression of additional gene signatures related to HIF-2α activity, such as the Hallmark EMT signature^35^, are also consistent with the idea that higher levels of HIF-2α-related signalling may portend worse survival for patients when treated with therapies directed at alternative pathways. Overall, these data suggest that most advanced ccRCC tumours have high levels of HIF-2α and HIF-2α-dependent biological pathways and may potentially respond favourably to casdatifan monotherapy and casdatifan-based combinations.

The safety profile of casdatifan was consistent with that of the therapeutic class^11–13^ and was clinically manageable. Anaemia of any grade occurred in most patients, and treatment-related grade ≥3 anaemia was observed in approximately one-quarter of those receiving 100 mg QD. Importantly, anaemia was well managed with intermittent supportive EPO-stimulating agents, occasional transfusions and limited dose reductions and did not lead to discontinuation in the 100 mg QD cohort. Hypoxia, another class-related TEAE^11–13^, occurred in approximately 15% of patients in the 100 mg QD cohort, with fewer than 10% experiencing a treatment-related grade ≥3 event and only one discontinuation of casdatifan due to treatment-related hypoxia. Overall, the duration of treatment interruption due to these on-target TEAEs was minimal and clinically manageable. These findings are generally consistent with those reported for belzutifan across multiple trials^11–13^.

Currently, HIF-2α inhibition is a therapeutic option for patients with advanced ccRCC after treatment with a PD-L1/PD-1 inhibitor and a VEGFR TKI^10,36^. The antitumour activity of casdatifan in this setting appears to be improved compared with previous HIF-2α inhibitors, although additional studies are needed to validate findings and inform casdatifan’s place in the current treatment landscape. The phase III randomized PEAK-1 study (NCT07011719) is evaluating casdatifan plus cabozantinib in previously treated advanced ccRCC. Based on the ongoing clinical development program, casdatifan is likely to be positioned in combination with current standard of care rather than as monotherapy.

Limitations of this study should be considered when interpreting the findings. First, ARC-20 was a single-arm, early-phase study. Without a randomized comparator, estimates of antitumour efficacy may be influenced by unmeasured confounders, including variable disease trajectories, differences in previous treatment responsiveness and the natural heterogeneity of refractory ccRCC. Moreover, this study included patients with ECOG performance status of 0 or 1, previous treatment with both immunotherapy and VEGFR TKI, and adequate organ and marrow function, which may limit generalizability to the broader real-world population of patients with metastatic ccRCC. Geographical representation was also limited to the USA, Australia and South Korea, and racial diversity was modest, further constraining external applicability. Finally, archival tissue samples were used rather than requiring on-trial biopsies. Overall, the findings from ARC-20 are encouraging and support continued clinical development of casdatifan. The ARC-20 study is ongoing, and extended follow-up will provide additional insights into the durability of clinical benefit among responders and the activity of casdatifan in combination with other therapies. Future studies (such as PEAK-1) will validate these findings in a larger study population and investigate connections between exploratory biomarkers and clinical outcomes.

In conclusion, casdatifan achieved meaningful and durable responses with a manageable safety profile in a heavily pretreated metastatic ccRCC population. Biomarkers of HIF-2α inhibition paralleled clinical benefit, linking a mechanistically precise pharmacodynamic marker to antitumour efficacy.

## Methods

### Study design and patients

ARC-20 is an ongoing, multicentre, phase I, open-label, dose-escalation and dose-expansion study (Extended Data Fig. 1) conducted at clinical sites across the USA, Australia and South Korea (Supplementary Table 1). ARC-20 was designed to establish the safety and biologically active dose of casdatifan and to directly link the depth of HIF-2α inhibition to pharmacodynamic effects, tumour biology and clinical benefit in refractory metastatic ccRCC.

Safety decisions were guided by a dose-escalation committee (DEC) and a safety-review committee (SRC). The DEC reviewed cohort-level safety and DLT data to guide dose escalation and dose clearance for expansion, while the SRC evaluated safety during the dose-expansion stage. The DEC and SRC were not independent of the study sponsor. Final decisions regarding continuation or termination of the trial or its components were made under sponsor governance and were informed by committee recommendations. Access to interim information was restricted to authorized study personnel and committee members and was kept confidential. Further details, including the composition of the DEC and SRC, are provided in the study protocol (Supplementary Information).

The ARC-20 study was registered on 7 September 2022 with clinicaltrials.gov (NCT05536141). Before the study was initiated, an institutional review board or independent ethics committee at each study site approved the protocol and other relevant study materials (Supplementary Table 1; the protocol is provided in the Supplementary Information). All of the patients or their legally authorized representatives provided written informed consent before enrolment; patients were not compensated monetarily for their participation in this trial. This study was conducted in full accordance with the Declaration of Helsinki, Council for International Organizations of Medical Sciences, the International Council for Harmonisation and all regional laws and regulations.

Preliminary findings from ARC-20 were previously presented at the EORTC-NCI-AACR Symposium, 23 to 25 October 2024, Barcelona, Spain^37^; American Society of Clinical Oncology Genitourinary Cancers Symposium, 30 May to 3 June 2025, Chicago, Illinois^38^; Kidney Cancer Research Summit, 17 to 18 July 2025, Boston, Massachusetts^39^; and American Society of Clinical Oncology Genitourinary Cancers Symposium, 26 to 28 February 2026, San Francisco, California^40^.

#### Dose-escalation stage

The dose-escalation stage was performed using a standard 3 + 3 design with a 21-day DLT evaluation period. The safety, tolerability and preliminary efficacy of casdatifan monotherapy were evaluated at doses ranging from 20–200 mg QD in patients with solid tumours for whom no standard-of-care therapies were available. The starting dose of 20 mg QD was selected on the basis of the totality of nonclinical data and findings from the first-in-human study of casdatifan in healthy participants (ARC-14)^2^, which suggested manageable toxicity at this dose. The dose range of 20–200 mg QD was selected to enable evaluation of a broad range of exposures and to inform clinical development. Patients were enrolled on a rolling basis in sets of three. After three patients in a set were dosed, enrolment was paused until the 3-week treatment cycle was completed. Dosing then resumed by adding another set of three to the same dose level or by escalating to the next dose level, according to the 3 + 3 design. The maximum tolerated dose corresponded to the highest dose at which the DLT incidence was below 33%. Further details on methodology for the dose-escalation stage are reported in Supplementary Table 2. Specific criteria for dose discontinuation, dose modification and dosing delays are provided in the study protocol (Supplementary Information).

#### Dose-expansion stage

Dose-expansion cohorts were designed to further characterize the safety, tolerability and efficacy of casdatifan (as monotherapy and as combination therapy) at dose levels selected on the basis of on data from the preceding dose-escalation stage. This report focused on patients who received casdatifan monotherapy. Dose-expansion cohorts were permitted to initiate before completion of dose escalation, provided that the dose had cleared DLT evaluation. The patients were eligible to participate if they were aged at least 18 years with histologically confirmed metastatic ccRCC, received previous treatment in the locally advanced or metastatic setting with an anti-PD-L1/anti-PD-1 inhibitor and a VEGFR TKI, were HIF-2α inhibitor naive, had a creatinine clearance of at least 40 ml min^−1^ and had at least 1 measurable tumour lesion based on RECIST v.1.1. The patients must also have had an ECOG performance status of 0 or 1 and adequate organ and marrow function. Further details on patient eligibility are shown in Supplementary Table 3.

The patients in each cohort received casdatifan monotherapy in 21-day cycles and continued treatment until occurrence of unacceptable toxicity, disease progression, loss to follow-up, withdrawal from the study, death or study termination, whichever occurred first. Stopping rules for each dose-expansion cohort included the occurrence of any of the following: treatment-related grade 5 adverse events in at least 10% of patients; two or more treatment-related, non-laboratory grade 4 adverse events; or grade 4 treatment-related hypoxia or anaemia in at least 25% of patients. Specific criteria for dose discontinuation, dose modification and dosing delays were prespecified in the study protocol (Supplementary Information).

### Assessments

The primary end point was safety, which included investigator evaluation of the type, incidence, causality, severity (based on the National Cancer Institute Common Terminology Criteria for Adverse Events v.5.0) and seriousness of TEAEs. TEAEs were coded using the Medication Dictionary for Regulatory Activities v.25.0. Anaemia was defined according to Common Terminology Criteria for Adverse Events v.5.0. Safety was monitored from the first dose of study treatment until 30 days after the last dose or until initiation of new systemic anticancer therapy, whichever occurred first. The secondary end point was the ORR, defined as the proportion of patients with a confirmed best overall response of either CR or PR as determined by the investigator according to RECIST v.1.1. Investigator-assessed PFS, an exploratory end point, was defined as the date of the first dose to first occurrence of disease progression per RECIST v.1.1 or death from any cause, whichever occurred first. Disease control rate was defined as the proportion of patients who achieved CR, PR or SD. Tumour imaging was performed at screening, at the start of cycle 3 and at every 6 weeks thereafter for up to 1 year of treatment. From 1 year to 2 years, imaging was performed every 9 weeks and every 12 weeks thereafter. Tumour assessments continued until disease progression, study discontinuation or initiation of alternative anticancer therapy.

### Statistical analysis

Safety assessments were performed on all patients who were enrolled and received at least 1 dose of study treatment. The efficacy-evaluable population included all patients who were enrolled, received at least 1 dose of study treatment and either had at least 1 post-baseline efficacy assessment or discontinued study treatment due to PD or death, excluding patients with major protocol deviations which may impact interpretation of efficacy end points. Data from the dose-escalation stage are presented in Extended Data Table 1 and Extended Data Table 2 and by each dose level. The dose-escalation sample size was determined using a rule-based 3 + 3 escalation method targeting a DLT threshold of 33%. Data from the dose-expansion stage are presented for the 100 mg QD cohort and a total group comprising all monotherapy cohorts (50 mg QD, 50 mg BID, 100 mg QD and 150 mg QD). The planned sample size for dose expansion was approximately 30 patients per cohort, which was selected to obtain a preliminary assessment of antitumour activity based on the ORR. With this sample size, the lower bound of the Clopper–Pearson 90% CI would be expected to exclude historical control response rates with differences on the order of 15% to 20%. If any individual expansion dose demonstrated preliminary signs of efficacy and an acceptable safety profile as determined by the SRC, an additional 30 patients could enrol into the cohort. Safety data were summarized by reporting the number and percentage of patients in each cohort or group experiencing TEAEs. The 95% CIs for the ORR were calculated using the Clopper–Pearson exact method. Where applicable, statistical tests were two-sided with an *α* of 0.05. The median PFS was estimated using Kaplan–Meier methodology. Patients without documented disease progression who were still alive at the time of analysis were censored at the time of their last tumour assessment. Patients with no post-baseline tumour assessments were censored at the date of the first dose. Patients with death or progression after more than one missed tumour assessment were censored at the last tumour assessment before the missed tumour assessment. Statistical analyses were conducted using SAS v.9.4.

### Exploratory biomarker analyses

#### sEPO sample collection and assay

Serum samples were collected at pre-dose of every cycle until cycle 6 followed by pre-dose of every even cycle until cycle 18 from every patient. Additional post-dose serum was also collected at 4 h and 8 h at cycle 1 day 1 and cycle 1 day 15. Serum samples were stored at −20 °C until being tested for EPO concentration at a central laboratory (LabCorp) using the Access EPO assay (A16364) on the Beckman-Coulter DxI800 system. The amount of analyte in the sample was determined from a stored, multi-point calibration curve. sEPO data collected within 28 days after treatment with EPO-stimulating agents and within 4 days after a dose hold or dose reduction of less than half the intended dose were excluded from data analysis.

#### Tumour RNA/DNA-seq, data processing and biomarker analysis

Archival tumour tissue block or tissue slides either from nephrectomy surgery or core needle biopsy were requested for every patient. For blocks, 4–5-µm sections were prepared in a nuclease-free manner at the tissue central laboratory, Cell Carta.

Macrodissection was performed if the tissue samples contained less than 80% tumour content, as determined by a pathologist on the basis of a haematoxylin and eosin-stained section. The numbers of slides needed for nucleic acid extraction were estimated on the basis of the estimated tumour area and percentage tumour.

RNA and DNA dual extraction was performed using the Thermo Fisher Scientific MagMAX FFPE DNA/RNA Ultra Kit. Tumour formalin-fixed paraffin-embedded (FFPE) samples were profiled using RNA sequencing (RNA-seq) and WES.

RNA-seq libraries were prepared using the Illumina TruSeq RNA Exome kit. RNA-seq was performed on Illumina systems using 150 bp paired-end, dual-index reads. The samples were sequenced to a depth of 100 million paired-end (200 million total) reads. RNA-seq data quality control was performed using FASTQC v.0.11.9. Reads were aligned to GRCh38 human reference genome (Ensembl v.104) using the STAR aligner v.2.6.1d, and quantification was performed using salmon v.1.4.0 with GENCODE v38 annotations. Count data were further normalized using library size adjustment, trimmed median of *M*-values and voom transformed. Limma v.3.64.1 with voom precision weights was used for differential gene expression analysis, with filtering performed to exclude low-expressed genes using the filterByExpr function. GSEA was performed on the ranking of *t*-statistics from the differential gene expression analyses using the Fast GSEA package v.1.34.0. Gene set scores were calculated using ssGSEA implemented in the Gene Set Variation Analysis (GSVA) package v.2.4.7. ssGSEA calculated enrichment scores based on the cumulative distribution of gene expression ranks within each sample.

WES was performed using the IDT xGen Exome panel at 200× coverage on tumour-derived FFPE DNA and at 100× coverage on PAXgene DNA, which served as the germline control. Raw data were processed using the nf-core sarek workflow v.3.4.2 and additional custom Nextflow pipelines. Reads from FASTQ files were trimmed and split using fastP v.0.23.4, and quality control was performed using MultiQC v.1.25.1. Trimmed reads were aligned to GRCh38 using bwa-mem v.0.7.17.post1188 using the default parameters. Somatic variants were identified using three different variant callers: Mutect2 v.4.5.0.0, Strelka v.2.9.10 and VarScan v.2.4.4, followed by ensemble variant calling to filter for variants supported by two or more callers. Variants were annotated using VEP v.108. Tumour mutational burden was calculated from ensemble variants, with variant allele frequencies ≥ 5%, and adjusted for target interval windows.

#### EPO RNA ISH assay

EPO ISH assay was performed using Automated RNAscope 2.5 LSx assay-Red (322150, Advanced Cell Diagnostics, Newark, California) on the BOND RX Autostainer (Leica Biosystems). FFPE tissues were sectioned at 4 μm, air-dried overnight and then baked for 20 min at 65 °C. Commercially available RNAscope LS 2.5 Probe, *Homo sapiens* EPO probe (414208), was used according to the manufacturer’s instructions using the default RNAScope 2.5 LSX Red ISH protocol. This staining procedure consisted of antigen retrieval for 15 min at 95 °C, LSx enzyme pretreatment for 15 min and probe hybridization at 42 °C for 2 h, followed by several rounds of probe amplification and chromogen/counterstain detection steps. Slides were air-dried and mounted using EcoMount (EM897L, Biocare Medical) and scanned at ×40 magnification using the 3DHistech Pannoramic MIDI II Scanner (3DHISTECH).

#### HIF-2α IHC assay and scoring

FFPE tissues from ARC-20 samples were sectioned at a 4-μm thickness using standard histological procedures. The slides were dried overnight at room temperature and subsequently baked for 1 h at 65 °C. After baking, the slides were dewaxed in xylene and rehydrated through a graded ethanol series using a DAKO coverstainer (DAKO, Agilent Technologies). IHC was carried out using a DAKO Autostainer Link 48 platform (DAKO, Agilent Technologies) using standard histological staining procedures. After quenching of endogenous peroxidase activity, the slides were incubated with either a rabbit anti-human HIF-2α antibody (1:100, 59973, Cell Signaling Technologies) or an isotype antibody (1:400, 3900S, Cell Signaling Technologies) as a control. Antibody specificity was validated using HIF-2α-engineered cell lines, western blotting, peptide competition assays and a multiorgan normal human tissue array. Detection of the antibodies was carried out using MACH3 rabbit polymer HRP (M3R531L, Biocare Medical), followed by Flex Dab+ substrate (SM803, DAKO, Agilent Technologies). Slides were counterstained with Tacha’s haematoxylin (Biocare Medical), dehydrated and coverslipped using the DAKO coverstainer. Once dried, the slides were scanned using a 3DHistech Pannoramic MIDI II Scanner (3DHISTECH), and the percentage tumour HIF-2α positivity was scored visually by a board-certified pathologist.

#### Biomarker clinical association analysis and statistics

The biomarker-evaluable population included patients with ccRCC in the dose-escalation and dose-expansion monotherapy cohorts who received daily doses of casdatifan from 50 mg to 200 mg and had available, analysable serum or tissue biomarker data. The biomarker-evaluable population also included two patients who experienced non-evaluable confirmed best overall response due to PD before their first on-treatment scan. These two patients were classified as experiencing PD for biomarker analyses. Clinical association of various biomarkers was analysed using the R programming language (v.4.3.1). For assessing associations between outcomes (PR/CR, PD and PFS) and maximum sEPO change on treatment, both logistic and Cox regression models were fit using both univariable and multivariable approaches to confirm that demographic variables do not explain the observed differences in outcome based on sEPO reduction. Both univariable and multivariable *P* values are significant and reported in the text. For comparing two groups, Wilcoxon ranked-sum tests were used to derive *P* values. Kaplan–Meier survival analyses for HIF-2α IHC staining of at least 25% or less than 25% were conducted with log-rank *P* values calculated using the survival v.3.8-3 and survminer v.0.5.0 R packages. Visualization of the differences between defined groups was performed using ggplot2 v.4.0.2 and base R plotting functions. Forest plots were generated for maximum EPO reduction and gene expression signatures of HIF-2α and others across different clinical trials to determine the HR and CI of each biomarker in relation to each other. Univariable tests were performed and reported for all analyses with the exception of the Cox regression modelling that was performed to assess the relationship between t*EPO* RNA-seq and PFS. For this analysis, sex, age (as a categorical variable; 18–64 years versus ≥65 years), IMDC risk (intermediate/poor versus favourable) and ECOG categories (0 versus 1) were used as demographic covariables during modelling.

RNA expression and somatic mutation data from IMmotion151 were generated by Genentech/gRED and used under agreement with Genentech. Expression data from JAVELIN Renal 101 are publicly available^41^. Gene set scores were calculated using ssGSEA implemented in the GSVA package v.2.4.7.

Where applicable, statistical tests were two-sided with an *α* of 0.05. No adjustments were performed for multiple comparisons, with the exception of GSEA analysis performed on Hallmark and literature-based gene signatures across primary progression groups. For this analysis, a false-discovery rate threshold of 0.05 was used.

### Reporting summary

Further information on research design is available in the Nature Portfolio Reporting Summary linked to this article.

## Online content

Any methods, additional references, Nature Portfolio reporting summaries, source data, extended data, supplementary information, acknowledgements, peer review information; details of author contributions and competing interests; and statements of data and code availability are available at 10.1038/s41586-026-10718-x.

## Supplementary information

Supplementary Information Supplementary Tables 1–3 and the study protocol.

Reporting Summary

Peer Review File

## Figures and Tables

**Extended Data Table 1:**
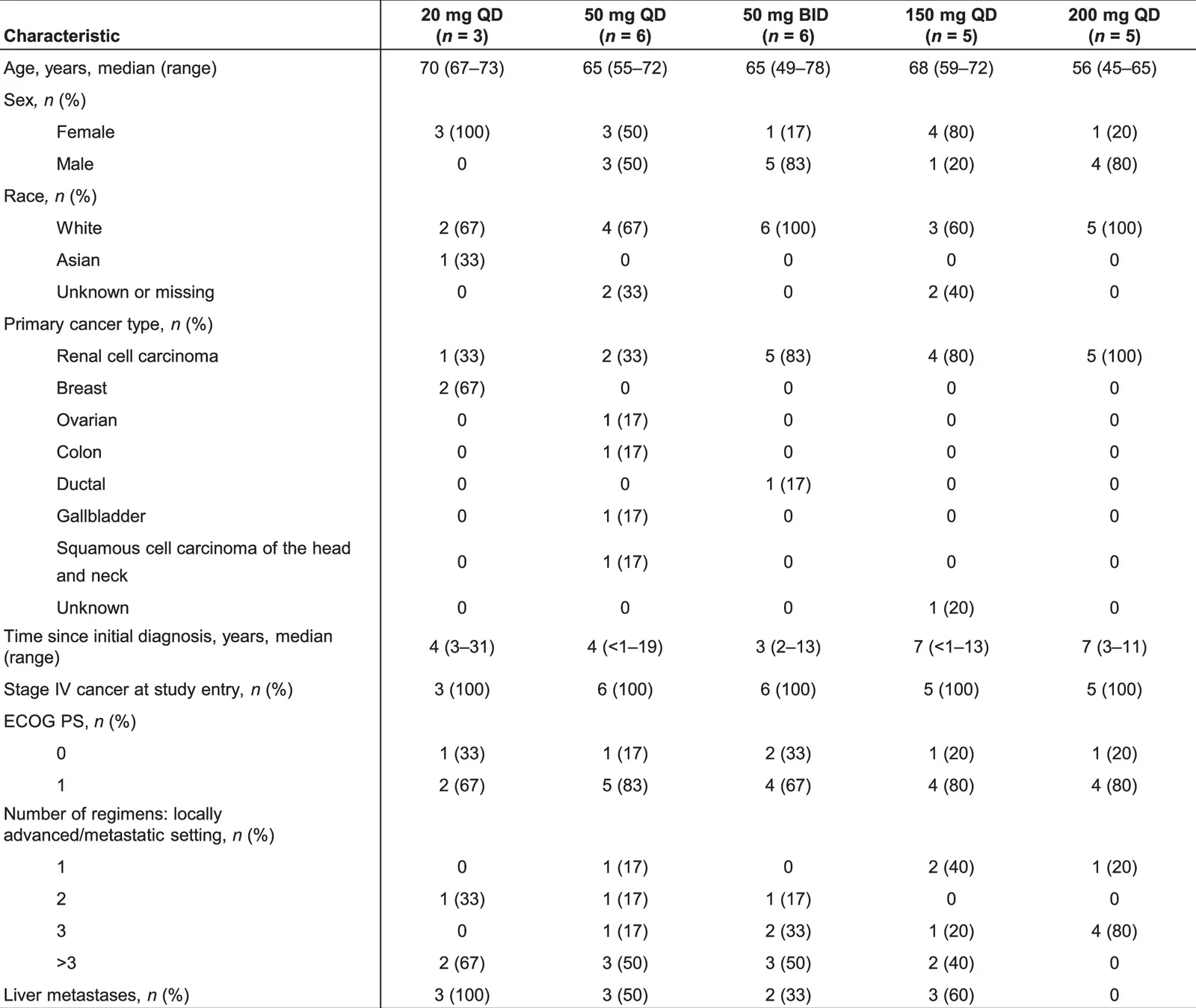
Baseline characteristics of patients enrolled in the dose-escalation stage

Data cutoff date: August 15, 2025. BID, twice daily; ECOG PS, Eastern Cooperative Oncology Group performance status; QD, once daily.

**Extended Data Table 2:**
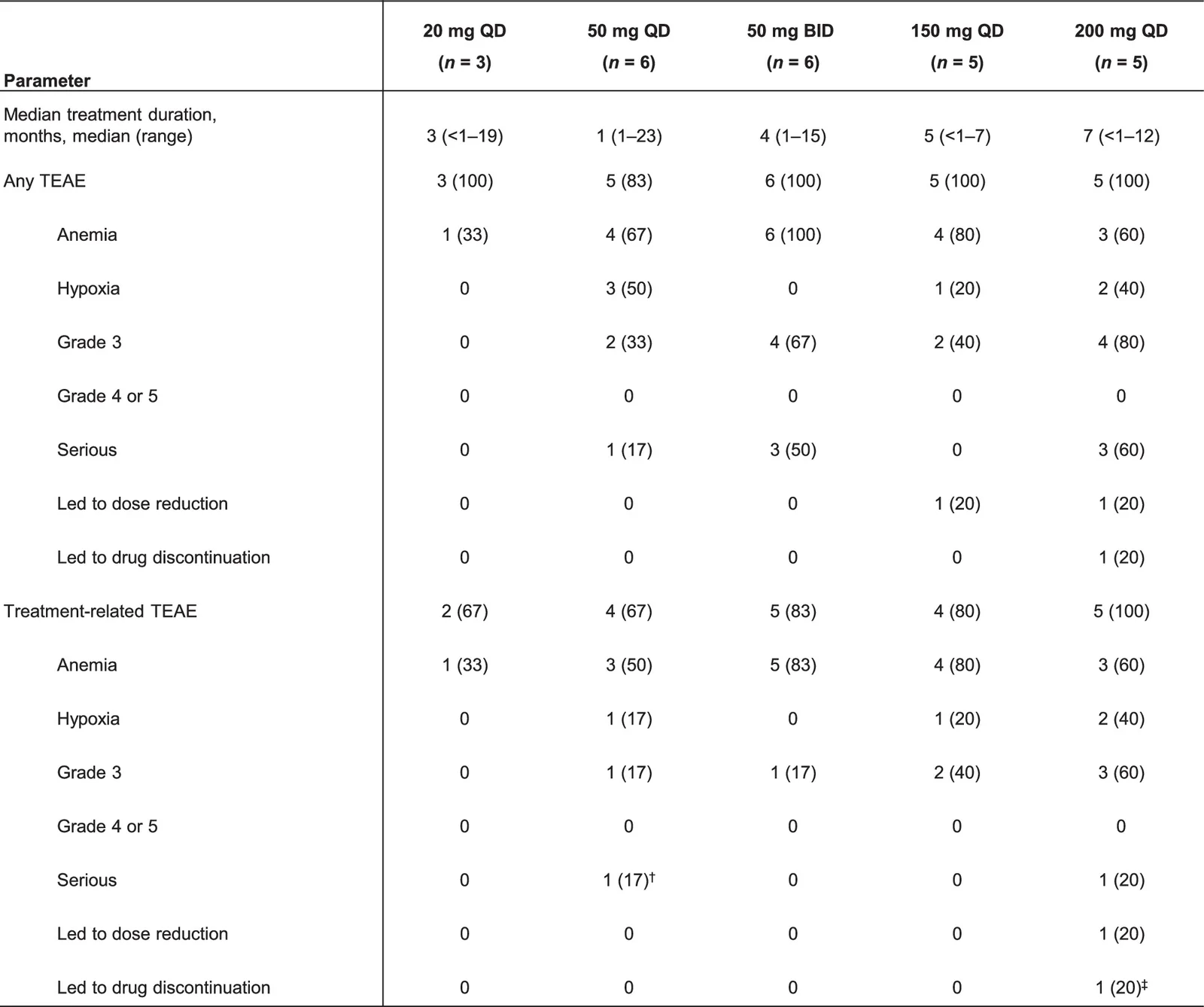
Safety overview of the dose-escalation stage (safety-evaluable population^∗^)

Data cutoff date: August 15, 2025. Data are n (%) unless otherwise specified. ^∗^All patients who enrolled and received any amount of study treatment. ^†^TEAE was dyspnoea. ^‡^Grade 3 TEAE of hypoxia. BID, twice daily; QD, once daily; TEAE, treatment-emergent adverse event.

**Extended Data Table 3:**
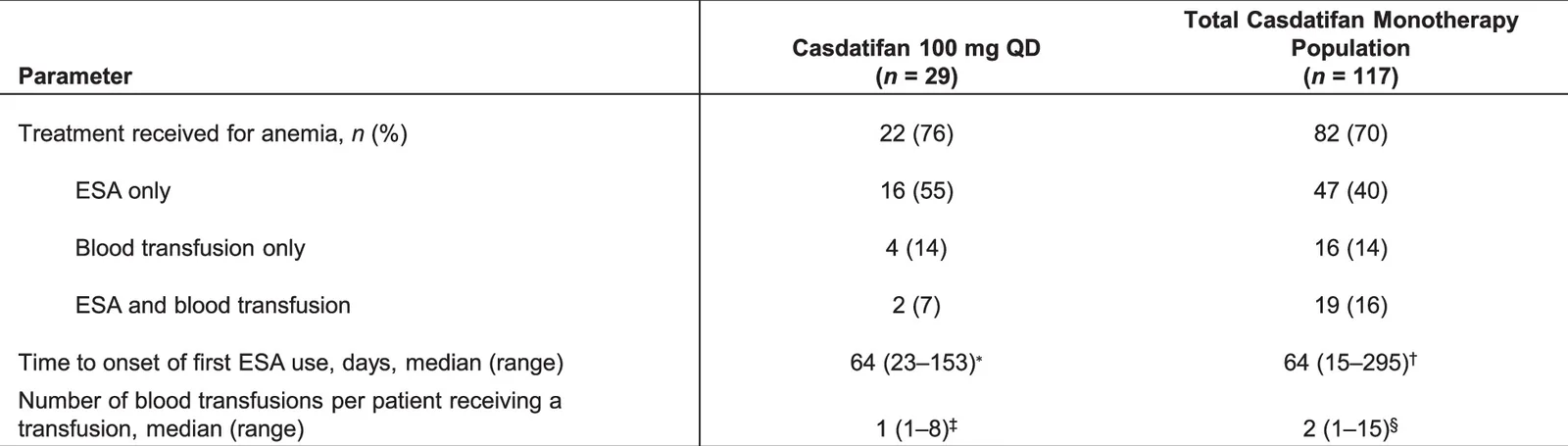
Management of patients with ≥ 1 event of any-grade anaemia (dose-expansion stage)

Data cutoff date: August 15, 2025. ^∗^*n* = 18; ^†^*n* = 66; ^‡^*n* = 6; ^§^*n* = 35. This table only includes patients who experienced anaemia as a treatment-emergent adverse event. ESA, erythropoietin-stimulating agent; QD, once daily.

**Extended Data Table 4:**
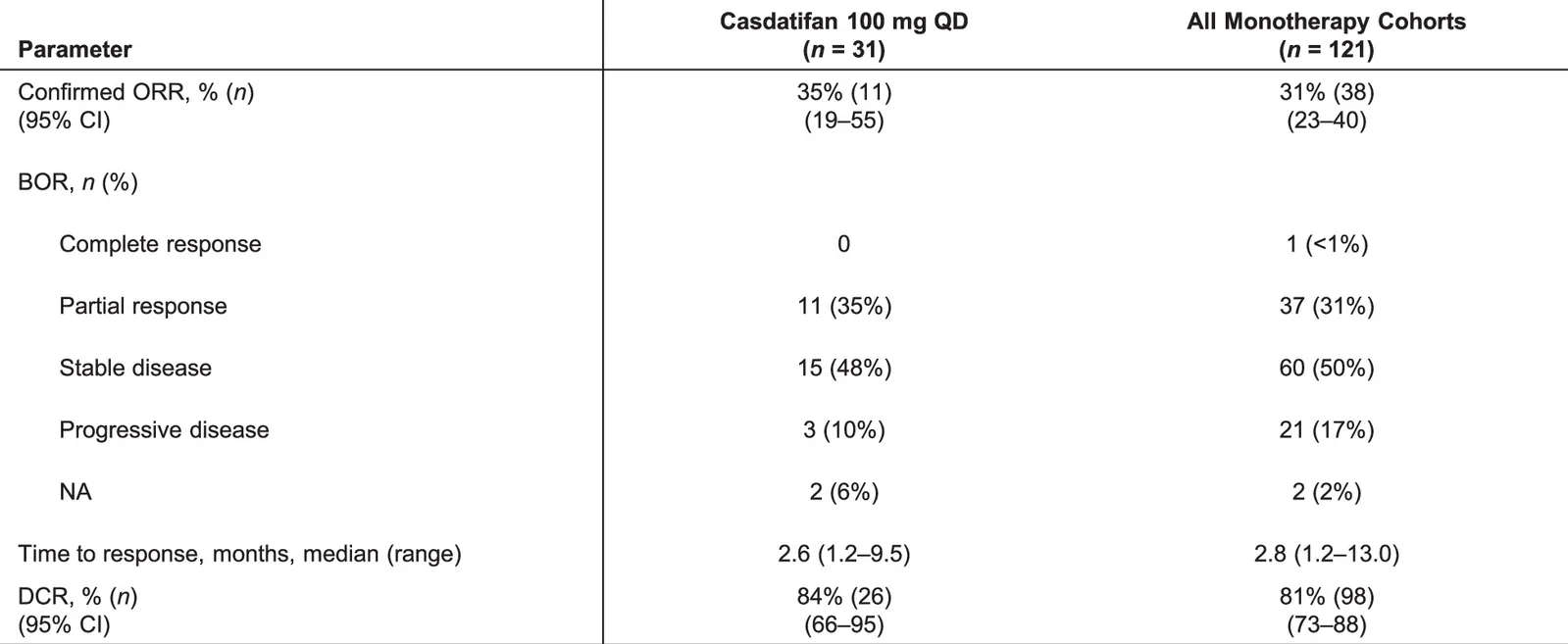
Tumour response (dose-expansion stage)

Data cutoff date: August 15, 2025. BOR, best overall response; DCR, disease control rate; NA, not available; ORR, objective response rate; QD, once daily.

**Extended Data Table 5:**
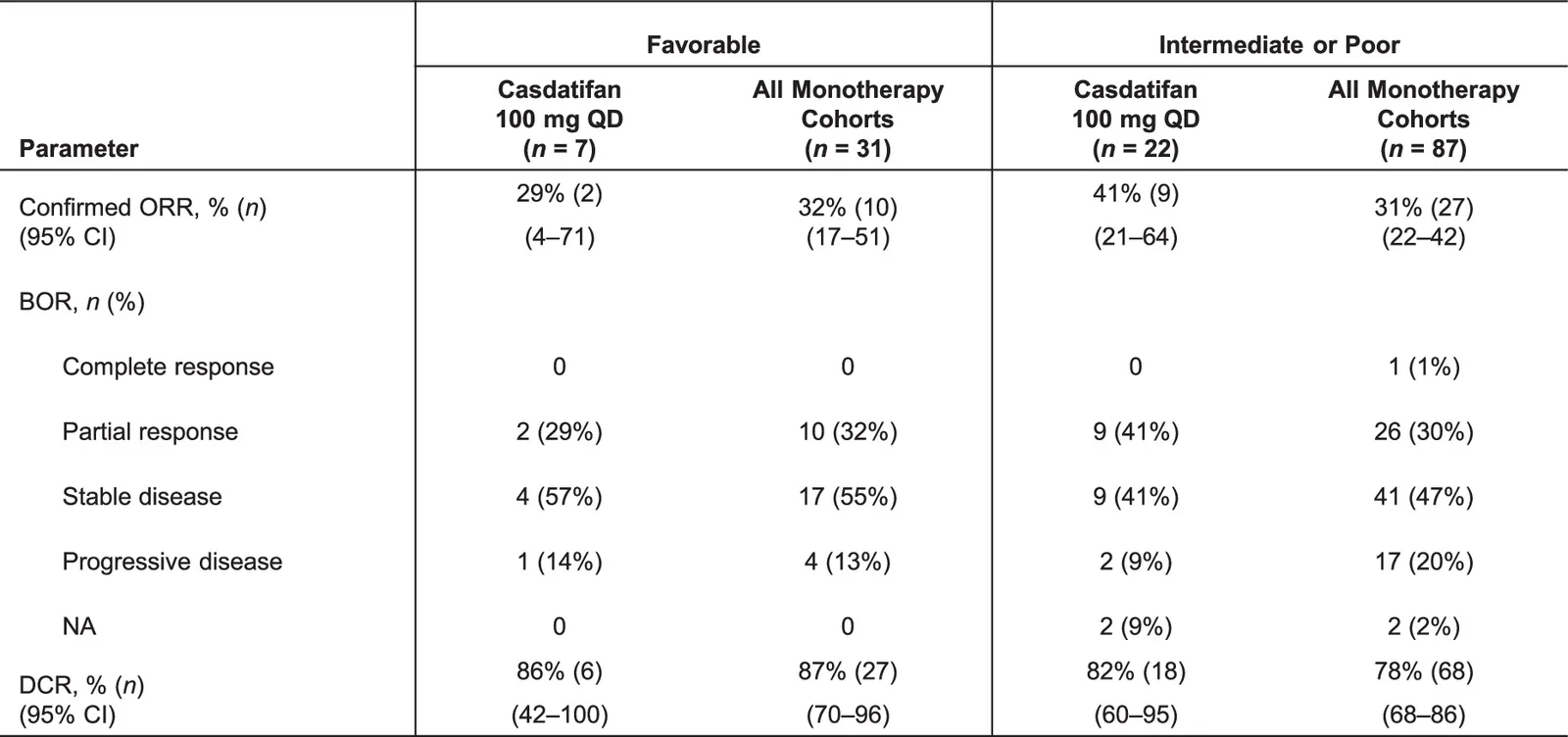
Tumour response by IMDC risk (dose-expansion stage)

Data cutoff date: August 15, 2025. BOR, best overall response; DCR, disease control rate; IMDC, International Metastatic Renal Cell Carcinoma Database Consortium; NA, not available; ORR, objective response rate; QD, once daily.

## Data Availability

Arcus Biosciences is committed to responsible sharing of data from clinical trials sponsored by Arcus Biosciences. Summary and de-identified individual participant data as well as other trial information (protocols, statistical analysis plans and clinical study reports) may be available on request. Arcus will continue to protect the privacy of our clinical trial participants. Requests for data from any qualified researcher who engages in rigorous, independent scientific research will be considered if the clinical trial data are not part of an ongoing or planned regulatory submission. Original data will be available for 12 months, beginning 3 months after approval of the study drug for use in patients or a new indication. For information on the process or to submit a request, visit https://trials.arcusbio.com/our-transparency-policy. The GRCh38 human reference genome is available from https://genome.ucsc.edu. RNA expression and somatic mutation data from IMmotion151 were generated by Genentech/gRED and used under agreement with Genentech from the European Genome Archive (https://ega-archive.org/studies/EGAS00001004353). Expression data from the JAVELIN Renal 101 study are publicly available^41^.

## Code availability

The custom codes used in this study contain confidential information and are therefore not publicly available. Code may be made available on reasonable request, subject to appropriate agreements. For information on the process or to submit a request, visit https://trials.arcusbio.com/our-transparency-policy.
