# Author Correction: Casdatifan shows durable response linked to HIF-2α biology in kidney cancer

**DOI:** 10.1038/s41586-026-10958-x

**Published:** 2026-08-04

**Authors:** Toni K. Choueiri, Jaime Merchán, Amita Patnaik, Alexandra Drakaki, Brian I. Rini, Sun Young Rha, Jae Lyun Lee, Moshe C. Ornstein, Rohit Kumar, Clara Hwang, Yusra Shao, Se Hoon Park, Pedro C. Barata, Bradley A. McGregor, Paul Foster, Jianfen Chen, Melissa Eisen, Hunter Cole, Ben Weeder, Yinghui Guan, Jaskirat Singh, Angelo Kaplan, Soonweng Cho, Richard Markus, Omar Kabbarah, Rana R. McKay

**Affiliations:** 1 Dana-Farber Cancer Institute, Harvard Medical School, Boston, MA USA; 2 University of Miami Sylvester Comprehensive Cancer Center, Miami, FL USA; 3 The START Center for Cancer Research, San Antonio, TX USA; 4 Division of Hematology/Oncology, Department of Medicine, David Geffen School of Medicine, University of California, Los Angeles, Los Angeles, CA USA; 5 Vanderbilt-Ingram Cancer Center, Nashville, TN USA; 6 Yonsei Cancer Center, Yonsei University College of Medicine, Seoul, South Korea; 7 Asan Medical Center, University of Ulsan College of Medicine, Seoul, South Korea; 8 Department of Hematology and Medical Oncology, Cleveland Clinic Taussig Cancer Institute, Cleveland, OH USA; 9 Division of Hematology Oncology, James Graham Brown Cancer Center, University of Louisville, Louisville, KY USA; 10 Department of Internal Medicine, Henry Ford Health System, Henry Ford Cancer Institute, Detroit, MI USA; 11 Karmanos Cancer Center, Detroit, MI USA; 12 Department of Medicine, Samsung Medical Center, Sungkyunkwan University School of Medicine, Seoul, Korea; 13 University Hospitals Seidman Cancer Center, Cleveland, OH USA; 14 Arcus Biosciences, Inc., Hayward, CA USA; 15 Division of Hematology Oncology, University of California, San Diego, San Diego, CA USA

**Keywords:** Targeted therapies, Renal cell carcinoma

Correction to: *Nature* 10.1038/s41586-026-10718-x Published online 1 July 2026

In the version of this article initially published, the name of Jaime Merchán was misspelled (Jamie Merchan) and is now amended in the HTML and PDF versions of the article.

