# Supplementary material for: Casdatifan shows durable response linked to HIF-2α biology in kidney cancer
Source: Nature. 2026 Jul 1;656(8128):734–41. doi: 10.1038/s41586-026-10718-x (PMC13489952; doi:10.1038/s41586-026-10718-x)
Supplement: Supplementary file 1 [file 41586_2026_10718_MOESM1_ESM.pdf]

nature portfolio

https://doi.org/10.1038/s41586-026-10718-x

Supplementary information

Casdatifan shows durable response linked to HIF-2α biology in kidney cancer

In the format provided by the authors and unedited

Supplementary Table 1 | Ethics committee information

Center number	Institution name	IRB name
200101	Macquarie University Hospital	Macquarie University Human Research Ethics Committee
200103	Chris O'Brien Lifehouse	Bellberry Human Resource Ethics Committee
200104	ICON (Ashford) cancer center research	Bellberry Human Resource Ethics Committee
200105	Box Hill Hospital	Eastern Health Office of Research and Ethics
200201	Icahn School of Medicine at Mount Sinai	Mount Sinai School of Medicine IRB #1 - Board B
200202	South Texas Accelerated Research Therapeutics	Salus IRB
200203	Barbara Ann Karmanos Cancer Center	Advarra
200204	University of Louisville Brown Cancer Center	The University of Louisville Institutional Review Board
200205	Sarah Cannon	Castle IRB
200206	Henry Ford Health System	Advarra
200207	Dana-Farber Cancer Institute	Dana-Farber Cancer Institute Institutional Review Board
200208	Nebraska Cancer Specialists - Oncology Hematology West PC	Advarra
200210	Vanderbilt-Ingram Cancer Center	Vanderbilt University Medical Center
200211	Ochsner Health	Ochsner Clinic Foundation Institutional Review Board
200212	University of Miami	University of Miami Institutional Review Board
200214	Emory University	Advarra

200215	University of California Los Angeles	The UCLA Institutional Review Board
200216	Sidney Kimmel Comprehensive Cancer Center at Johns Hopkins	Johns Hopkins Medicine Institutional Review Board
200218	Cleveland Clinic	Cleveland Clinic Institutional Review Board
200219	UPMC Hillman Cancer Center	Advarra
200221	University Hospitals Cleveland Medical Center	University Hospitals Cleveland Medical Center
200222	University of Alabama at Birmingham, O'neal Comprehensive Cancer Center	Advarra
200223	University of California at San Diego	Advarra
200230	Memorial Sloan Kettering Cancer Center	Memorial Sloan Kettering Cancer Center IRB
203401	Vall d'Hebron Institute of Oncology (VHIO)	Drug Research Ethics Committee of HM Hospitales
208201	Samsung Medical Center	IRB of Samsung Medical Center
208202	Seoul National University Bundang Hospital	Seoul National University Bundang Hospital Institutional Review Board
208203	Asan Medical Center	IRB of Asan Medical Center
208204	Severance Hospital, Yonsei University	Yonsei University Health System, Severance Hospital Institutional Review Board

IRB, institutional review board.

Supplementary Table 2 | Dose-escalation stage methodology (additional details)

**Study Design**
A total of five doses of casdatifan were planned for evaluation:<br>• 20 mg per day (2 × 10 mg capsules) taken QD<br>• 50 mg per day (5 × 10 mg capsules) taken QD<br>• 100 mg per day (10 × 10 mg capsules) taken BID (50 mg BID)<br>• 150 mg per day (6 × 25 mg tablets) taken QD<br>• 200 mg per day (8 × 25 mg tablets) taken QD<br><br>The dose-escalation stage followed a standard 3 + 3 design:<br>• If none of the three patients experienced a DLT, escalation proceeded to the next highest dose cohort<br>• If one of three patients experienced a DLT, the cohort was expanded to include six patients<br>• If more than one patient in a cohort of three to six experienced a DLT, the MTD was considered to have been exceeded, and the preceding dose cohort was designated as the MTD<br><br>Evaluation of additional dose levels was allowed based on emerging data on safety and/or pharmacokinetics but could not exceed 200 mg QD or a 100% dose increase from the previous cohort<br><br>For patients who may derive clinical benefit at a dose level lower than the highest dose that is deemed safe and tolerable, intra-patient dose escalation was allowed after discussion with the sponsor's medical monitor if:<br>• Escalation to the next dose level that has been deemed safe was at least 1 dose level below the actively enrolling dose<br>• Patients had signs of clinical benefit (eg, stable disease with some tumor shrinkage, improvement of symptoms, an objective response)<br>• Patients tolerated their current dose level (eg, did not experience a DLT) and had only toxicities considered grade ≤2 during the prior treatment cycle<br><br>Any of the following treatment-related toxicities were considered a DLT unless clearly attributable to underlying malignancy, disease progression or another extraneous cause:<br>• Death<br>• Any liver laboratory abnormality meeting Hy's law criteria, AST or ALT >8 × ULN, regardless of duration or AST or ALT 5–8 × ULN persisting >14 days<br>• Any grade 3 or higher non-hematologic (non-laboratory) toxicity, except nausea, vomiting, diarrhea or fatigue, which qualified as a DLT only if grade ≥3 and lasting >72 hours with appropriate supportive care (for nausea, vomiting or diarrhea) or ≥7 days (for fatigue)<br>• Grade 4 anemia<br>• Febrile neutropenia or grade 4 neutropenia lasting >7 days

- Grade ≥3 thrombocytopenia with clinically significant bleeding or grade 4 thrombocytopenia lasting >7 days
- Grade ≥3 electrolyte abnormality lasting >72 hours (or any grade 3 abnormality with clinical symptoms, regardless of duration)
- Grade ≥3 amylase or lipase elevation associated with clinical manifestations of pancreatitis
- Delay of a subsequent treatment cycle >14 days due to persistent grade ≥2 toxicity

Patient Eligibility Criteria

- Capable of providing signed informed consent
- Aged ≥18 years
- Female patients must not have been pregnant, breastfeeding or planning to become pregnant
- Male patients and female patients of childbearing potential must have been willing to use highly effective contraceptive methods during the treatment period and for 90 days after the last dose of casdatifan
- Pathologically confirmed solid tumor type where no other treatment options were available
- Creatinine clearance ≥40 mL/min
- ≥1 measurable tumor lesion per RECIST v1.1
- ECOG performance status score of 0 or 1
- Patients with hepatitis B and hepatitis C were eligible if they had resolution of infection or were on adequate suppressive antiviral therapy and not at risk for hepatic decompensation
- Patients with HIV were eligible if they had CD4 T cell count ≥350 cells/µL and were receiving adequate suppressive antiviral therapy
- Adequate organ and marrow function:
  - Absolute neutrophil count ≥1.5 × 10^9/L
  - Platelet count ≥100 × 10^9/L
  - Hemoglobin ≥10.0 g/dL
  - AST ≤2.5 × ULN in the absence of hepatic metastasis or ≤5 × ULN in the presence of hepatic metastasis
  - ALT ≤2.5 × ULN in the absence of hepatic metastasis or ≤5 × ULN in the presence of hepatic metastasis
  - Bilirubin ≤1.5 × ULN (except for patients with Gilbert's syndrome who must have total bilirubin <3.0 mg/dL)
  - aPTT ≤1.5 × ULN
- No exercise-induced desaturation of a 6-minute walk test, defined as a blood oxygen saturation by pulse oximetry ≤88%
- No active pneumonitis
- No history of COPD
- Left ventricular ejection fraction >LLN

- Patients with metastases of the central nervous system were eligible if they met the following criteria:
  - Patients were previously treated for brain metastases and were clinically stable and were receiving ≤10 mg of prednisone/day (or equivalent) for ≥2 weeks before the first dose of study treatment
  - Patients did not have carcinomatous meningitis
- Patients were excluded from participating if any of the below criteria applied:
  - Receipt of any live vaccines within 28 days of initiation of study treatment
  - Any clinically significant cardiac, respiratory or other medical or psychiatric condition that might interfere with participation in the study or make the administration of investigational product hazardous (eg, interstitial lung disease, active infections requiring antibiotics, recent hospitalizations with unresolved symptoms, poorly controlled hypertension) or obscure the interpretation of safety and toxicity data
  - History of trauma or major surgery within 28 days before the first dose of study treatment
  - Known psychiatric or substance abuse disorders that would interfere with cooperation with the requirements of the study
  - Other prior malignancy active within the previous year except for locally curable cancers that have been apparently cured; indolent malignancies, including but not limited to early stage chronic lymphocytic leukemia and follicular lymphoma, that do not require anticancer treatment were allowed after discussion with the medical monitor
  - Prior chemotherapy targeted small-molecule therapy, immunotherapy (tumor vaccine, cytokine or growth factor given to control the cancer), biologic agents or use of other investigational drugs within 28 days before study treatment administration; locoregional therapy (immunotherapy, chemotherapy or radioembolization) must have been conducted ≥2 weeks before the first dose of study treatment
  - QTc ≥480 milliseconds
  - Known hypersensitivity to any excipient contained in the study treatment formulation
  - Inability to swallow study treatment
  - Malabsorption condition that would alter the absorption of orally administered medications
  - Any major cardiovascular event within 6 months before study treatment administration, including but not limited to myocardial infarction, unstable angina, cerebrovascular accident, transient ischemic event, pulmonary embolism, clinically significant ventricular arrhythmias or New York Heart Association Class III or IV heart failure

ALT, alanine aminotransferase; aPTT, activated partial thromboplastin time; AST, aspartate aminotransferase; BID, twice daily; COPD, chronic obstructive pulmonary disease; DLT, dose-limiting toxicity; ECOG, Eastern Cooperative Oncology Group; LLN, lower limit of normal; MTD, maximum tolerated dose; QD, once daily; QTc, heart-rate corrected QT interval; ULN, upper limit of normal.

Supplementary Table 3 | Patient eligibility criteria for the dose-expansion stage

- Capable of providing signed informed consent
- Aged ≥18 years
- Female patients must not have been pregnant, breastfeeding or planning to become pregnant
- Male patients and female patients of childbearing potential must have been willing to use highly effective contraceptive methods during the treatment period and for 90 days after the last dose of casdatifan
- Histologically confirmed metastatic ccRCC
- Received prior treatment in the locally advanced or metastatic setting with an anti–PD-(L)1 inhibitor and a VEGFR-TKI
- Creatinine clearance ≥40 mL/min
- No prior treatment with a HIF-2α–targeting therapy
- ≥1 measurable tumor lesion per RECIST v1.1
- ECOG performance status score of 0 or 1
- Patients with hepatitis B and hepatitis C were eligible if they had resolution of infection or were on adequate suppressive antiviral therapy and not at risk for hepatic decompensation
- Patients with HIV were eligible if they had CD4 T cell count ≥350 cells/μL and were receiving adequate suppressive antiviral therapy
- Adequate organ and marrow function:
  - Absolute neutrophil count ≥1.5 × 10^9/L
  - Platelet count ≥100 × 10^9/L
  - Hemoglobin ≥10.0 g/dL
  - AST ≤2.5 × ULN in the absence of hepatic metastasis or ≤5 × ULN in the presence of hepatic metastasis
  - ALT ≤2.5 × ULN in the absence of hepatic metastasis or ≤5 × ULN in the presence of hepatic metastasis
  - Bilirubin ≤1.5 × ULN (except for patients with Gilbert's syndrome who must have total bilirubin <3.0 mg/dL)
  - aPTT ≤1.5 × ULN
- No exercise-induced desaturation of a 6-minute walk test, defined as a blood oxygen saturation by pulse oximetry ≤88%
- No active pneumonitis
- No history of COPD
- Left ventricular ejection fraction >LLN
- Patients with metastases of the central nervous system were eligible if they met the following criteria:
  - Patients were previously treated for brain metastases and were clinically stable and were receiving ≤10 mg of prednisone/day (or equivalent) for ≥2 weeks before the first dose of study treatment
  - Patients did not have carcinomatous meningitis

- Patients were excluded from participating if any of the below criteria applied:
  - Receipt of any live vaccines within 28 days of initiation of study treatment
  - Any clinically significant cardiac, respiratory or other medical or psychiatric condition that might interfere with participation in the study or make the administration of investigational product hazardous (eg, interstitial lung disease, active infections requiring antibiotics, recent hospitalizations with unresolved symptoms, poorly controlled hypertension) or obscure the interpretation of safety and toxicity data
  - History of trauma or major surgery within 28 days before the first dose of study treatment
  - Known psychiatric or substance abuse disorders that would interfere with cooperation with the requirements of the study
  - Other prior malignancy active within the previous year except for locally curable cancers that have been apparently cured; indolent malignancies, including but not limited to early stage chronic lymphocytic leukemia and follicular lymphoma, that do not require anticancer treatment were allowed after discussion with the medical monitor
  - Prior chemotherapy targeted small-molecule therapy, immunotherapy (tumor vaccine, cytokine or growth factor given to control the cancer), biologic agents or use of other investigational drugs within 28 days before study treatment administration; locoregional therapy (immunotherapy, chemotherapy or radioembolization) must have been conducted ≥2 weeks before the first dose of study treatment
  - QTc ≥480 milliseconds
  - Known hypersensitivity to any excipient contained in the study treatment formulation
  - Inability to swallow study treatment
  - Malabsorption condition that would alter the absorption of orally administered medications
  - Any major cardiovascular event within 6 months before study treatment administration, including but not limited to myocardial infarction, unstable angina, cerebrovascular accident, transient ischemic event, pulmonary embolism, clinically significant ventricular arrhythmias or New York Heart Association Class III or IV heart failure

ALT, alanine aminotransferase; aPTT, activated partial thromboplastin time; AST, aspartate aminotransferase; COPD, chronic obstructive pulmonary disease; ccRCC, clear cell renal cell carcinoma; ECOG, Eastern Cooperative Oncology Group; HIF, hypoxia-inducible factor; LLN, lower limit of normal; PD-(L)1, programmed death protein 1 or programmed death ligand 1; QTc, heart-rate corrected QT interval; ULN, upper limit of normal; VEGFR-TKI, vascular endothelial growth factor receptor tyrosine kinase inhibitor.

CLINICAL STUDY PROTOCOL

ARC-20

A Phase 1, Open-Label, Dose Escalation and Dose Expansion Study, to Investigate the Safety, Tolerability, and Pharmacokinetic Profile of AB521 Monotherapy and Combination Therapies in Participants With Clear Cell Renal Cell Carcinoma and Other Solid Tumors

Short Title:
A Phase 1 Study of AB521 Monotherapy and Combination Therapies in Renal Cell Carcinoma and Other Solid Tumors

Investigational Product	Casdatifan (AB521)
Protocol Number	ARC-20
Clinical Phase	Phase 1
Amendment	10
Sponsor	Arcus Biosciences, Inc. (Arcus)<br>3928 Point Eden Way,<br>Hayward, CA 94545
Regulatory Agency Identifier Numbers	IND: 160764<br>EU CT Number: 2024-519142-70-00
Protocol Version; Date of Approval	Version 11.0; 23 December 2025
Supersedes Version; Date of Approval	Version 10.0; 30 June 2025

Confidentiality Statement

This document contains information that is PROPRIETARY AND CONFIDENTIAL to Arcus Biosciences, Inc. It is intended solely for use by the Investigator's Team, the Independent Review Board, Ethics Committees, and Health Authorities. Any other use or disclosure of the information herein is strictly prohibited without the express prior written consent of Arcus Biosciences, Inc.

STATEMENT OF COMPLIANCE

The study will be conducted in compliance with this clinical study protocol, Good Clinical Practice (GCP) as outlined by International Council for Harmonisation of Technical Requirements for Pharmaceuticals for Human Use (ICH) E6 (R3), and all applicable local and national regulatory requirements. Enrollment at any clinical study site may not begin prior to that site receiving approval from the ethics committee of record for the protocol and all materials provided to potential patients.

Any amendments to the protocol or changes to the consent document will be approved before implementation of that amendment. Reconsent of previously enrolled patients may be necessary depending on the nature of the amendment.

The principal investigator will ensure that changes to the study plan as defined by this protocol will not be made without prior agreement from the sponsor and documented approval from the ethics committee of record, unless such a change is necessary to eliminate an immediate hazard to the study patients.

All personnel involved in the conduct of this study have completed Human Research Protection and GCP Training as outlined by their governing institution.

SPONSOR'S APPROVAL

**Title**	A Phase 1, Open-Label, Dose Escalation and Dose Expansion Study, to Investigate the Safety, Tolerability, and Pharmacokinetic Profile of AB521 Monotherapy and Combination Therapies in Participants With Clear Cell Renal Cell Carcinoma and Other Solid Tumors
**Protocol Number**	ARC-20
**Protocol Version**	Version 11.0

The design of this study as outlined by this protocol has been reviewed and approved by the sponsor's responsible personnel as indicated in the signature below.

**Name:**	**Title:**
**Signature:**<br>*See appended signature page*	**Date:** 23 December 2025

INVESTIGATOR'S AGREEMENT

I have read the protocol, appendices, and accessory materials related to Study ARC-20 and agree to the following:

- To conduct this study as described by the protocol and any accessory materials.
- To protect the rights, safety, and welfare of the patients under my care.
- To provide oversight to all personnel to whom study activities have been delegated.
- To control all investigational products provided by the sponsor and maintain records of the disposition of those products.
- To conduct the study in accordance with all applicable local and national regulations, the requirements of the ethics committee of record for my clinical site, and Good Clinical Practices as outlined by ICH E6 (R3).
- To obtain approval for the protocol and all written materials provided to patients prior to initiating the study at my site.
- To obtain informed consent – and updated consent in the event of new information or amendments – from all patients enrolled at my study site prior to initiating any study-specific procedures or administering investigational products to those patients.
- To maintain participation records and all required protocol data for each patient.

**Name:**	**Title:**	**Institution:**
**Signature:**		**Date:**

STUDY CONTACT INFORMATION

Table 1: Study Contact Information

Role in Study	Email Address	Telephone Number
Medical Monitor	medicalmonitor-arc-20@arcusbio.com	PPD

DOCUMENT HISTORY

Protocol Version	Issue Date
Protocol Version 11.0	23 December 2025
Protocol Version 10.0	30 June 2025
Protocol Version 9.0	10 October 2024
Protocol Version 8.0	20 March 2024
Protocol Version 7.0	12 March 2024
Protocol Version 6.0	16 February 2024
Protocol Version 5.0	19 December 2023
Protocol Version 4.0	14 September 2023
Protocol Version 3.0	04 May 2023
Protocol Version 2.0	29 July 2022
Original Protocol Version 1.0	13 June 2022

AMENDMENT SUMMARY

This Protocol Version 11.0 (Amendment 10) replaces Protocol Version 10.0. This amendment is considered substantial based on the criteria set forth in Article 10(a) of Directive 2001/20/EC of the European Parliament and the Council of the European Union.

The primary purpose of this amendment is to add 2 additional cohorts to support the development of casdatifan in combination with zimberelimab and ipilimumab in the first-line setting for clear cell renal cell carcinoma. Editorial and formatting changes are not included in this summary.

**Section Number and Name**	**Summary of Changes**	**Rationale for Changes**
Title Page and General Synopsis and Body Text	Title Page:<br>The structure of the title page was updated. A statement of compliance, a sponsor's approval page, an investigator's agreement page, and a study contact information page were added<br>General Synopsis and Body Text: The term "participant" was replaced throughout with "patient" to more accurately reflect the enrolled population (except in reference to the original Protocol Title)<br>Minor editorial changes were made to align with standard numbering and wording	To better align the document with the current protocol template, standard language, and wording structure
Section 1. Synopsis	The structure of the synopsis was updated	To align the document with the current protocol template
	The content of the synopsis was updated per the changes made in this amendment	To align the content provided in the synopsis with the changes made to the body of the document
Section 2.1. Study Rationale	Added background information for the nivolumab and ipilimumab combination therapy in patients with advanced renal cell carcinoma (RCC)	To provide context for the use of anti-programmed cell death protein-1 (PD-1) agents (eg, nivolumab) in combination with anti-CTLA4 agents (eg, ipilimumab) in patients with RCC
	Added statement regarding the discontinuation of ipilimumab after PD for Expansion Cohort 10	To provide justification for continuing treatment while discontinuing ipilimumab

Section Number and Name	Summary of Changes	Rationale for Changes
Section 2.2.1. Comparison Between Zimberelimab and Other Anti-PD-1 Agents	Added section comparing zimberelimab to other anti-PD-1 agents	To support the use of zimberelimab, as a similar agent to nivolumab, with ipilimumab
Section 2.3.1. Risk Assessment	Added safety information regarding the use of the nivolumab and ipilimumab combination therapy with comparisons to the casdatifan, zimberelimab, and ipilimumab combination regimens for newly added Expansion Cohorts 9 and 10	To provide a risk assessment for the casdatifan, zimberelimab, and ipilimumab combination regimens for newly added Expansion Cohorts 9 and 10
Section 2.3.2. Benefit Assessment	Added efficacy information regarding the use of the nivolumab and ipilimumab combination therapy	To provide a benefit assessment for the casdatifan, zimberelimab, and ipilimumab combinations for newly added Expansion Cohorts 9 and 10
Section 2.3.3. Overall Benefit-Risk Conclusion	Added references to zimberelimab and ipilimumab	To include the addition of ipilimumab as a study drug
Section 3. Objectives and Endpoints	Added ipilimumab to the objectives and endpoints	To ensure the evaluation of the casdatifan, zimberelimab, and ipilimumab combinations
	Added an exploratory objective and endpoint	CCI [redacted]
Section 4.1. Overall Design	Added ipilimumab and Expansion Cohorts 9 and 10 to the study design description	To align the study design description with the addition of Expansion Cohorts 9 and 10
Section 4.1. Overall Design<br>Section 4.2.2. Schedule of Activities<br>Section 8.2.1. Physical Examinations<br>Section 8.2.6. Pregnancy Testing	Stated that Expansion Cohorts 7, 9, and 10 would have either a 30-day safety follow-up (SFU) after the last dose of casdatifan, or would have both a 30-day SFU visit after the last dose of casdatifan and a 100-day SFU after the last dose of immune-oncology (IO; eg zimberelimab and/or ipilimumab) therapy	The addition of the 100-day SFU was done to account for the long half-lives of zimberelimab and ipilimumab
Section 4.1.3. Dose-Expansion	Descriptions for Expansion Cohorts 9 and 10 were added	To provide information on the study design for Expansion Cohorts 9 and 10

Section Number and Name	Summary of Changes	Rationale for Changes
Section 4.1.3.4. Expansion Cohort 9<br>Section 4.1.3.5. Expansion Cohort 10	Language around the number of expected patients to be enrolled in each cohort, along with the maximum, was modified	To clarify the expected number of patients along with the maximum number of patients allowed in any one dose-expansion cohort
Section 4.1.3. Dose-Expansion, Table 5	Added footnote “a”	To clarify the transition for Expansion Cohort 7 to 720 mg every 6 weeks (Q6W) after 1 year on study treatment
Section 4.1.3.3. Expansion Cohort 7 (ex-EU only)	Added language on the transition to a different dosage for zimberelimab after 1 year on study treatment	To transfer patients to 720 mg Q6W after 1 year on study treatment
Section 4.1.5. Scientific Rationale for Study Design	Added information regarding anti-PD-1/PD-L1 therapy combinations with VEGFR-TKIs, anti-CTLA-4 agents, and HIF-2α inhibitors in patients with advanced RCC	To provide a scientific rationale for evaluating the casdatifan, zimberelimab, and ipilimumab combination therapy
Section 4.1.6. Justification for Casdatifan Starting Dose for Escalation	Deleted “preliminary” in reference to the summary of results from Study ARC-14.	To reflect that data from Study ARC-14 are final.
Section 4.1.9. Justification for Dose Levels in Expansion Cohort 7	Added Figure 4 and language on the transition to a different dosage for zimberelimab after 1 year on study treatment	To justify the transfer of patients to 720 mg Q6W after 1 year on study treatment
Section 4.1.10. Justification for Treatments and Dose Levels in Expansion Cohorts 9 and 10	Added context regarding the dosage proposed for the casdatifan, zimberelimab, and ipilimumab combination therapy	To provide a dose justification for the casdatifan, zimberelimab, and ipilimumab combination therapy
Figure 6: Schema	Updated figure	To reflect the addition of Expansion Cohorts 9 and 10 to the study and the changes to Expansion Cohort 7.
Section 4.2.2. Schedule of Activities	Moved the Schedule of Activities to Section 4.2.2.	To better align the document with the current protocol template
	Updated Table 12	To align with the transition in zimberelimab dosage after 1 year on study treatment
		To provide activities for both the 30-day and 100-day SFU visits, as applicable

Section Number and Name	Summary of Changes	Rationale for Changes
		To update the pharmacokinetic (PK) sampling per the transition in zimberelimab dosage after 1 year on study treatment
	Added Table 13	To provide the Schedule of Activities for Expansion Cohorts 9 and 10
Section 5.1. Inclusion Criteria	Updated number 4. Disease-specific criteria for dose-expansion	To include Expansion Cohorts 9 and 10 in the inclusion criteria
Section 5.2. Exclusion Criteria	Added additional exclusion criteria for Expansion Cohorts 7, 9, and 10	To exclude patients from Expansion Cohorts 7, 9, and 10 with history of particular conditions or relevant therapies
Section 6.1. Study Intervention Administered	Updated Table 16	To align with the transition in zimberelimab dosage after 1 year on study treatment
	Added Table 17	To provide information on ipilimumab administration
Section 6.2.4. Ipilimumab	Added statement for ipilimumab	To ensure adherence to regionally approved prescribing information
Section 6.5.1. Casdatifan	Removed guidance on restarting casdatifan for [redacted] from Table 24	Corrected a typographical error. Casdatifan was not withheld under these conditions and would not need to be restarted.
	Updated the guidance for casdatifan and cabozantinib treatment modification in Table 24	The modification guidance for adverse events (AEs) of anemia, hypoxia, and fatigue was removed and was revised to be consistent with Table 23. The change was made based on review of emerging safety data with inputs from Study ARC-20 investigators and key opinion leaders treated RCC patients with belzutifan with or without TKI agent. It was concluded that anemia observed in the combination cohort was primarily driven by casdatifan through HIF-2α inhibition, not an overlapping toxicity effect between casdatifan and cabozantinib.<br>The toxicity management guidelines for changes in [redacted]

Section Number and Name	Summary of Changes	Rationale for Changes
		[redacted] have been updated as [redacted] This update allows for appropriate management of [redacted] without unnecessary interruption or discontinuation of casdatifan or cabozantinib in clinically stable patients. The revised guidance supports continued treatment to enable patients to receive the full potential benefit of the combination therapy, while maintaining [redacted] monitoring and timely intervention for clinically significant [redacted].
Section 6.5.3. Zimberelimab and Ipilimumab	Added reference to European Society for Medical Oncology (ESMO), National Comprehensive Cancer Network (NCCN), American Society of Clinical Oncology (ASCO), and institutional standard practice	To ensure sites adhere to providing appropriate treatment and management in accordance with local guidelines and institutional standards
	Updated content to include ipilimumab	To provide treatment and dose modification guidance for ipilimumab
	Updated Table 26	To provide toxicity management guidance for ipilimumab in combination with zimberelimab
Section 6.6. Treatment Beyond Disease Progression	Added statement for Expansion Cohort 10	To allow Expansion Cohort 10 to be treated beyond progressive disease (PD)
Section 6.8. Treatment of Overdose	Updated the definition of casdatifan overdose from > 125% to > 150%	As casdatifan has been shown to be generally well tolerated with a manageable safety profile up to daily dose of 150 mg, the definition for a casdatifan overdose was updated from > 125% to > 150% of the protocol designated dose.
	Added definition for ipilimumab overdose	To provide guidance on the criteria for an ipilimumab overdose

Section Number and Name	Summary of Changes	Rationale for Changes
Section 6.9.1.2. Radiotherapy	Updated when study drugs can be restarted	To allow patients to restart treatment with study drugs once AEs related to radiotherapy are Grade 1 or have resolved rather than a required 2 week wait before restarting treatment
Section 6.9.2. Prohibited Therapy	Added additional guidance for use of corticosteroids for patients receiving zimberelimab and/or ipilimumab	To minimize the use of corticosteroids in patients receiving zimberelimab and/or ipilimumab
Section 8.3.4.1. Safety Follow-Up Visit Adverse Event Reporting Period	Updated Table 31	To extend the safety reporting period from 30 days to 100 days after the last dose of IO study treatment for cohorts 7, 9, and 10 to allow for adequate monitoring of late-onset immune-mediated adverse events (imAEs) since zimberelimab and ipilimumab have prolonged half-lives
Previously Section 8.3.4.6. Sponsor Contact Information	Removed Section 8.3.4.6. and moved study contact information to the Study Contact Information page	To better align the document with the current protocol template
Section 8.3.4.9. Protocol-Specific Events that Quality as Serious Adverse Events	Removed the statement requiring immune-mediated adverse events to be reported as a serious adverse event (SAE) when considered clinically significant by the investigator	The requirement that imAEs considered clinically significant by the investigator be reported as SAEs was removed to avoid over reporting of SAEs and to align with regulatory definitions of seriousness. Mild to moderate imAEs are a well recognized and expected class effect, are often transient and manageable, and do not meet the objective SAE definition criteria as defined by ICH. Mandatory SAE reporting based on subjective clinical significance can lead to inconsistent classification and unnecessary inflation of SAE volumes, with potential dilution of clinically meaningful safety signals. Under the revised approach, all imAEs continue to be fully captured and monitored as adverse events, while only those imAEs that meet

Section Number and Name	Summary of Changes	Rationale for Changes
		established seriousness criteria (eg, hospitalization, life-threatening events, or other medically important conditions) are reported as SAEs.
Section 8.3.5. Definition of Dose-Limiting Toxicities	Updated to include Expansion Cohorts 9 and 10 and their 28-day DLT period	The DLT observation period is being extended to better capture delayed immune-mediated toxicities, specifically hepatitis, which is a recognized risk of immune checkpoint inhibitors and may occur beyond the initial treatment cycle; this extension ensures that clinically meaningful immune-mediated hepatic events are appropriately assessed and incorporated into DLT determinations.
Section 8.4. Pharmacokinetics	Added statement on the 1-hour, post-dose sampling	To provide instruction on the collection of 1-hour, post-dose samples with respect to the timing of study drug infusions
	Updated the collection of pharmacokinetic samples	To account for potentially longer infusion times preventing the collection of a 1-hour post-dose sample
	Updated Table 35	To adjust the time points for PK sampling per the changes with zimberelimab dosing after 1 year on treatment and to ad hoc casdatifan PK sampling in the event of a suspected safety issue
	Added Table 36 and Table 37	To provide the PK sampling information for Expansion Cohorts 9 and 10, respectively
Section 8.5.1. Pharmacodynamics	Added Table 41 and updated text for postdose samples	To provide the pharmacodynamic sampling information for Expansion Cohorts 9 and 10
Section 8.5.2. Exploratory Biomarkers	Updated Table 42	To update the timepoints for tumor tissue sampling for the update to include Expansion Cohorts 9 and 10
	Statement added regarding the collection of fresh tumor samples	To provide clarification on tumor biopsies and to avoid undue risk to the patient

Section Number and Name	Summary of Changes	Rationale for Changes
Section 9.3.6.1. Progression-Free Survival	Added statement regarding potential exploratory analysis	To allow for exploratory analysis for patients that discontinue ipilimumab in Expansion Cohort 10
Previously Section 10.9. Appendix 9: Abbreviations	Moved the list of abbreviations from Appendix 9 to the List of Abbreviations page	To better align the document with the current protocol template
Previously Section 10.10. Appendix 10: Amendment History	Removed the history of every previous amendment in lieu of this Amendment Summary	To better align the document with the current protocol template
Section 10. References	Added additional references	To provide supporting evidence for statements made within the document

1. SYNOPSIS

**Name of Sponsor**	Arcus Biosciences, Inc.
**Protocol Number**	ARC-20
**Title**	A Phase 1, Open-Label, Dose Escalation and Dose Expansion Study, to Investigate the Safety, Tolerability, and Pharmacokinetic Profile of AB521 Monotherapy and Combination Therapies in Participants With Clear Cell Renal Cell Carcinoma and Other Solid Tumors
**Short Title**	A Phase 1 Study of AB521 Monotherapy and Combination Therapies in Renal Cell Carcinoma and Other Solid Tumors
**Regulatory Agency Identifier Numbers**	IND: 160764<br>EU CT Number: 2024-519142-70-00
**Name of Investigational Product**	Casdatifan (AB521)
**Study Drugs**	Casdatifan, cabozantinib, zimberelimab, and ipilimumab
**Study Phase**	Phase 1
**Number of Study Centers**	Approximately 30
**Countries**	Australia, South Korea, Spain, and United States
**Target Population**	Adult patients with clear cell renal cell carcinoma (ccRCC)
**Planned Number of Patients**	Approximately 362
**Length of Study**	The length of the study is anticipated to be approximately 5 years
**Study Design**	This is a multicenter, open-label, Phase 1 study evaluating the safety, pharmacokinetics (PK), preliminary clinical activity, pharmacodynamics, and biomarkers of casdatifan as monotherapy in patients with advanced solid tumor malignancies (dose escalation) and as a monotherapy and in combination with cabozantinib, zimberelimab, or zimberelimab and ipilimumab in patients with ccRCC (dose-expansion).<br>This is a non-randomized study that has 2 stages: dose-escalation and dose-expansion. The dose-escalation stage consists of casdatifan monotherapy cohorts (Escalation Cohorts 1-5). The dose-expansion stage consists of casdatifan monotherapy and combination therapy cohorts (Expansion Cohorts 1-10). The combination therapy cohorts consist of casdatifan in combination with cabozantinib (Expansion Cohort 4), zimberelimab (Expansion Cohort 7), zimberelimab and ipilimumab (Expansion Cohort 9), and zimberelimab following 4 cycles of zimberelimab and ipilimumab (Expansion Cohort 10). Expansion Cohorts 4, 7, and 9 will begin with a determination of dose and safety for the combinations.

The dose-escalation and dose-expansion stages consist of 3 study periods:

- Screening: ≤ 28 days before beginning treatment;
- Treatment Period: 21-day cycles until unacceptable toxicity, lost to follow-up, progressive disease (PD), withdrawal from the study, death, or the sponsor closes the study, whichever occurs first;
- Post-Treatment Period: 30-Day (and 100-day post-IO for Expansion Cohorts 7, 9, and 10) Post-Study Follow-Up, Post-Treatment Efficacy Follow-Up, and a Long-Term Follow-Up telephone call every 6-months (counting from treatment discontinuation visit) for survival information.

<u>Dose Escalation</u>

Casdatifan Monotherapy

Dose escalation will follow a standard 3+3 design and will be governed by the occurrence of dose-limiting toxicities (DLTs) and by recommendations from the Dose Escalation Committee (DEC) based on review of all available data (evaluation of adverse events [AEs], vital signs, electrocardiograms, tolerability, and clinical laboratory results) from the cohort under consideration. The DLT evaluation for dose escalation is defined as the first 21 days following the first dose of casdatifan. Increasing doses of casdatifan will be administered to patients with a solid tumor who do not have standard-of-care options to identify the casdatifan dose and schedule for dose-expansion. Intra-patient dose escalation will be allowed under certain conditions. Patients receiving capsules can receive the same milligram dose delivered by tablets.

The starting dose of casdatifan is 20 mg administered once daily (QD). Subsequent doses for dose escalation are indicated below.

- Escalation Cohort 1: 20 mg per day (2 × 10 mg capsules) taken QD by mouth;
- Escalation Cohort 2: 50 mg per day (5 × 10 mg capsules) taken QD by mouth;
- Escalation Cohort 3: 100 mg per day (10 × 10 mg capsules) taken twice daily (BID) (50 mg BID) by mouth;
- Escalation Cohort 4: 150 mg per day (6 × 25 mg tablets) taken QD by mouth;
- Escalation Cohort 5: 200 mg per day (8 × 25 mg tablets) taken QD by mouth.

Additional dose levels may be evaluated based on emerging safety and/or PK data but will not exceed 100% dose increase from the previous cohort for either the total daily dose or the dose at each dosing event. The highest dose of casdatifan to be tested in dose escalation will be 200 mg.

Where a patient is not evaluable against DLT criteria, that patient may be replaced. Replacement of patients must be agreed upon by the sponsor.

The DEC will not assign the next dose level until the safety data for the DLT period for all patients from the current dose level have been reviewed, and the committee has determined that safety and tolerability have been demonstrated.

<u>Dose Expansion</u>

	Dose-expansion cohorts (Expansion Cohorts 1-10) will consist of casdatifan as a monotherapy (at multiple dose levels and frequencies) and in combination with cabozantinib, zimberelimab or zimberelimab and ipilimumab. Enrollment into Expansion Cohorts 6 and 7 will not be conducted in the European Union.

	Cohort No.	Treatment Arm	Population	Dose Level/ Frequency/ Route of Administration
	Expansion Cohort 1	Casdatifan monotherapy	Second-line (2L) + clear cell renal cell carcinoma (ccRCC)	100 mg total daily dose (50 mg twice daily) orally (PO)
	Expansion Cohort 2	Casdatifan monotherapy	2L+ ccRCC	50 mg once daily (QD) PO
	Expansion Cohort 3	Casdatifan monotherapy	2L+ ccRCC	150 mg QD PO
	Expansion Cohort 4	Casdatifan + cabozantinib combination	2L and third-line (3L; post anti–programmed-cell-death protein-1/anti–programmed cell death-ligand 1 therapy)	Casdatifan 100 mg QD PO + cabozantinib 60 mg QD PO
	Expansion Cohort 5	Casdatifan monotherapy	2L+ ccRCC	100 mg QD PO
	Expansion Cohort 6 (ex-EU only)	Casdatifan monotherapy	First-line (1L) favorable risk ccRCC	100 mg QD PO
	Expansion Cohort 7 (ex-EU only)	Casdatifan + zimberelimab combination	1L ccRCC	Casdatifan 100 mg QD PO + zimberelimab 360 mg every 3 weeks intravenous (IV) infusion[a]
	Expansion Cohort 8	Casdatifan monotherapy	2L post-immune-oncology (IO) ccRCC	100 mg QD PO
	Expansion Cohort 9	Casdatifan + zimberelimab + ipilimumab followed by casdatifan + zimberelimab	1L ccRCC	Casdatifan 100 mg QD PO + zimberelimab 3 mg/kg IV Q3W + ipilimumab 1 mg/kg IV Q3W for 4 cycles followed by casdatifan 100 mg QD and zimberelimab 360 mg Q3W for 2 cycles followed by casdatifan 100 mg QD and zimberelimab 720 mg Q6W
	Expansion Cohort 10	Zimberelimab + ipilimumab followed by casdatifan + zimberelimab	1L ccRCC	Zimberelimab 3 mg/kg IV Q3W + ipilimumab 1 mg/kg IV Q3W for 4 cycles followed by casdatifan 100 mg QD and zimberelimab 360 mg Q3W

			for 2 cycles followed by casdatifan 100 mg QD and zimberelimab 720 mg Q6W

1L = first-line; 2L = second-line; 3L = third-line; BID = twice daily; ccRCC = clear cell renal cell carcinoma; IO = immune-oncology; PO = orally; Q3W = every 3 weeks; Q6W = every 6 weeks; QD = once daily; IV = intravenous

[a] Following 1 year on study treatment (starting on Cycle 18) patients will move to zimberelimab at 720 mg IV infusion every 6 weeks (Q6W).

The dose-expansion phase will consist of a non-randomized and open-label evaluation of the safety and clinical activity of 3 dose levels of casdatifan monotherapy, casdatifan in combination with cabozantinib, casdatifan in combination with zimberelimab, and casdatifan in combination with zimberelimab and ipilimumab. Each dose-expansion cohort will enroll up to approximately 30 patients. Expansion Cohorts 4, 7, and 9 will begin with a determination of dose and safety for the combinations.

If any individual dose-expansion cohort demonstrates preliminary signs of efficacy and an acceptable safety profile as determined by the safety review committee (SRC), up to an additional 30 patients may be enrolled for a maximum of 60 patients in any single dose-expansion cohort. The enrollment of up to an additional 30 patients may occur in only 1 dose-expansion cohort or across multiple dose-expansion cohorts.

Casdatifan Dose Expansion

Any casdatifan monotherapy, dose-expansion cohort may start prior to the completion of dose escalation based on emerging safety and PK/pharmacodynamic data; however, the dose selected must have cleared DLT evaluation. The highest dosage of casdatifan in the monotherapy expansion phase will not exceed the highest dosage or dosage intensity cleared for safety in the casdatifan monotherapy escalation phase. Patients receiving capsules can receive the same milligram dose delivered by tablets.

The optimal casdatifan monotherapy dose established in Expansion Cohorts 1-3 will be used to initiate subsequent dose-expansion cohorts.

- Expansion Cohort 1 patients will take 100 mg orally (PO) (50 mg BID) and may begin enrolling after the DEC review of the safety, PK, and pharmacodynamic data in the dose-escalation phase of the study. The first 6 patients enrolled in this cohort will be reviewed by the SRC after the first cycle has been completed for early safety assessment and trends. Enrollment will continue during the safety review of the first 6 patients unless stopping criteria have been reached.
- Expansion Cohort 2 patients will take the 50 mg PO (50 mg QD) dose and may begin enrolling after the DEC review of the safety, PK, and pharmacodynamic data in the dose-escalation phase of the study.
- Expansion Cohort 3 patients will take a total daily dose level of 150 mg QD and may begin enrolling after the DEC review of the safety, PK, and pharmacodynamic data in the dose-escalation phase of the study.

Expansion Cohort 4: Casdatifan + Cabozantinib Combination

Expansion Cohort 4 will evaluate casdatifan at a target dose of 100 mg QD in combination with the approved 60 mg QD monotherapy dose of cabozantinib. To ensure patient safety, the initial casdatifan dose evaluated in combination with cabozantinib will be at least 1 dose level lower than the highest casdatifan monotherapy dose determined to be safe in dose escalation, as described below. The highest dosage of casdatifan in the combination therapy dose determination phase will not exceed the highest dosage or dosage intensity cleared for safety in the casdatifan monotherapy phase.

<u>Dose Determination for Expansion Cohort 4</u>

Prior to fully enrolling Expansion Cohort 4, safety will be evaluated for the first 6 patients to complete 21 days of treatment (safety run-in phase) at a given casdatifan dose level. The safety review will be conducted per DLT criteria to assess AEs including clinically significant laboratory findings.

A lower casdatifan dose level (ie, 50 mg) in combination with 40 mg or 60 mg QD cabozantinib may be evaluated as a separate cohort of approximately 30 patients based on emerging safety and/or PK data from the 100 mg QD casdatifan and 60 mg QD cabozantinib combination therapy.

Expansion Cohort 7: Casdatifan + Zimberelimab Combination (ex-EU only)

Expansion Cohort 7 will evaluate casdatifan at a starting dose of 100 mg QD in combination with zimberelimab at 360 mg IV infusion every 3 weeks (Q3W). Following 1 year on study treatment (starting on Cycle 18), patients will move to zimberelimab at 720 mg IV infusion every 6 weeks (Q6W). The maximum treatment duration for zimberelimab is 2 years. However, patients are allowed to continue treatment with casdatifan until any of the criteria for permanent discontinuation of study treatment are met.

Prior to fully enrolling Expansion Cohort 7, safety will be evaluated for the first 6 patients to complete 21 days of treatment (safety run-in phase). The safety review will be conducted per DLT criteria to assess AEs, including clinically significant laboratory findings.

A lower casdatifan dose level (ie, 50 mg) in combination with 360 mg Q3W zimberelimab may be evaluated as a separate cohort of approximately 30 patients based on emerging safety data from the 100 mg QD casdatifan and 360 mg Q3W zimberelimab combination therapy.

Expansion Cohort 9: Casdatifan + Zimberelimab + Ipilimumab Combination followed by Casdatifan + Zimberelimab Combination

Expansion Cohort 9 will evaluate casdatifan at a starting dose of 100 mg QD PO in combination with zimberelimab at 3 mg/kg IV Q3W and ipilimumab at 1 mg/kg IV Q3W for 4 cycles followed by casdatifan 100 mg QD and zimberelimab 360 mg Q3W for 2 cycles followed by casdatifan 100 mg QD and zimberelimab 720 mg Q6W. The maximum treatment duration for zimberelimab is 2 years; however, patients are allowed to continue treatment with casdatifan until any of the criteria for permanent discontinuation of study treatment are met.

Expansion Cohort 9 will begin with a safety evaluation for the first 6 patients who complete a minimum of 28 days on treatment (safety run-in phase). The safety review will be conducted by the SRC per DLT criteria to assess AEs, including clinically significant laboratory findings. If < 2 DLTs in the first 6 patients are observed, the cohort will be expanded to up to approximately 30 patients. The

sponsor may consider evaluating the triplet combination with casdatifan at lower doses based on recommendations from the SRC.

DLT-evaluable patients are defined as those who complete the 28-day DLT period and have received at least 80% of the intended dose of casdatifan or those who experience a DLT.

Any dose that clears the safety run-in phase may be expanded further to enroll up to an additional 30 patients at that dose level. Additionally, up to 30 patients may be enrolled at a lower dose of casdatifan than the highest dose that clears the safety run-in phase.

Expansion Cohort 10: Zimberelimab + Ipilimumab Combination followed by Casdatifan + Zimberelimab Combination

Expansion Cohort 10 will evaluate zimberelimab at 3 mg/kg IV Q3W and ipilimumab at 1 mg/kg IV Q3W for 4 cycles followed by casdatifan 100 mg QD and zimberelimab 360 mg Q3W for 2 cycles followed by casdatifan 100 mg QD and zimberelimab 720 mg Q6W. The maximum treatment duration for zimberelimab is 2 years. However, patients are allowed to continue treatment with casdatifan beyond the 2 years until any of the criteria for permanent discontinuation of study treatment are met.

Patients in Expansion Cohort 10 that have [redacted] on the combination with zimberelimab and ipilimumab may remain on treatment but will discontinue ipilimumab and transition to casdatifan 100 mg QD and zimberelimab 360 mg Q3W for 2 cycles followed by casdatifan 100 mg QD and zimberelimab 720 mg Q6W. Casdatifan at 100 mg QD can begin immediately following [redacted] while the next dose of zimberelimab should be consistent with Q3W dosing from the previous dose. All patients continuing study treatment with casdatifan in combination with zimberelimab after [redacted] on the combination therapy with zimberelimab and ipilimumab must continue tumor assessments at the same interval, until [redacted] and/or loss of clinical benefit as judged by the investigator.

Number of Patients:

Approximately 62 patients are anticipated in the dose-escalation part. This includes a minimum of 15 and up to a maximum of 42 patients planned for the 3+3 dose level cohorts (5 proposed cohorts). Additional dose levels may be evaluated if DLT criteria are not met at proposed cohort dose levels.

An additional 20 patients may be enrolled at any dose level during dose escalation at or below the highest dose cleared in order to collect additional PK, pharmacodynamic, and safety data.

Each dose-expansion cohort will enroll up to approximately 30 patients, for approximately 300 patients planned across the 10 dose-expansion cohorts. Each doss-expansion cohort may enroll up to 30 additional patients for a maximum of 60 patients in any single dose-expansion cohort.

Enrollment of additional patients beyond the original 30 patients in the dose expansion cohorts will be based on the determination of an acceptable safety profile and preliminary signs of efficacy. Dose-expansion cohorts will not commence dosing until the dose-escalation cohort at the dose entering expansion has cleared the DLT period.

<table>
<tr><td></td><td>The number of patients that enroll into the study may change as the study progresses based on emerging data.
Dose Escalation Committee (DEC) & Safety Review Committee (SRC):
The DEC (dose-escalation part) and SRC (dose-expansion part) will consist of the sponsor medical monitor [or designee] (chair), principal investigators [or designee], lead clinical scientist [or designee], safety representative [or designee], the lead statistician [or designee], lead clinical pharmacologist [or designee] and may include other subject matter experts (internal/external), as necessary.
For additional information, refer to the DEC/SRC charter.</td></tr>
<tr><td>Rationale</td><td>The purpose of this study is to characterize the safety and tolerability, PK, pharmacodynamic, and preliminary clinical activity of casdatifan in patients with solid tumor malignancies and ccRCC to inform on the dose and schedule of casdatifan for further clinical development as a monotherapy or in combination with cabozantinib, zimberelimab, or zimberelimab in combination with ipilimumab.</td></tr>
<tr><td>Data Monitoring Committee/Other Committee</td><td>Dose Escalation Committee and Safety Review Committee</td></tr>
<tr><td>Study Objectives and Endpoints</td><td>
<table>
<tr><th>Primary Objectives</th><th>Primary Endpoints</th></tr>
<tr><td>• To characterize the safety and tolerability of casdatifan monotherapy in patients with advanced solid tumor malignancies and clear cell renal cell carcinoma (ccRCC) during dose escalation
• To characterize the safety and tolerability of casdatifan monotherapy in patients with ccRCC
• To characterize the safety and tolerability of casdatifan in combination with cabozantinib, zimberelimab, or zimberelimab in combination with ipilimumab in patients with ccRCC</td><td>• The incidence of dose-limiting toxicities and adverse events (AEs) in patients with solid tumors during dose escalation
• The incidence of AEs in patients with ccRCC during dose expansion monotherapy with casdatifan
• The incidence of AEs in patients with ccRCC during combination with casdatifan and cabozantinib, zimberelimab, or zimberelimab in combination with ipilimumab</td></tr>
<tr><th>Secondary Objectives</th><th>Secondary Endpoints</th></tr>
<tr><td>• To assess the clinical activity of casdatifan monotherapy
• To assess the clinical activity of casdatifan in combination with cabozantinib, zimberelimab, or zimberelimab in combination with ipilimumab</td><td>• Objective response rate as assessed by the investigator according to Response Evaluation Criteria in Solid Tumors (RECIST) v.1.1</td></tr>
</table>
</td></tr>
</table>

	• To characterize the pharmacokinetics (PK) of casdatifan as a monotherapy and in combination with cabozantinib, zimberelimab, or zimberelimab in combination with ipilimumab	• Plasma concentration and PK parameters for casdatifan after oral dosing of casdatifan as monotherapy and in combination with cabozantinib, zimberelimab, or zimberelimab in combination with ipilimumab
	**Exploratory Objectives**	**Exploratory Endpoints**
	• To assess clinical activity from expansion phase following doses of casdatifan monotherapy in patients with ccRCC<br>• To assess clinical activity from expansion phase following doses of casdatifan in combination with cabozantinib, zimberelimab, or zimberelimab in combination with ipilimumab in patients with ccRCC	• Progression-free survival, defined as the time from date of first dose until progressive disease (PD) or death from any cause, whichever comes first as measured according to RECIST v1.1 as assessed by the investigator<br>• Overall survival, defined as the time from date of first dose until death due to any cause
	• CCI [redacted]	• CCI [redacted]
	• To investigate the pharmacodynamics of casdatifan as a monotherapy or in combination with cabozantinib, zimberelimab, or zimberelimab in combination with ipilimumab in patients with ccRCC or advanced solid tumor malignancies	• Changes in erythropoietin and other candidate pharmacodynamic biomarkers in the blood and tumor tissues
	• To investigate the relationship between the tumor characteristics and clinical activity following casdatifan treatment in patients with solid tumors and ccRCC<br>• To investigate the relationship between the tumor characteristics and clinical activity following casdatifan treatment in combination with cabozantinib, zimberelimab, or zimberelimab in combination with ipilimumab in patients with ccRCC	• Assess the predictive value of biomarkers in tumor tissue and blood and their association with clinical activity

	• CCI [redacted]<br>• CCI [redacted]<br>• CCI [redacted]<br>• CCI [redacted]<br>Enrollment into Expansion Cohorts 6 and 7 will not be conducted in the European Union.
**Ethical Considerations of Benefit-Risk**	Benefits of treatment with hypoxia inducible factor (HIF)-2α inhibitor therapy have been shown for patients with von Hippel-Lindau (VHL) disease and ccRCC and may potentially benefit patients in the ARC-20 study due to class action effect. This is the first clinical evaluation of casdatifan as monotherapy or in combination with cabozantinib, zimberelimab, or zimberelimab in combination with ipilimumab for potential benefit in patients with solid tumor malignancies and ccRCC. Hence, a comprehensive understanding of the benefits and risks in humans is unknown. Preliminary clinical data showed a manageable safety profile for casdatifan monotherapy and in combination with cabozantinib and zimberelimab, and early signs of efficacy of casdatifan monotherapy in advanced and metastatic ccRCC patients in late lines of treatment and casdatifan with cabozantinib in a post-anti-programmed cell death protein-1 (PD-1)/programmed cell death-ligand 1 (PD-L1) setting. Since an accurate estimation of the benefits and risks are unknown, patients will be closely monitored throughout the study, for both safety and potential clinical benefit. The mechanism of action of HIF-2α agent (casdatifan), and a vascular endothelial growth factor receptor-targeting tyrosine kinase inhibitor (VEGFR-TKI) (cabozantinib), anti-PD-1 (zimberelimab), or anti-PD-1 (zimberelimab)/anti-cytotoxic T-lymphocyte-associated protein 4 (CTLA4) (ipilimumab) combination, may provide added antitumor effect beyond monotherapy and combination therapies alone for patients with advanced ccRCC with limited treatment options, supporting a potential favorable benefit-risk profile.

TABLE OF CONTENTS

TITLE PAGE 1
STATEMENT OF COMPLIANCE 2
SPONSOR'S APPROVAL 3
INVESTIGATOR'S AGREEMENT 4
STUDY CONTACT INFORMATION 5
DOCUMENT HISTORY 6
AMENDMENT SUMMARY 7
1. SYNOPSIS 15
TABLE OF CONTENTS 25
LIST OF ABBREVIATIONS 33
2. INTRODUCTION 37
2.1. Study Rationale 38
2.2. Background 41
2.2.1. Comparison Between Zimberelimab and Other Anti-PD-1 Agents 41
2.2.2. Clinical Pharmacokinetics 42
2.3. Benefit/Risk Assessment 46
2.3.1. Risk Assessment 46
2.3.2. Benefit Assessment 50
2.3.3. Overall Benefit-Risk Conclusion 51
3. OBJECTIVES AND ENDPOINTS 52
4. STUDY DESIGN 54
4.1. Overall Design 54
4.1.1. Dose Escalation 54
4.1.1.1. Casdatifan Monotherapy 54
4.1.2. Intra-Patient Dose Escalation 56
4.1.3. Dose Expansion 56
4.1.3.1. Casdatifan Monotherapy 58
4.1.3.2. Expansion Cohort 4 58
4.1.3.3. Expansion Cohort 7 (ex-EU only) 59
4.1.3.4. Expansion Cohort 9 59
4.1.3.5. Expansion Cohort 10 60

4.1.4. Stopping Criteria for Dose Expansion 60
4.1.5. Scientific Rationale for Study Design 61
4.1.6. Justification for Casdatifan Starting Dose for Escalation 62
4.1.7. Justification for Dose Levels in Expansion Cohorts (Casdatifan Monotherapy) 63
4.1.8. Justification for Dose Levels in Expansion Cohort 4 64
4.1.9. Justification for Dose Levels in Expansion Cohort 7 64
4.1.10. Justification for Treatments and Dose Levels in Expansion Cohorts 9 and 10 65
4.1.11. Justification for Inclusion of Patients With Renal Impairment 66
4.1.12. End of Study Definition 69
4.2. Study Scheme and Schedule of Activities 70
4.2.1. Study Design 70
4.2.2. Schedule of Activities 71
5. STUDY POPULATION 89
5.1. Inclusion Criteria 89
5.2. Exclusion Criteria 92
5.3. Lifestyle Considerations 95
5.3.1. Meals and Dietary Restrictions 95
5.4. Screen Failures 95
6. STUDY INTERVENTION AND CONCOMITANT THERAPY 97
6.1. Study Intervention Administered 97
6.2. Preparation, Handling, Storage, and Accountability 99
6.2.1. Casdatifan 99
6.2.2. Cabozantinib 99
6.2.3. Zimberelimab 100
6.2.4. Ipilimumab 100
6.3. Measures to Minimize Bias: Randomization and Blinding 100
6.4. Study Intervention Compliance 100
6.5. Dose Modifications 100
6.5.1. Casdatifan 100
6.5.2. Cabozantinib 104
6.5.3. Zimberelimab or Zimberelimab in Combination With Ipilimumab 110
6.6. Treatment Beyond Disease Progression 115

6.7. Continued Access to Study Intervention After Study Termination 115
6.8. Treatment of Overdose 115
6.9. Concomitant Therapy 116
6.9.1. Permitted Therapy 117
6.9.1.1. Surgical Procedures While on Cabozantinib 117
6.9.1.2. Radiotherapy 117
6.9.2. Prohibited Therapy 117
7. DISCONTINUATION OF STUDY INTERVENTION AND PATIENT DISCONTINUATION/WITHDRAWAL 119
7.1. Discontinuation of Study Treatment 119
7.2. Patient Withdrawal from the Study 120
7.3. Lost to Follow-Up 120
7.4. Study Termination 121
8. STUDY ASSESSMENTS AND PROCEDURES 122
8.1. Efficacy and Survival Assessments 122
8.2. Safety Assessments 123
8.2.1. Physical Examinations 123
8.2.2. Vital Signs 124
8.2.3. Electrocardiograms and Echocardiogram / Multigated Acquisition Scan (MUGA) 124
8.2.4. Clinical Safety Laboratory Tests 125
8.2.5. COVID-19 Precautions 127
8.2.6. Pregnancy Testing 127
8.2.7. Eastern Cooperative Oncology Group Performance Status 127
8.3. Adverse Events, Serious Adverse Events, and Other Safety Reporting 128
8.3.1. Adverse and Serious Adverse Events 128
8.3.1.1. Definitions 128
8.3.1.2. Classifications 129
8.3.2. Assessment of Causality 129
8.3.3. Assessment of Severity 130
8.3.4. Reporting Adverse Events 131
8.3.4.1. Adverse Event Reporting Period 131
8.3.4.2. Method of Eliciting Adverse Event Information 132

8.3.4.3. Procedure for Recording Adverse Events 132
8.3.4.4. Events Requiring Expedited Reporting to the Sponsor 133
8.3.4.5. Serious Adverse Event Reporting Requirements 134
8.3.4.6. Regulatory Reporting Requirements for Serious Adverse Events 134
8.3.4.7. Collection of Pregnancy Information 135
8.3.4.8. Adverse Events of Special Interest 135
8.3.4.9. Protocol-Specific Events That Qualify as Serious Adverse Events 135
8.3.5. Definition of Dose-Limiting Toxicities 136
8.4. Pharmacokinetics 136
8.5. Biological Samples for Pharmacodynamics/Biomarker Assessments 143
8.5.1. Pharmacodynamics 143
8.5.2. Exploratory Biomarkers 145
8.5.3. Sample Collection and Testing 146
9. STATISTICAL CONSIDERATIONS AND ANALYSIS PLAN 148
9.1. Sample Size Considerations 148
9.2. Analysis Populations 149
9.3. Statistical Analyses 150
9.3.1. Study Patients 150
9.3.1.1. Disposition 150
9.3.2. Baseline Characteristics 150
9.3.2.1. Demographics 150
9.3.2.2. Disease History 150
9.3.2.3. Prior Anticancer Therapies 150
9.3.3. General Methodology 151
9.3.3.1. Summary of Treatment Cohorts 151
9.3.3.2. Reporting Conventions 151
9.3.4. Primary Endpoint Analyses 151
9.3.4.1. Adverse Events 151
9.3.5. Secondary Endpoint Analyses 152
9.3.5.1. Objective Response Rate 152
9.3.5.2. Pharmacokinetic Analyses 152
9.3.6. Exploratory Analyses 152

9.3.6.1. Progression-Free Survival 152
9.3.6.2. Overall Survival 152
9.3.7. Other Analyses 153
9.3.7.1. Pharmacodynamic/Biomarker Analyses 153
9.3.7.2. Population Pharmacokinetic Analyses or Pharmacokinetic/Pharmacodynamic Modeling 153
9.4. Interim Analysis 153
10. SUPPORTING DOCUMENTATION AND OPERATIONAL CONSIDERATIONS 154
10.1. Appendix 1: Regulatory, Ethical, and Study Oversight Considerations 154
10.1.1. Regulatory and Ethical Considerations 154
10.1.2. Financial Disclosure 154
10.1.3. Informed Consent Process 155
10.1.4. Data Protection 156
10.1.5. Committee Structure 156
10.1.5.1. Dose Escalation Committee 156
10.1.6. Dissemination of Clinical Study Data 156
10.1.7. Reporting Requirements for Serious Breaches 157
10.1.8. Data Management and Quality Assurance 157
10.1.8.1. Record Retention 158
10.1.9. Source Documents 158
10.1.10. Study and Site Start and Closure 159
10.1.11. Publication Policy 159
10.2. Appendix 2: Contraceptive and Barrier Guidance 161
10.2.1. Definitions 161
10.2.2. Contraception Guidance for Male and Female Patients 162
10.3. Appendix 3: Genetics 165
10.4. Appendix 4: Eastern Cooperative Oncology Group (ECOG) Performance Status 166
10.5. Appendix 5: RECIST 1.1 167
10.6. Appendix 6: Examples of Inhibitors for P450-Mediated Metabolism 170
10.7. Appendix 7: Concomitant Medications Associated With Risk of QTc Interval Prolongation and/or Torsades de Pointes 171

10.8. Appendix 8: International Metastatic Renal Cell Carcinoma Database Consortium (IMDC) Criteria 173
11. REFERENCES 174

LIST OF TABLES

Table 1: Study Contact Information 5
Table 2: Preliminary Plasma Pharmacokinetic Parameters of Casdatifan Following Single or Multiple Oral Doses of Casdatifan in Healthy Participants (Study ARC-14) and in Patients With Cancer (Study ARC-20) 44
Table 3: Summary of Statistical Comparisons of Plasma Casdatifan PK Parameters Between 100 mg Tablets-Fasted (Treatment B) vs 100 mg Capsules-Fasted (Treatment A) From Study ARC-28 45
Table 4: Summary of Statistical Comparisons of Plasma Casdatifan PK Parameters Between 100 mg Tablets-Fed (Treatment C) vs 100 mg Tablets-Fasted (Treatment B) From Study ARC-28 45
Table 5: Dose Expansion – Casdatifan Monotherapy and Combination Therapy Cohorts 57
Table 6: Rationale for Inclusion of Tumor Types 62
Table 7: Population Pharmacokinetic Parameters of Casdatifan Model 67
Table 8: Comparison of Predicted Exposures for Casdatifan in Patients With Normal ($CL_{CR} \geq 90$ mL/min) and Moderate Impairment Renal Function ($CL_{CR} \geq 40$ mL/min) 68
Table 9: Dose Escalation Assessments and Procedures (Casdatifan Monotherapy) 71
Table 10: Dose Expansion Assessments and Procedures (Casdatifan Monotherapy) 74
Table 11: Expansion Cohort 4 Assessments and Procedures 77
Table 12: Expansion Cohort 7 (ex-EU only) Assessments and Procedures 81
Table 13: Expansion Cohort 9 and Cohort 10 Assessments and Procedures 85
Table 14: Study Intervention – Casdatifan 97
Table 15: Study Intervention – Cabozantinib 98
Table 16: Study Intervention – Zimberelimab 98
Table 17: Study Intervention – Ipilimumab 99
Table 18: Examples for Recommended Dose Reductions for Casdatifan for Capsule Formulation (Monotherapy) 100
Table 19: Examples for Recommended Dose Reductions for Casdatifan for Tablet Formulation (Monotherapy) 101

Table 20: Examples for Recommended Dose Reductions for Combination Therapy Attributed to Casdatifan Tablet 101
Table 21: Examples for Recommended Dose Reductions for Combination Therapy Attributed to Cabozantinib 101
Table 22: Examples for Recommended Dose Reductions for Combination Therapy Attributed to Both Casdatifan Tablet and Cabozantinib....101
Table 23: Casdatifan Treatment Modification Guidance for Adverse Reactions....102
Table 24: Casdatifan and Cabozantinib Treatment Modification Guidance for Adverse Reactions....103
Table 25: Cabozantinib Treatment Modification Guidance for Adverse Reactions....104
Table 26: Toxicity Management Guidelines for Zimberelimab or Zimberelimab in Combination With Ipilimumab....110
Table 27: Protocol-Required Safety Laboratory Tests 126
Table 28: Eastern Cooperative Oncology Group Performance Status....128
Table 29: Causal Attribution Guidance 130
Table 30: Adverse Event Severity Grading Scale for Events Not Specifically Listed in NCI CTCAE 131
Table 31: Time Period for Collecting AE, SAE, and Other Reportable Safety Event Information 132
Table 32: Blood Sampling Schedule for Pharmacokinetic Analysis – Phase 1 Dose Escalation for Casdatifan Monotherapy (Escalation Cohorts 1-5) 138
Table 33: Blood Sampling Schedule for Pharmacokinetic Analysis – Phase 1 Dose Expansion for Casdatifan Monotherapy (Expansion Cohorts 1, 2, 3, 5, 6, and 8) 138
Table 34: Blood Sampling Schedule for Pharmacokinetic Analysis – Phase 1 Dose Expansion in Expansion Cohort 4 139
Table 35: Blood Sampling Schedule for Pharmacokinetic/Anti-Drug Antibody Analysis – Phase 1 Dose Expansion in Expansion Cohort 7....140
Table 36: Blood Sampling Schedule for Pharmacokinetic/Anti-Drug Antibody Analysis – Phase 1 Dose Expansion in Expansion Cohort 9....141
Table 37: Blood Sampling Schedule for Pharmacokinetic/Anti-Drug Antibody Analysis – Phase 1 Dose Expansion in Expansion Cohort 10....142
Table 38: Blood Sampling Schedule for EPO, [redacted] and Serum Pharmacodynamic Analysis – Phase 1 Dose Escalation (Escalation Cohorts 1-5)....143
Table 39: Blood Sampling Schedule for EPO, [redacted] and Serum Pharmacodynamic Analysis – Phase 1 Dose Expansion for Casdatifan Monotherapy (Expansion Cohorts 1, 2, 3, 5, 6, and 8)....144

Table 40: Blood Sampling Schedule for EPO, [redacted] and Serum Pharmacodynamic Analysis – Phase 1 Dose Expansion for Combination Therapies (Expansion Cohort 4 and 7) 144
Table 41: Blood Sampling Schedule for EPO, [redacted] and Serum Pharmacodynamic Analysis – Phase 1 Dose Expansion for Combination Therapies (Expansion Cohort 9 and 10) 145
Table 42: Proposed Biomarkers 145
Table 43: Associated Probabilities of Observing ≥ 1 Adverse Event With Various Underlying Probabilities in a Sample of 30 or 60 Patients 148
Table 44: Representative Confidence Intervals for Various Observed Response Rates in Dose Expansion Cohorts 1-10 149
Table 45: Analysis Populations 150
Table 46: Contraceptives Allowed During the Study[a] 163
Table 47: Response Based on Evaluation of Target Lesions at Each Assessment 168
Table 48: Response Based on Evaluation of Nontarget Lesions at Each Assessment 168
Table 49: Evaluation of Overall Response at Each Assessment 169
Table 50: Concomitant Medications Associated With Risk of QTc Interval Prolongation and/or Torsades de Pointes 171

LIST OF FIGURES

Figure 1: Mean (+SD) Plasma Concentration of Casdatifan vs Time Profiles Following a Single Oral Dose of Casdatifan at 3, 10, 30, or 100 mg in Healthy Participants (Study ARC-14) 43
Figure 2: Mean (+SD) Plasma Concentration of Casdatifan vs Time Profiles Following Single or Multiple Oral Doses of Casdatifan at 15, 30, or 50 mg Once Daily in Healthy Volunteers (Study ARC-14) and at 20 mg Once Daily, 50 mg Once Daily, 50 mg Twice Daily, 100 mg Once Daily, and 150 mg Once Daily in Patients With Cancer (Study ARC-20) 43
Figure 3: Expansion Cohort 4 Dose Determination Flowchart 59
Figure 4: Population PK Simulation of Zimberelimab in Patients With Cancer 65
Figure 5: Population PK Simulations of Casdatifan at Steady State at 50 mg QD, 50 mg BID, and 100 mg BID in Normal (CL_{CR} ≥ 90 mL/min) and Moderate Impairment (CL_{CR} ≥ 40 mL/min) Renal Function 68
Figure 6: Schema 70

LIST OF ABBREVIATIONS

Abbreviation	Definition
1L, 2L	first-line, second-line
ADA	anti-drug antibody
AE	adverse event
ALT	alanine aminotransferase
ARNT	aryl hydrocarbon receptor nuclear translocator
ASCO	American Society of Clinical Oncology
AST	aspartate transaminase
AUC	area under the concentration-time curve
$AUC_{0\text{-}inf}$	area under plasma concentration-time curve from time zero extrapolated to infinity
$AUC_{0\text{-}t}$	area under plasma concentration-time curve from time zero to the last measurable concentration
BID	twice daily
C	Cycle
ccRCC	clear cell renal cell carcinoma
CFR	Code of Federal Regulations
CI	confidence interval
CL_{CR}	creatinine clearance
C_{max}	maximum plasma concentration
CNS	central nervous system
cORR	confirmed objective response rate
COVID-19	coronavirus disease 2019
CR	complete response
CRF	case report form
CRO	contract research organization
CSR	Clinical Study Report
CT	computed tomography
CTIS	Clinical Trials Information System
CTLA-4	cytotoxic T-lymphocyte-associated protein 4
CYP	cytochrome P450
D	Day

Abbreviation	Definition
DDI	drug-drug interaction
DEC	Dose Escalation Committee
DLT	dose-limiting toxicity
DNA	deoxyribonucleic acid
ECG	electrocardiogram
ECOG	Eastern Cooperative Oncology Group
eCRF	electronic case report form
EDC	electronic data capture (clinical database)
EPO	erythropoietin
ESMO	European Society for Medical Oncology
ETV	Early Termination Visit
EU	European Union
FDA	Food and Drug Administration
CCI [redacted]	CCI [redacted]
FSH	follicle-stimulating hormone
GCP	Good Clinical Practice
GDPR	General Data Protection Regulation
GI	gastrointestinal
GLP	Good Laboratory Practice
Hct	hematocrit
Hgb	hemoglobin
HIF	hypoxia inducible factor
HNSTD	highest non-severely toxic dose
HRT	hormonal replacement therapy
IB	Investigator’s Brochure
ICF	Informed Consent Form
ICH	International Conference for Harmonisation
IEC	independent ethics committee
imAE	immune-mediated adverse event
IO	immune-oncology
IRB	institutional review board
LLN	lower limit of normal

Abbreviation	Definition
LMWH	low-molecular-weight heparin
LVEF	left ventricular ejection fraction
MRI	magnetic resonance imaging
MTD	maximum tolerated dose
MUGA	multigated acquisition scan
NCCN	National Comprehensive Cancer Network
NCI CTCAE	National Cancer Institute Common Terminology Criteria for Adverse Events
NOAEL	no-observed-adverse-effect level
ORR	objective response rate
PAS	Per-ARNT-SIM
PD	progressive disease
PD-1	programmed cell death protein-1
PD-L1	programmed cell death-ligand 1
PFS	progression-free survival
p-gp	p-glycoprotein
PK	pharmacokinetic(s)
PO	orally
PPE	palmar-plantar erythrodysesthesia
PR	partial response
pVHL	von Hippel-Lindau tumor suppressor protein
Q3W	every 3 weeks
Q6W	every 6 weeks
QD	once daily
QTc	QT corrected
QTcF	QT corrected using Fridericia's formula
RBC	red blood cell
RCC	renal cell carcinoma
RECIST	Response Evaluation Criteria in Solid Tumors
RNA	ribonucleic acid
SAE	serious adverse event
SFU	Safety Follow-Up

Abbreviation	Definition
SoA	Schedule of Activities
SRC	safety review committee
STD10	severely toxic dose in 10% of animals
SUSAR	suspected unexpected serious adverse reaction
TEAE	treatment-emergent adverse event
TKI	tyrosine kinase inhibitor
ULN	upper limit of normal
uORR	unconfirmed objective response rate
US	United States
VEGF	vascular endothelial growth factor
VEGFR	vascular endothelial growth factor receptor
VEGFR-TKI	vascular endothelial growth factor receptor-targeting tyrosine kinase inhibitor
VHL	von Hippel-Lindau
WOCBP	woman of childbearing potential
WONCBP	woman of nonchildbearing potential

2. INTRODUCTION

Renal cell carcinoma (RCC) is one of the more common neoplasms diagnosed in developed nations (Padala et al. 2020) and accounts for 90% of all kidney cancers, with clear cell renal cell carcinoma (ccRCC) comprising 83% to 88% of the various RCC histologic subtypes (Hsieh et al. 2017). Approximately 74,000 new cases of kidney-based neoplasms were registered in the United States (US) in 2019, accounting for 4.2% of all US-diagnosed cancers (Padala et al. 2020) and close to 14,000 deaths (Siegel et al. 2021).

The treatment landscape for advanced RCC has revolutionized over the past 2 decades. Targeted therapies like vascular endothelial growth factor receptor-targeting tyrosine kinase inhibitors (VEGFR-tyrosine kinase inhibitors [TKIs]; eg, cabozantinib, lenvatinib, axitinib, sunitinib) or mammalian target of rapamycin (mTOR) inhibitor everolimus were the primary systemic treatment options with improved clinical outcomes for patients (Choueiri and Motzer 2017). In recent years, immune checkpoint inhibitors, such as anti–programmed cell death protein-1 (anti-PD-1; pembrolizumab, nivolumab) and anti–programmed cell death-ligand 1 (anti-PD-L1; avelumab), or anti-cytotoxic T-lymphocyte-associated protein 4 (anti-CTLA-4; ipilimumab) have become primary backbone therapy for frontline treatment for advanced RCC based on single-agent antitumor activity and survival benefit (Tenold et al. 2020).

The combination of anti-PD-1/PD-L1 with VEGFR-TKIs is the current frontline standard of care for the majority of patients with advanced ccRCC, based on median progression-free survival (PFS) ranging from 13.3 – 23.9 months across multiple studies (Motzer et al. 2019; Rini et al. 2019; Choueiri et al. 2021a; Motzer et al. 2021). However, subsequent treatment options are limited to VEGFR-TKIs and everolimus following the inability of atezolizumab combined with cabozantinib to improve clinical outcomes in patients with anti-PD-1/PD-L1 experienced post-immune-oncology (post-IO) RCC (Choueiri et al. 2023a). Nevertheless, single-agent cabozantinib demonstrated improved response and PFS in this post-IO study in comparison to previous studies that led to first-line (1L) and second-line (2L) approvals, and remains the standard of care among treatment options for 2L patients with RCC (Choueiri et al. 2015; Choueiri et al. 2018).

A high proportion of ccRCCs have inactivation of the von Hippel-Lindau tumor suppressor protein (pVHL), which results in accumulation of hypoxia inducible factor (HIF) alpha subunits (Miikkulainen et al. 2019). HIF-2α expression is notably elevated in ccRCC cases with pVHL inactivation, promoting a concomitant aggressive growth pattern; in addition, elevated prolyl hydroxylase domain 3 expression in ccRCC cells leads to elevated HIF-2α expression, activating downstream target genes and promoting aggressive cell growth patterns (Miikkulainen et al. 2019). These findings have translated to the clinic, where the first-in-class HIF-2α inhibitor belzutifan demonstrated a clinically meaningful response rate (49%) in patients with von Hippel-Lindau (VHL) disease-associated RCC in a single-arm Phase 2 study (Jonasch et al. 2021). The results of this study led to the approval of belzutifan as treatment for adults with VHL disease who require therapy for associated ccRCC tumors.

Given the high frequency of dysregulation in the VHL pathway in ccRCC tumors, targeting HIF-2α offers a novel approach to the treatment of patients with advanced ccRCC. Patients with ccRCC in a single-arm Phase 1b study in 2L or later were treated with belzutifan monotherapy; the results showed a disease control rate of 80% in a heavily pre-treated population, with durable responses in 25% of patients and a median PFS of 14.5 months, similar to that observed with established VEGFR-TKIs (Choueiri et al. 2021b). Collectively, these results validate the development of therapies targeting HIF-2α as a novel approach to treat ccRCC.

Applying a pharmacophore mapping and structure-based design approach, we identified a novel small molecule HIF-2α inhibitor, casdatifan, for evaluation in patients with ccRCC.

2.1. Study Rationale

The purpose of this study is to characterize the safety and tolerability, pharmacokinetics (PK), pharmacodynamics, and preliminary clinical activity of casdatifan in patients with solid tumor malignancies and ccRCC to inform on the dose and schedule of casdatifan for further clinical development as a monotherapy or in combination with cabozantinib, zimberelimab, or zimberelimab and ipilimumab. After showing early signs of efficacy as a single agent in late line monotherapy treatment with a manageable toxicity profile that is different from current commonly used treatment options such as a VEGF-TKI or immunotherapy, additional treatment settings and combinations are proposed to be added per amendment. The rationale for each of these is described in the following sections.

Combination With a TKI

In vitro biochemical and/or cellular assays have shown that cabozantinib inhibits the tyrosine kinase activity of MET, VEGFR-1, -2 and -3, AXL, RET, ROS1, TYRO3, MER, KIT, TRKB, FLT-3, and TIE-2. These receptor tyrosine kinases are involved in both normal cellular function and pathologic processes such as oncogenesis, metastasis, tumor angiogenesis, drug resistance, and maintenance of the tumor microenvironment (CABOMETYX [cabozantinib] Prescribing Information).

The treatment options for patients with advanced RCC following progressive disease (PD) on anti-PD-1 or anti-PD-L1 immunotherapy (post-IO) are limited to single-agent VEGFR-TKIs, such as cabozantinib. Combining a HIF-2α inhibitor with a TKI that targets VEGFR, c-MET, and AXL may result in greater antitumor effects beyond their individual benefits alone. Therefore, a combination cohort that includes casdatifan and the TKI, cabozantinib, has the potential to provide additional clinical activity beyond that of cabozantinib alone. In the METEOR study, cabozantinib treatment alone in patients with advanced RCC who had relapsed from prior therapy resulted in an objective response rate (ORR) of 21% and a median PFS of 7.4 months (Choueiri et al. 2015).

Promising preliminary data for patients treated with a HIF-2α inhibitor and cabozantinib has been shown in the LITESPARK-003 trial (Choueiri et al. 2023a). In this study, the combination of belzutifan and cabozantinib was evaluated in 2L to third-line patients with ccRCC with primarily intermediate or poor International Metastatic RCC Database Consortium (IMDC) risk who were previously treated with immunotherapy and/or VEGFR-TKI. The belzutifan and cabozantinib combination resulted in an ORR of 31% with a median follow-up of 24.6 months. In addition, the 12-month median PFS rate was 56%, with a 12-month overall survival (OS) rate

of 77%. The emerging safety was manageable and consistent with the individual safety profiles for cabozantinib and belzutifan, suggesting a positive benefit to combined HIF-2α and cabozantinib therapy in this patient population.

IMDC Favorable-Risk

The treatment options for 1L-metastatic IMDC favorable-risk patients include surveillance, antiangiogenic therapy, single-agent immunotherapy, and combination therapy (immunotherapy combinations or immunotherapy and VEGF-TKI). IMDC favorable-risk patients have prolonged survival and slow disease progression (Zarba et al. 2024). While anti-PD-1 combinations with VEGF-TKIs are approved as treatment in 1L ccRCC across all IMDC risk groups and have demonstrated ORR and PFS improvement compared with sunitinib monotherapy, a pooled analysis of recent Phase 3 studies for the IO-TKI combinations demonstrated no advantage in OS for IMDC favorable-risk patients (Lee et al. 2023). Due to toxicities from immunotherapy and IO-TKI therapies, alternative options for favorable risk (IMDC = 0) could be pursued, specifically for patients contraindicated for established therapies or those who decline therapies due to related toxicities.

VEGF-TKI monotherapy has shown efficacy in patients with limited disease burden and favorable prognostic scores (IMDC = 0). A retrospective analysis of a pivotal Phase 3 trial comparing sunitinib versus IFN-α demonstrated an ORR of 53% among IMDC favorable-risk patients (Rini et al. 2018). Based on these data, sunitinib is recommended as a 1L, category 2A, and other recommended regimen for patients with ccRCC across all IMDC risk groups in the National Comprehensive Cancer Network guidelines (NCCN).

Activity of single-agent HIF-2α inhibitors has been demonstrated in prior studies with patients with VHL disease and ccRCC (Jonasch et al. 2021; Choueiri et al. 2024a; Agarwal et al. 2024). The HIF-2a inhibitor, belzutifan, demonstrated a clear benefit in ORR versus everolimus (21.9% vs 3.5%) and a statistically significant benefit in PFS in patients who had received prior immunotherapy and a VEGF-TKI in the Phase 3 LITESPARK-005 study (Choueiri et al. 2024a). Despite the benefit in response and median PFS, no improvement in median OS was observed for belzutifan compared to everolimus in the final analysis (Rini et al. 2024). However, in a recent subgroup analysis of the LITESPARK-005 study, belzutifan demonstrated superiority to everolimus in patients with IMDC favorable risk across ORR (22.8% vs 6.0%), median PFS (10.9 months vs 6.8 months, HR 0.74), and median OS (30.7 months vs 25.4 months, HR 0.74) (Albiges et al. 2024). A similar ORR was observed among belzutifan-treated patients with IMDC favorable risk compared to those with IMDC intermediate/poor risk (22.8% vs 22.7%); however, results showed double the median PFS (10.9 months vs 5.3 months) and approximately double the median OS (30.4 months vs 18.2 months), suggesting the potential for PFS and OS benefits similar to those with a VEGF-TKI monotherapy. Therefore, exploring casdatifan monotherapy in favorable-risk patients who have a likelihood of a response to a single-agent VEGF-TKI provides the potential to expand treatment options for patients who may not tolerate a TKI or immunotherapy agent.

Combination With Anti-PD-1

Immunotherapies that target PD-1/PD-L1 are the standard of care for advanced RCC, either as monotherapy in the adjuvant setting (Choueiri et al. 2024b) or in combination with anti-CTLA-4 or VEGF-TKIs for frontline metastatic patients (Motzer et al. 2018; Motzer et al. 2019; Rini et al. 2019; Choueiri et al. 2021a; Motzer et al. 2021). The Phase 3 KEYNOTE-

581/CLEAR study demonstrated promising efficacy of a PD-1 inhibitor in combination with a VEGF-TKI in the 1L setting for the treatment of advanced RCC. The combination of lenvatinib plus pembrolizumab demonstrated a median PFS of 23.9 months versus 9.2 months for sunitinib (Motzer et al. 2024). However, HIF-2α inhibitors also demonstrate antitumor activity; it is possible the combination of a HIF-2α with a checkpoint inhibitor may provide benefit in the 1L treatment of RCC with a more favorable safety profile than a TKI-based regimen.

Combination With Anti-PD-1 and Anti-CTLA-4

Treatment with nivolumab in combination with ipilimumab in the first-line setting is an established standard of care for patients with advanced RCC. In the Phase 3 CheckMate-214 clinical trial, nivolumab in combination with ipilimumab demonstrated improved survival benefits and a duration of response almost 3 times as long as sunitinib in patients with previously untreated advanced RCC (Tannir et al. 2024). Recent studies, such as COSMIC-313, have attempted to build on this first-line combination with the addition of cabozantinib; however, the improvements observed in PFS of this triplet regimen ultimately did not translate into a benefit in OS. Further, 79% of patients treated with the triplet developed Grade 3 or 4 adverse events (AEs) compared to 56% receiving the standard of care (Choueiri et al. 2023b). Given that HIF-2α inhibitors have demonstrated antitumor activity through a different mechanism of action than IO therapy and a lack of overlapping toxicity with IO therapy, it is possible that the addition of casdatifan to anti-PD-1/anti-CTLA-4 therapy may provide additional clinical benefit compared to anti-PD-1/anti-CTLA-4 therapy alone (see Section 2.2.1 for comparison between zimberelimab and nivolumab).

For patients enrolled in Expansion Cohort 10, primary PD on the combination with zimberelimab and ipilimumab may be the result of immunosuppression in the tumor due to elevated intratumor erythropoietin (EPO) consistent with a high HIF-2α driven tumor (Chiu et al. 2025). Therefore, if a patient on zimberelimab in combination with ipilimumab has PD, the patient may continue treatment on casdatifan in combination with zimberelimab until further PD or loss of clinical benefit as judged by the investigator.

Post-IO TKI-Naïve

First-line treatment for patients with advanced RCC has evolved towards dual checkpoint or checkpoint-VEGF-TKI combinations, with 2L options mainly representing VEGF-TKIs. The Phase 2 CaboPoint trial demonstrated a median PFS of 8.3 to 10.9 months for patients treated with cabozantinib in the 2L setting (Albiges et al. 2024). However, patients who cannot receive a VEGF-TKI require an effective therapy in the 2L setting. The Phase 2 LITESPARK-013 study evaluated belzutifan monotherapy at either 200 mg or 120 mg in previously treated patients with metastatic ccRCC (Agarwal et al. 2024). While the ORR (23.1% and 23.7%, respectively) for both arms was similar to that observed in LITESPARK-005, a pooled subgroup analysis demonstrated that patients who received prior immunotherapy-only (eg, TKI-naive) versus patients treated with immunotherapy and a VEGF-TKI had a substantially improved ORR (34.1% vs 22.0%) (Atkins et al. 2024). Evaluation of casdatifan in this setting may provide additional options for patients who refuse treatment with a VEGF-TKI or for whom VEGF-TKIs are contraindicated.

2.2. Background

Cells in the solid tumor microenvironment are frequently exposed to hypoxic (low oxygen) conditions, necessitating molecular adaptations for survival. The HIFs are a family of transcription factors that modulate the cellular response to hypoxia. HIF is a heterodimer consisting of an oxygen-regulated alpha subunit (of which there are 3 isoforms: HIF-1α, HIF-2α, and HIF-3α) that can heterodimerize with a constitutively expressed beta subunit (HIF-1β/aryl hydrocarbon receptor nuclear translocator [ARNT]) using Per-ARNT-SIM (PAS) protein-protein interaction domains.

Under normal tissue oxygen saturation, HIF-2α protein is continuously degraded. Prolyl hydroxylase domain-containing enzymes use oxygen as a substrate to hydroxylate proline residues located within the oxygen-dependent degradation domain of HIF-2α. Prolyl hydroxylation enables recognition of the HIF-2α protein by the VHL E3-ubiquitin ligase complex, resulting in ubiquitination and subsequent proteasomal degradation of HIF-2α. In hypoxic tissue oxygen status, or in the case of VHL mutation or epigenetic silencing, HIF-2α is stabilized and undergoes nuclear translocation. Upon dimerization with HIF-1β, HIF-2α mediates transcription of pro-tumorigenic genes associated with stemness, survival, metabolism, proliferation, and angiogenesis. Thus, targeting HIF-2α may be an effective strategy to thwart tumor progression. To that end, small molecules that bind the HIF-2α PAS-B domain and drive allosteric changes that disrupt dimerization with HIF-1β and prevent HIF-2α-mediated gene transcription have been identified (Yu et al. 2019).

VHL disease is a hereditary condition associated with constitutive HIF-2α activity and tumors arising in multiple organs. Additionally, an appreciable proportion of patients with sporadic ccRCC have somatic mutations or epigenetic modifications that inactivate VHL (Sato et al. 2013; Choueiri et al. 2021a). In these patients, HIF-2α, and not HIF-1α, has been shown to be the driver promoting tumorigenesis and disease progression.

Importantly, HIF-2α has been shown to be a tumorigenic driver in the archetypical VHL mutated 786-O ccRCC cell line (Kondo et al. 2002; Kondo et al. 2003). Ongoing clinical trials utilizing an allosteric small molecule HIF-2α inhibitor, belzutifan, have demonstrated clinical benefit in VHL disease (NCT03401788) and patients with ccRCC (NCT04195750). Based on the overall response rate in NCT03401788, the Food and Drug Administration (FDA) recently approved belzutifan for adult patients with VHL disease who require therapy for associated RCC, central nervous system (CNS) hemangioblastomas, or pancreatic neuroendocrine tumors.

Casdatifan is an inhibitor of HIF-2α with a high binding affinity for HIF-2α and potently inhibits transcription of HIF-2α-dependent genes in cell lines and when given orally to mice. Casdatifan is provided as a semi-solid solution formulation containing the inactive ingredients polyethylene glycol 1500 and polyethylene glycol 400. The study drug has been given to 18 healthy volunteers in doses of 3 mg, 10 mg, and 30 mg in an oral, single-ascending dose study (ARC-14).

2.2.1. Comparison Between Zimberelimab and Other Anti-PD-1 Agents

Zimberelimab, pembrolizumab, and nivolumab are all monoclonal antibodies using an IgG4 framework designed to bind the PD-1 receptor. In terms of efficacy and safety, zimberelimab, pembrolizumab, and nivolumab are [redacted] anti-PD-1 monoclonal antibodies. Although no randomized trials have been conducted comparing all 3 or any 2 of these, the cross-

trial and retrospective or real-world comparisons of pembrolizumab and nivolumab consistently conclude no differences in efficacy or safety (Cybulska-Stopa et al. 2023).

Zimberelimab has been shown to be a clinically equivalent anti-PD-1 antibody to pembrolizumab or nivolumab. The non-randomized within study comparison between the zimberelimab (n = 40) and pembrolizumab (n = 33) is available from the Phase 2 Study ARC-10 for patients with NSCLC. The confirmed objective response rate (95% CI) was 35% (20.6, 51.7) for the zimberelimab cohort and 33.3% (18.0, 51.8) for the pembrolizumab cohort. Safety data from 680 patients who received zimberelimab as monotherapy, or in combination with other oncology agents across Arcus and Gilead sponsored Phase 1 and 2 studies, demonstrate a safety profile consistent with the class effects of PD-1 inhibitors (see the Investigator's Brochure [IB] for zimberelimab).

2.2.2. Clinical Pharmacokinetics

As of 30 August 2024, preliminary casdatifan PK data are available for 168 patients/participants (including 56 healthy participants in Study ARC-14 and 112 patients in the Study ARC-20) who received either a single oral dose of casdatifan at 3, 10, 30, or 100 mg, or multiple oral doses of casdatifan at 15, 20, 30, 50, 100, 150 mg once daily (QD), or 50 mg twice daily (BID).

Following single (3 to 150 mg) or multiple (15 to 150 mg QD) oral dosing of casdatifan, median time to reach peak concentration at Day 1 was 1 to 3.5 hours and at steady state was 1.0 to 1.5 hours, followed by multiphasic decline in plasma concentrations (Figure 1). Mean apparent terminal half-life was approximately 18 to 24 hours, supporting QD dosing (Table 2). Exposure (area under the concentration-time curve [AUC]) to casdatifan increased in an approximately dose-proportional manner between 3 and 150 mg. After multiple oral dosing, casdatifan reaches steady state around Day 4, and the mean AUC accumulation ratio ranges from 1.6 to 2.0 (Figure 2 and Table 2). Casdatifan renal clearance ranges from 14.2 to 25.8 mL/min at dose levels of 15-100 mg and is similar across all dose levels. The effect of once-daily 50 mg dose of casdatifan to steady-state on the PK of an index cytochrome P450 (CYP)3A4 substrate midazolam was also evaluated in 12 healthy volunteers in ARC-14. When compared to the midazolam dosing in the absence of casdatifan, the AUC of oral midazolam co-administered with 50 mg casdatifan was reduced by 38%, indicating that casdatifan is a weak CYP3A4 inducer at this dose level.

Based on PK data from ARC-28 (a relative bioavailability and food effect study of casdatifan in healthy volunteers), the total exposure of casdatifan (AUC from time zero to the last measurable concentration [$AUC_{0\text{-}t}$] and AUC from time zero extrapolated to infinity [$AUC_{0\text{-}inf}$]) administered as tablets (4 × 25 mg) is generally equivalent to capsules (10 × 10 mg) with 90% confidence interval (CI) of exposure ratio lying within 80%-125% (Table 3). The upper bound of the 90% CIs for maximum plasma concentration (C_{max}) slightly exceeded the upper limit of this reference range, but this is unlikely to be clinically significant. Overall, casdatifan PK, when administered as tablets or capsules, can be considered to be generally equivalent. In addition, the total exposure of casdatifan ($AUC_{0\text{-}t}$ and $AUC_{0\text{-}inf}$) in tablets with or without food is equivalent, while food resulted in a reduced C_{max} and delayed T_{max} (Table 4), indicating that coadministration of casdatifan with food is unlikely to have clinically significant effect on its safety/efficacy profile. Preliminary PK data (Table 2) indicates casdatifan PK exposures at steady state (C_{avg}) are comparable between 50 mg BID capsule and 100 mg QD tablet.

Figure 1: Mean (+SD) Plasma Concentration of Casdatifan vs Time Profiles Following a Single Oral Dose of Casdatifan at 3, 10, 30, or 100 mg in Healthy Participants (Study ARC-14)

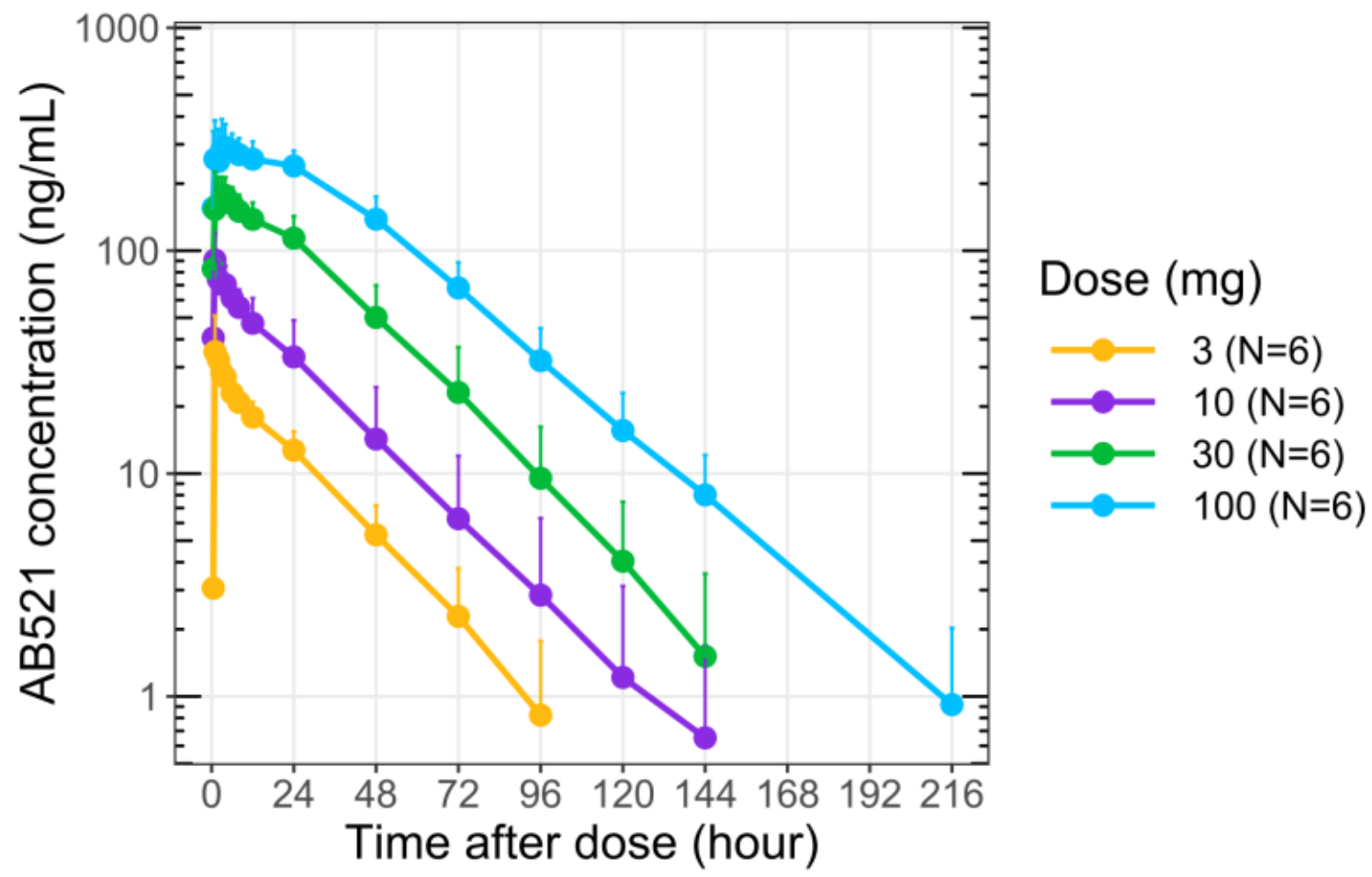

AB521 = casdatifan; SD = standard deviation; vs = versus

Figure 2: Mean (+SD) Plasma Concentration of Casdatifan vs Time Profiles Following Single or Multiple Oral Doses of Casdatifan at 15, 30, or 50 mg Once Daily in Healthy Volunteers (Study ARC-14) and at 20 mg Once Daily, 50 mg Once Daily, 50 mg Twice Daily, 100 mg Once Daily, and 150 mg Once Daily in Patients With Cancer (Study ARC-20)

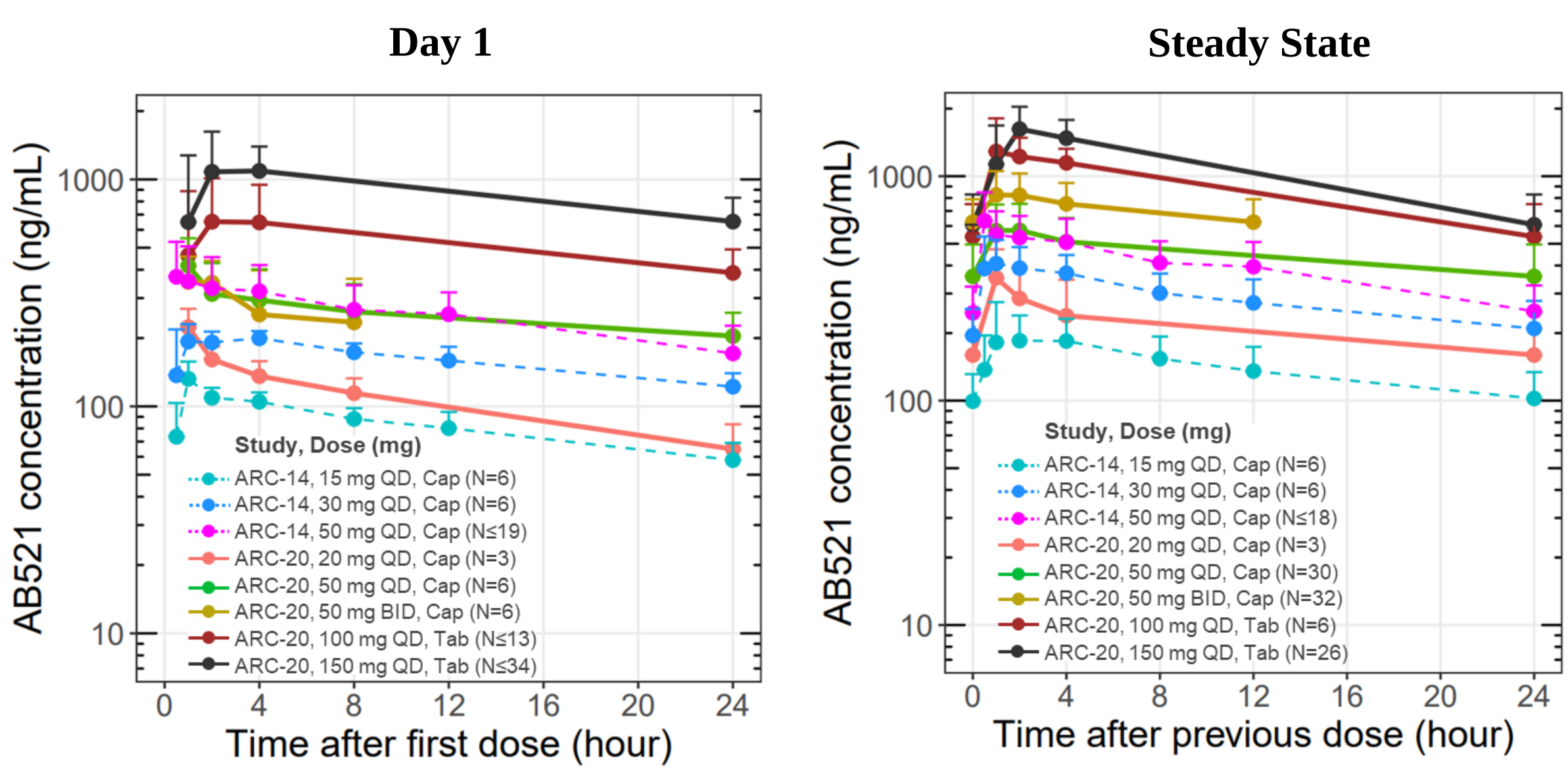

AB521 = casdatifan; BID = twice daily; Cap = capsule; DDI = drug-drug interaction; MAD = multiple ascending dose; PK = pharmacokinetic; QD = once daily; SD = standard deviation; Tab = tablet; vs = versus

Note: Steady state PK profiles were based on samples collected after the seventh dose for MAD cohorts at 15, 30, 50 mg QD and after the sixth dose for DDI cohort at 50 mg QD in Study ARC-14 and after Cycle 1 Day 15 (C1D15) in Study ARC-20. In Study ARC-20 at C1D15 profiles, timepoints at the end of dosing interval (24h in QD and 12h in BID) are based on pre-dose timepoints, as steady-state was established. Number of observations at each timepoint (ie, N) may be different.

Table 2: Preliminary Plasma Pharmacokinetic Parameters of Casdatifan Following Single or Multiple Oral Doses of Casdatifan in Healthy Participants (Study ARC-14) and in Patients With Cancer (Study ARC-20)

Study ID	Dose Regimen	Day 1						Steady State			
		N	C_{max} (ng/mL)	T_{max} (h)	AUC_{tau} (h•ng/mL)	AUC_{∞} (h•ng/mL)	$t_{1/2}$ (h)	N	C_{max} (ng/mL)	T_{max} (h)	AUC_{tau} (h•ng/mL)
ARC-14	3 mg (SAD, Cap)	6	39.5 (24%)	1.0 (1.0-2.0)	-	822 (25%)	20.1 (20%)	-	-	-	-
ARC-14	10 mg (SAD, Cap)	6	95.0 (30%)	1.0 (0.5-6.0)	-	2177 (45%)	17.9 (42%)	-	-	-	-
ARC-14	15 mg QD (Cap)	6	132 (19%)	1.0 (1.0-1.0)	1945 (13%)	-	-	6	203 (32%)	1.5 (1.0-4.0)	3360 (29%)
ARC-14	30 mg QD (Cap)	6	224 (17%)	2.5 (0.5-12)	3681 (9.1%)	-	-	6	443 (25%)	1.0 (0.5-4.0)	6844 (26%)
ARC-14	30 mg (SAD, Cap)	6	190 (26%)	3.0 (1.0-6.0)	-	6651 (28%)	18.2 (20%)	-	-	-	-
ARC-14	50 mg QD (Cap)	7	432 (21%)	1.0 (0.5-2.0)	6497 (26%)	-	-	6	666 (25%)	0.5 (0.5-4.0)	10067 (26%)
ARC-14	50 mg QD (DDI Cohort, Cap)	12	355 (44%)	1.0 (1.0-4.4)	5959 (26%)	-	-	12	559 (25%)	1.0 (1.0-4.0)	8416 (23%)
ARC-14	100 mg (SAD, Cap)	6	338 (28%)	3.5 (0.5-6.0)	-	15177 (23%)	23.9 (15%)	-	-	-	-
ARC-20	20 mg QD (Cap)	3	223 (21%)	1.0 (1.0-1.0)	2490 (13%)	-	-	3	353 (34%)	1.0 (1.0-1.0)	5020 (47%)
ARC-20	50 mg QD (Cap)	6	420 (31%)	1.0 (1.0-2.0)	5990 (30%)	-	-	30	611 (27%)	2.0 (1.0-4.0)	10400 (29%)
ARC-20	50 mg BID (Cap)	6	374 (24%)	1.5 (1.0-8.0)	ND	-	-	32	865 (25%)	2.0 (1.0-4.0)	8680 (24%)
ARC-20	100 mg QD (Tab)	2	694 (817, 570)	3.0 (2.0-4.0)	11400 (12600, 10100)	-	-	6	1380 (31%)	1.5 (1.0-4.0)	20500 (24%)
ARC-20	150 mg QD (Tab)	17	1230 (32%)	2.0 (1.0-24)	20200 (24%)	-	-	26	1710 (21%)	2.0 (1.0-4.0)	24800 (23%)

%CV = percentage coefficient of variation; AUC_{∞} = area under plasma concentration-time curve from time zero extrapolated to infinity; AUC_{tau} = AUC from one dosing interval (0-24 h for QD dosing and 0-12 h for BID dosing); BID = twice daily; Cap = capsule; CxDx = Cycle x Day x; C_{max} = maximum plasma concentration; C_{trough} = trough concentration; DDI = drug-drug interaction; h = hours; ID = identification; MAD = multiple ascending dose; ND = not determined; PK = pharmacokinetics; QD = once daily; SAD = single ascending dose; Tab = tablet; T_{max} = time to maximum plasma concentration; $t_{1/2}$ = apparent terminal elimination half-life

Note: Mean (%CV) for C_{max}, AUC_{tau}, AUC_{∞}, and $t_{1/2}$; Median (range) for T_{max}. The PK parameters were calculated using non-compartmental analysis based on nominal time of sample collection. Steady state in ARC-14 refers to 7th dose for MAD cohorts at 15, 30, 50 mg QD and 6th dose for DDI cohort at 50 mg QD. Steady State in ARC-20 refers to C1D15. For the calculation of AUC_{tau} at steady state (C1D15) in ARC-20, the C_{trough} (C1D16 pre-dose) sample was not collected per protocol and C_{trough} was assumed to be equal to the corresponding pre-dose concentration, assuming steady state was reached prior to C1D15. PK parameters following BID dosing were characterized based on PK profiles for the dose administration in the morning up to 8 hours postdose. AUC_{tau} for BID dosing on C1D1 is not calculated due to the absence of PK sampling at 12 hours postdose unless terminal phase is well characterized for the extrapolations of AUC_{tau}.

Table 3: Summary of Statistical Comparisons of Plasma Casdatifan PK Parameters Between 100 mg Tablets-Fasted (Treatment B) vs 100 mg Capsules-Fasted (Treatment A) From Study ARC-28

Comparison	PK Parameter	Test Geometric LSMs	Test n	Reference Geometric LSMs	Reference n	GMR (%)	90% Confidence Interval	Intra-volunteer CV% Test	Intra-volunteer CV% Reference
Treatment B (Test) vs Treatment A (Reference)	AUC_{0-t} (ng•h/mL)	24100	24	21270	24	113.33	107.16 - 119.85	8.71	13.53
	AUC_{0-inf} (ng•h/mL)	24260	24	21460	24	113.01	106.81 - 119.58	8.77	13.66
	C_{max} (ng/mL)	1023	24	868.8	24	117.77	109.71 - 126.43	11.18	17.13

CV% = percentage coefficient of variation; AUC_{0-inf} = area under plasma concentration-time curve from time zero extrapolated to infinity; AUC_{0-t} = area under plasma concentration-time curve from time zero to the last measurable concentration; C_{max} = maximum plasma concentration; PK = pharmacokinetics
Note: Treatment B: 100 mg casdatifan (4 × 25 mg tablets) on Day 1 under fasted conditions
Treatment A: 100 mg casdatifan (10 × 10 mg capsules) on Day 1 under fasted conditions
Geometric least-squares means (LSMs) are calculated by exponentiating the LSMs derived from the analysis of variance (ANOVA).
Geometric Mean Ratio (GMR) = 100 × (test/reference)
Intra-volunteer CV% was calculated as 100 × square root(exp[MSE]-1), where MSE = Treatment specific residual variance from ANOVA.

Table 4: Summary of Statistical Comparisons of Plasma Casdatifan PK Parameters Between 100 mg Tablets-Fed (Treatment C) vs 100 mg Tablets-Fasted (Treatment B) From Study ARC-28

Comparison	PK Parameter	Test Geometric LSMs	Test n	Reference Geometric LSMs	Reference n	GMR (%)	90% Confidence Interval	Intra-volunteer CV% Test	Intra-volunteer CV% Reference
Treatment C (Test) vs Treatment B (Reference)	AUC_{0-t} (ng•h/mL)	23620	24	24100	24	97.98	94.75 - 101.31	3.87	8.71
	AUC_{0-inf} (ng•h/mL)	23780	24	24260	24	98.04	94.84 - 101.35	3.57	8.77
	C_{max} (ng/mL)	787.0	24	1023	24	76.91	71.78 - 82.41	16.39	11.18

CV% = percentage coefficient of variation; AUC_{0-inf} = area under plasma concentration-time curve from time zero extrapolated to infinity; AUC_{0-t} = area under plasma concentration-time curve from time zero to the last measurable concentration; C_{max} = maximum plasma concentration; PK = pharmacokinetics
Note: Treatment C: 100 mg casdatifan (4 × 25 mg tablets) on Day 1, 30 minutes after the start of a high-fat meal
Treatment B: 100 mg casdatifan (4 × 25 mg tablets) on Day 1 under fasted conditions
Geometric least-squares means (LSMs) are calculated by exponentiating the LSMs derived from the analysis of variance (ANOVA).
Geometric Mean Ratio (GMR) = 100 × (test/reference)
Intra-volunteer CV% was calculated as 100 × square root(exp[MSE]-1), where MSE = Treatment specific residual variance from ANOVA.

2.3. Benefit/Risk Assessment

2.3.1. Risk Assessment

In casdatifan nonclinical safety assessments, monitorable drug-related effects included minimal to moderate anemia (characterized by reduced red cell mass parameters; hemoglobin [Hgb], hematocrit [Hct], and red blood cell [RBC] counts) following repeated doses in rats and dogs, and prolonged PR, QT, and QT corrected (QTc) intervals of ≤ 15 msec in dog without effects on electrocardiogram (ECG) morphology. Effects on male reproductive organs with no evidence of reversibility were evident in rat. These changes are similar to those reported for the approved HIF-2α inhibitor belzutifan (WELIREG [belzutifan] Prescribing Information 2025).

In the 28-day Good Laboratory Practice (GLP) toxicity study in rat, evidence of male reproductive toxicity without recovery was observed at doses ≥ 10 mg/kg/day. These changes were characterized by microscopic germ cell degeneration/atrophy of minimal severity in the testis and presence of cellular debris and reduced sperm of minimal to moderate severity in the epididymis, which correlated with decreased epididymis weights at ≥ 30 mg/kg/day and decreased testis weights at 100 mg/kg/day only at the recovery phase. Therefore, due to the potential for male reproductive toxicity, male study patients will be informed of this risk before participation both in the Informed Consent Form (ICF) and by the investigator.

In the 28-day GLP toxicity studies in rat and dog where the severely toxic dose in 10% of animals (STD10)/no-observed-adverse-effect levels (NOAELs) were determined to be 100 mg/kg/day in rat and 20 mg/kg/day in dog, casdatifan was well-tolerated, without clinical signs in both species. Minimal to moderate anemia (decreased Hgb, Hct, and RBC count) was observed following repeat dosing, but showed reversibility following cessation of dosing, and was considered non-adverse. These changes are consistent with target pharmacology of HIF-2α inhibition. Based on the potential for anemia, close monitoring of hematology parameters has been implemented to mitigate for the risk of severe anemia.

In the cardiovascular safety pharmacology study in conscious, telemetered dogs, casdatifan-related prolonged PR (≤ 5 msec), QT (≤ 19 msec) and QTc intervals (≤ 15 msec) at 20 mg/kg, and minor hemodynamic changes were observed at ≥ 2 mg/kg; these changes trended towards resolution by the end of telemetry collection. Therefore, appropriate safety measures (eg, exclusion criteria [Section 5.2] and close monitoring of QTc [12-lead safety ECG (Section 8.2.3)]) will be performed in this study. No ECG nor hemodynamic changes were observed in the 28-day GLP study.

No effects on respiratory or CNS parameters occurred in rats in the GLP safety pharmacology study at doses up to 100 mg/kg, which was also the NOAEL for female rats in the 28-day GLP toxicity study.

In vitro metabolism of casdatifan was evaluated in rat, dog, monkey, and human liver microsomes and hepatocytes. Metabolite profiles were qualitatively similar across species with no unique or disproportionate human metabolites. Studies with recombinant CYP enzymes and CYP-selective chemical inhibitors demonstrated that oxidative metabolism of casdatifan in human liver microsomes was catalyzed mainly by CYP3A. Therefore, recent or concurrent administration of any drugs or food that are strong or moderate inhibitors or inducers of CYP3A

will be prohibited during this trial. In vitro, casdatifan is a substrate for human multidrug resistance protein 1 (MDR1) efflux transporter; however, since casdatifan had high permeability in vitro (Caco-2 cell monolayer), the effect of efflux transport on casdatifan PK is expected to be limited. Concurrent administration of any drugs that are inhibitors of p-glycoprotein (p-gp) is prohibited during this trial. Casdatifan is an inducer of CYP2B6, CYP2C8, CYP2C9, CYP2C19, and CYP3A4 in human hepatocytes in vitro, and potentially could cause a clinically significant induction of CYP2B6, CYP2C8, CYP2C9, CYP2C19, and CYP3A4. Clinical drug-drug interaction (DDI) assessment of the coadministration of casdatifan and midazolam indicated that multiple oral doses of casdatifan at 50 mg QD (for 7 days) resulted in a 22% and 38% reduction in midazolam exposure in terms of C_{max} and AUC, respectively; therefore, casdatifan (at 50 mg QD) is considered a weak inducer of CYP3A4. Concurrent administration of any drugs that are substrates of CYP3A4, CYP2B6, CYP2C8, CYP2C9, or CYP2C19 with a narrow therapeutic window is prohibited, except for hormonal contraceptives without a gestodene component that are allowed. Cabozantinib is a substrate of CYP3A4 in vitro. Based on preliminary physiologically-based PK modeling, coadministration of casdatifan (100 mg QD or 50 mg BID) and cabozantinib (60 mg daily) is predicted to result in up to ~25% reduction in cabozantinib AUC at steady state, which is not considered clinically relevant based on the exposure-efficacy relationship of cabozantinib in patients with RCC (Castellano et al. 2020). The plasma concentrations of cabozantinib will be monitored in the combination arm of this study. Coadministration of casdatifan and cabozantinib is not expected to have impact on the exposure of casdatifan.

Casdatifan was initially evaluated in Study ARC-14, a first-in-human study that established safety, PK, and pharmacodynamics of casdatifan in healthy volunteers. Casdatifan was well tolerated at single and multiple oral doses, and no safety concerns were identified up to 100 mg single dose and up to 50 mg QD for 7 days.

Study ARC-20 is an ongoing, Phase 1, open-label, dose-escalation and dose-expansion study evaluating casdatifan as monotherapy in patients with advanced solid tumor malignancies (dose escalation) and as monotherapy and in combination with cabozantinib or zimberelimab in patients with ccRCC (dose expansion). Safety and tolerability of casdatifan during the dose-escalation part was designed to be monitored by the Dose Escalation Committee (DEC) (Section 10.1.5). As of the data cutoff (30 August 2024), the dose-escalation and dose-expansion phases have enrolled 22 and 119 patients, respectively. In the dose-escalation phase (doses: 20 mg QD, 50 mg QD, 50 mg BID, 150 mg QD, and 200 mg QD), 17 (77.3%) patients reported casdatifan-related treatment-emergent adverse events (TEAEs), with 1 (4.5%) patient experiencing Grade 3 treatment-related TEAEs of dyspnea and hypoxia, 1 patient (4.5%) experiencing a Grade 3 treatment-related TEAE of hypoxia, and 1 patient (4.5%) experiencing a Grade 3 treatment-related TEAE of anemia. In the dose-expansion phase (doses: 50 mg QD, 50 mg BID, 100 mg QD, and 150 mg QD), 105 (88.2%) patients experienced treatment-related TEAEs (37 [31.1%] patients with treatment-related TEAEs Grade ≥ 3). These casdatifan-related TEAEs Grade ≥ 3 were: Grade 3 anemia in 32 patients (26.9%), Grade 3 hypoxia in 7 patients (5.9%), Grade 3 fatigue in 2 patients (1.7%), and 1 (0.8%) patient with a Grade 4 hypoxia. These Grade ≥ 3 casdatifan-related TEAEs were managed with dose interruptions (35 patients; 17 with

anemia and 4 with hypoxia) and supportive measures. Ten (8.4%) patients underwent a dose reduction, and 2 (1.7%) patients discontinued treatment due to a related TEAE.

Casdatifan is not known to exert any effect on the immune system involved in response to viral infections; therefore, it is not expected to increase the risk of infection. Details of procedures and risk mitigation as they relate to this risk are included in the relevant sections of the protocol and in a dedicated section on coronavirus disease 2019 (COVID-19) precautions (Section 8.2.5).

Risk Assessment for Casdatifan in Combination With Cabozantinib

The safety profile for the 60 mg QD dose of cabozantinib monotherapy is well established across multiple clinical studies, including data from 3 Phase 3 studies in patients with advanced RCC (Choueiri et al. 2015; Tannir et al. 2022; Pal et al. 2023). The most common (> 20% of patients) all-grade TEAEs observed across all 3 studies were diarrhea (71%-74%), nausea (36%-54%), fatigue (24%-56%), palmar-plantar erythrodysesthesia (PPE) (39%-42%), hypertension (33%-37%), and hypothyroidism (20%-38%). The most common Grade 3 or higher TEAEs were hypertension (13%-18%), diarrhea (7%-13%), fatigue (4%-9%), and PPE (4%-8%).

While the preliminary safety for casdatifan (Section 2.3.2) appears distinct to the safety profile of a VEGF-TKI such as cabozantinib, there is the potential for overlapping toxicities to occur with the combination. The occurrence of anemia (17%-19%) among all-grade TEAEs for cabozantinib monotherapy in 2 of the Phase 3 studies is noteworthy, although the frequency of Grade 3 or higher events was low (1%-5%). Still, an ongoing Phase 2 study (Choueiri et al. 2023a) of 60 mg QD cabozantinib with 120 mg QD belzutifan in patients with both 1L and 2L ccRCC has demonstrated a safety profile for the combination that was well-tolerated and consistent with the safety profile for the individual agents (Choueiri et al. 2023a).

CCI [redacted]

In Study ARC-20, Arcus is exploring casdatifan as monotherapy in dose escalation (all solid tumors) and dose expansion in metastatic ccRCC patients as well as casdatifan 100 mg in combination with cabozantinib 60 mg in patients with metastatic ccRCC, with dosing in all cohorts occurring over 21-day treatment cycles. Of over 140 patients dosed with casdatifan (with or without cabozantinib), no cases of CCI [redacted] have been observed to date. The available safety data from Study ARC-20 show casdatifan has a similar manageable safety profile as that of belzutifan, an approved class of HIF-2α drugs (Jonasch et al. 2021; Choueiri et al. 2024a; Agarwal et al. 2024).

CCI [redacted]

[redacted]

Risk Assessment for Casdatifan in Combination With Zimberelimab or in Combination With Zimberelimab and Ipilimumab

Zimberelimab is a fully human IgG4 mAb targeting human PD-1 and is being evaluated for the treatment of advanced solid tumors including NSCLC and upper gastrointestinal cancer. Studies consisting of over 500 patients with selected advanced solid tumors showed a safety profile consistent with the class effects of PD-1 inhibitors. Integrated safety data from patients who received zimberelimab as monotherapy or with domvanalimab with or without chemotherapy showed the most commonly reported TEAEs were nausea, fatigue, anemia, diarrhea, and constipation (≥ 20%). The most commonly reported treatment-emergent SAE was pneumonia with hypothyroidism as the most commonly reported immune-mediated adverse events (imAEs). Immune-mediated AEs including immune-mediated skin AEs (including Stevens-Johnson syndrome and toxic epidermal necrolysis), immune-mediated myocarditis, immune-mediated pneumonitis immune-mediated colitis, immune-mediated hepatitis, immune-mediated adrenal insufficiency, immune-mediated encephalitis, immune-mediated nephritis, and immune-mediated thyroid disorders are important identified risks with zimberelimab. Toxicity management guidelines for zimberelimab are provided in Section 6.5.3. More detailed safety analyses for zimberelimab can be found in the most recent IB for zimberelimab.

HIF-2α inhibition, anti-PD-1, and anti-CTLA-4 are complementary mechanisms of action, and the HIF-2a inhibition generally does not have an overlapping toxicity profile compared to the 2 IO agents (anti-PD-1 and anti-CTLA-4). The CheckMate-214 study (a Phase 3, first-line study) investigated advanced ccRCC patients treated with nivolumab in combination with ipilimumab. In the 8-year follow-up Grade 3 and 4 treatment-related AEs occurred in 48.4% of patients, with 22% of patients requiring treatment-related discontinuation due to AEs (Tannir et al. 2024). The most common AEs seen with this treatment combination are imAEs: colitis, hepatitis, pneumonitis, endocrinopathies, and dermatitis. Most imAEs occur early into treatment with 35% of patients requiring high-dose steroids (> 40 mg/day of prednisone equivalent) for treatment-related AEs.

Casdatifan has demonstrated a generally well-tolerated safety profile in monotherapy and in combination with zimberelimab, with AEs largely reflecting the expected, on-target effects of HIF-2α inhibition. In casdatifan monotherapy, the most common treatment-related toxicities include anemia, fatigue, headache, dizziness, diarrhea, and nausea, with Grade ≥ 3 events primarily consisting of anemia and hypoxia (more details of the safety data can be found in the IB). These effects stem from reduced erythropoietin signaling and altered ventilatory regulation, which are intrinsic to HIF-2α blockade and are clinically manageable with supportive care such as erythropoiesis-stimulating agents (ESAs), transfusions, and dose holds.

In another combination with casdatifan, the eVOLVE-RCC02 study is a Phase 1b/III randomized, multicenter, global study sponsored by AstraZeneca, evaluating volrustomig (a bispecific anti-PD-1/anti-CTLA-4 antibody) plus casdatifan or volrustomig monotherapy versus nivolumab plus ipilimumab as first-line treatment for patients with advanced ccRCC. [redacted]

CCI [redacted]

Importantly, no hepatitis or Hy's Law cases were reported in casdatifan monotherapy, and only low-grade transaminase elevations have been observed. When combined with zimberelimab and ipilimumab, casdatifan is not expected to produce significant overlapping toxicity due to the distinct mechanisms of action between HIF-2α inhibitors and anti–PD-1 agents; anemia and hypoxia remain primarily casdatifan-related and are not characteristic toxicities of immune checkpoint inhibitors, supporting a low anticipated risk of additive or synergistic safety concerns.

2.3.2. Benefit Assessment

Study ARC-20 is a multicenter, open-label, Phase 1 study evaluating the safety, PK, preliminary clinical activity, pharmacodynamics, and the biomarkers of casdatifan as monotherapy in patients with advanced solid tumors. It is unknown if patients may benefit from treatment with casdatifan. However, inhibition of HIF-2α has demonstrated clinical activity in patients with tumors having VHL disease in renal cell kidney tumors, and therefore a benefit from class effect may have potential for patients in Study ARC-20 (Jonasch et al. 2021).

As of 30 August 2024, Study ARC-20 early assessment of efficacy shows an unconfirmed ORR (uORR) of 31.3% (confirmed ORR [cORR] of 25.0%) for patients treated with 50 mg BID and an uORR of 25% (cORR of 21.4%) for patients treated with 50 mg QD. Post data extraction, another patient in 50 mg BID had a new response increasing the uORR to 34% for 50 mg BID. The disease control rates are respectively 81% and 86%.

In tumor cells, HIF-2α activates VEGF expression as well as in hypoxic immune cells. The inclusion of a TKI such as cabozantinib that targets VEGFR as well as c-MET and AXL, might block or mitigate the HIF-2α-based VEGF gene expression and result in combined benefits beyond the benefits of monotherapy alone. In addition, combining both casdatifan and cabozantinib will result in downstream impact on other oncogenes that are HIF-2α dependent such as SLC2A1 and CXCR4 and those inhibited by cabozantinib including c-MET and AXL (Choueiri et al. 2023a). Immune checkpoint inhibitors targeting the PD-1/PD-L1 pathway are a centerpiece in the treatment of advanced RCC (Tenold et al. 2020). In the CheckMate-025 study, nivolumab was evaluated against everolimus in patients with metastatic RCC previously treated with a VEGF-TKI. Nivolumab demonstrated a higher response rate (25% vs. 5%) and improvement in median OS (25.8 months vs. 19.7 months) leading to approval as monotherapy in 2L RCC; however, the PFS endpoint was not met (Motzer et al. 2015a; Motzer et al. 2020). Subsequent Phase 3 studies focused on anti-PD-1/PD-L1 mAbs (eg, nivolumab, pembrolizumab, avelumab) in combination with second-generation VEGF-TKIs (axitinib, lenvantinib and cabozantinib) and anti-CTLA-4 (ipilimumab) in treatment-naïve advanced RCC patients, resulting in substantial improvements in ORR, PFS, and OS compared to single-agent sunitinib (Motzer et al. 2019; Rini et al. 2019; Choueiri et al. 2021a; Motzer et al. 2021).

Regarding the benefits of ipilimumab in combination with an anti-PD-1 (ie, nivolumab) for IMDC favorable risk patients, an 8-year follow-up to the CheckMate-214 study demonstrated a final HR of 0.82 with a median OS of 52.7 months for nivolumab in combination with

ipilimumab compared to 37.8 months for sunitinib (Tannir et al. 2024). This delayed benefit pattern led the National Comprehensive Cancer Network (NCCN) guidelines to recognize nivolumab in combination with ipilimumab as the preferred treatment for favorable risk patients in addition to intermediate and poor risk patients.

While anti-PD-1 and anti-PD-L1 combinations with VEGF-TKI agents have demonstrated improved benefit for 1L patients, these combinations have also shown increased toxicities and higher treatment-discontinuation rates over single-agent immunotherapy or VEGF-TKI therapies alone (Rini et al. 2023).

Zimberelimab is a fully human IgG4 mAb targeting human PD-1 and is being evaluated for the treatment of advanced solid tumors including NSCLC and upper gastrointestinal cancer. In ARC-7, evaluating patients with PD-L1 high in the 1L NSCLC setting, zimberelimab demonstrated a 30% ORR with a median PFS of 5.4 months. In a similar setting in ARC-10, zimberelimab showed a cORR of 35%, a median PFS of 6.2 months, and a median OS of 24.4 months (data on file). Nonclinical studies also demonstrated that zimberelimab is similar to currently approved anti-PD-1 agents. Further, zimberelimab has shown to be a clinically equivalent anti-PD-1 antibody to pembrolizumab or nivolumab (see Section 4.1.10 for additional information regarding the comparison between zimberelimab and nivolumab).

Therefore, the combination of zimberelimab and casdatifan may enhance antitumor activity with a more favorable safety profile. Additionally, casdatifan in combination with zimberelimab and ipilimumab may further enhance the antitumor activity in the first-line ccRCC setting.

2.3.3. Overall Benefit-Risk Conclusion

Benefits of treatment with HIF-2α inhibitor therapy have been shown for patients with VHL disease and ccRCC and may potentially benefit patients in Study ARC-20 due to class action effect. This is the first clinical evaluation of casdatifan as monotherapy or in combination with cabozantinib, zimberelimab, or zimberelimab and ipilimumab for potential benefit in patients with solid tumor malignancies and ccRCC. Hence, a comprehensive understanding of the benefits and risks in humans is unknown. Preliminary clinical data showed a manageable safety profile for casdatifan monotherapy and in combination with cabozantinib and zimberelimab, and early signs of efficacy of casdatifan monotherapy in advanced and metastatic ccRCC patients in late lines of treatment and casdatifan with cabozantinib in a post-anti-PD-1/PD(L)-1 setting. Since an accurate estimation of the benefits and risks are unknown, patients will be closely monitored throughout the study, for both safety and potential clinical benefit. The mechanism of action of HIF-2α agent (casdatifan), and a VEGFR-TKI (cabozantinib) or anti-PD-1 (zimberelimab) or anti-CTLA4 (ipilimumab), may provide added antitumor effect beyond monotherapy alone for patients with advanced ccRCC with limited treatment options, supporting a potential favorable benefit-risk profile.

3. OBJECTIVES AND ENDPOINTS

**Primary**	
• To characterize the safety and tolerability of casdatifan monotherapy in patients with advanced solid tumor malignancies and clear cell renal cell carcinoma (ccRCC) during dose escalation<br>• To characterize the safety and tolerability of casdatifan monotherapy in patients with ccRCC<br>• To characterize the safety and tolerability of casdatifan in combination with cabozantinib, zimberelimab, or zimberelimab in combination with ipilimumab in patients with ccRCC	• The incidence of dose-limiting toxicities and adverse events (AEs) in patients with solid tumors during dose escalation<br>• The incidence of AEs in patients with ccRCC during dose expansion monotherapy with casdatifan<br>• The incidence of AEs in patients with ccRCC during combination with casdatifan and cabozantinib, zimberelimab, or zimberelimab in combination with ipilimumab
**Secondary**	
• To assess the clinical activity of casdatifan monotherapy<br>• To assess the clinical activity of casdatifan in combination with cabozantinib, zimberelimab, or zimberelimab in combination with ipilimumab	• Objective response rate as assessed by the investigator according to Response Evaluation Criteria in Solid Tumors (RECIST) v1.1
• To characterize the pharmacokinetics (PK) of casdatifan as a monotherapy and in combination with cabozantinib, zimberelimab, or zimberelimab in combination with ipilimumab	• Plasma concentration and PK parameters for casdatifan after oral dosing of casdatifan as monotherapy and in combination with cabozantinib, zimberelimab, or zimberelimab in combination with ipilimumab
**Exploratory**	
• To assess clinical activity from expansion phase following doses of casdatifan monotherapy in patients with ccRCC<br>• To assess clinical activity from expansion phase following doses of casdatifan in combination with cabozantinib, zimberelimab, or zimberelimab in combination with ipilimumab in patients with ccRCC	• Progression-free survival, defined as the time from date of first dose until progressive disease (PD) or death from any cause, whichever comes first as measured according to RECIST v1.1 as assessed by the investigator<br>• Overall survival, defined as the time from date of first dose until death due to any cause

• CCI [redacted]	• CCI [redacted]
• To investigate the pharmacodynamics of casdatifan as a monotherapy or in combination with cabozantinib, zimberelimab, or zimberelimab in combination with ipilimumab in patients with ccRCC or advanced solid tumor malignancies	• Changes in erythropoietin and other candidate pharmacodynamic biomarkers in the blood and tumor tissues
• To investigate the relationship between the tumor characteristics and clinical activity following casdatifan treatment in patients with solid tumors and ccRCC<br>• To investigate the relationship between the tumor characteristics and clinical activity following casdatifan treatment in combination with cabozantinib, zimberelimab, or zimberelimab in combination with ipilimumab in patients with ccRCC	• Assess the predictive value of biomarkers in tumor tissue and blood and their association with clinical activity
• CCI [redacted]	• CCI [redacted]
• CCI [redacted]	• CCI [redacted]

Enrollment into Expansion Cohorts 6 and 7 will not be conducted in the European Union.

4. STUDY DESIGN

4.1. Overall Design

This is a multicenter, open-label, Phase 1 study evaluating the safety, PK, preliminary clinical activity, pharmacodynamics, and biomarkers of casdatifan as monotherapy in patients with advanced solid tumor malignancies (dose escalation) and as a monotherapy and in combination with cabozantinib, zimberelimab, or zimberelimab in combination with ipilimumab in patients with ccRCC (dose expansion).

This is a non-randomized study that has 2 stages: dose escalation and dose expansion (Figure 6). The dose-escalation stage consists of casdatifan monotherapy cohorts (Escalation Cohorts 1-5). The dose-expansion stage consists of casdatifan monotherapy and combination therapy cohorts (Expansion Cohorts 1-10). The combination therapy cohorts consist of casdatifan in combination with cabozantinib (Expansion Cohort 4), zimberelimab (Expansion Cohort 7), zimberelimab and ipilimumab (Expansion Cohort 9), and with zimberelimab following 4 cycles of zimberelimab and ipilimumab (Expansion Cohort 10). Expansion Cohorts 4, 7, and 9 will begin with a determination of dose and safety for the combinations. Enrollment into Expansion Cohorts 6 and 7 will not be conducted in the European Union (EU).

The dose-escalation and dose-expansion stages consist of 3 study periods:

- Screening: ≤ 28 days before beginning treatment;
- Treatment Period: 21-day cycles until unacceptable toxicity, lost to follow-up, PD, withdrawal from the study, death, or the sponsor closes the study, whichever occurs first (Section 7);
- Post-Treatment Period: 30-Day (and 100-day post-IO for Expansion Cohorts 7, 9, and 10) Post-Study Follow-Up, Post-Treatment Efficacy Follow-Up, and a Long-Term Follow-Up telephone call every 6-months (counting from treatment discontinuation visit) for survival information.

4.1.1. Dose Escalation

4.1.1.1. Casdatifan Monotherapy

Dose escalation will follow a standard 3+3 design (FDA 2011) and will be governed by the occurrence of dose-limiting toxicities (DLTs), which are defined in Section 8.3.5, and by recommendations from the DEC based on review of all available data (evaluation of adverse events [AEs], vital signs, ECGs, tolerability, and clinical laboratory results) from the cohort under consideration. The DLT evaluation for dose escalation is defined as the first 21 days following the first dose of casdatifan. Increasing doses of casdatifan will be administered to patients with a solid tumor who do not have standard-of-care options to identify the casdatifan dose and schedule for dose expansion.

The starting dose of casdatifan is 20 mg administered QD. See Section 4.1.6 for justification of the starting dose. Subsequent doses for dose escalation are indicated below.

- Escalation Cohort 1: 20 mg per day (2 × 10 mg capsules) taken QD by mouth;

- Escalation Cohort 2: 50 mg per day (5 × 10 mg capsules) taken QD by mouth;
- Escalation Cohort 3: 100 mg per day (10 × 10 mg capsules) taken BID (50 mg BID) by mouth (see Section 6.1 for BID timing and dose windows);
- Escalation Cohort 4: 150 mg per day (6 × 25 mg tablets) taken QD by mouth;
- Escalation Cohort 5: 200 mg per day (8 × 25 mg tablets) taken QD by mouth.

Additional dose levels may be evaluated based on emerging safety and/or PK data but will not exceed 100% dose increase from the previous cohort for either the total daily dose or the dose at each dosing event. The highest dose of casdatifan to be tested in dose escalation will be 200 mg.

Approximately 62 patients are anticipated in the dose-escalation phase. This includes a minimum of 15 and up to a maximum of 42 patients that are planned in the 3+3 dose level cohorts (5 proposed cohorts). Additional dose levels may be evaluated if DLT criteria are not met at the proposed cohort dose levels.

An additional 20 patients may be enrolled at any dose level during dose escalation at or below the highest dose cleared in order to collect additional PK, pharmacodynamic, and safety data (Figure 6).

Where a patient is not evaluable against DLT criteria, that patient may be replaced. Replacement of patients must be agreed upon by the sponsor. Refer to Section 7.1 for additional patient withdrawal information.

The DEC will consist of the sponsor medical monitor [or designee] (chair), principal investigator [or designee], lead clinical scientist [or designee], safety representative [or designee], the lead statistician [or designee], lead clinical pharmacologist [or designee] and may include other subject matter experts (internal/external), as necessary.

The DEC will not assign the next dose level until the safety data for the DLT period for all patients from the current dose level have been reviewed, and the committee has determined that safety and tolerability have been demonstrated.

Dose escalation parameters are as follows:

- If 0 of 3 patients experience a DLT, escalate to the next highest dose cohort.
- If 1 of 3 patients experiences a DLT, expand the dose cohort to 6 patients.
- If > 1 patient in a cohort of 3-6 experience a DLT, the maximum tolerated dose (MTD) has been surpassed and the preceding dose cohort will be considered the MTD.

DLT-evaluable patients are defined as those who complete the 21-day DLT period and have received at least 80% of the intended dose of casdatifan or those who experience a DLT.

Additional details for the DEC processes and procedures will be provided in the DEC charter.

4.1.2. Intra-Patient Dose Escalation

For patients who derive clinical benefit at a dose level lower than the highest dose that is deemed safe and tolerable by the DEC, intra-patient dose escalation may be allowed after discussion with the sponsor's medical monitor if:

- Escalation to the next dose level that has been deemed safe is at least 1 dose level below the actively enrolling dose.
- Patients must have signs of clinical benefit (eg, stable disease with some tumor shrinkage, improvement of symptoms, an objective response).
- Patients must have tolerated their current dose level (eg, have not experienced a DLT) and have had only ≤ Grade 2 toxicities during the prior treatment cycle.

The decision to allow for intra-patient dose escalation should be made after considering the potential safety profile of casdatifan, including cumulative drug toxicity. Patients must have completed a minimum of 3 cycles of treatment and all dose level-specific assessments, including PK, pharmacodynamic, and tumor assessments, at the originally assigned dose level before escalation can be considered. Intra-patient dose escalation is not allowed for patients enrolled to dose-expansion cohorts that are evaluating safety and efficacy at a specific dose.

4.1.3. Dose Expansion

Dose-expansion cohorts (Expansion Cohorts 1-10) will consist of casdatifan as a monotherapy (at multiple dose levels and frequencies) and in combination with cabozantinib, zimberelimab, or zimberelimab in combination with ipilimumab. Refer to Table 5 for dose-expansion cohort details. Enrollment into Expansion Cohorts 6 and 7 will not be conducted in the EU.

The dose-expansion phase will consist of a non-randomized and open-label evaluation of the safety and clinical activity of 3 dose levels of casdatifan monotherapy, along with a single dose level of casdatifan in combination with cabozantinib, a single dose level of casdatifan in combination with zimberelimab, and casdatifan in combination with zimberelimab and ipilimumab. Each dose-expansion cohort will enroll up to approximately 30 patients. Expansion Cohorts 4, 7, and 9 will begin with a determination of dose and safety for the combinations.

If any individual dose-expansion cohort demonstrates preliminary signs of efficacy and an acceptable safety profile as determined by the safety review committee (SRC), up to an additional [redacted] patients may be enrolled for a maximum of [redacted] patients in any single dose-expansion cohort. The enrollment of up to an additional [redacted] patients may occur in only 1 dose-expansion cohort or across multiple dose-expansion cohorts.

Table 5: Dose Expansion – Casdatifan Monotherapy and Combination Therapy Cohorts

Cohort No.	Treatment Arm	Population	Dose Level/ Frequency/ Route of Administration
Expansion Cohort 1	Casdatifan monotherapy	2L+ ccRCC	100 mg total daily dose (50 mg BID) PO
Expansion Cohort 2	Casdatifan monotherapy	2L+ ccRCC	50 mg QD PO
Expansion Cohort 3	Casdatifan monotherapy	2L+ ccRCC	150 mg QD PO
Expansion Cohort 4	Casdatifan + cabozantinib combination	2L and 3L (post anti-PD-1/PD-L1 therapy)	Casdatifan 100 mg QD PO + cabozantinib 60 mg QD PO
Expansion Cohort 5	Casdatifan monotherapy	2L+ ccRCC	100 mg QD PO
Expansion Cohort 6 (ex-EU only)	Casdatifan monotherapy	1L favorable-risk ccRCC	100 mg QD PO
Expansion Cohort 7 (ex-EU only)	Casdatifan + zimberelimab combination	1L ccRCC	Casdatifan 100 mg QD PO + zimberelimab 360 mg Q3W IV infusion[a]
Expansion Cohort 8	Casdatifan monotherapy	2L post-IO ccRCC	100 mg QD PO
Expansion Cohort 9	Casdatifan + zimberelimab + ipilimumab followed by casdatifan + zimberelimab	1L ccRCC	Casdatifan 100 mg QD PO + zimberelimab 3 mg/kg IV Q3W + ipilimumab 1 mg/kg IV Q3W for 4 cycles, followed by casdatifan 100 mg QD + zimberelimab 360 mg Q3W for 2 cycles, followed by casdatifan 100 mg QD + zimberelimab 720 mg Q6W
Expansion Cohort 10	Zimberelimab + ipilimumab followed by casdatifan + zimberelimab	1L ccRCC	Zimberelimab 3 mg/kg IV Q3W + ipilimumab 1 mg/kg IV Q3W for 4 cycles followed by casdatifan 100 mg QD and zimberelimab 360 mg Q3W for 2 cycles followed by casdatifan 100 mg QD and zimberelimab 720 mg Q6W

1L = first-line; 2L = second-line; 3L = third-line; anti-PD-1/PD-L1 = anti–programmed-cell-death protein-1/anti–programmed cell death-ligand 1; BID = twice daily; ccRCC = clear cell renal cell carcinoma; EU = European Union; IO = immune-oncology; PO = orally; Q3W = every 3 weeks; Q6W = every 6 weeks; QD = once daily; IV = intravenous

[a] Following 1 year on study treatment (starting on Cycle 18) patients will move to zimberelimab at 720 mg IV infusion every 6 weeks (Q6W)

4.1.3.1. Casdatifan Monotherapy

Refer to Section 5.1 and Section 5.2 for detailed patient eligibility requirements for the dose-expansion cohorts.

Any casdatifan monotherapy dose-expansion cohort may start prior to the completion of dose escalation based on emerging safety and PK/pharmacodynamic data; however, the dose selected must have cleared DLT evaluation. The highest dosage of casdatifan in the monotherapy expansion phase will not exceed the highest dosage or dosage intensity cleared for safety in the casdatifan monotherapy escalation phase. Patients receiving capsules can receive the same milligram dose delivered by tablets.

The optimal casdatifan monotherapy dose established in Expansion Cohorts 1-3 will be used to initiate subsequent dose-expansion cohorts.

- Expansion Cohort 1 patients will take 100 mg orally (PO) (50 mg BID) and may begin enrolling after the DECs review of the safety, PK, and pharmacodynamic data in the dose-escalation phase of the study. The first 6 patients enrolled in this cohort will be reviewed by the SRC after the first cycle has been completed for early safety assessment and trends. Enrollment will continue during the safety review of the first 6 patients unless stopping criteria have been reached.
- Expansion Cohort 2 patients will take the 50 mg PO (50 mg QD) and may begin enrolling after the DECs review of the safety, PK, and pharmacodynamic data in the dose-escalation phase of the study.
- Expansion Cohort 3 patients will take a total daily dose level 150 mg QD and may begin enrolling after the DECs review of the safety, PK, and pharmacodynamic data in the dose-escalation phase of the study.

4.1.3.2. Expansion Cohort 4

Expansion Cohort 4 will evaluate casdatifan at a target dose of 100 mg QD in combination with the approved 60 mg QD monotherapy dose of cabozantinib. To ensure patient safety, the initial casdatifan dose evaluated in combination with cabozantinib will be at least 1 dose level lower than the highest casdatifan monotherapy dose determined to be safe in dose escalation, as described below. The highest dosage of casdatifan in the combination therapy dose determination phase will not exceed the highest dosage or dosage intensity cleared for safety in the casdatifan monotherapy phase.

Dose Determination for Expansion Cohort 4

Prior to fully enrolling Expansion Cohort 4, safety will be evaluated for the first 6 patients to complete 21 days of treatment (safety run-in phase) at a given casdatifan dose level (Figure 3). The safety review will be conducted per DLT criteria to assess AEs, including clinically significant laboratory findings.

Figure 3: Expansion Cohort 4 Dose Determination Flowchart

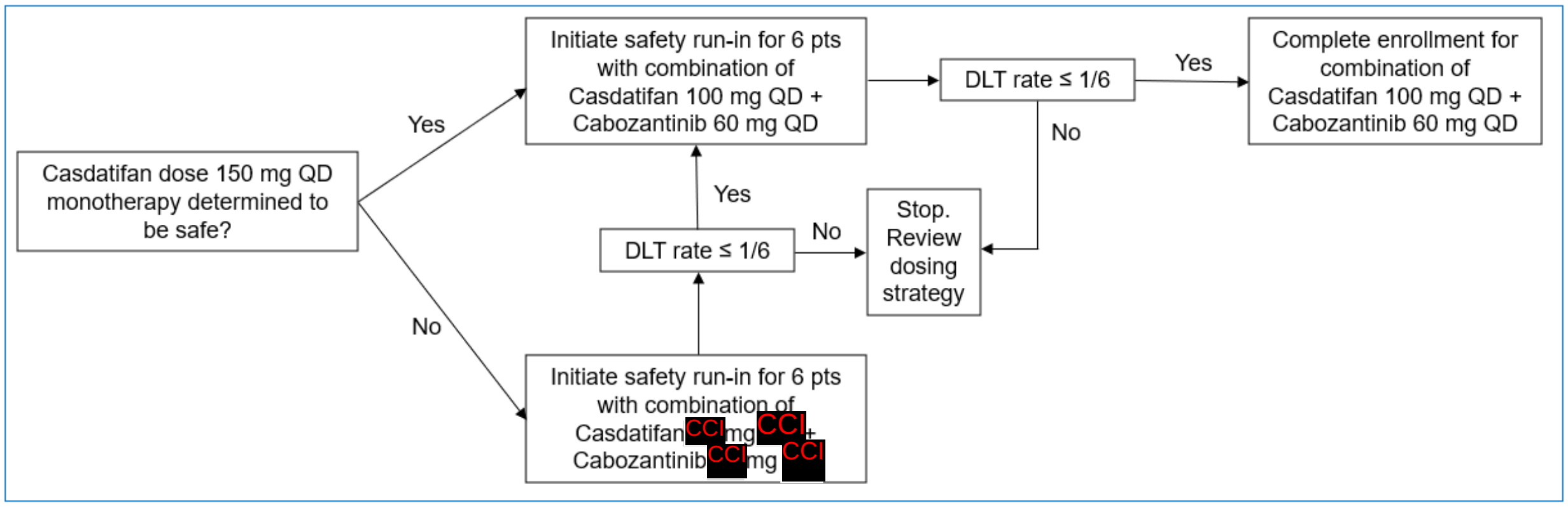

QD = once daily; pts = patients; DLT = dose-limiting toxicity

A lower casdatifan dose level (ie, [redacted] mg) in combination with [redacted] mg or [redacted] mg QD cabozantinib may be evaluated as a separate cohort of approximately 30 patients based on emerging safety and/or PK data from the 100 mg QD casdatifan and 60 mg QD cabozantinib combination therapy.

4.1.3.3. Expansion Cohort 7 (ex-EU only)

Expansion Cohort 7 will evaluate casdatifan at a starting dose of 100 mg QD in combination with zimberelimab at 360 mg IV infusion every 3 weeks (Q3W). Following 1 year on study treatment (starting on Cycle 18) patients will move to zimberelimab at 720 mg IV infusion every 6 weeks (Q6W). The maximum treatment duration for zimberelimab is 2 years. However, patients are allowed to continue treatment with casdatifan until any of the criteria for permanent discontinuation of study treatment are met, as described in Section 7.1.

Prior to fully enrolling Expansion Cohort 7, safety will be evaluated for the first 6 patients to complete 21 days of treatment (safety run-in phase). The safety review will be conducted per DLT criteria to assess AEs, including clinically significant laboratory findings.

A lower casdatifan dose level (ie, [redacted] mg) in combination with 360 mg Q3W zimberelimab may be evaluated as a separate cohort of approximately 30 patients based on emerging safety data from the 100 mg QD casdatifan and 360 mg Q3W zimberelimab combination therapy.

4.1.3.4. Expansion Cohort 9

Expansion Cohort 9 will evaluate casdatifan at the standard starting dose of 100 mg QD in combination with zimberelimab at 3 mg/kg IV Q3W and ipilimumab at 1 mg/kg IV Q3W for 4 cycles, followed by casdatifan 100 mg QD and zimberelimab 360 mg Q3W for 2 cycles, followed by casdatifan 100 mg QD and zimberelimab 720 mg Q6W. The maximum treatment

duration for zimberelimab is 2 years; however, patients are allowed to continue treatment with casdatifan beyond 2 years until any of the criteria for permanent discontinuation of study treatment are met.

Expansion Cohort 9 will begin with a safety evaluation for the first 6 patients who complete a minimum of 28 days on treatment (safety run-in phase). The safety review will be conducted by the SRC per DLT criteria to assess AEs, including clinically significant laboratory findings. If < 2 DLTs in the first 6 patients are observed, the cohort will be expanded to up to 30 patients. The sponsor may consider evaluating the triplet combination with casdatifan at lower doses based on recommendations from the SRC.

DLT-evaluable patients are defined as those who complete the 28-day DLT period and have received at least 80% of the intended dose of casdatifan or those who experience a DLT.

Any dose that clears the safety run-in phase may be expanded further to enroll up to an additional 30 patients at that dose level. Additionally, up to 30 patients may be enrolled at a lower dose of casdatifan than the highest dose that clears the safety run-in phase.

4.1.3.5. Expansion Cohort 10

Expansion Cohort 10 will evaluate zimberelimab at 3 mg/kg IV Q3W and ipilimumab at 1 mg/kg IV Q3W for 4 cycles followed by casdatifan 100 mg QD and zimberelimab 360 mg Q3W for 2 cycles followed by casdatifan 100 mg QD and zimberelimab 720 mg Q6W. The maximum treatment duration for zimberelimab is 2 years. However, patients are allowed to continue treatment with casdatifan beyond 2 years until any of the criteria for permanent discontinuation of study treatment are met.

Patients in Expansion Cohort 10 that have [redacted] on the combination with zimberelimab and ipilimumab may remain on treatment but will discontinue ipilimumab and transition to casdatifan 100 mg QD and zimberelimab 360 mg Q3W for 2 cycles followed by casdatifan 100 mg QD and zimberelimab 720 mg Q6W. Casdatifan at 100 mg QD can begin immediately following [redacted] while the next dose of zimberelimab should be consistent with Q3W dosing from the previous dose. All patients continuing study treatment with casdatifan in combination with zimberelimab after [redacted] on the combination therapy with zimberelimab and ipilimumab must continue tumor assessments at the same interval, until [redacted] and/or loss of clinical benefit as judged by the investigator.

4.1.4. Stopping Criteria for Dose Expansion

Dose expansion stopping rules for each cohort are as follows:

- ≥ 10% treatment-related Grade 5 AE, or
- Occurrence of 2 or more non-laboratory Grade 4 events that are related to study agent, or
- Grade 4 treatment-related hypoxia or anemia events in ≥ 25% of patients

In the event stopping criteria are met, the cohort and any cohort utilizing a higher dose will be placed on enrollment hold, and a decision about the cohort and higher dosing cohorts will be made following review of the cumulative safety data for the cohort by the SRC. The study design

or study conduct may be modified prior to further enrollment to the cohort. Where criteria are met across 2 or more cohorts, study enrollment will be halted across all cohorts until the cumulative safety data for the study is reviewed by the SRC.

4.1.5. Scientific Rationale for Study Design

This is a Phase 1, open-label, dose-escalation and dose-expansion, first-in-patient study to evaluate the safety, tolerability, PK, pharmacodynamic, and clinical activity of casdatifan (an inhibitor of HIF-2α) as a monotherapy and in combination with cabozantinib, zimberelimab, or zimberelimab in combination with ipilimumab in patients with advanced solid tumors (dose escalation) and advanced ccRCC (dose expansion). The rationale for the inclusion of solid tumors and ccRCC is found in Table 6.

Treatment options for patients with advanced RCC who have experienced PD following treatment with anti-PD-1 or anti-PD-L1 are primarily single-agent VEGFR-TKIs, where responses are limited to 25% to 30% of patients. HIF-2α plays a central role in the progression of RCC tumors, and combining a HIF-2α-inhibitor with a VEGFR-TKI could provide more antitumoral activity than either agent alone. An ongoing investigational study exploring the combination of cabozantinib with belzutifan, a HIF-2α inhibitor similar to casdatifan, has demonstrated good tolerability along with promising antitumoral effects when administered together (Choueiri et al. 2023a). The sponsor proposes that the exploration of casdatifan in combination with cabozantinib is warranted on the unmet need in these previously treated PD-1-based or anti-PD-L1 patients and promising results to date. In addition, the sponsor proposes that the evaluation of casdatifan in combination with zimberelimab could provide an additional treatment option with a more favorable safety profile for patients with 1L ccRCC.

First-line treatment for patients with advanced RCC either include anti-PD-1/PD-L1 therapy in combination with a VEGFR-TKI or with an anti-CTLA-4. For patients treated with anti-PD-1/anti-CTLA-4 combination therapy of nivolumab in combination with ipilimumab in the CheckMate-214 study, response rates were 42% (all IMDC risk) and PFS was 11.6 months (Choueiri et al. 2025). Given the significant improvements in efficacy observed with HIF-2α inhibitors in later line settings and the distinct safety profile observed with HIF-2α monotherapy when compared to IO therapies, the sponsor proposes exploration of 2 combination regimens adding casdatifan to first-line standard-of-care: ipilimumab in combination with nivolumab (anti-PD-1) followed by nivolumab:

- casdatifan with anti-PD1 zimberelimab and ipilimumab followed by casdatifan and zimberelimab
- zimberelimab and ipilimumab followed by casdatifan and zimberelimab

Table 6: Rationale for Inclusion of Tumor Types

Tumor Type	Scientific Justification	Reference(s)
Solid tumors	HIF-2α may have a role in other tumor types through upregulation of tumor-promoting genes and pathways. Non-small cell lung cancer, hepatoma and breast cancer have been the target of potential therapies that target inhibition of HIF-2α through prevention of accumulation; inhibition of dimerization of the HIF-2α-ARNT pathway; prevention of transcription activation; and inhibition of HIF-2α by promoting interaction of HIF-2α mRNA with IRP1 and subsequent repression of chemoresistance.	Yang et al. 2020
ccRCC	ccRCC is the most common RCC histologic subtype with known association with mutation of the VHL gene resulting in accumulation of HIF-2α and upregulation of genes involved with tumorigenesis. Suppression of HIF-2α in patients with ccRCC results in the suppression of tumorigenic genes and more favorable outcomes of progression-free survival.	Choueiri et al. 2021a

ARNT = aryl hydrocarbon receptor nuclear translocator; ccRCC = clear cell renal cell carcinoma; HIF-2α = hypoxia inducible factor 2α; IRP1 = iron regulatory protein 1; mRNA = messenger ribonucleic acid; RCC = renal cell carcinoma; VHL = von Hippel-Lindau

4.1.6. Justification for Casdatifan Starting Dose for Escalation

The proposed starting dose of casdatifan at 20 mg QD is expected to be safe and well-tolerated in patients with solid tumors. This dose was selected based on the totality of nonclinical data (including 28-day GLP toxicology studies), in compliance with International Conference for Harmonisation (ICH) S9 guidance, and the clinical PK and pharmacodynamic data from the first-in-human study in healthy participants (ARC-14).

As of 28 April 2022 (prior to the start of Study ARC-20), casdatifan was given to a total of 18 healthy participants at 3 mg, 10 mg, or 30 mg single oral dose in Study ARC-14 and was well tolerated with no safety events of concern. Of these 18 healthy participants dosed with casdatifan, 4 cases of headache and 1 case of sleepiness (all Grade 1) were attributed to the study drug (see the IB for further details). Pharmacokinetic data indicated that exposure (area under plasma concentration-time curve from time zero extrapolated to infinity) increased in an approximately dose-proportional manner between 3 mg and 30 mg. Mean apparent terminal half-life is approximately 18 to 20 hours. Note that updated PK data are summarized in Section 2.2.2. Pharmacodynamic data suggested pharmacological effects at dose levels ≥ 10 mg, with reduction in EPO concentrations observed for the first 48-72 hours post-dose. Note that reduction in EPO is an expected pharmacological effect from on-target inhibition of the EPO gene by casdatifan.

According to ICH S9 guidance, the starting dose for anticancer pharmaceuticals may be selected based on 1/6 the highest non-severely toxic dose (HNSTD) in non-rodent species or 1/10 the STD10 in rodents. From the 28-day GLP toxicology studies, the HNSTD was 20 mg/kg/day in dogs (human equivalent dose = 11.1 mg/kg), and the STD10 was > 100 mg/kg/day in rats (human equivalent dose = 16.1 mg/kg). Therefore, the starting dose may be as high as 110 mg based on 1/6 HNSTD (ie, 1.9 mg/kg assuming 60 kg body weight) or 97 mg based on 1/10 STD10 (ie, 1.6 mg/kg assuming 60 kg body weight). However, a lower starting dose of 20 mg is proposed as this dose level is expected to have pharmacologic effects based on the PK and pharmacodynamic data from the first-in-human study for casdatifan (ARC-14). In addition, the proposed subsequent dose levels of 50 and 100 mg per day are below or similar to the starting doses calculated by 1/6 HNSTD (110 mg) or 1/10 STD10 (97 mg); therefore, these 2 proposed subsequent dose levels are also expected to be well-tolerated. In summary, the proposed starting dose of 20 mg QD is expected to be safe and well-tolerated and have pharmacological effects.

4.1.7. Justification for Dose Levels in Expansion Cohorts (Casdatifan Monotherapy)

The casdatifan monotherapy expansion cohorts (Expansion Cohorts 1-3) will test up to 3 dose levels of casdatifan: 50 mg QD, 50 mg BID, and a higher dose level to be determined based on emerging safety and PK data in the dose-escalation portion of the current study.

Preliminary pharmacodynamic results for casdatifan included reductions in serum EPO, indicating potent HIF-2α inhibition by casdatifan. A preliminary population PK/pharmacodynamic model was developed using the available PK and EPO data from ARC-14 and ARC-20 studies. The PK/pharmacodynamic model simulations suggested that a 100 mg QD (or 50 mg BID) casdatifan dose would yield maximal EPO reduction of ≥ 90% in the majority of patients, an extent of pharmacodynamic response typically associated with optimal clinical efficacy and safety. A lower dose of 50 mg QD is predicted to yield EPO reduction of ~90% in some of the patients, indicating that this dose is also likely to be clinically active. A third dose level, 150 mg daily, was identified in further dose-escalation cohorts that are deemed to be safe and provide sufficiently different exposures compared to 50 mg BID to give the best opportunity to identify overall trends in exposure versus safety/efficacy response.

4.1.8. Justification for Dose Levels in Expansion Cohort 4

The starting dose of casdatifan for Expansion Cohort 4 is 1 dose level lower than the highest casdatifan monotherapy dose determined to be safe in the ongoing dose-escalation portion of the current study.

The starting dose for cabozantinib in Expansion Cohort 4 is 60 mg QD. This dosage of cabozantinib is the approved single-agent dosage for the treatment of patients with RCC, hepatocellular carcinoma, and differentiated thyroid cancer (CABOMETYX [cabozantinib] Prescribing Information).

In addition to cabozantinib approval for use as monotherapy, the 60 mg QD of cabozantinib was the dose used in combination with 120 mg QD belzutifan in the ongoing Phase 2 study in patients with both 1L and 2L ccRCC (Choueiri et al. 2023a). The resulting safety profile for the combination is consistent with that previously observed for the single agents. Most treatment-related AEs were Grade 1 or 2. Grade 3 treatment-related AEs occurred in 37% and 63% of patients with treatment-naive and previously treated RCC, respectively. The most common Grade 3 treatment-related AEs were anemia, fatigue, PPE, and hypertension. There were no Grade 4 treatment-related AEs; there was 1 Grade 5 event of respiratory failure that was considered possibly treatment-related by the investigator. Overlapping AEs such as fatigue, nausea, hypertension, and anemia were manageable.

In addition, based on the PBPK modeling analysis using available data for cabozantinib (CABOMETYX [cabozantinib] Prescribing Information) and casdatifan, a clinically relevant DDI between cabozantinib and casdatifan is not expected to occur when coadministering both compounds. Therefore, dose adjustment for possible DDI is not necessary.

4.1.9. Justification for Dose Levels in Expansion Cohort 7

The starting dose of casdatifan for Expansion Cohort 7 is 100 mg QD, which is [redacted]

The starting dose for zimberelimab in Expansion Cohort 7 is 360 mg Q3W up to 1 year, followed by 720 mg Q6W (starting on Cycle 18). Zimberelimab dosage of 360 mg Q3W is the recommended Phase 2/3 dose for the Q3W dosing schedule and has been shown to be safe and efficacious as a single agent or in combination with domvanalimab with or without chemotherapy agents in multiple cancer populations. At this dose level, the steady state trough concentrations of zimberelimab are maintained above the target concentration of 15 μg/mL in a vast majority of patients. One year after treatment, the zimberelimab dose will be changed to 720 mg Q6W. Available data (Study AB154CSP0001) have shown that this dose of zimberelimab is safe and well-tolerated in patients with advanced malignancies. Population PK simulations indicate this dose will also maintain zimberelimab concentrations above the effective levels (Figure 4). In addition, the reduced frequency of dosing will provide increased convenience for patients and caregivers.

Considering that zimberelimab is a monoclonal antibody mainly eliminated through proteolysis, it is unlikely that its systemic exposure will be affected by casdatifan coadministration. Similarly, zimberelimab is not expected to affect the CYP enzymes involved in metabolism of casdatifan. Therefore, the likelihood of a PK DDI between casdatifan and zimberelimab is low.

As a result, no dose adjustments to account for any PK DDI would be required when casdatifan and zimberelimab are coadministered.

Additional information on PK, anti-drug antibodies (ADA), safety, and efficacy of zimberelimab can be found in the most recent version of the zimberelimab IB.

Figure 4: Population PK Simulation of Zimberelimab in Patients With Cancer

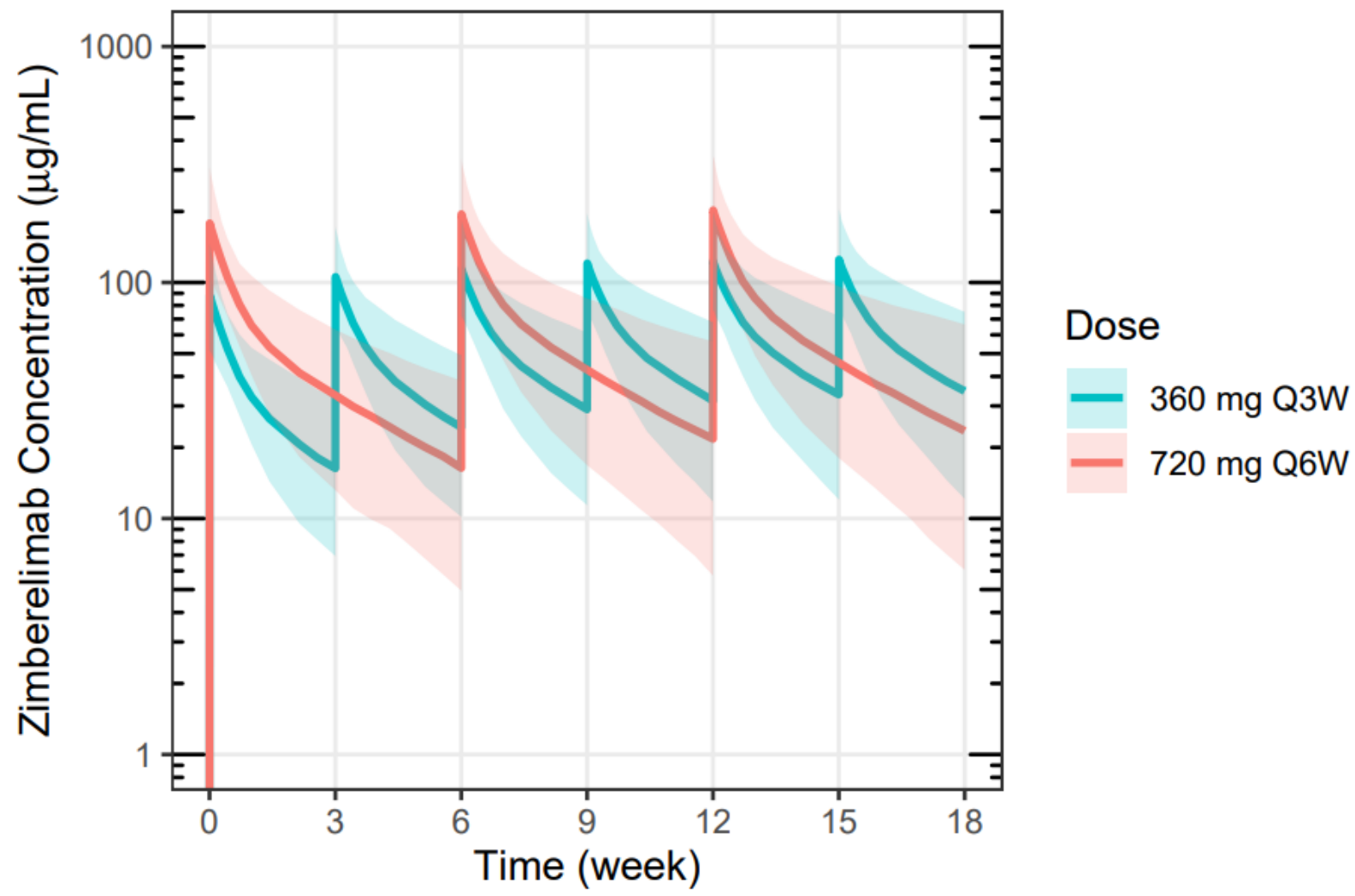

PK = pharmacokinetics; QXW = every X weeks.
Note: Solid line indicates population median. Shaded areas indicate 5th to 95th quantile of interindividual variability in predicted zimberelimab concentrations.

4.1.10. Justification for Treatments and Dose Levels in Expansion Cohorts 9 and 10

The starting dose of casdatifan for Expansion Cohorts 9 and 10 is 100 mg QD. Casdatifan monotherapy at doses up to 150 mg QD has been shown to be safe and tolerable in patients with RCC. No MTD of casdatifan has been identified. The 100 mg casdatifan dose has also demonstrated adequate tolerability in combination with both cabozantinib and zimberelimab (refer the casdatifan IB for safety data). The starting dose of casdatifan at 100 mg QD has been identified as the optimum dose for a Phase 3 study based on the totality of the available data including casdatifan safety, efficacy, PK/pharmacodynamic, and ER analyses.

The dose for ipilimumab in Expansion Cohorts 9 and 10 is 1 mg/kg Q3W for a maximum of 4 doses. This dosage of ipilimumab is the approved dosage in combination with nivolumab for the first-line treatment of patients with RCC (YERVOY [ipilimumab] Prescribing Information).

The starting dose for zimberelimab in Expansion Cohorts 9 and 10 during combination with ipilimumab is 3 mg/kg Q3W for 4 doses. This dosage is the mg-equivalent of nivolumab dosage when combined with ipilimumab for the first-line treatment of patients with RCC (OPDIVO [nivolumab] Prescribing Information). Available PK data for zimberelimab and nivolumab at 240 mg indicated similar exposures for both compounds – $C_{avg\ 0\text{-}Day14}$ of 29.4 µg/mL versus 28.1 µg/mL for zimberelimab (Study AB154CSP0001 CSR) and nivolumab (Zhao et al. 2017), respectively. [redacted] Clinical efficacy data have also indicated a similar efficacy profile of

zimberelimab versus nivolumab at equivalent doses (see Section 2.2.1 for comparison between zimberelimab and nivolumab). Overall, these data support the use of zimberelimab 3 mg/kg as the equivalent dose of nivolumab 3 mg/kg in combination with ipilimumab.

The dose for zimberelimab in Expansion Cohorts 9 and 10, after completing the combination period with ipilimumab, is 360 mg Q3W for 2 doses followed by 720 mg Q6W. Zimberelimab dosage of 360 mg Q3W is the recommended Phase 2/3 dose for the Q3W dosing schedule, and has been shown to be safe and efficacious as a single agent or in combination with other agents. At this dose level, the steady state trough concentrations of zimberelimab are maintained above the target concentration of 15 µg/mL in a vast majority of patients. After 6 cycles of treatment, the zimberelimab dose will be changed to 720 mg Q6W. Available data (Study AB154CSP0001 CSR) have shown this dose of zimberelimab is safe and well-tolerated in patients with advanced malignancies. Population PK simulations indicate this dose also will maintain zimberelimab concentrations above the effective levels (Figure 4). In addition, the reduced frequency of dosing will provide increased convenience for patients and caregivers. Furthermore, the zimberelimab doses of 360 mg Q3W and 720 mg Q6W are similar mg/week doses as nivolumab 240 mg Q2W and 480 mg Q4W, the doses of nivolumab used in this patient population (nivolumab package insert).

Considering zimberelimab and ipilimumab are monoclonal antibodies mainly eliminated through proteolysis, it is unlikely that their systemic exposure will be affected by casdatifan coadministration. Similarly, neither zimberelimab nor ipilimumab is not expected to affect the CYP enzymes involved in metabolism of casdatifan. Therefore, the likelihood of a PK DDI between casdatifan and zimberelimab or ipilimumab is low. As a result, no dose adjustments to account for any PK DDI would be required when casdatifan, zimberelimab, and ipilimumab are co-administered.

Additional information on PK, ADAs, safety, and efficacy of zimberelimab can be found in the most recent version of the zimberelimab IB.

4.1.11. Justification for Inclusion of Patients With Renal Impairment

In order to allow a broader cancer patient population to have access to study treatment, Study ARC-20 (as of Protocol version 3.0) plans to enroll patients with moderate renal impairment of creatinine clearance (CL_{CR}) ≥ 40 mL/min (Cockcroft and Gault 1976). The rationale for this inclusion criteria is further discussed as follows.

As of 16 March 2023, preliminary casdatifan urine PK data are available for 27 patients/participants including 24 healthy participants in Study ARC-14 (who received either a single oral dose of casdatifan at 100 mg, or multiple oral doses of casdatifan at 15, 30, or 50 mg QD), and 3 patients in Study ARC-20 (who received multiple oral doses of casdatifan at 20 mg QD). Casdatifan renal clearance ranged from 14.2 to 25.8 mL/min at dose levels of 15-100 mg (n = 3-7 per dose level) and was similar across all dose levels.

A population PK model was developed using the casdatifan plasma and urine PK data. The model was able to describe the plasma and urine PK profiles well across all dose levels. Statistically significant covariate included body weight on volume of distribution and CL_{CR} on renal clearance. The population PK model parameters, including the renal clearance, are presented in Table 7.

Table 7: Population Pharmacokinetic Parameters of Casdatifan Model

Parameter	Parameter Estimate (%RSE)
Apparent Non-Renal Clearance (CLnr/F) (L/h)	3.32 (6)
Renal Clearance (CLr) (L/h)	1.30 (4)
Apparent Volume Distribution (V/F) (L)	141 (3)
Absorption Rate Constant (Ka) (1/h)	1.9 (19)
Relative Bioavailability at 100 mg ($F_{100\ mg}$)	0.75§
Covariate Parameters	
Weight on V/F ($\alpha_{V/F\text{-}WT}$)	0.804 (19)
CL_{CR} on CLr ($\alpha_{CLr\text{-}CL_{CR}}$)	1§
Interindividual Variability (%CV)	
$\eta_{CLnr/F}$	42 (10)
η_{CLr}	21 (17)
$\eta_{V/F}$	19 (12)
η_{ka}	118 (13)
Residual Error (%CV)	
Proportional Error (additive log-transformed) on plasma data	25 (9)
Proportional Error (additive log-transformed) on urine data	11 (12)

§ The parameter was fixed at the value presented and not estimated by the model.
%CV = percentage coefficient of variation; $F_{100\ mg}$ = Relative bioavailability at 100 mg compared doses ≤ 50 mg; PK = pharmacokinetic(s); RSE = relative standard error
The following η-correlations were estimated (CV%): CLnr/F-CLr = -5%, CLnr/F-V/F = 43%, CLr-V/F = 11%.
Equations for the PK parameters were as follows:
$CLnr/F_{,i} = CLnr/F \times \exp(\eta_{CLnr/F})$
$CLr_{,i} = CLr \times (CL_{CR,i}/123.2\ mL/min)^{\alpha_{CLr\text{-}CL_{CR}}} \times \exp(\eta_{CLr})$
$Ka_{,i} = Ka \times \exp(\eta_{Ka})$
$V/F_{,i} = V/F \times (WT_{,i}/67.8\ kg)^{\alpha_{V/F\text{-}WT}} \times \exp(\eta_{V/F})$
$F = 1 \times F_{100\ mg}$ (at 100 mg)

Simulations were performed using the population PK model to compare the exposure between 2 categories of normal renal function ($CL_{CR} \geq 90$ mL/min) and moderate renal impairment with $40 \leq CL_{CR} < 60$ mL/min based on the calculated CL_{CR} data (Cockcroft and Gault 1976) from the National Health and Nutrition Examination Survey (NHANES) database 2009 to 2018. Simulation results indicate that the exposure measures (average concentration at steady state and C_{max}) in patients with moderate renal impairment $CL_{CR} \geq 40$ mL/min are approximately 20% higher than that in patients with normal ($CL_{CR} \geq 90$ mL/min) renal function (Table 8 and Figure 5). This difference is within the range of casdatifan PK variability seen in ongoing studies (Table 2). In addition, casdatifan has been shown to be safe and well-tolerated in patients with

solid tumors at doses up to 50 mg QD so far. Therefore, this predicted small increase in exposure in patients with $CL_{CR} \geq 40$ mL/min is expected to be clinically insignificant, thus supporting inclusion of moderate renally impaired patients in StudyARC-20 moving forward. This will also allow a broader renal cancer patient population to have access to this novel therapeutic candidate.

Furthermore, nonclinical data in bile duct canulated male Sprague-Dawley rats indicated that 4.9% of the administered dose was recovered as unchanged casdatifan in urine following a single intravenous (1.0 mg/kg) dose of casdatifan. This nonclinical data also suggest that renal excretion does not play a major role in casdatifan elimination.

Table 8: Comparison of Predicted Exposures for Casdatifan in Patients With Normal ($CL_{CR} \geq 90$ mL/min) and Moderate Impairment Renal Function ($CL_{CR} \geq 40$ mL/min)

Dose	PK Exposure (Steady-State)	Casdatifan Geometric Mean, ng/mL (%CV)		GM Exposure Ratio of Renal Impairment vs Normal: Moderate $CL_{CR[40-60)}$ / Normal $CL_{CR[90-)}$
		Normal Renal Function $CL_{CR} \geq 90$ mL/min	Moderate Renal Impairment $40 \leq CL_{CR} < 60$ mL/min	
50 mg QD	C_{avg}	442 (32)	531 (37)	1.20
	C_{max}	583 (27)	672 (31)	1.15
50 mg BID	C_{avg}	887 (31)	1066 (36)	1.20
	C_{max}	1003 (29)	1183 (34)	1.18
100 mg BID	C_{avg}	1328 (32)	1596 (37)	1.20
	C_{max}	1501 (29)	1770 (34)	1.18

%CV = percentage coefficient of variation; AUC_{tau} = area under plasma concentration-time curve from 1 dosing interval; BID = twice daily; C_{avg} = average concentration at steady state; CL_{CR} = creatinine clearance; C_{max} = peak concentration at steady state, GM = geometric mean; PK = pharmacokinetics; QD = once daily
Note: C_{avg} is calculated using the formula AUC_{tau}/tau.

Figure 5: Population PK Simulations of Casdatifan at Steady State at 50 mg QD, 50 mg BID, and 100 mg BID in Normal ($CL_{CR} \geq 90$ mL/min) and Moderate Impairment ($CL_{CR} \geq 40$ mL/min) Renal Function

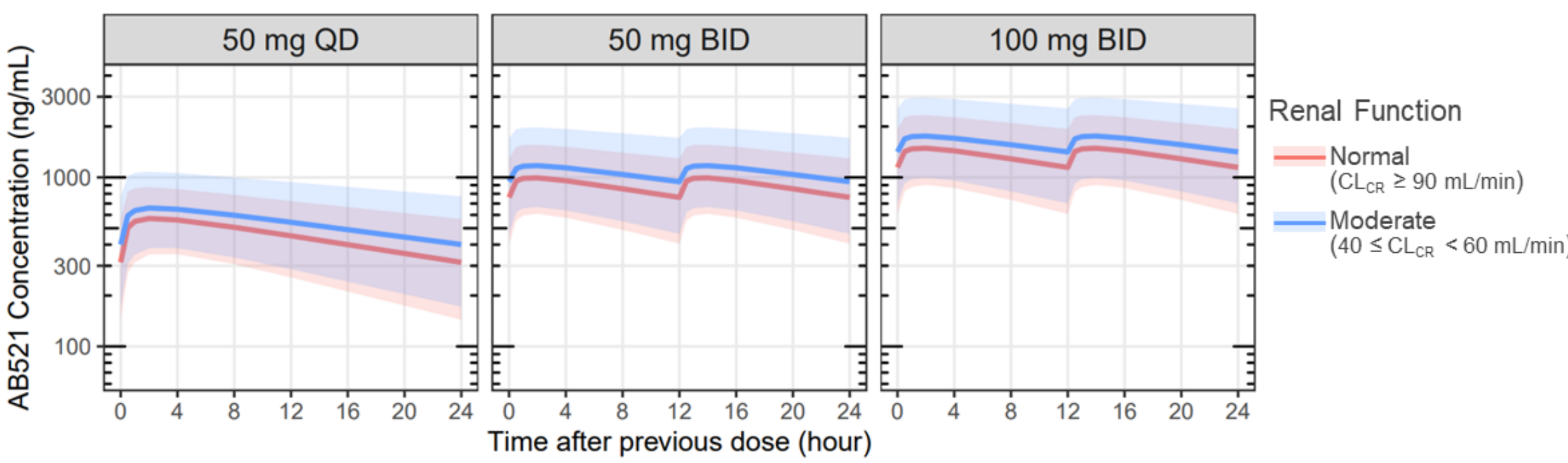

AB521 = casdatifan; BID = twice daily; CL_{CR} = creatinine clearance; PK = pharmacokinetic; QD = every day

Solid lines indicate population median. Shaded areas indicate 5th to 95th quantile of interindividual variability in predicted casdatifan concentrations.

4.1.12. End of Study Definition

The end of the study is defined as the date on which the last patient in the clinical study was evaluated for the primary, secondary, or exploratory outcome measures.

A patient is considered to have completed the study if he/she has completed all periods of the study including the last visit or the last scheduled procedure shown in the SoA, has died, is lost to follow-up, or withdraws consent to participate.

4.2. Study Scheme and Schedule of Activities

4.2.1. Study Design

Figure 6: Schema

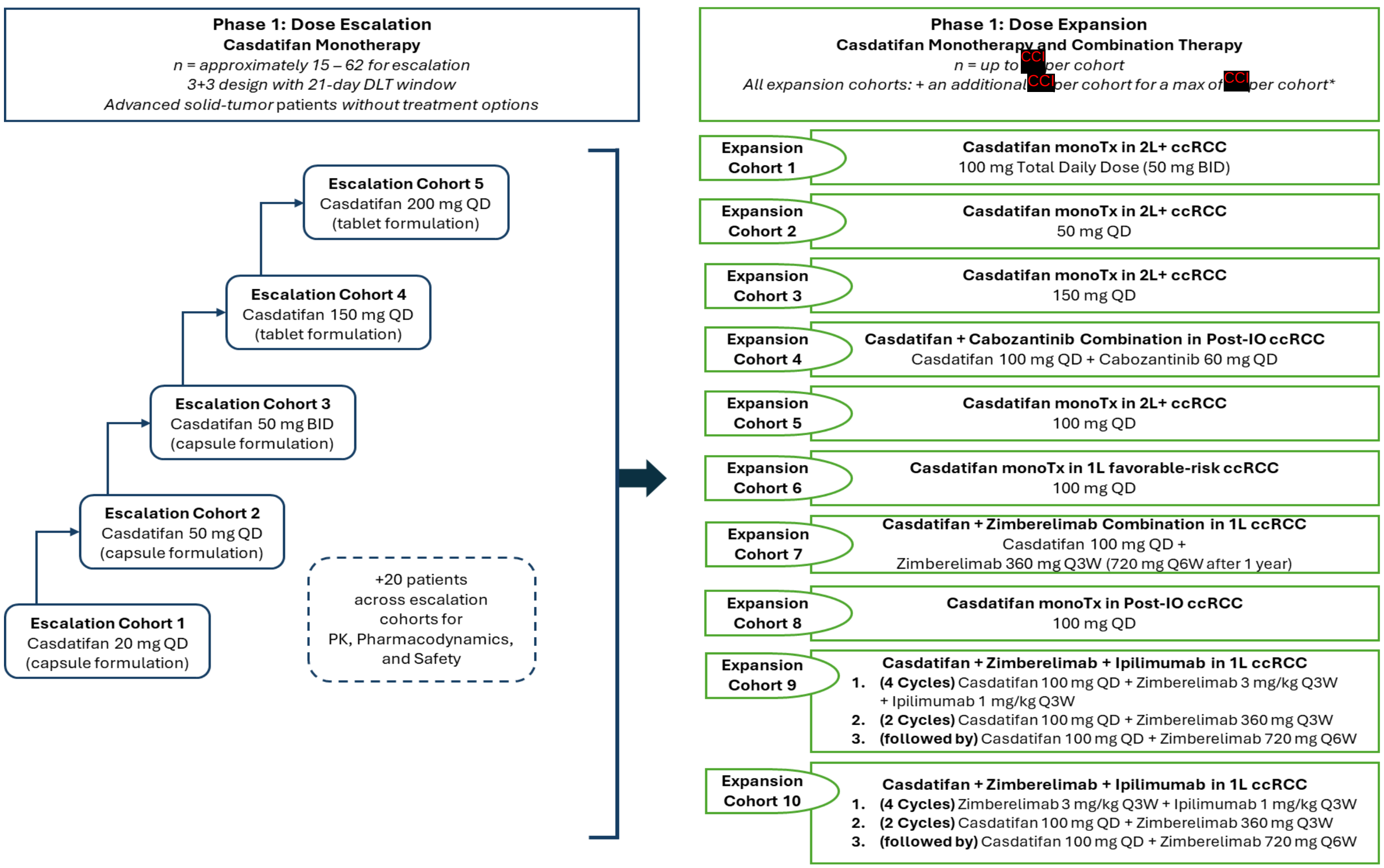

1L = first-line; 2L = second-line; BID = twice daily; ccRCC = clear cell renal cell carcinoma; DLT = dose-limiting toxicity; IO = immune-oncology; monoTx = monotherapy treatment; n = number; PK = pharmacokinetics; Q3W = every 3 weeks; Q6W = every 6 weeks; QD = once daily
Enrollment into Expansion Cohorts 6 and 7 will not be conducted in the European Union. The maximum treatment duration for zimberelimab is 2 years
*Enrollment of additional participants beyond the original number of stated participants in the dose-expansion cohorts will be based on the determination of an acceptable safety profile and preliminary signs of efficacy.

4.2.2. Schedule of Activities

Table 9: Dose Escalation Assessments and Procedures (Casdatifan Monotherapy)

**Treatment Cycle (21-day cycle)**	**Screening (≤ 28 days)**	**Cycle 1**				**Cycle 2**		**Odd Cycles ≥ 3**	**Even Cycles ≥ 4**	**1-2 Years**	**2 Years Onwards[a]**	**TxDC**	**30-day SFU/ ETV**	**Long-term FU Call**
Cycle Day (visit window in days)		D1	D2	D8 (±1)	D15 (±1)	D1	D8 (±1)	D1 (±2)	D1 (±2)	Every 3 weeks (±3)	Every 6 weeks (±3)	(+3)	(±5)	Every 6 months (±5)
**Assessments/ Procedures**														
Informed consent	x													
Record medical history and demographics	x	x												
Prior anticancer therapies	x													
Confirmation of eligibility	x													
Physical exam[b]	x	x				x		x	x	x	x	x	x	
Height	x													
Weight	x	x				x		x	x	x	x	x	x	
Vital signs[c]	x	x	x	x	x	x	x	x	x	x	x	x	x	
6-minute walk test	x													
ECOG status	x	x				x		x	x	x	x	x	x	
Echocardiogram/MUGA	x	[redacted]												
Triplicate 12-lead ECG[d]	x	x			x	x		x[d]				x	x	
IMDC risk score	x													
Concomitant medications	x	x	x	x	x	x	x	x	x	x	x	x	x	
Adverse events	x	x	x	x	x	x	x	x	x	x	x	x	x	
**Safety Laboratory Assessments[e]**														
Hematology	x	x		x	x	x		x	x	x	x	x	x	
Chemistry	x	x		x	x	x		x	x	x	x	x	x	
Coagulation	x	x				x		x	x	x	x	x	x	
Urinalysis	x	x				x		x	x	x	x	x	x	
Serum testosterone[f]	x	x						x	x	x	x	x		
Serum or urine pregnancy test[g]	x	x				x		x	x	x[g]	x[g]	x	x	
Thyroid tests (TSH, FT3, FT4)	x													
Hepatitis B, C; HIV-1, HIV-2	x													

Table 9: Dose Escalation Assessments and Procedures (Casdatifan Monotherapy)

Treatment Cycle (21-day cycle)	Screening (≤ 28 days)	Cycle 1				Cycle 2		Odd Cycles ≥ 3	Even Cycles ≥ 4	1-2 Years	2 Years Onwards[a]	TxDC	30-day SFU/ ETV	Long-term FU Call
Cycle Day (visit window in days)		D1	D2	D8 (±1)	D15 (±1)	D1	D8 (±1)	D1 (±2)	D1 (±2)	Every 3 weeks (±3)	Every 6 weeks (±3)	(+3)	(±5)	Every 6 months (±5)
**Pharmacokinetic and Biomarker Assessments**														
Casdatifan blood PK[h]		x	x	x	x	x		x	x					
[redacted]		x												
	x					x						x[j]		
		x												
		x	x	x	x	x		x	x			x		
**Study Treatment**														
Study drug dispensation[m]		x				x		x	x	x[n]	x[n]			
Dosing compliance		x				x		x	x	x	x			
**Disease Response Assessment**														
Tumor imaging[o]	x	x[o]												
**Survival Follow-up**														
Survival status													x	x
Subsequent anticancer therapy (post-TxDC)												x	x	x

CT = computed tomography; CxDx = Cycle x Day x; [redacted]; Dx = Day x; ECG = electrocardiogram; ECOG = Eastern Cooperative Oncology Group; EPO = erythropoietin; ETV = Early Termination Visit; [redacted]; FT3 = free triiodothyronine; FT4 = free thyroxine; FU = follow-up; HIV = human immunodeficiency virus; hr = hour(s); IMDC = International Metastatic RCC Database Consortium; MUGA = multigated acquisition scan; PK = pharmacokinetic(s); PD = progressive disease RECIST = Response Evaluation Criteria in Solid Tumors; SFU = Safety Follow-Up Visit; TSH = thyroid stimulating hormone; TxDC = treatment discontinuation

[a] From year 2 of treatment onwards, patients are required to visit the clinic every 6 weeks. However, visit frequency may occur every 3 weeks at the investigator's discretion.

[b] Full physical examination at Screening, C1D1, TxDC, and 30-day SFU/ETV. All other visits, the physical examination can be symptom-directed at the investigator's discretion. See Section 8.2.1 for specific details of body systems to be included in examinations.

[c] At study visits for which multiple assessments are planned at the same timepoint, vital signs should be measured first, followed by ECGs, and then blood collection unless site-specific process dictate a specific site-based order. Vitals should be collected within a 60-minute window prior to dosing. See Section 8.2.2 for further details of specific vital assessments.

[d] Triplicate ECG assessments should be collected at screening, pre-dose, 1-hr, and 4-hr post-dose on C1D1 and C1D15; as well as pre-dose, 1-hr and 4-hr post-dose on C2D1 and C3D1 and the TxDC and 30-day SFU/ETV visits. ECG assessments should be collected within a 60-minute window prior to dosing and within a 15-minute window at post-dose timepoints. ECGs can be performed after C3D1 if medically indicated.

[e] Safety laboratory assessments only: C1D1 safety laboratory assessments may be performed up to 7 days prior to visit (see Section 8.2.4).

[f] For males (sex at birth) only: At Screening, C1D1, and at D1 of every 3rd cycle during the first year of treatment, every 9 weeks from year 1 to year 2 of treatment, and every 12 weeks from year 2 onwards, and the TxDC.

[g] At Screening, Day 1 of each Cycle during the first year of study treatment, TxDC Visit, and 30-day SFU/ETV, pregnancy testing will be performed on serum samples. For all other visits where pregnancy testing is required including those from year 1 of treatment onwards, a urine or serum pregnancy test is permissible. For pregnancy testing from year 1 of treatment onwards, a pregnancy test should be performed on all persons of childbearing potential at each 21-day cycle (±3 days) visit when the patient attends clinic to collect study drug.

[h] PK blood sample timepoints are C1D1 pre-dose, 1-, 2-, 4-, and 8-hr post-dose; C1D2 and C1D8 pre-dose; C1D15 pre-dose, 1-, 2-, 4-, and 8-hr post-dose; C2D1 pre-dose, 1-hr and 4-hr post-dose; C3D1 pre-dose, 1-hr and 4-hr post-dose; and pre-dose C4D1 and every cycle beyond through C18D1. Permitted windows for PK samples are detailed in Section 8.4. Urine PK samples will be collected at C1D1 pre-dose, and cumulative urine collection from 0 to 8 hr post-dose. See Section 8.4 for additional information.

[i] CCI [redacted]

[j] CCI [redacted]

[k] CCI [redacted]

[l] CCI [redacted]

[m] The first dose of study drug should be dispensed within 3 days of the site receiving a signed screening enrollment form.

[n] Study drug should be dispensed from the site every 3 weeks (± 3 days) after 1 year on treatment onwards. From year 2 onwards, study drug should be dispensed every 6 weeks. However, study drug dispensation and visit frequency may occur every 3 weeks at the investigator's discretion.

[o] Tumor imaging should occur at screening and again at C3D1 and every 6 weeks thereafter up to year 1 on treatment. From year 1 to year 2, imaging should be every 9 weeks, and every 12 weeks from year 2 onwards. Tumor imaging may be collected starting from 7 days before clinic visit day. Disease assessments will be performed until PD (regardless of whether or not the patient is still receiving study treatment), until patient comes off study, or starts alternative anticancer treatment. CT scans of the neck, bone, and/or extremities should be performed as clinically indicated in accordance with RECIST v1.1 (Appendix 5, Section 10.5). If the patient discontinues treatment for reasons other than PD, the patient will continue tumor assessments per the disease assessment schedule until PD or end of study (see Section 8.1 for further details).

Table 10: Dose Expansion Assessments and Procedures (Casdatifan Monotherapy)

Treatment Cycle (21-day cycle)	Screening (≤ 28 days)	Cycle 1				Cycle 2		Odd Cycles ≥ 3	Even Cycles ≥ 4	1-2 Years	2 Years Onwards[a]	TxDC	30-day SFU/ ETV	Long-term FU Call
Cycle Day (visit window in days)		D1	D2	D8 (±1)	D15 (±1)	D1 (±1)	D8 (±1)	D1 (±2)	D1 (±2)	Every 3 weeks (±3)	Every 6 weeks (±3)	(+3)	(±5)	Every 6 months (±5)
**Assessments/ Procedures**														
Informed consent	x													
Record medical history and demographics	x	x												
Prior anticancer therapies	x													
Confirmation of eligibility	x													
Physical exam[b]	x	x				x		x	x	x	x	x	x	
Height	x													
Weight	x	x				x		x	x	x	x	x	x	
Vital signs[c]	x	x	x	x	x	x	x	x	x	x	x	x	x	
6-minute walk test	x													
ECOG status	x	x				x		x	x	x	x	x	x	
Echocardiogram/MUGA	x	[redacted]												
Triplicate 12-lead ECG[d]	x	x			x	x		x[d]				x	x	
IMDC risk score	x													
Concomitant medications	x	x	x	x	x	x	x	x	x	x	x	x	x	
Adverse events	x	x	x	x	x	x	x	x	x	x	x	x	x	
**Safety Laboratory Assessments[e]**														
Hematology	x	x		x	x	x		x	x	x	x	x	x	
Chemistry	x	x		x	x	x		x	x	x	x	x	x	
Coagulation	x	x				x		x	x	x	x	x	x	
Urinalysis	x	x				x		x	x	x	x	x	x	
Serum testosterone[f]	x	x						x	x	x	x	x		
Serum or urine pregnancy test[g]	x	x				x		x	x	x[g]	x[g]	x	x	
Thyroid tests (TSH, FT3, FT4)	x													
Hepatitis B, C; HIV-1, HIV-2	x													

Table 10: Dose Expansion Assessments and Procedures (Casdatifan Monotherapy)

**Treatment Cycle (21-day cycle)**	**Screening (≤ 28 days)**	**Cycle 1**				**Cycle 2**		**Odd Cycles ≥ 3**	**Even Cycles ≥ 4**	**1-2 Years**	**2 Years Onwards[a]**	**TxDC**	**30-day SFU/ ETV**	**Long-term FU Call**
Cycle Day (visit window in days)		D1	D2	D8 (±1)	D15 (±1)	D1 (±1)	D8 (±1)	D1 (±2)	D1 (±2)	Every 3 weeks (±3)	Every 6 weeks (±3)	(+3)	(±5)	Every 6 months (±5)
**Pharmacokinetic and Biomarker Assessments**														
Casdatifan blood PK[h]		x	x	x	x	x		x	x					
[redacted]	x					x						x[j]		
		x												
		x		x	x	x		x	x			x		
		x						x				x		
**Study Treatment**														
Study drug dispensation[n]		x				x		x	x	x[o]	x[o]			
Dosing compliance		x				x		x	x	x	x			
**Disease Response Assessment**														
Tumor imaging[p]	x	x[p]												
**Survival Follow-up**														
Survival status													x	x
Subsequent anticancer therapy (post-TxDC)												x	x	x

ccRCC = clear cell renal cell carcinoma; CT = computed tomography; [redacted]; CxDx = Cycle x Day x; [redacted] Dx = Day x; ECG = electrocardiogram; ECOG = Eastern Cooperative Oncology Group; EPO = erythropoietin; ETV = Early Termination Visit; [redacted]; FT3 = free triiodothyronine; FT4 = free thyroxine; FU = follow-up; HIV = human immunodeficiency virus; hr = hour(s); IMDC = International Metastatic RCC Database Consortium; MUGA = multigated acquisition scan; PK = pharmacokinetic(s); PD = progressive disease; RECIST = Response Evaluation Criteria in Solid Tumors; SFU = Safety Follow-Up Visit; TSH = thyroid stimulating hormone; TxDC = treatment discontinuation

[a] From year 2 of treatment onwards, patients are required to visit the clinic every 6 weeks. However, visit frequency may occur every 3 weeks at the investigator's discretion.

[b] Full physical examination at Screening, C1D1, TxDC, and 30-day SFU/ETV. All other visits, the physical examination can be symptom-directed at the investigator's discretion. See Section 8.2.1 for specific details of body systems to be included in examinations.

[c] At study visits for which multiple assessments are planned at the same timepoint, vital signs should be measured first, followed by ECGs, and then blood collection unless site-specific process dictate a specific site-based order. Vitals should be collected within a 60-minute window prior to dosing. See Section 8.2.2 for further details of specific vital assessments.

[d] Triplicate ECG assessments should be collected at screening, pre-dose, 1-hr, and 4-hr post-dose on C1D1 and C1D15; as well as pre-dose, 1- and 4-hr post-dose on C2D1 and C3D1; and the TxDC and 30-day SFU/ETV visit. ECG assessments should be collected within a 60-minute window prior to dosing and within a 15-minute window at post-dose timepoints. ECGs may be performed after C3D1 if medically indicated.

[e] Safety laboratory assessments only: C1D1 safety laboratory assessments may be performed up to 7 days prior to visit (see Section 8.2.4).

[f] For males (sex at birth) only: At Screening, C1D1, and at D1 of every 3rd cycle during the first year of treatment, every 9 weeks from year 1 to year 2 of treatment, and every 12 weeks from year 2 onwards, and the TxDC.

[g] At Screening, Day 1 of each Cycle during the first year of study treatment, TxDC Visit, and 30-day SFU/ETV, pregnancy testing will be performed on serum samples. For all other visits where pregnancy testing is required including those from year 1 onwards, a urine or serum pregnancy test is permissible. For pregnancy testing from year 1 of treatment onwards, a pregnancy test should be performed on all persons of childbearing potential at each 21-day cycle (± 3 days) visit when the patient attends clinic to collect study drug.

[h] PK blood sample timepoints are C1D1 pre-dose, 1-, 2-, and 4-hr post-dose; C1D2 and C1D8 pre-dose; C1D15 pre-dose, 1-, 2-, and 4-hr post-dose; C2D1 pre-dose, 1-hr and 4-hr post-dose; C3D1 pre-dose, 1-hr and 4-hr post-dose; and pre-dose C4D1 and every cycle beyond through C18D1. Permitted windows for PK samples are detailed in Section 8.4.

[i] CCI [redacted]

[j] CCI [redacted]

[k] CCI [redacted]

[l] CCI [redacted]

[m] CCI [redacted]

[n] The first dose of study drug should be dispensed within 3 days of the site receiving a signed screening enrollment form.

[o] Study drug should be dispensed from the site every 3 weeks (±3 days) after 1 year on treatment onwards. From year 2 onwards, study drug should be dispensed every 6 weeks. However, study drug dispensation and visit frequency may occur every 3 weeks at the investigator's discretion.

[p] Tumor imaging should occur at screening and again at C3D1 and every 6 weeks thereafter up to year 1 on treatment. From 1 year to year 2, imaging should be every 9 weeks, and every 12 weeks from year 2 onwards. Tumor imaging may be collected from 7 days before clinic visit day. Disease assessments will be performed until PD (regardless of whether or not the patient is still receiving study treatment), until patient comes off study, or starts alternative anticancer treatment. CT scans of the neck, bone, and/or extremities should be performed as clinically indicated in accordance with RECIST v1.1 (Appendix 5, Section 10.5). If the patient discontinues treatment for reasons other than PD, the patient will continue tumor assessments per the disease assessment schedule until PD or end of study (see Section 8.1 for further details).

Table 11: Expansion Cohort 4 Assessments and Procedures

**Treatment Cycle (21-day cycle)**	**Screening (≤ 28 days)**	**Cycle 1**				**Cycle 2**		**Odd Cycles ≥ 3**	**Even Cycles ≥ 4**	**1-2 Years**	**2 Years Onwards[a]**	**TxDC**	**30-day SFU/ ETV**	**Long-term FU Call**
Cycle Day (visit window in days)		D1	D2	D8 (±1)	D15 (±1)	D1 (±1)	D8 (±1)	D1 (±2)	D1 (±2)	Every 3 weeks (±3)	Every 6 weeks (±3)	(+3)	(±5)	Every 6 months (±5)
**Assessments/ Procedures**														
Informed consent	x													
Record medical history and demographics	x	x												
Prior anticancer therapies	x													
Confirmation of eligibility	x													
Physical exam[b]	x	x				x		x	x	x	x	x	x	
Height	x													
Weight	x	x				x		x	x	x	x	x	x	
Vital signs[c]	x	x	x	x	x	x	x	x	x	x	x	x	x	
6-minute walk test	x													
ECOG status	x	x				x		x	x	x	x	x	x	
Echocardiogram/MUGA[d]	x	[redacted]												
Triplicate 12-lead ECG[e]	x	x			x	x		x[e]				x	x	
IMDC score	x													
Concomitant medications	x	x	x	x	x	x	x	x	x	x	x	x	x	
Adverse events	x	x	x	x	x	x	x	x	x	x	x	x	x	
**Safety Laboratory Assessments[f]**														
Hematology	x	x		x	x	x		x	x	x	x	x	x	
Chemistry	x	x		x	x	x		x	x	x	x	x	x	
Coagulation	x	x				x		x	x	x	x	x	x	
Urinalysis	x	x				x		x	x	x	x	x	x	
Serum testosterone[g]	x	x						x	x	x	x	x		
Serum or urine pregnancy test[h]	x	x				x		x	x	x[h]	x[h]	x	x	
[redacted]														
Thyroid tests (TSH, FT3, FT4)	x													
Hepatitis B, C; HIV-1, HIV-2	x													

Table 11: Expansion Cohort 4 Assessments and Procedures

**Treatment Cycle (21-day cycle)**	**Screening (≤ 28 days)**	**Cycle 1**				**Cycle 2**		**Odd Cycles ≥ 3**	**Even Cycles ≥ 4**	**1-2 Years**	**2 Years Onwards[a]**	**TxDC**	**30-day SFU/ ETV**	**Long-term FU Call**
Cycle Day (visit window in days)		D1	D2	D8 (±1)	D15 (±1)	D1 (±1)	D8 (±1)	D1 (±2)	D1 (±2)	Every 3 weeks (±3)	Every 6 weeks (±3)	(+3)	(±5)	Every 6 months (±5)
**Pharmacokinetic and Biomarker Assessments**														
Casdatifan blood PK[j]		x	x	x	x	x		x	x					
[redacted]		x	x	x	x	x		x	x					
	x					x						x[m]		
		x												
		x		x	x	x		x	x			x		
		x						x				x		
**Study Treatment**														
Casdatifan dispensation[q]		x				x		x	x	x[r]	x[r]			
Cabozantinib dispensation[q]		x				x		x	x	x	x			
Dosing compliance		x				x		x	x	x	x			
**Disease Response Assessment**														
Tumor imaging[s]	x	x[s]												
**Survival Follow-up**														
Survival status													x	x
Subsequent anticancer therapy (post-TxDC)												x	x	x

CT = computed tomography; [redacted]; CxDx = Cycle x Day x; [redacted]; Dx = Day x; ECG = electrocardiogram; ECOG = Eastern Cooperative Oncology Group; EPO = erythropoietin; ETV = Early Termination Visit; [redacted]; FT3 = free triiodothyronine; FT4 = free thyroxine; FU = follow-up; HIV = human immunodeficiency virus; hr = hour(s); IMDC = International Metastatic RCC Database Consortium; MUGA = multigated acquisition scan; PK = pharmacokinetic(s); PD = progressive disease; RECIST = Response Evaluation Criteria in Solid Tumors; SFU = Safety Follow-Up Visit; TSH = thyroid stimulating hormone; TxDC = treatment discontinuation

[a] From year 2 of treatment onwards, patients are required to visit the clinic every 6 weeks. However, visit frequency may occur every 3 weeks at the investigator's discretion.

[b] Full physical examination at Screening, C1D1, TxDC, and 30-day SFU/ETV. All other visits, the physical examination can be symptom-directed at the investigator's discretion. See Section 8.2.1 for specific details of body systems to be included in examinations.

[c] At study visits for which multiple assessments are planned at the same timepoint, vital signs should be measured first, followed by ECGs, and then blood collection unless site-specific process dictate a specific site-based order. Vitals should be collected within a 60-minute window prior to dosing. See Section 8.2.2 for further details of specific vital assessments.

[d] Echocardiogram/MUGA imaging should occur at Screening, [redacted].

[e] Triplicate ECG assessments should be collected at screening, pre-dose, 1-hr, and 4-hr post-dose on C1D1 and C1D15; as well as pre-dose, 1- and 4-hr post-dose on C2D1; pre-dose on C3D1; and the TxDC and 30-day SFU/ETV visit. ECG assessments should be collected within a 60-minute window prior to dosing and within a 15-minute window at post-dose timepoints. ECGs can be performed at any time after C3D1 where medically indicated.

[f] Safety laboratory assessments only: C1D1 safety laboratory assessments may be performed up to 7 days prior to visit (see Section 8.2.4).

[g] For males (sex at birth) only: At Screening, C1D1, and at D1 of every 3rd cycle during the first year of treatment, every 9 weeks from year 1 to year 2 of treatment, and every 12 weeks from year 2 onwards, and the TxDC.

[h] At Screening, Day 1 of each Cycle during the first year of study treatment, TxDC Visit, and 30-day SFU/ETV, pregnancy testing will be performed on serum samples. For all other visits where pregnancy testing is required including those from year 1 onwards, a urine or serum pregnancy test is permissible. For pregnancy testing from year 1 of treatment onwards, a pregnancy test should be performed on all persons of childbearing potential at each 21-day cycle (±3 days) visit when the patient attends clinic to collect study drug.

[i] [redacted]

[j] Casdatifan PK blood sample timepoints are C1D1 pre-dose, 1-, 2-, 4-, and 8-hr post-dose; C1D2 and C1D8 pre-dose; C1D15 pre-dose, 1-, 2- 4-, and 8-hr post-dose; C2D1 pre-dose, 1-hr and 4-hr post-dose; C3D1 pre-dose; and pre-dose C4D1 and every 2-cycles beyond through C18D1. The 8-hours post-dose samples at C1D1 and C1D15 will be only collected from patients who are enrolled in the safety run-in phase (see Section 4.1.3.2). Permitted windows for PK samples are detailed in Section 8.4.

[k] Cabozantinib PK blood sample timepoints are C1D1 pre-dose, 1-, 2-, 4-, and 8-hr post-dose; C1D2 and C1D8 pre-dose; C1D15 pre-dose, 1-, 2-, 4-, and 8-hr post-dose; C2D1 pre-dose, 1-hr and 4-hr post-dose; C3D1 pre-dose; and pre-dose C4D1 and every 2-cycles through C18D1. The 8-hours post-dose samples at C1D1 and C1D15 will be only collected from patients who are enrolled in the safety run-in phase (see Section 4.1.3.2). Permitted windows for PK samples are detailed in Section 8.4. See Table 34 for further details on the blood sampling schedule for PK analysis in Expansion Cohort 4.

[l] [redacted]

[m] [redacted]

[n] [redacted]

[o] [redacted]

[p] [redacted]

[q] The first dose of study drug should be dispensed within 3 days of the site receiving a signed screening enrollment form.

[r] Casdatifan should be dispensed from the site every 3 weeks (±3 days) after 1 year on treatment. From year 2 onwards, casdatifan should be dispensed every 6 weeks. However, casdatifan dispensation and visit frequency may occur every 3 weeks at the investigator's discretion. Cabozantinib should be dispensed per regionally approved prescribing information.

[s] Tumor imaging should occur at screening and again at C3D1 and every 6 weeks thereafter up to year 1 on treatment. From 1 year to year 2, imaging should be every 9 weeks, and every 12 weeks from year 2 onwards. Tumor imaging may be collected from 7 days before clinic visit day. Disease assessments will be

performed until PD (regardless of whether or not the patient is still receiving study treatment), until patient comes off study, or starts alternative anticancer treatment. CT scans of the neck, bone, and/or extremities should be performed as clinically indicated in accordance with RECIST v1.1 (Appendix 5, Section 10.5). If the patient discontinues treatment for reasons other than PD, the patient will continue tumor assessments per the disease assessment schedule until PD or end of study (see Section 8.1 for further details).

Table 12: Expansion Cohort 7 (ex-EU only) Assessments and Procedures

**Treatment Cycle (21-day cycle [Cycles 1-17]) (42-day cycle [Cycles ≥ 18])**	**Screening (≤ 28 days)**	**Cycle 1**				**Cycle 2**		**Odd Cycles ≥ 3**	**Even Cycles ≥ 4**	**1-2 Years**		**2 Years Onwards[a]**	**TxDC**	**30-day SFU/ ETV[b]**	**100-day SFU/ ETV[b]**	**Long-term FU Call**
Cycle Day (visit window in days)		D1	D2	D8 (±1)	D15 (±1)	D1 (±1)	D8 (±1)	D1 (±2)	D1 (±2)	D1 (±2)	D22 (±2)	Every 6 weeks (±3)	(+3)	(±5)	(±5)	Every 6 months (±5)
**Assessments/ Procedures**																
Informed consent	x															
Record medical history and demographics	x	x														
Prior anticancer therapies	x															
Confirmation of eligibility	x															
Physical exam[c]	x	x				x		x	x	x	x	x	x	x	x	
Height	x															
Weight	x	x				x		x	x	x	x	x	x	x	x	
Vital signs[d]	x	x	x	x	x	x	x	x	x	x	x	x	x	x	x	
6-minute walk test	x															
ECOG status	x	x				x		x	x	x	x	x	x	x	x	
Echocardiogram/MUGA	x	[redacted]														
Triplicate 12-lead ECG[e]	x	x			x	x		x[e]					x	x	x	
IMDC risk score	x															
Concomitant medications	x	x	x	x	x	x	x	x	x	x	x	x	x	x	x	
Adverse events	x	x	x	x	x	x	x	x	x	x	x	x	x	x	x	
**Safety Laboratory Assessments[f]**																
Hematology	x	x		x	x	x		x	x	x	x	x	x	x	x	
Chemistry	x	x		x	x	x		x	x	x	x	x	x	x	x	
Coagulation	x	x				x		x	x	x	x	x	x	x	x	
Urinalysis	x	x				x		x	x	x	x	x	x	x	x	
Serum testosterone[g]	x	x						x	x	x	x	x	x			
Serum or urine pregnancy test[h]	x	x				x		x	x	x[h]	x[h]	x[h]	x	x	x	
Thyroid tests (TSH, FT3, FT4)	x	x				x		x	x	x	x	x	x	x	x	

Table 12: Expansion Cohort 7 (ex-EU only) Assessments and Procedures

Treatment Cycle (21-day cycle [Cycles 1-17]) (42-day cycle [Cycles ≥ 18])	Screening (≤ 28 days)	Cycle 1				Cycle 2		Odd Cycles ≥ 3	Even Cycles ≥ 4	1-2 Years		2 Years Onwards[a]	TxDC	30-day SFU/ ETV[b]	100-day SFU/ ETV[b]	Long-term FU Call
Cycle Day (visit window in days)		D1	D2	D8 (±1)	D15 (±1)	D1 (±1)	D8 (±1)	D1 (±2)	D1 (±2)	D1 (±2)	D22 (±2)	Every 6 weeks (±3)	(+3)	(±5)	(±5)	Every 6 months (±5)
Hepatitis B, C; HIV-1, HIV-2	x															
**Pharmacokinetic and Biomarker Assessments**																
Casdatifan blood PK[i]		x	x		x	x		x	x							
Zimberelimab blood PK[j]		x				x		x	x	x[j]						
Zimberelimab blood ADA[j]		x				x		x	x	x[j]						
[redacted]	x					x							x[l]			
		x														
		x		x	x	x		x	x				x			
		x						x					x			
**Study Treatment**																
Casdatifan dispensation[p, q]		x				x		x	x	x	x	x				
Zimberelimab infusion[p, q]		x				x		x	x	x[q]						
Dosing compliance		x				x		x	x	x		x				
**Disease Response Assessment**																
Tumor imaging[r]	x										x[r]					
**Survival Follow-up**																
Survival status														x	x	x
Subsequent anticancer therapy (post-TxDC)													x	x	x	x

ADA = anti-drug antibody; CT = computed tomography; [redacted]; CxDx = Cycle x Day x; [redacted]; Dx = Day x; ECG = electrocardiogram; ECOG = Eastern Cooperative Oncology Group; EPO = erythropoietin; ETV = Early Termination Visit; [redacted]; FT3 = free triiodothyronine; FT4 = free thyroxine; FU = follow-up; HIV = human immunodeficiency virus; hr = hour(s); IMDC = International Metastatic RCC Database Consortium; IV = intravenous; MUGA = multigated acquisition scan; PK = pharmacokinetic(s); PD = progressive disease; Q6W = every 6 weeks; RECIST = Response Evaluation Criteria in Solid Tumors; SFU = Safety Follow-Up Visit; TSH = thyroid stimulating hormone; TxDC = treatment discontinuation

[a] From year 2 of treatment onwards, patients are required to visit the clinic every 6 weeks. However, visit frequency may occur every 3 weeks at the investigator’s discretion.

[b] If the patient had discontinued zimberelimab > 100 days prior to the discontinuation of casdatifan, only one SFU visit should occur 30 days after the last dose of casdatifan. Otherwise, two SFU visits should occur at 30 days after the discontinuation of casdatifan and at 100 days after the last dose of zimberelimab.

[c] Full physical examination at Screening, C1D1, TxDC, and 30-day (and 100-day post-IO) SFU/ETV. All other visits, the physical examination can be symptom-directed at the investigator’s discretion. See Section 8.2.1 for specific details of body systems to be included in examinations.

[d] At study visits for which multiple assessments are planned at the same timepoint, vital signs should be measured first, followed by ECGs, and then blood collection unless site-specific process dictate a specific site-based order. Vitals should be collected within a 60-minute window prior to dosing. See Section 8.2.2 for further details of specific vital assessments.

[e] Triplicate ECG assessments should be collected at screening, pre-dose, 1-hr, and 4-hr post-dose on C1D1 and C1D15; as well as pre-dose, 1- and 4-hr post-dose on C2D1; pre-dose on C3D1; and the TxDC and 30-day (and 100-day post-IO) SFU/ETV visit. ECG assessments should be collected within a 60-minute window prior to dosing and within a 15-minute window at post-dose timepoints. ECGs can be performed at any time after C3D1 where medically indicated.

[f] Safety laboratory assessments only: C1D1 safety laboratory assessments may be performed up to 7 days prior to visit (see Section 8.2.4).

[g] For males (sex at birth) only: At Screening, C1D1, and at D1 of every 3rd cycle during the first year of treatment, every 9 weeks from year 1 to year 2 of treatment, and every 12 weeks from year 2 onwards, and the TxDC.

[h] At Screening, Day 1 of each Cycle during the first year of study treatment, TxDC Visit, and 30-day (and 100-day post-IO) SFU/ETV, pregnancy testing will be performed on serum samples. For all other visits where pregnancy testing is required including those from year 1 onwards, a urine or serum pregnancy test is permissible. For pregnancy testing from year 1 of treatment onwards, a pregnancy test should be performed on all persons of childbearing potential every 21 days (±3 days).

[i] Casdatifan PK blood sample timepoints are C1D1 pre-dose, 1- and 4-hr post-dose; C1D2 pre-dose; C1D15 pre-dose, 1- and 4-hr post-dose; C2D1 pre-dose, 1-and 4-hr post-dose; C3D1 pre-dose; and C4D1 pre-dose and every 2-cycles through C18D1. Permitted windows for PK samples are detailed in Section 8.4.

[j] Zimberelimab PK blood sample timepoints are C1D1 pre-dose; C2D1 pre-dose; C3D1 pre-dose; pre-dose C4D1 and every 2 cycles through C18D1; and pre-dose C19D1 and every cycle through C22D1. Zimberelimab ADA blood sample timepoints are C1D1 pre-dose; C2D1 pre-dose; C3D1 predose; C4D1 pre-dose and every 2 cycles through C18D1; and pre-dose C19D1 and every cycle through C22D1. Permitted windows for PK/ADA samples are detailed in Section 8.4. See Table 35 for further details on the blood sampling schedule for PK analysis in Expansion Cohort 7.

[k] [redacted]

[l] [redacted]

[m] [redacted]

[n] CCI [redacted]

[o] CCI [redacted]

[p] The first dose of study drug should be dispensed within 3 days of the site receiving a signed screening enrollment form.

[q] Casdatifan should be dispensed from the site every 3 weeks (±3 days) after 1 year on treatment onwards. From year 2 onwards, casdatifan should be dispensed every 6 weeks. However, casdatifan dispensation and visit frequency may occur every 3 weeks at the investigator's discretion. On clinic visit dates, patients will hold casdatifan administration until directed by study site personnel. Following 1 year on study treatment (starting on Cycle 18) patients will move to zimberelimab at 720 mg IV infusion Q6W. Casdatifan should be administered 30 to 60 minutes prior to the start of IV infusion of zimberelimab. Patients will be administered zimberelimab for a maximum of 2 years following C1D1.

[r] Tumor imaging should occur at screening and again at C3D1 and every 6 weeks thereafter up to year 1 on treatment. From 1 year to year 2, imaging should be every 9 weeks, and every 12 weeks from year 2 onwards. Tumor imaging may be collected from 7 days before clinic visit day. Disease assessments will be performed until PD (regardless of whether or not the patient is still receiving study treatment), until patient comes off study, or starts alternative anticancer treatment. CT scans of the neck, bone, and/or extremities should be performed as clinically indicated in accordance with RECIST v1.1 (Appendix 5, Section 10.5). If the patient discontinues treatment for reasons other than PD, the patient will continue tumor assessments per the disease assessment schedule until PD or end of study (see Section 8.1 for further details).

Table 13: Expansion Cohort 9 and Cohort 10 Assessments and Procedures

**Treatment Cycle (21-day cycle [Cycles 1-6]) (42-day cycle [Cycles ≥ 7])**	**Screening (≤ 28 days)**	**Cycle 1**				**Cycle 2**		**Cycles 3 and 4**	**Cycles 5 and 6**	**Cycles ≥ 7 up to 2 years**		**2 Years Onwards[a]**	**TxDC**	**30-day SFU/ ETV[b]**	**100-day SFU/ ETV[b]**	**Long-term FU Call**
Cycle Day (visit window in days)		D1	D2	D8 (±1)	D15 (±1)	D1 (±1)	D8 (±1)	D1 (±2)	D1 (±2)	D1 (±2)	D22 (±2)	Every 6 weeks (±3)	(+3)	(±5)	(±5)	Every 6 months (±5)
**Assessments/ Procedures**																
Informed consent	x															
Record medical history and demographics	x	x														
Prior anticancer therapies	x															
Confirmation of eligibility	x															
Physical exam[c]	x	x				x		x	x	x	x	x	x	x	x	
Height	x															
Weight	x	x				x		x	x	x	x	x	x	x	x	
Vital signs[d]	x	x	x	x	x	x	x	x	x	x	x	x	x	x	x	
6-minute walk test	x															
ECOG status	x	x				x		x	x	x	x	x	x	x	x	
Echocardiogram/MUGA	x	CCI [redacted]														
Triplicate 12-lead ECG[e] (Expansion Cohort 9)	x	x			x	x		x[e]					x	x	x	
Triplicate 12-lead ECG[e] (Expansion Cohort 10)	x								x[e]	x			x	x	x	
IMDC risk score	x															
Concomitant medications	x	x	x	x	x	x	x	x	x	x	x	x	x	x	x	
Adverse events	x	x	x	x	x	x	x	x	x	x	x	x	x	x	x	
**Safety Laboratory Assessments[f]**																
Hematology	x	x		x	x	x	x	x	x	x	x	x	x	x	x	
Chemistry	x	x		x	x	x	x	x	x	x	x	x	x	x	x	
Coagulation	x	x				x		x	x	x	x	x	x	x	x	
Urinalysis	x	x				x		x	x	x	x	x	x	x	x	
Serum testosterone[g]	x	x						x	x	x	x	x	x			

Table 13: Expansion Cohort 9 and Cohort 10 Assessments and Procedures

**Treatment Cycle (21-day cycle [Cycles 1-6]) (42-day cycle [Cycles ≥ 7])**	**Screening (≤ 28 days)**	**Cycle 1**				**Cycle 2**		**Cycles 3 and 4**	**Cycles 5 and 6**	**Cycles ≥ 7 up to 2 years**		**2 Years Onwards[a]**	**TxDC**	**30-day SFU/ ETV[b]**	**100-day SFU/ ETV[b]**	**Long-term FU Call**
Cycle Day (visit window in days)		D1	D2	D8 (±1)	D15 (±1)	D1 (±1)	D8 (±1)	D1 (±2)	D1 (±2)	D1 (±2)	D22 (±2)	Every 6 weeks (±3)	(+3)	(±5)	(±5)	Every 6 months (±5)
Serum or urine pregnancy test[h]	x	x				x		x	x	x[h]	x[h]	x[h]	x	x	x	
Thyroid tests (TSH, FT3, FT4)	x	x				x		x	x	x	x	x	x	x	x	
Hepatitis B, C; HIV-1, HIV-2	x															
**Pharmacokinetic and Biomarker Assessments**																
Casdatifan blood PK[i] (Expansion Cohort 9)		x	x		x	x		x	x	x[i]						
Casdatifan blood PK[i] (Expansion Cohort 10)									x	x[i]						
Zimberelimab blood PK[j]		x				x		x	x	x[j]						
Zimberelimab blood ADA[j]		x				x		x	x	x[j]						
CCI [redacted]	x								x[k]				x[l]			
[redacted]																
[redacted]		x														
[redacted]		x				x			x	x[n]						
[redacted]		x						x		x			x			
**Study Treatment**																
Casdatifan dispensation[p, q] (Expansion Cohort 9)		x				x		x	x	x	x	x				
Casdatifan dispensation[p, r] (Expansion Cohort 10)									x	x	x	x				
Zimberelimab infusion[p, s]		x				x		x	x	x						
Ipilimumab infusion[p, s]		x				x		x								
Dosing compliance		x				x		x	x	x		x				

Table 13: Expansion Cohort 9 and Cohort 10 Assessments and Procedures

**Treatment Cycle (21-day cycle [Cycles 1-6]) (42-day cycle [Cycles ≥ 7])**	**Screening (≤ 28 days)**	**Cycle 1**				**Cycle 2**		**Cycles 3 and 4**	**Cycles 5 and 6**	**Cycles ≥ 7 up to 2 years**		**2 Years Onwards[a]**	**TxDC**	**30-day SFU/ ETV[b]**	**100-day SFU/ ETV[b]**	**Long-term FU Call**
Cycle Day (visit window in days)		D1	D2	D8 (±1)	D15 (±1)	D1 (±1)	D8 (±1)	D1 (±2)	D1 (±2)	D1 (±2)	D22 (±2)	Every 6 weeks (±3)	(+3)	(±5)	(±5)	Every 6 months (±5)
**Disease Response Assessment**																
Tumor imaging[t]	x	x[t]														
**Survival Follow-up**																
Survival status														x	x	x
Subsequent anticancer therapy (post-TxDC)													x	x	x	x

ADA = anti-drug antibody; CT = computed tomography; [redacted] CxDx = Cycle x Day x; [redacted] Dx = Day x; ECG = electrocardiogram; ECOG = Eastern Cooperative Oncology Group; EPO = erythropoietin; ETV = Early Termination Visit; [redacted]; FT3 = free triiodothyronine; FT4 = free thyroxine; FU = follow-up; HIV = human immunodeficiency virus; hr = hour(s); IMDC = International Metastatic RCC Database Consortium; IV = intravenous; MUGA = multigated acquisition scan; PK = pharmacokinetic(s); PD = progressive disease; RECIST = Response Evaluation Criteria in Solid Tumors; SFU = Safety Follow-Up Visit; TSH = thyroid stimulating hormone; TxDC = treatment discontinuation

[a] From year 2 of treatment onwards, patients are required to visit the clinic every 6 weeks. However, visit frequency may occur every 3 weeks at the investigator's discretion.

[b] If the patient had discontinued zimberelimab and/or ipilimumab > 100 days prior to the discontinuation of casdatifan, only one SFU visit should occur at 30 days after the last dose of casdatifan. Otherwise, two SFU visits should occur at 30 days after the discontinuation of casdatifan and at 100 days after the last dose of zimberelimab and/or ipilimumab.

[c] Full physical examination at Screening, C1D1, TxDC, and 30-day (and 100-day post-IO) SFU/ETV. All other visits, the physical examination can be symptom-directed at the investigator's discretion. See Section 8.2.1 for specific details of body systems to be included in examinations.

[d] At study visits for which multiple assessments are planned at the same timepoint, vital signs should be measured first, followed by ECGs, and then blood collection unless site-specific process dictate a specific site-based order. Vitals should be collected within a 60-minute window prior to dosing. See Section 8.2.2 for further details of specific vital assessments.

[e] Triplicate ECG assessments should be collected at screening, pre-dose, 1-hr, and 4-hr post-dose on C1D1 and C1D15; as well as pre-dose, 1- and 4-hr post-dose on C2D1; pre-dose on C3D1; and the TxDC and 30-day (and 100-day post-IO) SFU/ETV visit. ECG assessments should be collected within a 60-minute window prior to dosing and within a 15-minute window at post-dose timepoints. ECGs can be performed at any time after C3D1 where medically indicated. For Expansion Cohort 10, ECGs start on C5D1 and skip C6.

[f] Safety laboratory assessments only: C1D1 safety laboratory assessments may be performed up to 7 days prior to visit (see Section 8.2.4).

[g] For males (sex at birth) only: At Screening, C1D1, and at D1 of every 3rd cycle during the first year of treatment, every 9 weeks from year 1 to year 2 of treatment, and every 12 weeks from year 2 onwards, and the TxDC.

[h] At Screening, Day 1 of each Cycle during the first year of study treatment, TxDC Visit, and 30-day (and 100-day post-IO) SFU/ETV, pregnancy testing will be performed on serum samples. For all other visits where pregnancy testing is required including those from Cycle 7 onwards, a urine or serum pregnancy test is permissible. For pregnancy testing from Cycle 7 of treatment onwards, a pregnancy test should be performed on all persons of childbearing potential every 21 days (±3 days).

[i] Casdatifan PK blood sample timepoints in Expansion Cohort 9 are C1D1 pre-dose, 1- and 4-hr post-dose; C1D2 pre-dose; C1D15 pre-dose, 1- and 4-hr postdose; C2D1 pre-dose, 1-and 4-hr post-dose; and pre-dose C3D1 and every cycle through C13D1 (see Table 36). Casdatifan PK blood sample timepoints in Expansion Cohort 10 are C5D1 pre-dose, 1- and 4-hr postdose; C6D1 pre-dose, 1- and 4-hr postdose; and C7D1 pre-dose and every cycle through C13D1 (see Table 37). Permitted windows for PK samples are detailed in Section 8.4.

[j] Zimberelimab PK blood sample timepoints are pre-dose C1D1 and every cycle through C13D1. Zimberelimab ADA blood sample timepoints are pre-dose C1D1 and every cycle through C13D1. Permitted windows for PK/ADA samples are detailed in Section 8.4. See Table 36 and Table 37 for further details on the blood sampling schedule for PK analysis in Expansion Cohorts 9 and 10, respectively.

[k] CCI [redacted]

[l] CCI [redacted]

[m] CCI [redacted]

[n] CCI [redacted]

[o] CCI [redacted]

[p] The first dose of study drug should be dispensed within 3 days of the site receiving a signed screening enrollment form.

[q] For Cohort 9, casdatifan should be dispensed from the site every 3 weeks (±3 days) from Cycle 1 until 2-year mark, while on treatment. From Year 2 onwards, casdatifan should be dispensed every 6 weeks. However, casdatifan dispensation and visit frequency may occur every 3 weeks at the investigator's discretion. On clinic visit dates, patients will hold casdatifan administration until directed by study site personnel. Casdatifan should be administered 30 to 60 minutes prior to the start of IV infusion of zimberelimab.

[r] For Cohort 10, casdatifan should be dispensed from the site every 3 weeks (±3 days) from the first cycle (eg, Cycle 5) following discontinuation of ipilimumab until 2-year mark, while on treatment. From Year 2 onwards, casdatifan should be dispensed every 6 weeks. However, casdatifan dispensation and visit frequency may occur every 3 weeks at the investigator's discretion. On clinic visit dates, patients will hold casdatifan administration until directed by study site personnel. Casdatifan should be administered 30 to 60 minutes prior to the start of IV infusion of zimberelimab.

[s] After the end of the zimberelimab infusion, ipilimumab will be administered per the prescribing information's dosing window for a total of 4 cycles. Patients will be administered zimberelimab for a maximum of 2 years following C1D1. Weight-based dosing for zimberelimab and ipilimumab may be calculated using weight at C1D1. If the patient's weight changes by ≥ 10%, the dose should be recalculated using the patient's new weight.

[T] Tumor imaging should occur at screening and again at C3D1 and every 6 weeks thereafter up to year 1 on treatment. From 1 year to year 2, imaging should be every 9 weeks, and every 12 weeks from year 2 onwards. Tumor imaging may be collected from 7 days before clinic visit day. Disease assessments will be performed until PD (regardless of whether or not the patient is still receiving study treatment), until patient comes off study, or starts alternative anticancer treatment. CT scans of the neck, bone, and/or extremities should be performed as clinically indicated in accordance with RECIST v1.1 (Appendix 5, Section 10.5). If the patient discontinues treatment for reasons other than PD, the patient will continue tumor assessments per the disease assessment schedule until PD or end of study (see Section 8.1 for further details).

5. STUDY POPULATION

Prospective approval of protocol deviations to recruitment and enrollment criteria, also known as protocol waivers or exemptions, is not permitted. The investigator must verify that the patient satisfies all eligibility criteria, and document approval to enroll the patient, before the patient can be enrolled. Every effort will be made to enroll a diverse patient population.

5.1. Inclusion Criteria

1. Capable of giving signed informed consent, which includes compliance with the requirements and restrictions listed in the ICF and in this protocol.
2. Male or female patients ≥ 18 years of age (or at least the age that is regionally approved age of consent for participation in investigational clinical studies) at the time of signing the ICF.
3. Disease-specific criteria for dose escalation:
    a. Patients may have any pathologically confirmed solid tumor type where no other treatment options are available, and
    b. Creatinine clearance ≥ 40 mL/min as determined by Cockcroft-Gault equation (Cockcroft and Gault 1976) or other acceptable method.
4. Disease-specific criteria for dose expansion:
    a. Histologically confirmed ccRCC and,
    b. **Casdatifan expansion cohorts**:
        – **Expansion Cohorts 1, 2, 3, and 5**: Patients must have received prior treatment in the locally advanced/unresectable or metastatic setting with an anti-PD-1/PD-L1 therapy and a VEGFR-targeting TKI (either individually or in combination).
        – **Expansion Cohort 6 (ex-EU only):** Patients who did not receive prior systemic treatment in the locally advanced/unresectable or metastatic setting are eligible. Patients who received adjuvant anti-PD-1/PD-L1 therapy are permitted (must have recurred on or within 12 months of the last dose of therapy). Only patients with IMDC risk scores of 0 are permitted.
        – **Expansion Cohort 8:** Patients should have received 1 systemic line in the locally advanced/unresectable or metastatic setting that includes anti-PD-1/PD-L1 therapy (as monotherapy or in combination with another immunotherapy). Patients should have never received a VEGF-TKI or a HIF-2α inhibitor. Patients who received anti-PD-1/PD-L1 therapy in the adjuvant setting (must have recurred on or within 12 months of the last dose of therapy) are also eligible. Treatment in the adjuvant setting is considered as a prior line of therapy.
        – **Expansion Cohort 4 (casdatifan + cabozantinib combination):** Patients must have received anti-PD-1/PD(L)-1 (as monotherapy or in combination with another immunotherapy; VEGFR-TKI or bevacizumab). Patients who received anti-PD-1 in the adjuvant setting must have had PD on or within 12 months of the last dose of therapy. Patients must have received at least 2 doses of anti-PD-1/PD(L)-1 therapy. No more than 2 prior lines of therapy; treatment in the adjuvant setting is considered as prior line of therapy. No prior cabozantinib therapy is permitted in Expansion Cohort 4.

c. **<u>Expansion Cohort 7 (ex-EU only) (casdatifan + zimberelimab combination), Expansion Cohort 9 and Expansion Cohort 10 (zimberelimab + ipilimumab +/- casdatifan followed by zimberelimab + casdatifan)</u>:** Patients must not have received systemic therapy for locally advanced/unresectable or metastatic disease. All IMDC risk scores (ie, favorable, intermediate, and poor) are permitted. Patients who received anti-PD-1/PD-L1 therapy in the adjuvant setting are eligible if recurrence occurs more than 12 months after the last dose.
d. No prior treatment with a HIF-2α-targeting therapy.
e. Creatinine clearance ≥ 40 mL/min as determined by Cockcroft-Gault equation (Cockcroft and Gault 1976) or other acceptable method.

5. Must have at least 1 measurable lesion per RECIST guidance (Version 1.1; see Appendix 5 in Section 10.5).

6. Eastern Cooperative Oncology Group (ECOG) performance status score of ≤ 1 (see Appendix 4 in Section 10.4).

7. Patients with a history of hepatitis B, hepatitis C, or human immunodeficiency virus (HIV) must:

   a. For hepatitis B, demonstrate resolution of infection or be on adequate suppressive antiviral therapy and not at risk for hepatic decompensation.

   b. For hepatitis C, demonstrate resolution of infection or be on adequate suppressive antiviral therapy and not at risk for hepatic decompensation.

   c. For HIV, demonstrate CD4 T-cell count of at least 350 cells/microliter and be on adequate suppressive antiviral therapy.

8. Adequate organ and marrow function including:

   a. Absolute neutrophil count ≥ 1.5 × 10^9/L
   b. Platelet count ≥ 100 × 10^9/L
   c. Hemoglobin ≥ 10.0 g/dL (or 6.2 mmol/L)
   d. Aspartate transaminase (AST) ≤ 2.5 × upper limit of normal (ULN) without hepatic metastasis and ≤ 5 × ULN with hepatic metastasis
   e. Alanine aminotransferase (ALT) ≤ 2.5 × ULN without hepatic metastasis and ≤ 5 × ULN with hepatic metastasis
   f. Bilirubin ≤ 1.5 × ULN (except patients with Gilbert syndrome who must have total bilirubin < 3.0 mg/dL).
   g. Activated partial thromboplastin time (aPTT) ≤ 1.5 × ULN

9. No exercise-induced desaturation on a 6-minute walk test, defined as a blood oxygen saturation by pulse oximetry ≤ 88%.

10. No active pneumonitis at screening.

11. No medical history of severe chronic obstructive pulmonary disease (COPD).

12. Screening echocardiogram or multigated acquisition scan (MUGA) scan must demonstrate a left ventricular ejection fraction (LVEF) greater than the institutional lower limit of normal (LLN). See specific requirements for Expansion Cohort 4 in Section 4.1.3.2.

Sex and Contraceptive/Barrier Requirements

13. Contraceptive use by males and females should be consistent with local regulations regarding the methods of contraception for those participating in clinical studies.
    a. Male patients must use highly effective contraceptive methods during treatment and for 7 days post last dose of casdatifan, for 4 months after the final dose of cabozantinib, and for 120 days after the last dose of zimberelimab (whichever is later); as well as refrain from donating sperm during treatment and for 7 days post last dose of casdatifan, for 4 months after the final dose of cabozantinib, and for 120 days after the last dose of zimberelimab (whichever is later) (see Section 10.2 for additional information).
    b. Given the potential effect of some hormonal contraceptives, females of childbearing potential who are using hormonal contraceptives must be willing to use, in addition, a second effective non-hormonal method of contraception (eg, male condom, diaphragm with spermicide).
    c. For Expansion Cohort 4 only: Women of childbearing potential must agree to remain abstinent (refrain from intercourse of childbearing potential) or use contraception and agree to refrain from donating eggs, as defined below:
        - Women must remain abstinent or use contraceptive methods with a failure rate of < 1% per year during the treatment period, and for 7 days post last dose of casdatifan and for 4 months after the final dose of cabozantinib (whichever is later). Women must refrain from donating eggs during this same period.
14. A female patient is eligible to participate if she is not pregnant (Section 10.2), not breastfeeding, not planning to become pregnant, and at least 1 of the following conditions applies:
    a. Not a woman of childbearing potential (WOCBP) as defined in Appendix 2 (Section 10.2)

    OR

    b. A WOCBP who agrees to follow the contraceptive guidance in Appendix 2 (Section 10.2) during the treatment period and for 7 days post last dose of casdatifan, for 4 months after the final dose of cabozantinib, and for 120 days after the last dose of zimberelimab (whichever is later); as well as agrees to not donate ova during the treatment period and for 7 days post last dose of casdatifan, for 4 months after the final dose of cabozantinib, and for 120 days after the last dose of zimberelimab (whichever is later).

Other Inclusions

15. Willing and able to comply with the requirements and restrictions in this protocol.
16. If a patient has CNS metastases the patient must meet the following criteria:
    a. Patients with previously treated brain metastases may participate provided they are clinically stable and without steroid treatment for at least 14 days prior to the first dose of study treatment. Patients may be on a dose of 10 mg QD prednisone (or

equivalent) or less for at least 2 weeks prior to the first dose of study treatment. Patients with carcinomatous meningitis are excluded.

5.2. Exclusion Criteria

Patients are excluded from the study if any of the following criteria below and any of the relevant disease-specific criteria for dose escalation or expansion apply:

1. Use of any live vaccines against infectious diseases (eg, influenza, varicella) within 4 weeks (28 days) of initiation of investigational product.
2. Has any other clinically significant cardiac, respiratory, or other medical or psychiatric condition that might interfere with a patient's participation in the clinical study or make the administration of investigational product hazardous (eg, interstitial lung disease, active infections requiring antibiotics, recent hospitalizations with unresolved symptoms, poorly controlled hypertension) or obscure the interpretation of toxicity determination or AEs.
3. History of trauma or major surgery within 28 days prior to the first dose of investigational product. (Note that placement of central venous access catheter [eg, port or similar] is not considered a major surgical procedure).
4. For all dose-expansion cohorts: prior treatment with an HIF-2α inhibitor.
5. Has known psychiatric or substance abuse disorders that would interfere with cooperation with the requirements of the trial.
6. Positive serum pregnancy test at screening and prior to dosing on C1D1 (WOCBP only).
7. Is pregnant or breastfeeding, or is trying to conceive or father children within the projected duration of the study, starting with the pre-screening or screening visit through 7 days after the last dose of casdatifan, for 4 months after the final dose of cabozantinib, and for 120 days after the last dose of zimberelimab (whichever is later).
8. Other prior malignancy active within the previous year except for locally curable cancers that have been apparently cured, such as basal or squamous cell skin cancer, superficial bladder cancer, or carcinoma in situ of the cervix, breast, or prostate cancer. Also, indolent malignancies, including but not limited to early-stage chronic lymphocytic leukemia and follicular lymphoma, that don't require anti-cancer treatment, could be allowed after discussion with the medical monitor.
9. Prior chemotherapy targeted small-molecule therapy, immunotherapy (tumor vaccine, cytokine, or growth factor given to control the cancer), or biologic agents, or use of other investigational drugs (drugs not marketed for any indication) within 28 days or 5 half-lives (whichever is shorter) before investigational product administration. Locoregional therapy (immunotherapy, chemotherapy, or radioembolization) must have been conducted at least 2 weeks before the first dose of study treatment.
10. QTc $\geq$ 480 msec using Fridericia's correction (QTcF) (based on an average of triplicate recordings).
11. Known hypersensitivity to any excipient contained in the investigational product formulations.

12. Inability to swallow study treatment.
13. Malabsorption condition that would alter the absorption of orally administered medications.
14. Has had any major cardiovascular event within 6 months prior to study drug administration including but not limited to myocardial infarction, unstable angina, cerebrovascular accident, transient ischemic event, pulmonary embolism, clinically significant ventricular arrhythmias (eg, sustained ventricular tachycardia, ventricular fibrillation, torsades de pointes) or New York Heart Association Class III or IV heart failure.

Expansion Cohort 4

Patients in Expansion Cohort 4 will be excluded from this study if they meet any of the following criteria in addition to the criteria outlined above.

15. Any prior treatment with cabozantinib.
16. Concomitant treatment with therapeutic doses of anticoagulants such as heparin, thrombin or Factor Xa inhibitors, or antiplatelet agents (eg, clopidogrel).

    Note: Low-dose aspirin (< 81 mg/daily) and prophylactic low-molecular-weight heparin (LMWH) are permitted. Anticoagulation with therapeutic doses of LMWH is allowed in patients without radiographic evidence of uncontrolled brain metastases, who are on a stable dose of LMWH for at least 2 weeks before administration of the first dose of study treatment, and who have had no complications from a thromboembolic event or the anticoagulation regimen.
17. Has uncontrolled or poorly controlled hypertension, as defined by a sustained blood pressure > 140/90 mm Hg while on more than 3 antihypertensives. Furthermore, patients must not have had any changes in antihypertensives at least 2 weeks before administration of the first dose of study treatment.
18. Stroke (including transient ischemic attack [TIA]), myocardial infarction, or other ischemic event or thromboembolic event (eg, deep venous thrombosis, pulmonary embolism [DVT/PE]) within 6 months before administration of the first dose of study treatment. Patients with a diagnosis of DVT within 6 months are allowed if stable and treated with LMWH for at least 3 weeks before administration of the first dose of study treatment.
19. Has gastrointestinal (GI) disorders including those associated with a high risk of perforation or fistula formation:
    a. Tumors invading the GI-tract, active peptic ulcer disease, inflammatory bowel disease, diverticulitis, cholecystitis, symptomatic cholangitis or appendicitis, acute pancreatitis or acute obstruction of the pancreatic or biliary duct, or gastric outlet obstruction.
    b. Abdominal fistula, GI perforation, bowel obstruction, or intra-abdominal abscess within 6 months before administration of the first dose of study treatment.

Note: Complete healing of an intra-abdominal abscess must be confirmed before administration of the first dose of study treatment.

20. Has clinically significant hematuria, hematemesis or hemoptysis of > 0.5 teaspoon (2.5 mL) of red blood, or other history of significant bleeding (eg, pulmonary hemorrhage) within 3 months before administration of the first dose of study treatment.
21. Has tumor invading or encasing any major blood vessels.
22. Has other clinically significant disorders such as:
    a. Serious non-healing wound/ulcer/bone fracture.
    b. Moderate to severe hepatic impairment (Child-Pugh B or C).
        - The initial 6 patients enrolled into the study cannot have mild hepatic impairment (Child-Pugh A) as they must be eligible to receive cabozantinib at the full dose of 60 mg/day.
    c. Requirement for hemodialysis or peritoneal dialysis.
    d. History of solid organ transplantation:
        - Has had major surgery (eg, GI surgery, removal or biopsy of brain metastasis) within 6 weeks, prior laparoscopic nephrectomy within 4 weeks, or minor surgeries (eg, simple excision, tooth extraction) within 10 days before the first study drug administration. Complete wound healing from major surgery must have occurred before the first study drug administration.

Expansion Cohort 7 (ex-EU only), Expansion Cohort 9, and Expansion Cohort 10

Patients in Expansion Cohorts 7, 9, and 10 will be excluded from this study if they meet any of the following criteria in addition to the criteria outlined above.

23. Has any active autoimmune disease, uncontrolled endocrinopathy, or a documented history of autoimmune disease or syndrome that required systemic treatment in the past 3 years.

    Note: Patients with type I diabetes mellitus, hypothyroidism only requiring hormone replacement, skin disorders (such as vitiligo, psoriasis, or alopecia) not requiring systemic treatment, or conditions not expected to recur in the absence of an external trigger are permitted to enroll.

24. Treatment with systemic immunosuppressive medication (including but not limited to corticosteroids, cyclophosphamide, azathioprine, methotrexate, thalidomide, and anti-TNFα agents) administered at > 10 mg/day prednisone or equivalent within 2 weeks prior to initiation of study treatment, or anticipation of need for systemic immunosuppressant medication during study treatment. Corticosteroids given as short course premedication are acceptable.

    Note: inhaled, intranasal, intra-articular, or topical steroids are permitted. Adrenal replacement steroid doses > 10 mg daily prednisone equivalent are permitted in the absence of active autoimmune disease. Transient short-term use of systemic corticosteroids for allergic conditions (eg, contrast allergy) is also allowed.

25. If patients received prior adjuvant PD-1 treatment, they cannot have discontinued treatment for toxicities.
26. If patients received prior adjuvant CTLA-4 treatment.
27. Has uncontrolled or poorly controlled hypertension, as defined by a sustained blood pressure > 140/90 mm Hg while on more than 3 antihypertensives. Furthermore, patients must not have had any changes in antihypertensives at least 2 weeks before administration of the first dose of study treatment.
28. QTc ≥ 480 msec using QTcF (based on an average of triplicate recordings).
29. History of idiopathic pulmonary fibrosis, organizing pneumonia, drug-induced pneumonitis, idiopathic pneumonitis, or evidence of active pneumonitis on screening chest computed tomography (CT) scan.
30. Radiation therapy for bone metastasis within 2 weeks or any other radiation therapy within 4 weeks prior to randomization. Patients with clinically relevant ongoing complications from prior radiation therapy are not eligible.

5.3. Lifestyle Considerations

5.3.1. Meals and Dietary Restrictions

- Refrain from consumption of red wine, Seville oranges, grapefruit, or grapefruit juice from 14 days before the start of study intervention until after the final dose.
- Casdatifan will be administered orally with or without food. Patients will drink approximately 240 mL water with dosing. This applies to QD and BID dosing.
- Cabozantinib will be administered orally at least 1 hour before or at least 2 hours after eating. Patients receiving casdatifan and cabozantinib in combination should adhere to the cabozantinib prescribing information. For additional information, consult the cabozantinib prescribing information (regionally approved prescribing information).

5.4. Screen Failures

A screen failure occurs when a patient consents to participate in the clinical study but does not satisfy eligibility requirements. A minimal set of screen failure information is required to ensure transparent reporting of screen failure patients to meet the Consolidated Standards of Reporting Trials publishing requirements and to respond to queries from regulatory authorities. Minimal information includes demography, screen failure details, eligibility criteria, and any SAE resulting from a screening-related procedure.

If during the screening period a patient’s laboratory assessment(s) are out of range, the relevant laboratory assessment(s) can be retested once and, if within the normal range and the patient is still within the screening window, the patient may be included in the study without being considered a screen failure.

However, if a patient is considered a screen failure, 1 rescreening per patient is allowed at the discretion of the principal investigator.

Patients who are rescreened are not required to sign another ICF if the originally signed consent is still the current version and the screening window has not lapsed. If a new ICF version exists at the time of rescreening or the screening window has expired, reconsent is required.

Screen failures will be recorded in the electronic case report form (eCRF).

6. STUDY INTERVENTION AND CONCOMITANT THERAPY

Study intervention is defined as any investigational intervention(s), marketed product(s), or placebo, intended to be administered to a study patient according to the study protocol.

6.1. Study Intervention Administered

Casdatifan, cabozantinib, zimberelimab, and ipilimumab are being administered in this study (Table 14, Table 15, Table 16, and Table 17, respectively). Information on administration of casdatifan and zimberelimab is found in the pharmacy manual and administration of cabozantinib and ipilimumab is found in the prescribing information (per regionally approved prescribing information). The investigator will provide instruction to the patients on administration of study intervention.

Table 14: Study Intervention – Casdatifan

**Intervention Label**	**Casdatifan**
Type	Drug
Dose Formulation	Capsule<br>Tablet
Unit Dose Strength(s)	10 mg per capsule<br>25 mg per tablet
Dosage Level(s)	All Cycles:<br>The assigned dose taken once daily (or twice daily, as defined by the dose-escalation cohort/expansion group) on Days 1-21.
Route of Administration	Oral (PO)
Sourcing	[redacted]
Packaging and Labeling	Study intervention will be provided in HDPE bottle. Each bottle will be labeled as required per country requirement.
Dosing Window	Study drug should be taken at approximately the same time each day, but within a 4-hour window of usual dosing time where the study drug is taken in the morning and with adherence to protocol-defined beverage restrictions. In dose-escalation cohorts/expansion groups where twice a day dosing is implemented, the study drug should be taken at approximately the same time each day, but within a 2-hour window in the morning and evening with adherence to the protocol-defined beverage restrictions. Furthermore, if casdatifan is taken within 2 hours of the administration of cabozantinib, food restrictions defined in the cabozantinib prescribing information should also be adhered.

HDPE = high-density polyethylene

Table 15: Study Intervention – Cabozantinib

**Intervention Label**	**Cabozantinib**
Type	Drug
Dose Formulation	Tablets
Unit Dose Strength	Per prescribing information at local site
Dosage Levels	20, 40, or 60 mg QD
Route of Administration	Oral (PO)
Sourcing	CCI [redacted]
Packaging and Labeling	Per prescribing information at local site
Dosing Window	Per regionally approved prescribing information

QD = once daily

Table 16: Study Intervention – Zimberelimab

**Intervention Label**	**Zimberelimab (AB122)**
Type	Drug
Dose Formulation	Concentrate for solution for infusion
Unit Dose Strength	360 mg, 720 mg
Dosage Levels	360 mg Q3W, 720 mg Q6W
Route of Administration	Intravenous (IV) infusion
Sourcing	CCI [redacted]
Packaging and Labeling	Study intervention will be provided in 8-mL clear Type I glass vials. Each vial is provided in a carton. The vial and carton will be labeled as required per country requirement.
Dosing Window	Administer 360 mg or 720 mg zimberelimab over a 30-minute (± 5 minutes) IV infusion on Day 1 of each 21-day cycle (or of each 42-day cycle, as applicable).

Q3W = every 3 weeks; Q6W = every 6 weeks

Table 17: Study Intervention – Ipilimumab

**Intervention Label**	**Ipilimumab**
Type	Drug
Dose Formulation	Concentrate for solution for infusion
Unit Dose Strength	50 mg, 200 mg
Dosage Level	1 mg/kg Q3W
Route of Administration	Intravenous (IV) infusion
Sourcing	CCI [redacted]
Packaging and Labeling	[redacted]
Dosing Window	Per regionally approved prescribing information

Q3W = every 3 weeks

6.2. Preparation, Handling, Storage, and Accountability

6.2.1. Casdatifan

Casdatifan is provided as capsules and tablets to sites for oral administration. Preparation methods, storage, and handling instructions for casdatifan capsules and tablets are described in a separate pharmacy manual.

- The investigator or designee must confirm appropriate temperature conditions have been maintained during transit for all study intervention received, and any discrepancies are reported and resolved before use of the study intervention.
- Only patients enrolled in the study may receive study intervention, and only authorized site staff may supply or administer study intervention. All study intervention must be stored in a secure, environmentally controlled, and monitored (manual or automated) area in accordance with the labeled storage conditions with access limited to the investigator and authorized site staff.
- The investigator, institution, or the head of the medical institution (where applicable) is responsible for study intervention accountability, reconciliation, and record maintenance (ie, receipt, reconciliation, and final disposition records).
- Further guidance and information for the final disposition of unused study interventions are provided in the pharmacy manual.

6.2.2. Cabozantinib

Prepare cabozantinib as indicated on the current regionally approved prescribing information.

6.2.3. Zimberelimab

Zimberelimab is provided as a concentrate for solution for infusion to sites for IV administration. Preparation methods, storage, and handling instructions for zimberelimab are described in a separate pharmacy manual.

6.2.4. Ipilimumab

Prepare ipilimumab as indicated on the current regionally approved prescribing information.

6.3. Measures to Minimize Bias: Randomization and Blinding

Randomization and blinding are not used in this Phase 1 study.

6.4. Study Intervention Compliance

When patients self-administer study intervention(s) at home, compliance with study intervention will be assessed at each visit. Compliance will be assessed by counting returned capsules/tablets during the site visits and documented in the source documents and relevant form. Deviation(s) from the prescribed dosage regimen should be recorded.

A record of the quantity of casdatifan dispensed to and administered by each patient must be maintained and reconciled with study intervention and compliance records. Intervention start and stop dates, including dates for intervention delays and/or dose reductions, will also be recorded.

6.5. Dose Modifications

6.5.1. Casdatifan

For any acute toxicity during the DLT (evaluation) period within dose escalation, dose modifications should be managed by withholding the casdatifan dose until resolved to Grade 1 or less. For toxicities that occur outside of the DLT period of dose escalation, or at any time during the dose expansion, dose modification will be administered as follows:

Up to a maximum of 2 dose reductions for casdatifan from drug-related toxicity is allowed. If treatment is not tolerated following the second dose reduction of casdatifan and/or cabozantinib, then discontinue dosing with either 1 or both agents. Each of the reduced dose levels are listed in the tables below; for capsule formulation examples, see Table 18 and for tablet formulation examples, see Table 19. Combination therapy dose reductions will be based on drug attribution (see Table 20, Table 21, and Table 22 for examples). For overlapping toxicity, first reduce casdatifan and if adverse event grade does not resolve to ≤ Grade 1 within 7 days, dose reduce cabozantinib. Following a dose reduction of casdatifan, the dose will not be re-escalated.

Table 18: Examples for Recommended Dose Reductions for Casdatifan for Capsule Formulation (Monotherapy)

	Initial Dose	First Dose Reduction	Second Dose Reduction
Casdatifan	[redacted] mg once daily	[redacted] mg once daily	Discontinue dosing
Casdatifan	[redacted] mg once daily	[redacted] mg once daily	[redacted] mg once daily

Casdatifan	[redacted] mg per day ([redacted] mg twice daily)	[redacted] mg once daily	[redacted] mg once daily

Table 19: Examples for Recommended Dose Reductions for Casdatifan for Tablet Formulation (Monotherapy)

	Initial Dose	First Dose Reduction	Second Dose Reduction
Casdatifan	[redacted] mg once daily	[redacted] mg once daily	Discontinue dosing
Casdatifan	[redacted] mg once daily	[redacted] mg once daily	[redacted] mg once daily
Casdatifan	[redacted] mg once daily	[redacted] mg once daily	[redacted] mg once daily
Casdatifan	[redacted] mg once daily	[redacted] mg once daily	[redacted] mg once daily

Table 20: Examples for Recommended Dose Reductions for Combination Therapy Attributed to Casdatifan Tablet

	Initial Dose	First Dose Reduction	Second Dose Reduction
Casdatifan	[redacted] mg once daily	[redacted] mg once daily	Discontinue dosing
Casdatifan	[redacted] mg once daily	[redacted] mg once daily	[redacted] mg once daily

Table 21: Examples for Recommended Dose Reductions for Combination Therapy Attributed to Cabozantinib

	Initial Dose	First Dose Reduction	Second Dose Reduction
Cabozantinib	[redacted] mg once daily	[redacted] mg once daily	[redacted] mg once daily

Table 22: Examples for Recommended Dose Reductions for Combination Therapy Attributed to Both Casdatifan Tablet and Cabozantinib

	Initial Dose	First Casdatifan Dose Reduction*	First Cabozantinib Dose Reduction	Second Casdatifan Dose Reduction*	Second Cabozantinib Dose Reduction
Casdatifan	[redacted] mg once daily	[redacted] mg once daily	N/A	Discontinue dosing	N/A
Casdatifan	[redacted] mg once daily	[redacted] mg once daily	N/A	[redacted] mg once daily	N/A
Cabozantinib	[redacted] mg once daily	N/A	[redacted] mg once daily	N/A	[redacted] mg once daily

N/A = not applicable

* For overlapping toxicity, first reduce casdatifan and if adverse event grade does not resolve to ≤ Grade 1 within 7 days, dose reduce cabozantinib (unless specified otherwise in Table 24).

Treatment modification of casdatifan for toxicity management is shown in Table 23 below.

Table 23: Casdatifan Treatment Modification Guidance for Adverse Reactions

Adverse Reaction	Severity	Treatment Modification
CCI [redacted]		

Temporary dose delay is defined as an intentional delay in the scheduled administration of a study treatment (eg, stopping treatment during a cycle or delaying the start of a new cycle until

toxicity resolves). Dose delays for reasons other than an AE, such as a surgical procedure, may be allowed with medical monitor approval. If treatment with casdatifan and/or cabozantinib has been withheld for ≤ 28 days, treatment may be resumed. If treatment with casdatifan and/or cabozantinib has been withheld for > 28 days, treatment may be resumed following assessment of clinical benefit (labs, performance status, disease assessment) and approval by the medical monitor.

Table 24: Casdatifan and Cabozantinib Treatment Modification Guidance for Adverse Reactions

Adverse Reaction	Criteria	Treatment Modification
CCI [redacted]		

Table 24: Casdatifan and Cabozantinib Treatment Modification Guidance for Adverse Reactions

Adverse Reaction	Criteria	Treatment Modification
CCI [redacted]		

6.5.2. Cabozantinib

For modifications to the dose(s) and/or dose regimen(s) of cabozantinib (including dosing delays), refer to the current regionally approved prescribing information. Following a dose reduction of cabozantinib, the dose will not be re-escalated. Cabozantinib treatment modification guidance for adverse reactions is provided in Table 25.

Table 25: Cabozantinib Treatment Modification Guidance for Adverse Reactions

Adverse Reaction	Severity	Treatment Modification
Acute myocardial infarction or arterial or venous thromboembolic events that require medical intervention	Any Grade	• Permanently discontinue cabozantinib treatment.
Development of fistula, intra-abdominal abscess and pelvic abscess	Grade 4	• Permanently discontinue cabozantinib treatment.

Table 25: Cabozantinib Treatment Modification Guidance for Adverse Reactions

Adverse Reaction	Severity	Treatment Modification
Development of GI perforation	Any Grade	• Permanently discontinue cabozantinib treatment.
Proteinuria	< 1 mg/mg<br>(< 113.1 mg/mmol)	• No change in cabozantinib treatment or monitoring.
	≥ 1 and < 3.5 mg/mg<br>(≥ 113.1 and < 395.9 mg/mmol)	• Consider confirming with a 24-hour protein assessment within 7 days.<br>○ If UPCR < 2 mg/mg or urine protein < 2 g/24 hours on 24-hour urine collection, no change in cabozantinib treatment required.<br>○ If UPCR ≥ 2 mg/mg on repeat UPCR testing or urine protein ≥ 2 g/24 hours on 24-hour urine collection, reduce cabozantinib treatment dose by 1 level or withhold cabozantinib treatment.<br>▪ If UPCR decreases to < 2 mg/mg, continue cabozantinib treatment on a reduced dose.<br>▪ If UPCR remains ≥ 2 mg/mg despite a dose reduction, consider interrupting cabozantinib treatment until UPCR decreases to < 2 mg/mg.<br>▪ Resume cabozantinib treatment at a reduced dose after withholding, unless otherwise approved by the sponsor.<br>• If UPCR < 2 mg/mg, repeat UPCR monitoring within 7 days and once per week.<br>○ If UPCR < 2 mg/mg on 2 consecutive readings, UPCR monitoring can revert to protocol-specific times (second reading is confirmatory and can be done within 1 week of first reading).
	≥ 3.5 mg/mg<br>(≥ 395.9 mg/mmol)	• Withold cabozantinib treatment pending repeat UPCR monitoring within 7 days and/or 24-hour urine protein.<br>• If ≥ 3.5 mg/mg on repeat UPCR monitoring, continue to withhold cabozantinib treatment and check UPCR every 7 days.<br>○ If UPCR decreases to < 2 mg/mg, resume cabozantinib treatment with a dose reduction by 1 level and continue monitoring UPCR until it remains < 2 mg/mg for 2 consecutive measurements.<br>○ If UPCR monitoring is determined to be stable (< 20% change) for 1 month, then continue with UPCR monitoring per protocol, or as clinically indicated.

Table 25: Cabozantinib Treatment Modification Guidance for Adverse Reactions

Adverse Reaction	Severity	Treatment Modification
Elevated ALT/AST	Grade 1	• Dose adjustment is usually not required.<br>• Consider discontinuing concomitant hepatotoxic medications and adding supportive care as indicated.
	Grade 2	• Withhold cabozantinib treatment.<br>o Resume cabozantinib treatment at the same dose or reduce cabozantinib treatment by 1 dose level at the investigator's discretion, after laboratory abnormalities have resolved to Grade ≤ 1 or baseline grade.
	≥ Grade 3	• Withhold cabozantinib treatment and consider more frequent monitoring of ALT and/or AST.<br>o Resume cabozantinib treatment with a dose reduction by 1 level after laboratory abnormalities have resolved to Grade ≤ 1 or baseline grade.<br>o Discontinue cabozantinib treatment if laboratory abnormalities cannot be reversed despite interruption of cabozantinib.
Nephrotic syndrome	Any Grade	• Permanently discontinue cabozantinib treatment.
Reversible posterior leukoencephalopathy syndrome	Any Grade	• Permanently discontinue cabozantinib treatment.
Severe hemorrhage (or recent hemoptysis greater than 2.5 mL of blood)	Any Grade	• Permanently discontinue cabozantinib treatment.
Diarrhea	Tolerable Grade 1-2 (under 48 hours)	• Continue cabozantinib treatment and consider reducing cabozantinib treatment by 1 dose level.<br>o Initiate treatment with an anti-diarrheal agent (eg, loperamide 4 mg followed by 2 mg after each episode of diarrhea [maximum: 16 mg loperamide per day]).<br>o Dietary modifications (eg, small lactose-free meals, bananas and rice).<br>o Increase intake of isotonic fluids (11.5L/day).<br>• Reassess after 24 hours:<br>o Diarrhea resolving to baseline bowel habits: Gradually add solid foods and discontinue or decrease anti-diarrheal treatment after 12 hr diarrhea-free interval.<br>o Diarrhea not resolving: Continue/resume antidiarrheal treatment.

Table 25: Cabozantinib Treatment Modification Guidance for Adverse Reactions

Adverse Reaction	Severity	Treatment Modification
	Intolerable Grade 2, Grade 2 ≥ 48 hours, or ≥ Grade 3	• Withhold cabozantinib treatment.<br>○ Ask the patient to attend the clinic.<br>○ Rule out infection (eg, stool sample for culture).<br>○ Administer antibiotics as needed (eg, if fever or Grade 3 or 4 neutropenia persists for 24 hours).<br>○ Administer fluids (1-1.5 L/day, orally or IV, as appropriate) for hydration or to correct electrolyte abnormalities.<br>○ For Grade ≥ 3, or complicated lower-grade diarrhea, consider hospitalization and IV hydration.<br>• Reassess after 24 hours.<br>○ Diarrhea resolving to baseline bowel habits or Grade 1: Consider resuming cabozantinib treatment with a dose reduction by 1 level.<br>○ Diarrhea not resolving: Start and/or continue anti-diarrheal treatment (eg, loperamide 4 mg followed by 2 mg after each episode of diarrhea [maximum: 16 mg loperamide per day]). Consider starting second-line antidiarrheal or referral to gastroenterologist.
GI: Nausea or vomiting (if persistent for > 48 hours despite optimal antiemetic)	≥ Grade 3	• For 1st and 2nd episodes: Withhold cabozantinib treatment.<br>○ Once toxicity has resolved to ≤ Grade 2 or baseline, resume cabozantinib treatment with a dose reduction by 1 level.<br>• For 3rd episode: permanently discontinue cabozantinib treatment.
Hypertensive Blood Pressure	> 150 mm Hg (systolic)<br>and < 160 mm Hg<br>OR<br>> 100 mm Hg (diastolic)<br>and < 110 mm Hg	• Optimize antihypertensive medications by adding new or additional antihypertensive medications and/or increase dose of existing medications.<br>○ If optimal antihypertensive therapy (usually to include 3 agents) does not result in BP ≤ 150 mm Hg systolic and ≤ 100 mm Hg diastolic, resume cabozantinib treatment with a dose reduction by 1 level.<br>○ If patient is symptomatic, withhold cabozantinib treatment until symptoms resolved and BP is below 150 mm Hg (systolic) and 100 mm Hg (diastolic).

Table 25: Cabozantinib Treatment Modification Guidance for Adverse Reactions

Adverse Reaction	Severity	Treatment Modification
	> 160 mm Hg (systolic)<br>OR<br>> 110 mm Hg (diastolic)	• Reduce cabozantinib treatment by 1 dose level or withhold cabozantinib treatment per investigator discretion.<br>• Add new or additional antihypertensive medications and/or increase dose of existing medications and monitor patient closely for hypotension.<br>○ If optimized antihypertensive therapy (usually to include 3 agents) does not result in BP ≤ 150 mm Hg systolic and ≤ 100 mm Hg diastolic, cabozantinib treatment should be reduced by 1 dose level or withheld.<br>• Cabozantinib treatment should be withheld if:<br>○ The upper limits of systolic BP (> 160 mm Hg) are sustained and not adequately manageable.<br>○ If systolic BP is > 180 mm Hg.<br>○ If sustained diastolic BP > 110 mm Hg.<br>○ If the patient is symptomatic.<br>• Resume cabozantinib treatment at the most tolerable dose.
Hypertensive emergency	Any Grade	• Permanently discontinue cabozantinib treatment.
Hand-Foot Syndrome/Palmar-plantar erythrodysesthesia	Grade 1	• If PPE is clinically insignificant and tolerable, continue cabozantinib treatment at current dose.<br>○ Otherwise, cabozantinib treatment should be reduced by 1 dose level.<br>• Start urea 20% cream twice daily AND high potency steroid cream (eg, clobetasol 0.05%) once daily.<br>• Reassess at least weekly; if PPE worsens at any time or does not improve after 2 weeks, proceed to the intervention guidelines for Grade 2.

Table 25: Cabozantinib Treatment Modification Guidance for Adverse Reactions

Adverse Reaction	Severity	Treatment Modification
	Grade 2	• If PPE is tolerated, continue cabozantinib treatment at current dose.<br>o Otherwise, cabozantinib treatment should be reduced by 1 dose level.<br>• If PPE is intolerable, withhold cabozantinib treatment.<br>o If PPE recovers to ≤ Grade 1, resume cabozantinib treatment at a reduced dose by 1 dose level.<br>• Continue, or start, urea 20% cream twice daily AND high potency steroid cream (eg, clobetasol 0.05%) once daily. Add analgesics (eg, NSAIDs/gamma-aminobutyric acid agonists) for pain control if needed.<br>• Reassess at least weekly; if PPE worsens or affects self-care, proceed to the intervention guidelines for Grade 3.
	Grade 3	• Withhold cabozantinib treatment until severity decreases ≤ Grade 1.<br>• Continue, or start, urea 20% cream twice daily AND high potency steroid cream (eg, clobetasol 0.05%) once daily AND analgesics (eg, NSAIDs/gamma-aminobutyric acid agonists) for pain control.<br>• If PPE recovers to ≤ Grade 1, resume cabozantinib treatment at a reduced dose by 1 dose level.<br>• If PPE does not improve within 6 weeks, permanently discontinue cabozantinib treatment.
Intolerable adverse reactions	Grade 2	• Withhold cabozantinib treatment until resolved to < Grade 1.<br>• Upon resolution/improvement (ie, return to baseline or resolution to Grade 1), reduce cabozantinib treatment by 1 dose level.<br>o If previously receiving the lowest dose (ie, 20 mg), resume at the same dose.<br>o If the lowest dose is still not tolerated, discontinue cabozantinib.

ALT = alanine aminotransferase; AST = aspartate aminotransferase; BP = blood pressure; GI = gastrointestinal; IV = intravenously; NSAIDs = non-steroidal anti-inflammatory drugs; PPE = palmar-plantar erythrodysesthesia; UPCR = urine protein-to-creatinine ratio

6.5.3. Zimberelimab or Zimberelimab in Combination With Ipilimumab

Treatment modifications and toxicity management guidelines for zimberelimab and ipilimumab are displayed in Table 26. For management/treatment guidelines for the signs and symptoms of toxicities listed in the table, please follow local guidelines (eg, European Society for Medical Oncology [ESMO], NCCN, American Society of Clinical Oncology [ASCO] etc.) or institutional standard practice and consult the study medical monitor if there are differences between these toxicity management guidelines and local guidelines and/or institutional standard practice. All toxicities will be graded according to National Cancer Institute Common Terminology Criteria for Adverse Events (NCI CTCAE) v5.0. In case of doubt, the investigator should consult with the medical monitor.

Treatment modifications of zimberelimab and ipilimumab, including dose delays, may be required in the event of treatment-related toxicity. Dose reductions are not permitted for zimberelimab or ipilimumab treatment. Zimberelimab and ipilimumab treatment may be temporarily interrupted in patients experiencing toxicity considered to be related. When zimberelimab is administered in combination with ipilimumab, withhold or permanently discontinue both zimberelimab and ipilimumab for toxicity. In the event of an imAE requiring a dose hold, both zimberelimab and ipilimumab should be held until criteria to resume treatment is met, both agents must be restarted together in cycles 1-4 (Expansion Cohorts 9 and 10). If zimberelimab and/or ipilimumab has been withheld for more than 42 days because of toxicity, treatment may be resumed after discussion with the medical monitor and the medical monitor agrees that the patient is likely to derive clinical benefit. Zimberelimab and/or ipilimumab should be restarted at the next scheduled start of a cycle when the patient meets criteria to resume treatment. If zimberelimab and ipilimumab treatment is required to be temporarily held or discontinued in the event of an imAE (eg, rash), patients may continue receiving casdatifan treatment.

Table 26: Toxicity Management Guidelines for Zimberelimab or Zimberelimab in Combination With Ipilimumab

Event	Severity	Zimberelimab	Zimberelimab + Ipilimumab
CCI [redacted]			

Table 26: Toxicity Management Guidelines for Zimberelimab or Zimberelimab in Combination With Ipilimumab

Event	Severity	Zimberelimab	Zimberelimab + Ipilimumab
CCI [redacted]			

Table 26: Toxicity Management Guidelines for Zimberelimab or Zimberelimab in Combination With Ipilimumab

Event	Severity	Zimberelimab	Zimberelimab + Ipilimumab
CCI [redacted]			

Table 26: Toxicity Management Guidelines for Zimberelimab or Zimberelimab in Combination With Ipilimumab

Event	Severity	Zimberelimab	Zimberelimab + Ipilimumab

CCI

[redacted]

Table 26: Toxicity Management Guidelines for Zimberelimab or Zimberelimab in Combination With Ipilimumab

Event	Severity	Zimberelimab	Zimberelimab + Ipilimumab

CCI

[redacted]

Table 26: Toxicity Management Guidelines for Zimberelimab or Zimberelimab in Combination With Ipilimumab

Event	Severity	Zimberelimab	Zimberelimab + Ipilimumab
CCI [redacted]			

6.6. Treatment Beyond Disease Progression

Patients can receive treatment beyond PD if the following criteria is met:

- Absence of clinical signs and symptoms indicative of PD
- Not requiring immediate therapeutic intervention
- Patient consents to treatment beyond PD
- Evidence of clinical benefit, as determined by the investigator-based review of the available clinical data for the patient

CCI [redacted]

All patients continuing study treatment after initial RECIST v1.1-defined PD must continue tumor assessments at the same interval, until further PD and/or loss of clinical benefit as judged by the investigator.

6.7. Continued Access to Study Intervention After Study Termination

The sponsor will evaluate the appropriateness of continuing to provide the investigational product casdatifan to patients assigned to these treatments after completion of the trial, based on safety and efficacy data. In general, post-trial access may be provided in compliance with local regulation if justified by the benefit-risk profile, if development of the investigational product(s) has not been discontinued, and if not commercially available.

6.8. Treatment of Overdose

For this study, casdatifan overdose is defined as > 150% of the protocol-specified dose of casdatifan.

For this study, zimberelimab overdose is defined as > 250% of the protocol-designated dose.

For this study, ipilimumab overdose is defined as > 100% of the protocol-designated dose.

For this study, cabozantinib overdose is defined as > 125% of the protocol-designated dose.

Excessive dosing (beyond that prescribed in the protocol and including overdose) in either part of the study should be recorded on the appropriate eCRF. Overdoses should also be captured as protocol deviations.

Arcus does not recommend specific treatment for an overdose.

In the event of an overdose, the investigator should:

- Contact the sponsor medical monitor immediately.
- Closely monitor the patient for any AE/SAE and laboratory abnormalities until casdatifan can no longer be detected systemically (at least 5 times the half-life) and/or until AE/SAE resolution, whichever is longer.
- Obtain a plasma sample for PK analysis at approximately 3 hours and 1.5 day after the last dose of study treatment if requested by the medical monitor (determined on a case-by-case basis).
- Investigational site symptom-directed supportive care in accordance with the investigational-site standard practice.
- Report the overdose on the SAE report form (Section 8.3.4.5) and document the quantity of the excess dose in the eCRF. If the patient experiences any AE/SAE associated with overdose, report AE in eCRF and SAE on the SAE Report Form.

Refer to the respective IB for overdose information and guidance.

Decisions regarding dose interruptions or modifications will be made by the investigator in consultation with the medical monitor based on the clinical evaluation of the patient. Dose modifications will not be allowed during the 21-day DLT (evaluation) period within the dose -escalation part of the study.

Details of signs and symptoms, clinical management, and outcome should be reported, if applicable, and should follow guidelines in Section 8.3.4. Overdoses should also be captured as a protocol deviation.

6.9. Concomitant Therapy

Any medication or vaccine (including over-the-counter or prescription medicines, recreational drugs, vitamins, and/or herbal supplements) that the patient is receiving at the time of enrollment or receives during the study must be recorded on the appropriate eCRF along with the following:

- Reason for use
- Dates of administration including start and end dates
- Dosage information including dose and frequency

The medical monitor should be contacted if there are any questions regarding concomitant or prior therapy.

6.9.1. Permitted Therapy

Concomitant medications or treatments (eg, acetaminophen/paracetamol, diphenhydramine, and vitamins) may be prescribed if considered necessary for adequate prophylactic or supportive care except for those medications identified as "excluded" in Section 6.9.2.

6.9.1.1. Surgical Procedures While on Cabozantinib

Investigators should follow local guidance regarding withholding cabozantinib prior to and following surgical procedures, including dental surgery.

6.9.1.2. Radiotherapy

For patients receiving casdatifan monotherapy, casdatifan should be held 4 days prior to palliative radiotherapy. For patients receiving casdatifan and cabozantinib combination therapy, both drugs should be held 7 days prior to radiotherapy. For patients receiving casdatifan and zimberelimab combination therapy, casdatifan should be held 4 days prior to radiotherapy. Radiation therapy can be administrated once 24 hours have elapsed since the last zimberelimab infusion. Study drugs can be restarted after resolution of any AEs related to radiation are Grade 1 or resolved. A discussion with the medical monitor is required for patients requiring radiotherapy.

6.9.2. Prohibited Therapy

For patients receiving casdatifan, the medications listed below are prohibited during the study. The sponsor must be notified if a patient receives any of these during the study.

- Any concurrent anticancer therapy, including chemotherapy, radiotherapy (except palliative radiotherapy), immunotherapy, biologic, or hormonal treatment. Concurrent use of hormones for noncancer-related conditions is permitted.
- Herbal and natural remedies are not recommended. Concomitant use of therapies that contain cannabinoids may be permitted based on the investigator's discretion.
- Strong/moderate inhibitors or strong/moderate inducers of CYP3A4.
- Inhibitors of p-gp.
- Substrates of CYP3A4, CYP2B6, CYP2C8, CYP2C9, CYP2C19 with a narrow therapeutic window, excepting hormonal contraceptives without a gestodene component, which are allowed. If such a hormonal contraceptive is used during the study, it should be recorded in the source document.
- Examples of commonly used strong/moderate/weak substrates/inducers/inhibitors for CYP enzymes and transporters can be found at https://www.fda.gov/drugs/drug-interactions-labeling/drug-development-and-drug-interactions-table-substrates-inhibitors-and-inducers and are shown in Section 10.6.

For patients receiving cabozantinib, the medications listed below are prohibited during the study. The sponsor must be notified if a patient receives any of these during the study.

- For patients receiving cabozantinib, caution should be used when treating patients with other drugs associated with QTc prolongation. Additional QTc monitoring is

suggested for patients who are treated concomitantly with QTc prolonging drugs. Refer to Section 10.7 for the list of concomitant medications associated with risk of QTc interval prolongation.

- For patients receiving cabozantinib: concomitant treatment with therapeutic doses of anticoagulants such as heparin, thrombin or Factor Xa inhibitors, or antiplatelet agents (eg, clopidogrel).

  Note: Low-dose aspirin (≤ 100 mg/daily) and prophylactic LMWH are permitted. Anticoagulation with therapeutic doses of LMWH is allowed in patients without radiographic evidence of uncontrolled brain metastases, who are on a stable dose of LMWH for at least 2 weeks before administration of the first dose of study treatment, and who have had no complications from a thromboembolic event or the anticoagulation regimen.

For patients receiving cabozantinib, refer to the regionally approved prescribing information for more information.

For patients receiving zimberelimab and/or ipilimumab, the medications prohibited during the study include systemic corticosteroids at a supraphysiologic level (10 mg of prednisone per day or equivalent). The sponsor must be notified if a patient receives any of these during the study. High dose corticosteroids given as short course for adverse event management are acceptable. Inhaled, intranasal, intra-articular, and topical corticosteroids are allowed if there is minimal systemic absorption. Systemic corticosteroids are allowed for control of infusion reactions or imAEs and must be tapered to a dose level ≤ 10 mg/day of prednisone equivalent before the next zimberelimab dose with or without ipilimumab administration. Prophylactic steroid treatment for patients with contrast allergies prior to tumor imaging is allowed.

7. DISCONTINUATION OF STUDY INTERVENTION AND PATIENT DISCONTINUATION/WITHDRAWAL

Discontinuation of specific sites or of the study as a whole are detailed in Appendix 1 (Section 10.1.10).

7.1. Discontinuation of Study Treatment

If study treatment is permanently discontinued, the primary reason for discontinuation of each treatment will be documented in the source documents and eCRF. Patients in Expansion Cohorts 4 and 7 that permanently discontinue from either casdatifan, cabozantinib, or zimberelimab for toxicity may continue the remaining study treatment if the patient is still receiving clinical benefit.

Reasons for permanent discontinuation of study treatment may include:

- Radiographic PD per RECIST v1.1
- Symptomatic deterioration attributed to PD
- Adverse event that may jeopardize patient safety if the patient continues study treatment
- Death
- Pregnancy
- Initiation of non-protocol anticancer therapy
- Patient noncompliance
- Patient decision
- Physician decision
- Lost to follow-up
- Site or study termination

For zimberelimab only: patient has completed a maximum treatment duration of 2 years for zimberelimab. However, patients are allowed to continue treatment with casdatifan until any of the criteria for permanent discontinuation of study treatment are met.

Note that discontinuation from study treatment is NOT the same as withdrawal from the study.

Upon discontinuation of study treatment, there are 3 options:

1. Continue with interventional study assessments (eg, radiographic assessments, physical exams, blood draws) and non-interventional study assessments (eg, medical record review, survival contacts by phone call), or
2. Withdrawal from interventional study assessments, but continue with non-interventional study assessments, or
3. Withdrawal from both interventional and non-interventional study assessments.

- Following end of treatment, patients should continue with follow-up assessments as outlined in the SoA, including tumor assessments until RECIST v1.1-defined PD, initiation of other anticancer therapy, or death. Protocol-specified follow-up assessments, including end of treatment assessments, survival follow-up, and documentation of anticancer treatments should continue per schedule unless the patient refuses to undergo non-interventional study procedures.
- Patient refusal of interventional study procedures with willingness to continue non-interventional assessments should be documented in the study file and should not be considered as a protocol deviation.

Withdrawn patients will not be replaced. If a patient is discontinued from the study before completion, regardless of whether they have received study drug, the reason for discontinuation must be documented in the patient's source document and eCRF. Whenever a patient is discontinued, the procedures/assessments listed for the End of Study Visit in the SoA should be performed. If a patient discontinues on Day 1, assessments completed at that visit should be entered into the eCRF for that scheduled visit. Assessments required for the End of Study Visit that are not required at the scheduled visit (eg, a complete physical examination, clinical laboratory tests) should be completed and entered into the eCRF for the End of Study Visit.

7.2. Patient Withdrawal from the Study

A patient will be discontinued from the study for the following reasons:

- Death
- Withdrawal of consent from study
- Lost to follow-up
- Site or study termination

The primary reason for withdrawal from study will be documented on the source documents and recorded on the appropriate eCRF.

If the patient withdraws from all interventional and non-interventional assessments, the sponsor may retain and continue to use any data collected before such a withdrawal of consent. After withdrawal, the study staff may, depending on country specific regulations, use a public information source (eg, county records) to obtain information about survival status.

If a patient withdraws from the interventional study assessments, samples collected up to the point of withdrawal will continue to be tested and used in the study and will be kept as part of the study records. If a patient requests that no further testing be performed on the samples or if a patient requests destruction of any samples taken and not tested, reasonable steps will be taken to comply with this request and the investigator must document this in the site study records.

7.3. Lost to Follow-Up

A patient will be considered lost to follow-up if he/she repeatedly fails to return for scheduled visits and is unable to be contacted by the study site.

The following actions must be taken if a patient fails to return to the clinic for a required study visit:

- The site must attempt to contact the patient and reschedule the missed visit as soon as possible, counsel the patient on the importance of maintaining the assigned visit schedule and ascertain whether the patient wishes to and/or should continue in the study.
- Should the patient continue to be unreachable, he/she will be considered lost to follow-up and will be withdrawn from the study if the following parameter is met, at a minimum:
  - Site staff is unable to contact the patient within 30 days after the first missed scheduled visit, with 3 documented attempts at contact.
  - Where local law/regulation permits, the sponsor [or designee] will endeavor to collect survival data on patients who are lost to follow-up through publicly available records.

7.4. Study Termination

The sponsor reserves the right to halt or terminate the study at any time for any reason.

Reasons for the early halt and any subsequent closure of the study may include:

- Discontinuation of further investigational product development.
- Incidence or severity of AEs in the study that indicates the potential hazard to patients.
- Emerging information from nonclinical studies, this current study, or FDA or European Medicines Agency (EMA) rulings, or other sources related to adverse effect that alter the benefit-risk balance and indicates potential hazard to patients.
- Patient enrollment is unsatisfactory.

Additional site and study termination details may be found in Appendix 1 (Section 10.1.10).

8. STUDY ASSESSMENTS AND PROCEDURES

- Study procedures and their schedule and frequency are summarized in the SoA (Section 4.2.2).
- Protocol waivers or exemptions are not allowed.
- Assessments/ procedures should be completed on the day indicated; if that is not possible because of a weekend, holiday, or emergency, the assessment/ procedure should be completed within the timeframe noted in the SoA.
- All planned assessments on dosing days should be completed prior to treatment administration unless otherwise indicated in the SoA.
- Urgent safety concerns should be discussed with the sponsor immediately upon occurrence or awareness to determine if the patient should continue or discontinue study intervention.
- Adherence to the study design requirements, including those specified in the SoA, is essential and required for study conduct.
- All screening evaluations must be completed and reviewed by the investigator to confirm that potential patients meet all eligibility criteria. The investigator will maintain a screening log to record details of all patients screened and to confirm eligibility or record reasons for screening failure, as applicable.
- Procedures conducted as part of the patient's routine clinical management and obtained before signing of the ICF may be utilized for screening or baseline purposes provided the procedures met the protocol-specified criteria and were performed within the timeframe defined in the SoA.
- Ad-hoc biological sample collection for safety reasons is permitted during the course of the study.
- Repeat or unscheduled samples may be taken for technical issues with the samples.

8.1. Efficacy and Survival Assessments

Planned timepoints for all efficacy assessments are provided in the SoA (Section 4.2.2).

Patients will undergo tumor assessments at study visits described in the SoA (Section 4.2.2). Also, at the investigator's discretion and based on their medical judgment, tumor assessments may be repeated at any time. Disease response will follow RECIST v1.1 (Section 10.5).

- If a disease response assessment performed as part of standard of care occurred prior to obtaining informed consent and is within 28 days prior to first dose of study treatment, the baseline disease assessment at the Screening Visit does not need to be repeated.
- Baseline/pretreatment disease assessments will include a CT scan of the chest and abdomen/pelvis, and all sites where metastases are evident, and a magnetic resonance imaging (MRI) scan of the brain for all patients with a history of brain metastases. All lesions should be assessed and documented at screening.

- The same radiographic procedures used to assess disease sites at screening should be used for all subsequent tumor assessments.
- On-treatment disease assessments will include a CT scan of the chest and abdomen/pelvis, and all locations with disease, for all patients.
- All scans should be performed with contrast. If contrast is medically contraindicated, a chest CT without contrast and an abdomen/pelvis MRI without contrast may be performed. If a brain MRI scan is contraindicated, a brain CT with contrast should be performed. If a CT scan for a tumor assessment is performed in a positron emission tomography/CT scanner, the CT acquisition must be consistent with the standards for a full contrast diagnostic CT scan.
- Assessments should be performed by the same evaluator, if possible, to ensure internal consistency across visits. Results must be reviewed by the investigator before dosing at the next cycle.
- All patients who discontinue study intervention for reasons other than PD (eg, AEs) will continue tumor assessments until death, PD, initiation of another systemic anticancer therapy, lost to follow-up, withdrawal of consent, or study termination, whichever occurs first.
- Disease response assessment beyond PD will follow RECIST v1.1 criteria (Section 10.5).

Following treatment discontinuation, patients should be followed up for survival status and any subsequent anticancer therapy following the SoA (Section 4.2.2). Where patient discontinues treatment for reasons other than PD, the patient will continue tumor assessments until PD or end of study.

8.2. Safety Assessments

Planned timepoints for all safety assessments are provided in the SoA (Section 4.2.2).

8.2.1. Physical Examinations

A full or symptom-directed examination will be conducted at timepoints detailed in the SoA (Section 4.2.2). A full physical examination should occur at screening, C1D1, treatment discontinuation, and 30-day (and 100-day post-IO for Expansion Cohorts 7, 9, and 10) Safety Follow-Up (SFU)/Early Termination Visit (ETV). At all other study visits, the physical examination can be symptom-directed at the investigator’s discretion.

A full physical examination should include an evaluation of the head, eyes, ears, nose, and throat and the cardiovascular, dermatological, musculoskeletal, respiratory, GI, genitourinary, and neurological systems. Any abnormality identified at baseline should be recorded on the appropriate eCRF page. A symptom-directed examination, which may include evaluation of the head, eyes, ears, nose, and throat; cardiovascular, neurological, respiratory, and GI systems; as well as any other abnormalities, will be performed on all days subsequent to C1D1, unless a full physical examination is clinically indicated.

Height and weight will be measured and recorded at timepoints detailed in the SoA (Section 4.2.2) and as clinically indicated throughout study treatment. The investigator should pay special attention to clinical signs related to COVID-19.

Demographic information will be collected per institutional standard practice and will specifically include, at a minimum, race and ethnicity in accordance with the FDA Collection of Race and Ethnicity Data in Clinical Trials. Guidance for Industry and Food and Drug Administration Staff (FDA 2016).

8.2.2. Vital Signs

Vital signs will include measurements of temperature, systolic and diastolic blood pressure, pulse rate, respiratory rate, and pulse oximetry (at screening and then as clinically indicated). Vital signs should be measured at pre-dose at the specified timepoints outlined in the SoA (Section 4.2.2) should be collected within a 60-minute window prior to dosing with casdatifan at the stipulated clinic visits in the SoA.

8.2.3. Electrocardiograms and Echocardiogram / Multigated Acquisition Scan (MUGA)

An echocardiogram or MUGA will be recorded at screening for both the dose-escalation and dose-expansion parts after the patient has been resting for 10 minutes in the supine position. [redacted]

CCI [redacted]

8.2.4. Clinical Safety Laboratory Tests

A list of clinical safety laboratory tests (including hematology, coagulation, serum/plasma chemistry, and urinalysis) to be performed in this study are provided in Table 27. The timing and frequency of biological sample collection for clinical safety laboratory tests are detailed in the SoA (Section 4.2.2). The tests detailed in Table 27 will be performed by the local laboratory. C1D1 safety laboratory assessments may be performed up to 7 days prior to visit. Protocol-specific requirements for inclusion or exclusion for patients are detailed in Section 5 of the protocol. Additional tests may be performed at any time during the study as deemed necessary by the investigator or required by local regulations (eg, for safety concerns).

- The investigator must review the laboratory report, document this review, and record any clinically significant changes occurring during the study as an AE in the AE section of the eCRF.
- Clinically significant abnormal laboratory findings are those which are not associated with the underlying disease, unless judged by the investigator to be more severe than expected for the patient’s condition.
- All laboratory tests with out-of-range values considered abnormal, clinically significant, or clinically significant abnormal during participation in the study or within 5 times the terminal half-life after the last dose of study medication should be followed up and repeated until the values return to normal or baseline or are no longer considered clinically significant by the investigator or referred to a relevant specialist for further investigation and management.
- All protocol-required laboratory tests, as defined in Table 27 below, must be conducted in accordance with the laboratory manual and the SoA (Section 4.2.2).
- If laboratory values from non-protocol-specified laboratory tests performed at the institution’s local laboratory require a change in patient management or are considered clinically significant by the investigator (eg, SAE or AE or dose modification), then the results should be recorded. In addition, the laboratory normal range should also be collected.

Table 27: Protocol-Required Safety Laboratory Tests

Laboratory Assessments	Parameters	
Hematology	• Hematocrit<br>• Hemoglobin<br>• Platelet count<br>• Red blood cell count<br>• MCV<br>• Percent reticulocytes	• White blood cell count (absolute counts) with differential:<br>○ Neutrophils<br>○ Lymphocytes<br>○ Monocytes<br>○ Eosinophils<br>○ Basophils
Clinical Chemistry	• ALT<br>• Albumin<br>• ALP<br>• AST<br>• Gamma-glutamyl transpeptidase (if clinically indicated)<br>• Bicarbonate or CO_2<br>• Blood urea nitrogen (BUN) or urea<br>• Calcium<br>• Chloride	• Creatinine<br>• Ferritin<br>• Glucose (may be non-fasting or fasting per site process)<br>• Iron<br>• Lactate dehydrogenase (LDH)<br>• Magnesium<br>• Phosphorus<br>• Potassium<br>• Sodium<br>• Total and either direct or indirect bilirubin<br>• Total Protein
Renal Function	• Creatinine Clearance (screening)	
Coagulation panel	• INR<br>• Fibrinogen	• PT<br>• aPTT
Lipid panel[a]	• Total cholesterol, triglycerides, HDL cholesterol, and LDL cholesterol	
Thyroid tests	• Thyroid stimulating hormone (TSH)<br>• Free Triiodothyronine (FT3)<br>• Free Thyroxine (FT4)	
Serology[b]	• HBsAg<br>• Anti-HCV antibody or qualitative HCV RNA	• HIV-1 (mandatory)<br>• HIV-2 (screening only; where locally available)<br>• COVID-19 (where regional or site policy dictates)
Other tests	• Serum pregnancy test<br>• Serum testosterone<br>• Urinalysis (dipstick and microscopy or urinalysis)	**Expansion Cohort 4 only:**<br>• CCI [redacted]

ALT = alanine aminotransferase; ALP = alkaline phosphatase; aPTT = activated partial thromboplastin time; AST = aspartate transaminase; COVID-19 = coronavirus disease 2019; HBsAg = hepatitis B surface antigen; HCV = hepatitis C virus; HDL = high-density lipoprotein; HIV = human-immunodeficiency virus; INR = international normalized ratio; LDL = low-density lipoproteins; MCV = mean corpuscular volume; PT = prothrombin time; RNA = ribonucleic acid

[a] Lipid panel only performed at Screening.

[b] Serology only performed at Screening.

8.2.5. COVID-19 Precautions

Screening for COVID-19 infection at the time of study entry is not currently recommended by the sponsor and will only be conducted if required by local regulatory, healthcare, or investigational site guidance.

On each visit to the clinical unit, patients will be asked about their potential exposure to COVID-19 infection (contacts, family members, etc) in accordance with the clinical unit's own procedures and local regulatory/healthcare authority guidance.

Patients will be closely monitored for any signs and symptoms of COVID-19 (eg, fever, dysgeusia [taste loss/change], anosmia [loss or distortion/change of smell], dry cough, dyspnea, sore throat, hypersalivation, and fatigue) throughout the study. If symptoms and/or clinical signs of infection are identified, the investigator will decide whether these findings will be handled as suspected or confirmed COVID-19. The use of testing for COVID-19 will be determined by reference to the regulatory and healthcare guidance in place locally at the time.

In the case that COVID-19 is suspected or confirmed, administration of casdatifan to that patient will be suspended, reporting to health authority as appropriate per local regulations, and the investigator will determine in discussion with the medical monitor how soon the patient may be discharged from the clinical unit, and whether this will be to their home or another healthcare facility. COVID-19 confirmed events should be followed to resolution of illness and reported as an AE/SAE, as applicable.

8.2.6. Pregnancy Testing

Pregnancy testing will be performed by obtaining serum (from whole blood) or urine samples at study visits indicated in the SoA (Section 4.2.2) for all patients reported as female sex at birth. At Screening, Day 1 of each Cycle during the first year of study treatment, Treatment Discontinuation Visit, and 30-day (and 100-day post-IO for Expansion Cohorts 7, 9, and 10) SFU/ETV, pregnancy testing will be performed on serum samples. For all other visits where pregnancy testing is required, including those from year 1 of treatment onwards, a urine or serum pregnancy test is permissible. However, if results of the urine test are ambiguous, a serum test must be conducted to confirm pregnancy status. Testing must be repeated until the result is unambiguous. Results must be reviewed prior to dosing. In the event of a patient becoming pregnant or a patient's partner becoming pregnant, refer to Section 8.3.4.7 for pregnancy reporting requirements.

8.2.7. Eastern Cooperative Oncology Group Performance Status

Patient's ECOG status (Section 10.4) will be assessed at study visits detailed in the SoA (Section 4.2.2) using the following grading scale (Table 28).

Table 28: Eastern Cooperative Oncology Group Performance Status

Grade	ECOG Performance Status
0	Fully active, able to carry on all pre-disease performance without restriction
1	Restricted in physically strenuous activity but ambulatory and able to carry out work of a light or sedentary nature, eg, light housework, office work
2	Ambulatory and capable of all self-care but unable to carry out any work activities; up and about more than 50% of waking hours
3	Capable of only limited self-care; confined to bed or chair more than 50% of waking hours
4	Completely disabled; cannot carry on any self-care; totally confined to bed or chair
5	Dead

8.3. Adverse Events, Serious Adverse Events, and Other Safety Reporting

8.3.1. Adverse and Serious Adverse Events

The investigator and any qualified designees are responsible for detecting, documenting, and recording events that meet the definition of an AE or SAE. For each AE, the investigator will make an assessment of seriousness (Section 8.3.1.1.2), causality (Section 8.3.2), and severity (Section 8.3.3). Investigators will seek information on AEs at each patient contact as defined by the SoA for each treatment cohort (Section 4.2.2). Adverse Events reported by the patient (or, when appropriate, by a caregiver, surrogate, or the patient's legally authorized representative) or noted by study personnel, will be recorded in the patient's medical record and on the AE eCRF page.

8.3.1.1. Definitions

8.3.1.1.1. Adverse Event

An AE is any untoward medical occurrence in a clinical investigation patient administered a pharmaceutical product regardless of causal attribution. An AE can therefore be any of the following:

- Any unfavorable and unintended sign (including an abnormal laboratory finding), symptom, or disease temporally associated with the use of a medicinal product, whether or not considered related to the medicinal product
- Any new disease or exacerbation of an existing disease (a worsening in the character, frequency, or severity of a known condition)
- Recurrence of an intermittent medical condition (eg, headache) not present at baseline
- Any deterioration in a laboratory value or other clinical test (eg, ECG, X-ray) that is associated with symptoms or leads to a change in study treatment or concomitant treatment or discontinuation from study treatment

- Events that are related to a protocol-mandated intervention, including those that occur prior to assignment of study treatment (eg, invasive screening procedures such as biopsies)

8.3.1.1.2. Serious Adverse Event

A serious adverse event is any adverse event that meets any of the following criteria:

- Is fatal (ie, the adverse event actually causes or leads to death)
- Is life-threatening (ie, the adverse event, in the view of the investigator, places the patient at immediate risk of death)
- Requires or prolongs inpatient hospitalization
- Results in persistent or significant disability/incapacity (ie, the adverse event results in substantial disruption of the patient's ability to conduct normal life functions)
- Is a congenital anomaly/birth defect
- Is a significant medical event in the investigator's judgment (eg, may jeopardize the patient or may require medical/surgical intervention to prevent 1 of the outcomes listed above)

8.3.1.1.3. Event Not Qualifying as Adverse Event

An event that is part of the expected course of the disease under study (ie, PD or death due to PD) should not be recorded as an AE or SAE term. If there is any uncertainty as to whether an AE is due to PD, or if the course of PD is deemed related to the investigational medicinal product by the investigator, it should be reported as an AE or SAE. All deaths that occur while on study will be recorded on the appropriate eCRF.

8.3.1.2. Classifications

The terms "severe" and "serious" are not synonymous. Severity refers to the intensity of an adverse event (eg, rated as mild, moderate, or severe, or according to the NCI CTCAE; see Section 8.3.3); the event itself may be of relatively minor medical significance (such as severe headache without any further findings).

Severity and seriousness need to be independently assessed for each AE recorded on the eCRF.

Serious adverse events are required to be reported by the investigator or designee to the sponsor immediately (ie, no more than 24 hours after learning of the event; see Section 8.3.4.5 for reporting instructions).

8.3.2. Assessment of Causality

Investigators should use their knowledge of the patient, the circumstances surrounding the event, and an evaluation of any potential alternative causes to determine whether an adverse event is considered to be related to study treatment, indicating "yes" or "no" accordingly. The following guidance should be taken into consideration (Table 29):

- Temporal relationship of event onset to the initiation of study treatment

- Course of the event, with special consideration of the effects of dose reduction, discontinuation of study treatment, or reintroduction of study treatment (as applicable)
- Known association of the event with study treatment or with similar treatments
- Known association of the event with the disease under study
- Presence of risk factors in the patient or use of concomitant medications known to increase the occurrence of the event

Table 29: Causal Attribution Guidance

**Is the adverse event suspected to be caused by study treatment on the basis of facts, evidence, science-based rationales, and clinical judgment?**	
YES	There is a plausible temporal relationship between the onset of the adverse event and administration of study treatment, and the adverse event cannot be readily explained by the patient's clinical state, intercurrent illness, or concomitant therapies; and/or the adverse event follows a known pattern of response to study treatment; and/or the adverse event abates or resolves upon discontinuation of study treatment or dose reduction and, if applicable, reappears upon re-challenge. "Related" means that the event is at least possibly related to study treatment.
NO	Evidence exists that the adverse event has an etiology other than study treatment (eg, preexisting medical condition, underlying disease, intercurrent illness, or concomitant medication); and/or the adverse event has no plausible temporal relationship to administration of study treatment (eg, cancer diagnosed 2 days after first dose of study treatment). "Unrelated" means that the event is unlikely related to study treatment.

8.3.3. Assessment of Severity

An assessment of severity grade will be made by the investigator according to the NCI CTCAE (version 5.0). Table 30 will be used for assessing severity for AEs that are not specifically listed in the NCI CTCAE.

Table 30: Adverse Event Severity Grading Scale for Events Not Specifically Listed in NCI CTCAE

Grade	Severity
1	Mild; asymptomatic or mild symptoms; clinical or diagnostic observations only; or intervention not indicated
2	Moderate; minimal, local, or non-invasive intervention indicated; or limiting age-appropriate instrumental activities of daily living[a]
3	Severe or medically significant, but not immediately life-threatening; hospitalization or prolongation of hospitalization indicated; disabling; or limiting self-care activities of daily living[b,c]
4	Life-threatening consequences or urgent intervention indicated[d]
5	Death related to adverse event[d]

NCI CTCAE = National Cancer Institute Common Terminology Criteria for Adverse Events

[a] Instrumental activities of daily living refer to preparing meals, shopping for groceries or clothes, using the telephone, managing money, etc.

[b] Examples of self-care activities of daily living include bathing, dressing and undressing, feeding oneself, using the toilet, and taking medications, as performed by patients who are not bedridden.

[c] If an event is assessed as a "significant medical event," it must be reported as a serious adverse event (see Section 8.3.4.5 for reporting instructions), per the definition of a serious adverse event in Section 8.3.1.1.2.

[d] Grade 4 and 5 events must be reported as serious adverse events (see Section 8.3.4.5 for reporting instructions) should the event meet the definition of serious adverse events in Section 8.3.1.1.2.

8.3.4. Reporting Adverse Events

The investigator is responsible for ensuring that all adverse events are recorded on the Adverse Event eCRF and reported to the sponsor in accordance with instructions provided in this section.

8.3.4.1. Adverse Event Reporting Period

The time periods for collection of AE, SAE, and other reportable safety event information for this protocol are defined in Table 31. All AEs and SAEs will be followed until resolution, stabilization, the event is otherwise explained, the patient is lost to follow-up, or the patient withdraws consent in accordance with the specified time periods.

Table 31: Time Period for Collecting AE, SAE, and Other Reportable Safety Event Information

Reportable Safety Event	Time Period[a]
SAEs related to a protocol-mandated procedure[b]	Signed informed consent until first dose of study treatment
SAEs regardless of causality	From the first dose of study treatment until 30 days (or 100 days post-IO for Expansion Cohorts 7, 9, and 10) after the last dose of study treatment or until initiation of new systemic anticancer therapy, whichever occurs first
AEs regardless of causality	From the first dose of study treatment until 30 days (or 100 days post-IO for Expansion Cohorts 7, 9, and 10) after the last dose of study treatment or until initiation of new systemic anticancer therapy, whichever occurs first
Pregnancy in female partner of a study patient or a study patient	From the first dose of study treatment until (whichever is later)<br>• 4 months after the final dose of cabozantinib<br>• 7 days after the last dose of casdatifan<br>• 120 days after the last dose of zimberelimab

AE = adverse event; eCRF = electronic case report form; IO = immune-oncology; SAE = serious adverse event

[a] Investigators are not obligated to actively seek AE or SAE information after the end of the reporting period. However, if the investigator learns of any SAE, including a death, at any time after the end of the reporting period, and he/she considers the event to be reasonably related to study treatment or study participation, the event should be promptly reported to the sponsor or its designee according to Section 8.3.4.5. Every effort should be made to follow AEs and SAEs considered related to study treatment or protocol-related procedures until a final outcome can be reported.

[b] Other clinically relevant medical occurrences that begin before the start of study treatment but after obtaining informed consent will be recorded on the medical history eCRF.

8.3.4.2. Method of Eliciting Adverse Event Information

A consistent methodology of non-directive questioning should be adopted for eliciting AE information at all patient evaluation timepoints. Open-ended and non-leading verbal questioning of the patient is the preferred method to inquire about AE occurrences. Examples of non-directive questions include: “How have you felt since your last clinic visit?”, “Have you had any new or changed health problems since you were last here?”

8.3.4.3. Procedure for Recording Adverse Events

Investigators should use correct medical terminology/concepts when recording AEs on the Adverse Event eCRF. Only 1 AE term should be recorded in the event field on the Adverse Event eCRF.

Diagnosis versus Signs and Symptoms

A diagnosis (if known) should be recorded on the Adverse Event eCRF rather than individual signs and symptoms (eg, record only liver failure or hepatitis rather than jaundice, asterixis, and elevated transaminases). However, if a constellation of signs and/or symptoms cannot be

medically characterized as a single diagnosis or syndrome at the time of reporting, each individual event should be recorded on the Adverse Event eCRF. If a diagnosis is subsequently established, all previously reported AEs/SAEs based on signs and symptoms should be nullified and replaced by 1 AE/SAE report based on the single diagnosis, with a starting date that corresponds to the starting date of the first sign or symptom of the eventual diagnosis.

Deaths

All deaths that occur during the reporting period, regardless of relationship to study treatment or PD, must be recorded on the appropriate eCRF and immediately reported to the sponsor (Section 8.3.4.4). However, if the death was due to PD, then the death will not be reported as an SAE or AE.

Hospitalization or Prolonged Hospitalization

An event that leads to hospitalization under the following circumstances should not be reported as an AE or SAE:

- Hospitalization for respite care
- Planned hospitalization required by the protocol (eg, for study treatment administration or performance of an efficacy measurement for the study)
- Hospitalization for a preexisting condition, provided that all of the following criteria are met:
  - The hospitalization was planned prior to the study or was scheduled during the study when elective surgery became necessary because of the expected normal progression of the disease
  - The patient has not experienced an AE related to the preexisting condition
  - The patient has not experienced a worsening of preexisting condition/medical history
- Hospitalization due solely to the progression of the underlying cancer

An event that leads to hospitalization under the following circumstances is not considered to be an SAE, but should be reported as an AE instead:

- Hospitalization was necessary because of patient requirement for outpatient care outside of normal outpatient clinic operating hours

Overdose

For information relating to overdose, refer to Section 6.8.

8.3.4.4. Events Requiring Expedited Reporting to the Sponsor

Certain events require immediate reporting to allow the sponsor to take appropriate measures to address potential new risks in a clinical trial. The investigator must report such events to the sponsor immediately; under no circumstances should reporting take place more than 24 hours after the investigator becomes aware of the event. The following is a list of events that the investigator must report to the sponsor within 24 hours of becoming aware of the event, regardless of relationship to investigational product:

- All SAEs (defined in Section 8.3.1.1.2)
- Pregnancies (see Section 8.3.4.7 for details on reporting requirements)
- Overdose

The investigator must report new significant follow-up information for these events to the sponsor immediately (ie, no more than 24 hours after becoming aware of the information). New significant information includes the following:

- New signs or symptoms or a change in the diagnosis
- Significant new diagnostic test results
- Change in causality based on new information
- Change in the event's outcome, including recovery
- Additional narrative information on the clinical course of the event

8.3.4.5. Serious Adverse Event Reporting Requirements

Once the investigator determines that an event meets the protocol definition of an SAE, the SAE will be reported to the sponsor **no more than 24 hours after site personnel become aware of the event**. Any follow-up information on a previously reported SAE will also be reported to the sponsor **no more than 24 hours after becoming aware of the new information**.

If the investigator does not have all the information regarding an SAE, he/she will not wait to receive additional information before notifying the sponsor of the event and completing the appropriate data collection tool. The investigator will always **provide an assessment of causality** at the time of the initial report as described in Section 8.3.2.

Serious Adverse Events will be reported to the sponsor via the Serious Adverse Event Form, submitted by email/fax as described below:

Email: Safety@arcusbio.com

Fax: Refer to the Serious Adverse Event Form for region-specific numbers.

Serious adverse event information recorded on the Serious Adverse Event Form must also be entered on the Adverse Event eCRF.

8.3.4.6. Regulatory Reporting Requirements for Serious Adverse Events

Prompt notification by the investigator to the sponsor of an SAE is essential so that legal obligations and ethical responsibilities towards the safety of patients and the safety of an investigational product under clinical investigation are met. Investigators must also comply with local requirements for reporting SAEs to the institutional review board (IRB) or independent ethics committee (IEC) or other local health authorities.

The sponsor has a legal responsibility to notify both the local regulatory authority and other regulatory agencies about the safety of an investigational product under clinical investigation. The sponsor will promptly evaluate all SAEs against cumulative product experience to identify and expeditiously communicate possible new safety findings to relevant health authorities based

on applicable legislation. The sponsor will comply with country-specific regulatory requirements relating to safety reporting to the regulatory authority, IRB/IEC, and investigators.

Upon receipt of a suspected unexpected serious adverse reaction (SUSAR) report from the sponsor or designee, the investigator must comply with all applicable requirements related to the reporting of SUSARs per institutional guidelines.

8.3.4.7. Collection of Pregnancy Information

The investigator will attempt to collect pregnancy information on any female patient or any male patient's female partner who becomes pregnant during the course of study participation. Information on pregnancies in female patients or female partners will be collected from the first dose of study treatment until up to 7 days after the last dose of casdatifan, for 4 months after the final dose of cabozantinib, and for 120 days after the last dose of zimberelimab (whichever is later). Monitoring of the mother should continue until conclusion of the pregnancy. The newborn should be monitored for up to 12 weeks for a healthy newborn or at least 1 year for a newborn with complications.

After obtaining the necessary signed ICF from the pregnant female partner directly, the investigator will record pregnancy information on the appropriate form and submit it to the sponsor within 24 hours of learning of the partner's pregnancy. The female partner will also be followed to determine the outcome of the pregnancy. Information on the status of the mother and child will be forwarded to the sponsor. Generally, the follow-up will be no longer than 12 weeks following the estimated delivery date. Any termination of the pregnancy will be reported regardless of fetal status (presence or absence of anomalies) or indication for the procedure.

In addition, abnormal pregnancy outcomes (eg, spontaneous abortion, fetal death, stillbirth, congenital anomalies, and ectopic pregnancy) are considered SAEs and must be reported using the Adverse Event eCRF and other SAE eCRFs.

8.3.4.8. Adverse Events of Special Interest

No adverse events of special interest have been defined for this study. All AEs and SAEs should be reported as described in Section 8.3.4.3 and Section 8.3.4.5, respectively.

8.3.4.9. Protocol-Specific Events That Qualify as Serious Adverse Events

All laboratory abnormalities meeting Hy's Law case criteria, defined below, qualify as SAEs and must be reported as per Section 8.3.4.5.

Patients meeting the following criteria will meet Hy's Law case criteria:

- ALT or AST $\geq 3 \times$ ULN concurrent with serum total bilirubin $> 2 \times$ ULN and serum alkaline phosphatase $< 2 \times$ ULN; and,
- No other reason is found to explain the combination of increased ALT/AST and serum total bilirubin, such as evidence of new or worsening liver disease/metastasis, preexisting liver disease, viral hepatitis, alcohol abuse, ischemia, or another drug capable of causing the observed injury.

8.3.5. Definition of Dose-Limiting Toxicities

The occurrence of any of the following toxicities during the 21-day (28-day for Expansion Cohorts 9 and 10) DLT period will be considered a DLT as judged by the investigator unless it is clearly related to the underlying malignancy, PD, or an extraneous cause:

- Any death
- Any liver laboratory abnormalities, regardless of existing liver metastases, that:
  - Meet Hy's law criteria, or
  - Any AST or ALT > 8 × ULN regardless of duration, or AST or ALT 5-8 × ULN that persists for greater than 14 days
- Any Grade 3 or greater non-hematologic (non-laboratory) toxicity, with the **exception of** nausea, vomiting, diarrhea, or fatigue where the following DLT criteria should be used:
  - Grade 3 or greater nausea/vomiting or diarrhea lasting > 72 hours with appropriate supportive care
  - Grade 3 fatigue lasting ≥ 7 days
- Grade 4 anemia
- Febrile neutropenia, or Grade 4 neutropenia lasting > 7 days
- Grade 3 or greater thrombocytopenia with clinically significant bleeding or Grade 4 thrombocytopenia lasting > 7 days
- Grade 3 or greater electrolyte abnormality that lasts > 72 hours, unless the patient has clinical symptoms, in which case all ≥ Grade 3 electrolyte abnormality regardless of duration should be considered a DLT.
- A Grade 3 or greater amylase or lipase elevation NOT associated with symptoms or clinical manifestations of pancreatitis does not need to be considered a DLT.
- Delay of a subsequent cycle for > 14 days due to persistent ≥ Grade 2 toxicity

8.4. Pharmacokinetics

Plasma samples will be collected for measurement of plasma concentrations of casdatifan (as monotherapy and in combination with cabozantinib or zimberelimab), of cabozantinib (in combination with casdatifan), and of zimberelimab (in combination with casdatifan) at study days specified in the SoA (Section 4.2.2). For casdatifan monotherapy, details on PK sampling schedules are shown in Table 32 (dose escalation) and Table 33 (dose expansion) through C18D1 on study drug. For Expansion Cohort 4, details on PK sampling schedules are shown in Table 34. For Expansion Cohort 7, 9, and 10, details on PK/ADA sampling schedules are shown in Table 35, Table 36, and Table 37, respectively. Based on emergent data, plasma samples for PK (or ADA) analysis may be added or removed or the timing of sampling may be altered at the discretion of the sponsor and will be conveyed to investigators via an administrative letter. Additionally, ad hoc samples may be collected at any time if there is a suspected safety issue.

On the days of clinic visits where PK sample collection is scheduled, the patient should withhold the morning dose until the pre-dose PK sample is collected in clinic. After the collection of pre-dose PK samples, dose administration will take place in the clinic and the dosing time will be recorded in the eCRF.

Patients must abide by all dietary restrictions indicated in Section 5.3.1, if applicable, to ensure accuracy and consistency of PK measurements.

Instructions for the collection and handling of biological samples will be provided by the sponsor in the laboratory manual. The actual date and time (24-hour clock time) of each plasma sample for PK analysis must be recorded. Every effort should be made to collect PK samples at the nominal time; however, samples collected within the following windows will not be recorded as a protocol deviation:

- Pre-dose samples: Within 60 min prior to dosing
- Post-dose samples: Within -10% to +10% of elapsed time since dosing (eg, ± 3 min for the 30-min post-dose sample)

Samples will be used to evaluate the PK of casdatifan, cabozantinib, or zimberelimab; as well as zimberelimab ADA. Samples collected for analyses of casdatifan, cabozantinib, zimberelimab plasma/serum concentrations may also be used to evaluate safety or efficacy aspects related to concerns arising during or after the study or used for exploratory analysis on metabolite identification. If one drug is discontinued in a combination therapy cohort, PK sample collection specific to the discontinued drug will cease following the first PK visit after discontinuation. PK sample collection for the continued (maintained) drug will proceed as scheduled, in accordance with the protocol. If infusion of a study drug prevents collection of the 1-hour, post-dose samples within the post-dose collection window, the sample should be collected immediately following the end of the infusion.

Urine samples will be collected at C1D1 pre-dose, and cumulative urine collection from 0 to 8 hours post-dose in the dose-escalation phase only for casdatifan monotherapy.

Table 32: Blood Sampling Schedule for Pharmacokinetic Analysis – Phase 1 Dose Escalation for Casdatifan Monotherapy (Escalation Cohorts 1-5)

Study Cycle/Day	Dosing Day?	Timepoints (hours)
C1D1	Yes	Pre-dose, 1-, 2-, 4- and 8-hours post-dose
C1D2	Yes	Pre-dose
C1D8	Yes	Pre-dose
C1D15	Yes	Pre-dose, 1-, 2-, 4- and 8-hours post-dose
C2D1	Yes	Pre-dose, 1- and 4-hours post-dose
C3D1	Yes	Pre-dose, 1- and 4-hours post-dose
Day 1 of every cycle from Cycle 4 to Cycle 18	Yes	Pre-dose
Ad hoc sample(s)	N/A	N/A

C = cycle; D = day; N/A = not applicable

Table 33: Blood Sampling Schedule for Pharmacokinetic Analysis – Phase 1 Dose Expansion for Casdatifan Monotherapy (Expansion Cohorts 1, 2, 3, 5, 6, and 8)

Study Cycle/Day	Dosing Day?	Timepoints (hours)
C1D1	Yes	Pre-dose, 1-, 2- and 4-hours post-dose
C1D2	Yes	Pre-dose
C1D8	Yes	Pre-dose
C1D15	Yes	Pre-dose, 1-, 2- and 4-hours post-dose
C2D1	Yes	Pre-dose, 1- and 4-hour post-dose
C3D1	Yes	Pre-dose, 1- and 4-hour post-dose
Day 1 of every cycle from Cycle 4 to Cycle 18	Yes	Pre-dose
Ad hoc sample(s)	N/A	N/A

C = cycle; D = day; N/A = not applicable

Table 34: Blood Sampling Schedule for Pharmacokinetic Analysis – Phase 1 Dose Expansion in Expansion Cohort 4

Study Cycle/Day	Timepoints[a] (hours)	Casdatifan PK	Cabozantinib PK
C1D1	Pre-dose	X	X
	1-hour post-dose	X	X
	2-hours post-dose	X	X
	4-hours post-dose	X	X
	8-hours post-dose	X[b]	X[b]
C1D2	Pre-dose	X	X
C1D8	Pre-dose	X	X
C1D15	Pre-dose	X	X
	1-hour post-dose	X	X
	2-hours post-dose	X	X
	4-hours post-dose	X	X
	8-hours post-dose	X[b]	X[b]
C2D1	Pre-dose	X	X
	1-hour post-dose	X	X
	4-hours post-dose	X	X
C3D1	Pre-dose	X	X
Day 1 of every 2 cycles from Cycle 4 to Cycle 18	Pre-dose	X	X
Ad hoc sample(s)	N/A	X[c]	X[c]

C = cycle; D = day; N/A = not applicable; PK = pharmacokinetics
[a] Time post-dose for each compound will be calculated based on the start of dose administration of the corresponding compound.
[b] The 8-hour post-dose samples will be only collected from patients who are enrolled in the safety run-in phase.
[c] Ad hoc samples may be collected at any time if there is a suspected safety issue.

Table 35: Blood Sampling Schedule for Pharmacokinetic/Anti-Drug Antibody Analysis – Phase 1 Dose Expansion in Expansion Cohort 7

Study Cycle/Day	Timepoints[a] (hours)	Casdatifan PK	Zimberelimab PK	Zimberelimab ADA
C1D1	Pre-dose	X	X	X
	1-hour post-dose	X		
	4-hours post-dose	X		
C1D2	Pre-dose	X		
C1D15	Pre-dose	X		
	1-hour post-dose	X		
	4-hours post-dose	X		
C2D1	Pre-dose	X	X	X
	1-hour post-dose	X		
	4-hours post-dose	X		
C3D1	Pre-dose	X	X	X
Day 1 of every 2 cycles from Cycle 4 to Cycle 18	Pre-dose	X	X	X
Day 1 of every cycle from Cycle 19 to Cycle 22.	Pre-dose		X	X
Ad hoc sample(s)	N/A	X[b]	X[b]	

ADA = anti-drug antibody; C = cycle; D = day; N/A = not applicable; PK = pharmacokinetics
[a] Time post-dose for each compound will be calculated based on the start of dose administration of the corresponding compound.
[b] Ad hoc samples may be collected at any time if there is a suspected safety issue.

Table 36: Blood Sampling Schedule for Pharmacokinetic/Anti-Drug Antibody Analysis – Phase 1 Dose Expansion in Expansion Cohort 9

Study Cycle/Day	Timepoints[a] (hours)	Casdatifan PK	Zimberelimab PK	Zimberelimab ADA
C1D1	Pre-dose	X	X	X
	1-hour post-dose	X		
	4-hours post-dose	X		
C1D2	Pre-dose	X		
C1D15	Pre-dose	X		
	1-hour post-dose	X		
	4-hours post-dose	X		
C2D1	Pre-dose	X	X	X
	1-hour post-dose	X		
	4-hours post-dose	X		
Day 1 of every cycle from Cycle 3 to Cycle 13	Pre-dose	X	X	X
Ad hoc sample(s)	N/A	X[b]	X[b]	

ADA = anti-drug antibody; C = cycle; D = day; N/A = not applicable; PK = pharmacokinetics
[a] Time post-dose for each compound will be calculated based on the start of dose administration of the corresponding compound.
[b] Ad hoc samples may be collected at any time if there is a suspected safety issue.

Table 37: Blood Sampling Schedule for Pharmacokinetic/Anti-Drug Antibody Analysis – Phase 1 Dose Expansion in Expansion Cohort 10

Study Cycle/Day	Timepoints[a] (hours)	Casdatifan PK	Zimberelimab PK	Zimberelimab ADA
C1D1	Pre-dose		X	X
C2D1	Pre-dose		X	X
C3D1	Pre-dose		X	X
C4D1	Pre-dose		X	X
C5D1	Pre-dose	X	X	X
	1-hour post-dose	X		
	4-hours post-dose	X		
C6D1	Pre-dose	X	X	X
	1-hour post-dose	X		
	4-hours post-dose	X		
Day 1 of every cycle from Cycle 7 to Cycle 13	Pre-dose	X	X	X
Ad hoc sample(s)	N/A	X[b]	X[b]	

ADA = anti-drug antibody; C = cycle; D = day; N/A = not applicable; PK = pharmacokinetics
[a] Time post-dose for each compound will be calculated based on the start of dose administration of the corresponding compound.
[b] Ad hoc samples may be collected at any time if there is a suspected safety issue.

8.5. Biological Samples for Pharmacodynamics/Biomarker Assessments

8.5.1. Pharmacodynamics

Blood samples will be collected for pharmacodynamic analysis from all patients at specified timepoints outlined in the SoA (Section 4.2.2) and further detailed in Table 38 (Expansion Cohorts 1-5), Table 39 (Expansion Cohorts 1, 2, 3, 5, 6, and 8), Table 40 (Expansion Cohorts 4 and 7), and Table 41 (Expansion Cohorts 9 and 10) up to 1 year on study treatment. Pharmacodynamic biomarkers of casdatifan in the periphery may include but are not limited to serum or plasma EPO.

The exact dates and times of biomarker sampling must be recorded on the laboratory requisition form. Refer to the laboratory manual for sample collection requirements.

The actual date and time (24-hour clock time) of each serum and plasma sample for pharmacodynamic analysis must be recorded. Every effort should be made to collect pharmacodynamic samples at the nominal time; however, samples collected within the following windows will not be recorded as a protocol deviation:

- Pre-dose samples: Within 60 min prior to dosing
- Post-dose samples: Within -10% to +10% of elapsed time since dosing (eg, ± 24 min for the 240-min/4-hr post-dose sample)

Collected samples must be stored and shipped under specified, controlled conditions at the temperatures indicated in the laboratory manual. Based on emerging data, blood samples for pharmacodynamic analysis may be altered at the discretion of the sponsor.

Table 38: Blood Sampling Schedule for EPO, [redacted] and Serum Pharmacodynamic Analysis – Phase 1 Dose Escalation (Escalation Cohorts 1-5)

[redacted]

Table 39: Blood Sampling Schedule for EPO, [redacted] and Serum Pharmacodynamic Analysis – Phase 1 Dose Expansion for Casdatifan Monotherapy (Expansion Cohorts 1, 2, 3, 5, 6, and 8)

CCI [redacted]

Table 40: Blood Sampling Schedule for EPO, [redacted] and Serum Pharmacodynamic Analysis – Phase 1 Dose Expansion for Combination Therapies (Expansion Cohort 4 and 7)

CCI [redacted]

Table 41: Blood Sampling Schedule for EPO, [redacted] and Serum Pharmacodynamic Analysis – Phase 1 Dose Expansion for Combination Therapies (Expansion Cohort 9 and 10)

[redacted]

8.5.2. Exploratory Biomarkers

[redacted]

Table 42: Proposed Biomarkers

Sample type	Timepoints	Proposed Biomarkers
Plasma, serum	• Baseline and longitudinal timepoints during casdatifan treatment	• EPO, other HIF-2α related protein markers, [redacted]<br>• [redacted]
[redacted]	• Baseline	• [redacted]
[redacted]	• Archival or pre-treatment ([redacted] if medically feasible and not posing undue risk to the patient)<br>• On treatment (C2D1 ± 7 days for Escalation and Expansion Cohorts 1-8; [redacted] days for Expansion Cohorts 9 and 10)<br>• At PD (any time after treatment discontinuation)	• [redacted]<br>• [redacted]<br>• [redacted]

C2D1 = Cycle 2 Day 1; [redacted]

EPO = erythropoietin; [redacted]

[redacted] will be collected at baseline for genomic, histopathological, and gene expression assessments for understanding of disease pathobiology and mechanisms of action/resistance of study treatments.

- [redacted] are requested to be collected on-treatment at [redacted] for assessing tumor PD and at any time after treatment discontinuation for studying mechanisms of resistance if medically feasible and do not pose undue risk to patients. [redacted]

While the goal of biomarker assessments is to provide correlative data for the clinical study, there may be circumstances when a decision is made to stop a collection, or not to perform or discontinue an analysis due to logistical or strategic reasons (eg, issues related to sample quality or inadequate number of samples). Therefore, depending upon the results obtained, the sample collection and analysis may be modified during the study at the discretion of the sponsor to remove tests no longer indicated.

The data generated from this research are exploratory in nature and are not expected to provide clinically meaningful information. Therefore, the sponsor will not provide the investigator, patient, or anyone else (eg, family members, study investigators, primary care physicians, insurers, or employers) with the results of this research, unless required by law.

8.5.3. Sample Collection and Testing

[redacted] samples will be collected and stored in accordance with applicable law.

Patients in this study will be asked to sign an optional consent to permit the use of remaining specimens from the [redacted] samples taken during the study (detailed in the SoA, Section 4.2.2) for use in future research.

Such research is for biomarker testing and hypothesis testing to address emergent questions not described elsewhere in the protocol (as part of the main study). This research may include genetic and genomic analyses (DNA), gene expression profiling (RNA), immunophenotyping, proteomics, metabolomics (blood or tissue), and/or the measurement of other analytes.

Samples may be sent to 1 or more central laboratories, collaborators, or research partners of the sponsor. Analysis may be performed by the sponsor or sponsor's designees to contribute to the understanding of disease biology, the development of related or new treatments, assays, or

research methods. Samples not analyzed during study conduct may be stored for up to fifteen (15) years after the last patient completes the study.

9. STATISTICAL CONSIDERATIONS AND ANALYSIS PLAN

This section is intended to provide an overview of the planned statistical analyses for this study, including statistical methods, algorithms, and data handling. Any deviation from the protocol-defined analysis will be described in the Clinical Study Report (CSR).

9.1. Sample Size Considerations

Dose Escalation (Escalation Cohorts 1-5)

The dose-escalation sample size with casdatifan monotherapy is determined using a rule-based 3+3 escalation method targeting a DLT threshold of 33%. The planned sample size for dose escalation is approximately 15 to 42 patients, depending on the toxicities observed. Up to [redacted] patients may be added to further evaluate safety and PK/pharmacodynamic at doses at or below the highest dose cleared in escalation. More patients may be enrolled in additional dose-escalation cohorts if recommended by the DEC to explore the safety and tolerability of casdatifan. A patient deemed not evaluable for DLT assessment will be replaced with another patient at the same dose level.

Dose Expansion (Expansion Cohorts 1-10)

The planned sample size for dose expansion is approximately [redacted] patients per dose-expansion cohort for the casdatifan monotherapy and combination therapy cohorts (Dose Expansion Cohorts 1-10). Up to an additional 30 patients may be enrolled for a maximum of [redacted] patients in any single dose-expansion cohort. To characterize safety, the chances of detecting at least 1 AE with various underlying probabilities in a ccRCC dose-expansion cohort of sample size [redacted] and [redacted] are given below in Table 43.

Table 43: Associated Probabilities of Observing ≥ 1 Adverse Event With Various Underlying Probabilities in a Sample of 30 or [redacted] Patients

Underlying True AE Rates	Probability of Observing ≥ 1 AE in a Sample of N = 30	Probability of Observing ≥ 1 AE in a Sample of N = [redacted]
[redacted]		

AE = adverse event; N = number

The sample size of N = 30 was also selected to obtain a preliminary assessment of antitumor activity in terms of ORR. Without loss of generality, with a sample size of N = 30, the lower bound of the Clopper-Pearson 90% CI will begin to exclude assumed, underlying historical control overall response rates with differences on the order of 15% to 20% (Table 44).

To account for the probability of enrolling an additional [redacted] patients in any cohort, the scenario with a sample size of N = [redacted] is also provided in Table 44.

Table 44: Representative Confidence Intervals for Various Observed Response Rates in Dose Expansion Cohorts 1-10

Historical Control ORR	ORR in Dose Expansion	Clopper-Pearson 90% CI	ORR in Dose Expansion	Clopper-Pearson 90% CI
CCI [redacted]				

CI = confidence interval; ORR = objective response rate

For Expansion Cohorts 4, 7 and 9, dose determination will be performed prior to full enrollment. Safety will be evaluated in the first 6 patients during the first 21-days (28-days for Expansion Cohort 9) of treatment. The starting dose for casdatifan will depend on the dose level cleared in the dose-escalation phase for casdatifan monotherapy. Refer to Sections 4.1.3.2, 4.1.3.3, and 4.1.3.4 for dose determination in Expansion Cohorts 4, 7, and 9, respectively.

9.2. Analysis Populations

The analysis populations are defined in Table 45.

Table 45: Analysis Populations

Population	Description
Safety Population	All patients who are enrolled and receive any amount of study treatment.
Efficacy Evaluable Population	All patients who have received any study treatment and have at least 1 post-baseline efficacy assessment or discontinue study treatment due to PD or death, excluding patients with major protocol deviations that may impact efficacy interpretation.
Pharmacokinetic Evaluable Population	All patients in the safety population with interpretable and analyzable pharmacokinetic data.
Biomarker Evaluable Population	All patients in the safety population with interpretable and analyzable biomarker data.

PD = progressive disease

Safety analyses will be performed in the safety population while efficacy analyses will be performed in the efficacy evaluable population, unless otherwise specified. Additional analysis populations such as PK and biomarker evaluable populations will be defined based on sample and data availability, as appropriate.

9.3. Statistical Analyses

9.3.1. Study Patients

9.3.1.1. Disposition

Disposition will be summarized for all enrolled study patients by cohort and overall patients combined. Summaries will include the count and percentage of patients in each analysis population and the primary reasons for discontinuing treatment and discontinuing study.

9.3.2. Baseline Characteristics

9.3.2.1. Demographics

Demographic and baseline measurements will be summarized by cohort and overall for the safety and efficacy evaluable populations.

9.3.2.2. Disease History

A summary of prior therapies will be provided for the safety and efficacy evaluable populations, as well as time since initial diagnosis/metastatic diagnosis, IMDC score (for RCC patients), and tumor stage at initial diagnosis and at study entry.

9.3.2.3. Prior Anticancer Therapies

Prior systemic anticancer therapies will be coded to anatomical therapeutic chemical (ATC) classes and preferred terms using the World Health Organization (WHO) Drug Dictionary and will be summarized as appropriate.

9.3.3. General Methodology

9.3.3.1. Summary of Treatment Cohorts

Tables and figures will be summarized by study cohort, unless otherwise specified. Tables summarizing baseline characteristics and safety data will also include a column for all patients combined. Select data may also be pooled, as warranted. Listings will be ordered by assigned study cohort, patient identification number, and assessment or event date.

9.3.3.2. Reporting Conventions

Continuous variables will be summarized to indicate the study cohort size (N), number of patients with available data (n), mean, standard deviation, median, interquartile range, minimum, and maximum values, as appropriate.

Categorical variables will be summarized by the study cohort size (N), number of patients with available data (n), number of patients in each category, and the percentage of patients in each category rounded to 1 decimal place. Percentages will not be displayed for counts of 0. Unless otherwise noted, the denominator to determine the percentage of patients in each category will be based on the number of patients with available data. Counts of missing data may also be presented, as appropriate.

Select data may be summarized as both continuous and categorical variables as appropriate. Unless otherwise specified, estimates will be presented along with their 2-sided 90% CIs. Time-to-event endpoints will be estimated using the Kaplan-Meier method.

9.3.4. Primary Endpoint Analyses

The primary endpoint for both the dose-escalation and dose-expansion phases is safety. The safety analysis will be based on the safety population, as defined in Table 45. Safety will be assessed through summaries of AEs and DLTs, as well as relevant and abnormal changes in laboratory test results, changes in vital signs, and exposure to study drugs.

9.3.4.1. Adverse Events

Treatment-emergent adverse events are AEs occurring between first dose date and the earlier date of end of AE collection period and initiation of a new anticancer therapy. At each level of summarization (eg, any AE, system organ class, preferred term, related to study treatment), patients experiencing more than 1 TEAE will be counted only once. In the summary of TEAEs by severity grade, patients will be counted once at the highest severity reported at each level of summarization.

All AE data will be presented in a data listing where TEAEs will be flagged. Serious AEs and TEAEs leading to discontinuation of study treatment(s) will also be presented in separate data listings.

9.3.5. Secondary Endpoint Analyses

9.3.5.1. Objective Response Rate

An objective response is defined as a confirmed best response of either complete response (CR) or partial response (PR), as determined by the investigator according to RECIST v1.1. Unconfirmed best overall response results may also be presented. Patients not meeting these criteria or who discontinue before obtaining post-baseline tumor assessments will be considered non-responders. The ORR is defined as the proportion of the patients who achieve an objective response and will be analyzed in the efficacy evaluable population. For each study cohort, an estimate of ORR will be presented along with its corresponding exact binomial Clopper-Pearson 95% CI.

9.3.5.2. Pharmacokinetic Analyses

Pharmacokinetic analyses will be based on the pharmacokinetic evaluable population, as defined in Table 45, with sufficient data to determine PK parameters. Individual concentration-time plots for casdatifan, cabozantinib, or zimberelimab will be presented for each patient as well as mean concentration-time plots for each cohort. PK parameters including but not limited to AUC and C_{max} will be estimated using noncompartmental methods. Summary statistics will be generated for each PK parameter for each dose cohort for each part of the study. A population PK analysis may also be performed to obtain additional PK parameters for casdatifan, cabozantinib, or zimberelimab; if performed, it will be reported separately.

9.3.6. Exploratory Analyses

9.3.6.1. Progression-Free Survival

Progression-free survival as assessed by the investigator according to RECIST v1.1 is defined from the date of first dose until first documentation of PD or death from any cause, whichever occurs first. Patients without documented PD who are still alive at the time of analysis will be censored at the time of their last tumor assessment. Patients with no post-baseline tumor assessments will be censored at the date of first dose.

Additional exploratory analysis may be performed investigating the clinical benefit for patients in [redacted].

9.3.6.2. Overall Survival

Overall survival is defined as the time from date of first dose until death due to any cause. Patients who are still alive at the time of analysis will be censored at their last known alive date.

9.3.7. Other Analyses

9.3.7.1. Pharmacodynamic/Biomarker Analyses

Pharmacodynamic/biomarker analyses will be based on the biomarker analysis population as described in Table 45. Samples collected for [redacted], and/or other exploratory markers (Section 8.5.2) will be examined by timepoint and dose group to determine biomarker changes that may occur relative to baseline. Such changes may be summarized by dose group or representative individual patients plotted over time. Possible PK/pharmacodynamic relationships will also be explored. If relevant data are available, relationships between biomarker changes and clinical outcome will be explored. Optional samples, such as tumor biopsies, may also be presented depending on available datasets.

9.3.7.2. Population Pharmacokinetic Analyses or Pharmacokinetic/Pharmacodynamic Modeling

Pharmacokinetic and pharmacodynamic data from this study may be analyzed using compartmental modeling approaches and may also be pooled with data from other studies to evaluate any association between casdatifan exposure and biomarkers of safety and/or efficacy endpoints. The results of these analyses, if performed, may be reported separately.

9.4. Interim Analysis

No interim analyses are planned. Safety data will be evaluated throughout dose escalation and during study conduct.

10. SUPPORTING DOCUMENTATION AND OPERATIONAL CONSIDERATIONS

10.1. Appendix 1: Regulatory, Ethical, and Study Oversight Considerations

10.1.1. Regulatory and Ethical Considerations

- This study will be conducted in accordance with the protocol and with the following:
  - Consensus ethical principles derived from international guidelines including the Declaration of Helsinki and Council for International Organizations of Medical Sciences (CIOMS) international ethical guidelines
  - Applicable ICH Good Clinical Practice (GCP) guidelines
  - Applicable laws and regulations (eg, FDA regulations, EU Clinical Trial Directive [2001/20/EC], etc)
  - Guidelines included in the American Thoracic Society Statement: Guidelines for the Six-Minute Walk Test (American Thoracic Society 2022)
- The protocol, protocol amendments, ICF, IB, and other relevant documents (eg, advertisements) must be submitted to an IRB/IEC by the investigator and reviewed and approved by the IRB/IEC before the study is initiated.
  - Any amendments to the protocol will require IRB/IEC approval before implementation of changes made to the study design, except for changes necessary to eliminate an immediate hazard to study patients.
  - Protocols and any substantial amendments to the protocol will require health authority approval prior to initiation except for changes necessary to eliminate an immediate hazard to study patients.
  - The investigator will be responsible for the following:
    a. Providing written summaries of the status of the study to the IRB/IEC annually or more frequently in accordance with the requirements, policies, and procedures established by the IRB/IEC
    b. Notifying the IRB/IEC of SAEs or other significant safety findings as required by IRB/IEC procedures
    c. Providing oversight of the conduct of the study at the site and adherence to requirements of 21 Code of Federal Regulations (CFR), ICH guidelines, the IRB/IEC, European regulation 536/2014 for clinical studies (if applicable), and all other applicable local regulations

10.1.2. Financial Disclosure

Investigators and sub-investigators will provide the sponsor with sufficient, accurate financial information as requested to allow the sponsor to submit complete and accurate financial certification or disclosure statements to the appropriate regulatory authorities. Investigators are

responsible for providing information on financial interests before the study, during the study, and for 1 year after completion of the study.

10.1.3. Informed Consent Process

- The investigator or his/her representative will explain the nature of the study, including the risks and benefits, to the patients or their legally authorized representative, as defined by applicable local laws, and answer all questions regarding the study.
- Patients must be informed that their participation is voluntary. Patients or their legally authorized representatives will be required to sign a statement of informed consent that meets the requirements of 21 CFR 50, local regulations, ICH guidelines, privacy and data protection requirements, where applicable, and the IRB/IEC or study center.
- The medical record must include a statement that written informed consent was obtained before the patients were enrolled in the study and the date the written consent was obtained. The authorized person obtaining the informed consent must also sign the ICF.
- If applicable, the ICF will contain separate sections for any optional procedures. The investigator or authorized designee will explain to each patient the objectives, methods, and potential risks associated with each optional procedure. Patients will be told that they are free to refuse to participate and may withdraw their consent at any time for any reason. A separate, specific signature will be required to document a patient's agreement to participate in optional procedures. Patients who decline to participate will not provide a separate signature.
- Patients must be reconsented to the most current version of the ICF(s) during their participation in the study. The source document should document the reconsent process and that written informed consent was obtained using the updated/revised ICF for continued participation in the study.
- A copy of the ICF(s) must be provided to the patients or their legally authorized representative.

Patients who are rescreened are not required to sign another ICF if the original consent is still the current version, and the screening window has not lapsed. If a new ICF version exists at the time of rescreening or the screening window has expired, then reconsent is required.

The ICF will contain a separate optional consent that addresses the use of remaining specimens from the blood and tissue samples taken during the study (detailed in the SoA, Section 4.2.2) for use in future research. The investigator or authorized designee will explain to each patient the objectives of future research. Patients will be told that they are free to refuse to participate and may withdraw their consent at any time and for any reason during the storage period. A separate signature will be required to document a patient's agreement to allow any remaining specimens to be used for future research. Patients who decline to participate in this optional research will not provide this separate signature.

10.1.4. Data Protection

- Patients will be assigned a unique identifier by the sponsor. Any patient records or datasets that are transferred to the sponsor will contain the identifier only; patient names or any information which would make the patient identifiable will not be transferred.
- The patient must be informed that his/her personal study-related data will be used by the sponsor in accordance with local data protection law. The level of disclosure must also be explained to the patient who will be required to give consent for their data to be used as described in the ICF.
- The patient must be informed that his/her medical records may be examined by Clinical Quality Assurance auditors or other authorized personnel appointed by the sponsor, the patient's personal medical doctor or other appropriate medical personnel responsible for the patient's welfare, by appropriate IRB/IEC members, and by inspectors from regulatory authorities.
- Under the General Data Protection Regulation (GDPR), patients have certain data protection rights in respect of personal data. One key right is that of objection to the processing of personal data in the context of a clinical study. Patients of a clinical study also have a right to access, rectify, restrict, and delete personal data, and the right to data portability, patient to limitations provided for in the GDPR Regulations. GDPR data privacy rights will be provided in detail in the Informed Consent Document and Patient Information Sheet and indicate that data and bodily material will be stored for no more than 25 years.

10.1.5. Committee Structure

This study utilizes a DEC to ensure the continued safety of patients in the study.

10.1.5.1. Dose Escalation Committee

To ensure patients' safety during the dose escalation part of the study, a DEC will review all available safety data from each cohort prior to escalation to the next highest dose of casdatifan. The DEC will consist of members from the sponsor, investigators, the lead statistician, and may include other subject matter experts as necessary. During DEC meetings, aggregated safety data and individual patient data derived from medical data listing reviews will be presented, reviewed, and discussed. Once data are reviewed, the DEC will determine how to proceed (eg, escalate, de-escalate, etc). DEC members eligible to vote consist of the sponsor or designee and an investigator from each enrolling site.

Additional details for the DEC are provided in the DEC charter.

10.1.6. Dissemination of Clinical Study Data

All data generated in this study required to be publicly available will follow all local and global reporting requirements. Any request to delay submission of study results will follow the appropriate regulatory procedures, if warranted.

Peer reviewed publication(s) of the study results will follow the publication policy detailed in Section 10.1.11.

10.1.7. Reporting Requirements for Serious Breaches

A ‘serious breach’ means a breach likely to affect to a significant degree the safety and rights of a patient or the reliability and robustness of the data generated in the clinical study.

Immediate notification by the investigator to Arcus of any (potential) serious breach of the protocol, or regulations is essential so that legal and ethical obligations are met.

- In certain regions/countries, Arcus has a legal responsibility to notify both the local regulatory authority and other regulatory agencies about such breaches.
  - Arcus will comply with country-specific regulatory requirements relating to serious breach reporting to the regulatory authority, IRB/IEC, and investigators. If EU Clinical Trials Regulation 536/2014 applies, Arcus is required to enter details of serious breaches into the EMA Clinical Trials Information System (CTIS). It is important to note that redacted versions of serious breach reports will be available to the public via CTIS.
- The investigators should have a process in place to ensure that:
  - The site staff or service providers delegated by the investigator/institution are able to identify the occurrence of a (potential) serious breach.
  - If any (potential) serious breach occurs over the course of the study, investigators or other site personnel will inform Arcus through their contract research organization (CRO) representative, immediately after they become aware of the event.

10.1.8. Data Management and Quality Assurance

- Patient data relating to the study will be recorded on the appropriate eCRFs. Sites will be responsible for data entry into the electronic data capture (EDC) system and will receive training for data entry. The investigator is responsible for verifying that data entries are accurate and correct by electronically signing the case report form (CRF).
- Guidance on completion of CRFs will be provided in CRF Completion Guidelines.
- The investigator must permit study-related monitoring, quality assurance audits, IRB/IEC review, and regulatory agency inspections and provide direct access to source data documents.
- Monitoring details describing strategy, including definition of study critical data items and processes (eg, risk-based initiatives in operations and quality such as risk management and mitigation strategies and analytical risk-based monitoring), methods, responsibilities, and requirements, including handling of noncompliance issues and monitoring techniques (central, remote, or on-site monitoring) are provided in the monitoring plan.

- The sponsor or designee is responsible for the data management of this study, including quality checking of the data.
- The sponsor assumes accountability for actions delegated to other individuals (eg, CROs).

10.1.8.1. Record Retention

- At the end of the study, the investigator will receive patient data for his/her site in a readable format on a compact disc (or other readable digital format) that must be kept with the study records. Acknowledgement of receipt of the data is required. Records and documents (including signed ICFs) pertaining to the conduct of this study must be retained by the investigator for 2 years after study completion unless local regulations or institutional policies require a longer retention period. No records may be destroyed during the retention period without the written approval of the sponsor. No records may be transferred to another location or party without written notification to the sponsor.

10.1.9. Source Documents

- Source documents provide evidence for the existence of the patient and substantiate the integrity of the data collected. The investigator must maintain accurate documentation (source data) that supports the information entered in the CRF and keep source documents filed at the investigator's site.
- Data entered in the eCRF that are transcribed from source documents must be consistent with the source documents or the discrepancies must be explained. The investigator may need to request previous medical records or transfer records, depending on the study. Also, current medical records must be available.
- Source documents (paper or electronic) are original documents, data, and records. They include, but are not limited to, hospital records, clinical and office charts, laboratory notes, memoranda, patient-reported outcomes, evaluation checklists, pharmacy dispensing records, recorded data from automated instruments, copies of transcriptions that are certified after verification as being accurate and complete, microfiche, photographic negatives, microfilm or magnetic media, X-rays, patient files, and records kept at pharmacies, laboratories, and medico technical departments involved in a clinical trial.
- Study monitors will perform ongoing source data verification to confirm that data entered into the CRF by authorized site personnel are accurate, complete, and verifiable from source documents; that the safety and rights of patients are being protected; and that the study is being conducted in accordance with the currently approved protocol and any other study agreements, ICH GCP, and all applicable regulatory requirements.

10.1.10. Study and Site Start and Closure

First Act of Recruitment

The study start date is the date on which the clinical study will be open for recruitment of patients.

The first act of recruitment is the first site activated and will be the study start date.

Study/Site Termination

The sponsor or designee reserves the right to close the study site or terminate the study at any time for any reason at the sole discretion of the sponsor. Study sites will be closed upon study completion. A study site is considered closed when all required documents and study supplies have been collected and a study-site closure visit has been performed.

The investigator may initiate study-site closure at any time, provided there is reasonable cause and sufficient notice is given in advance of the intended termination.

Reasons for the early closure of a study (by the sponsor or designee) or study site by the sponsor or investigator may include:

For study termination:

- Discontinuation of further study intervention development
- The incidence or severity of AEs in this or other studies indicate the potential hazard to patients
- Patient enrollment is unsatisfactory

For site termination:

- Failure of the investigator to comply with the protocol, the requirements of the IRB/IEC or local health authorities, the sponsor's procedures, or GCP guidelines
- Inadequate or no recruitment (evaluated after a reasonable amount of time) of patients by the investigator
- Total number of patients included in the study occurred earlier than expected

If the study is prematurely terminated or suspended, the sponsor shall promptly inform the investigators, the IECs/IRBs, the regulatory authorities, and any CRO(s) used in the study of the reason for termination or suspension, as specified by the applicable regulatory requirements. The investigator shall promptly inform the patient and should assure appropriate patient therapy and/or follow-up.

10.1.11. Publication Policy

- The results of this study may be published in peer-reviewed journals or presented at scientific meetings to openly provide information on the trial to health care professionals and to the public. If this is foreseen, the investigator must agree to submit all manuscripts or abstracts to the sponsor before submission or presentation. This allows the sponsor to protect proprietary information and to provide comments

based on information from other studies that may not yet be available to the investigator.

- The sponsor will comply with the requirements for publication of study results. In accordance with standard editorial and ethical practice, the sponsor will generally support publication of multicenter studies only in their entirety and not as individual site data. In this case, a coordinating investigator will be designated by mutual agreement.
- Authorship will be determined by mutual agreement and in line with International Committee of Medical Journal Editors authorship requirements.

10.2. Appendix 2: Contraceptive and Barrier Guidance

10.2.1. Definitions

Woman of Childbearing Potential (WOCBP)

Females in the following categories are considered WOCBP (fertile):

- Following menarche
- From the time of menarche until becoming postmenopausal unless permanently sterile (see below)
- A postmenopausal state is defined as no menses for 12 months without an alternative medical cause.
  - A high follicle-stimulating hormone (FSH) level in the postmenopausal range may be used to confirm a postmenopausal state in females not using hormonal contraception or hormonal replacement therapy (HRT). However, in the absence of 12 months of amenorrhea, confirmation with more than 1 FSH measurement is required.
  - Females on HRT and whose menopausal status is in doubt will be required to use 1 of the non-estrogen hormonal highly effective contraception methods if they wish to continue their HRT during the study. Otherwise, they must discontinue HRT to allow confirmation of postmenopausal status before study enrollment.
- Permanent sterilization methods (for the purpose of this study) include:
  - Documented hysterectomy
  - Documented bilateral salpingectomy
  - Documented bilateral oophorectomy
  - For individuals with permanent infertility due to an alternate medical cause other than the above (eg, Mullerian agenesis, androgen insensitivity, gonadal dysgenesis), investigator discretion should be applied to determine study entry.

Note: Documentation can come from the site personnel's review of the patient's medical records, medical examination, or medical history interview.

- If fertility is unclear (eg, amenorrhea in adolescents or athletes) and a menstrual cycle cannot be confirmed before first dose of study intervention, additional evaluation should be considered.

Woman of Nonchildbearing Potential (WONCBP)

Females in the following categories are considered WONCBP:

- Premenopausal female with permanent infertility due to 1 of the following:
  - Documented hysterectomy
  - Documented bilateral salpingectomy

- Documented bilateral oophorectomy
- For individuals with permanent infertility due to an alternate medical cause other than the above (eg, Mullerian agenesis, androgen insensitivity, gonadal dysgenesis), investigator discretion should be applied to determine study entry.

Note: Documentation can come from the site personnel's review of the patient's medical records, medical examination, or medical history interview.

- Postmenopausal female
  - A postmenopausal state is defined as no menses for 12 months without an alternative medical cause.
    - i. A high FSH level in the postmenopausal range may be used to confirm a postmenopausal state in females not using hormonal contraception or HRT. However, in the absence of 12 months of amenorrhea, confirmation with more than 1 FSH measurement is required.
    - ii. Females on HRT and whose menopausal status is in doubt must discontinue HRT to allow confirmation of postmenopausal status before study enrollment.

10.2.2. Contraception Guidance for Male and Female Patients

Male Patients

Nonclinical toxicity findings indicate that casdatifan may temporarily or permanently impact the male sperm cycle. Males may wish to consider sperm banking prior to dosing and should discuss this with the principal investigator or study physician.

Male patients are eligible to participate if they agree to the following contraception requirement during the study intervention period and for 7 days after the last dose of casdatifan, for 4 months after final dose of cabozantinib, and for 120 days after the last dose of zimberelimab (whichever is later):

- Refrain from donating sperm for 7 days after the last dose of casdatifan.
- Refrain from donating sperm for 4 months after final dose of cabozantinib.
- Refrain from donating sperm for 120 days after the last dose of zimberelimab.

PLUS, either:

- Be abstinent from heterosexual intercourse as their preferred and usual lifestyle (abstinent on a long-term and persistent basis) and agree to remain abstinent

OR

- Must agree to use contraception as detailed below:
  - Agree to use a male condom and should also be advised of the benefit for a female partner to use a highly effective method of contraception as a condom may break or leak when having sexual intercourse with a WOCBP who is not currently pregnant

- Agree to use a male condom with female partner who uses an additional highly effective contraceptive method with a failure rate of < 1% per year as described in Table 46 when having sexual intercourse with a WOCPB who is not currently pregnant.

Female Patients

Female patients are eligible to participate if they agree to use at least 1 highly effective measure of contraception during the study intervention period and for 7 days after the last dose of casdatifan, or 4 months after the final dose of cabozantinib, or 120 days after the last dose of zimberelimab (whichever is later). HIF-2α inhibitors have been shown to impact the effectiveness of hormonal methods of contraception. If hormonal method is preferred form of contraception, a second method of contraception in addition should also be used. Female patients should not donate ova until 7 days after the last dose of casdatifan, or until 4 months after the final dose of cabozantinib, or until 120 days after the last dose of zimberelimab (whichever is later). A summary of contraceptives allowed during the study is provided in Table 46.

Table 46: Contraceptives Allowed During the Study[a]

**Highly Effective Methods[b] That Have Low User Dependency**
• Implantable progestogen-only hormone contraception associated with inhibition of ovulation[c]
• Intrauterine device (IUD)
• Intrauterine hormone-releasing system (IUS)[c]
• Bilateral tubal occlusion
• Azoospermic partner (vasectomized or due to a medical cause)<br>• *Azoospermia is a highly effective contraceptive method provided that the partner is the sole sexual partner of the woman of childbearing potential and the absence of sperm has been confirmed. If not, an additional highly effective method of contraception should be used. Spermatogenesis cycle is approximately 90 days.*<br>Note: documentation of azoospermia for a male patient can come from the site personnel's review of the patient's medical records, medical examination, or medical history interview.
**Highly Effective Methods[b] That Are User Dependent**<br>***NOTE: HIF-2α inhibitors have been shown to impact the effectiveness of hormonal methods of contraception. If hormonal method is preferred form of contraception, a second method of contraception should also be used.***
• Combined (estrogen- and progestogen-containing) hormonal contraception associated with inhibition of ovulation<br>○ oral<br>○ intravaginal<br>○ transdermal<br>○ injectable
• Progestogen-only hormone contraception associated with inhibition of ovulation[c]<br>○ oral<br>○ injectable
• Sexual abstinence<br>*Sexual abstinence is considered a highly effective method only if defined as refraining from heterosexual intercourse during the entire period of risk associated with the study intervention. The reliability of sexual abstinence needs to be evaluated in relation to the duration of the study and the preferred and usual lifestyle of the patient.*

[a] Contraceptive use by males or females should be consistent with local regulations regarding the use of contraceptive methods for those participating in clinical studies.

[b] Failure rate of < 1% per year when used consistently and correctly. Typical use failure rates differ from those when used consistently and correctly.

[c] Male condoms must be used in addition to hormonal contraception. If locally required, in accordance with Clinical Trial Facilitation Group (CTFG) guidelines, acceptable contraceptive methods are limited to those which inhibit ovulation as the primary mode of action.

Note: Periodic abstinence (calendar, symptothermal, postovulation methods), withdrawal (coitus interruptus), spermicides only, and lactational amenorrhea method (LAM) are not acceptable methods of contraception for this study. Male condom and female condom should not be used together (due to risk of failure from friction).

10.3. Appendix 3: Genetics

Use/Analysis of DNA

- Genetic variation may impact a patient's response to study intervention, susceptibility to, and severity and progression of disease. Variable response to study intervention may be due to genetic determinants that impact intervention absorption, distribution, metabolism, and excretion; mechanism of action of the intervention; disease etiology; and/or molecular subtype of the disease being treated. Therefore, where local regulations and IRB/IEC allow, a blood and tumor sample may be collected for DNA analysis from consenting patients.
- DNA samples will be used for research related to future casdatifan combination therapy in solid tumors or evaluation of new assay technologies. They may also be used to develop tests/assays, including diagnostic tests related to future casdatifan combination therapy and/or therapies of this drug class, and advanced malignancies. Genetic research may consist of the analysis of 1 or more candidate genes or the analysis of genetic markers throughout the genome. Patients will not have access to the results of any genetic research conducted under the study.
- DNA samples may be analyzed for whole exome sequencing and relationship to clinical outcomes in response to future casdatifan combination therapies. Additional analyses may be conducted if it is hypothesized that this may help further understand the clinical data.
- The samples may be analyzed as part of a multi-study assessment of genetic factors involved in the response to casdatifan or study interventions of this class to understand the study disease or related conditions.
- The results of genetic analyses may be reported in the CSR or in a separate study summary.
- The sponsor will store the DNA samples in a secure storage space with adequate measures to protect confidentiality.
- The samples will be retained while research on casdatifan-based therapy continues but no longer than 15 years or other period as per local requirements.

10.4. Appendix 4: Eastern Cooperative Oncology Group (ECOG) Performance Status

Grade	ECOG Performance Status
0	Fully active, able to carry on all pre-disease performance without restriction
1	Restricted in physically strenuous activity but ambulatory and able to carry out work of a light or sedentary nature, eg, light housework, office work
2	Ambulatory and capable of all self-care but unable to carry out any work activities; up and about more than 50% of waking hours
3	Capable of only limited self-care; confined to bed or chair more than 50% of waking hours
4	Completely disabled; cannot carry on any self-care; totally confined to bed or chair
5	Dead

10.5. Appendix 5: RECIST 1.1

Tumor response will be assessed according to the RECIST guideline (Version 1.1) (Eisenhauer et al. 2009), as described below.

Measurability of Tumor at Baseline

At baseline, tumor lesions/lymph nodes will be categorized as measurable or non-measurable as follows:

Measurable

Tumor lesions: Must be accurately measured in ≥ 1 dimension (longest diameter in the plane of measurement to be recorded) with a minimum size of:

- 10 mm by CT scan (CT scan slice thickness no greater than 5 mm)

Malignant lymph nodes: To be considered pathologically enlarged and measurable, a lymph node must be ≥ 15 mm in short axis when assessed by CT scan (CT scan slice thickness recommended to be no greater than 5 mm)

Non-measurable

- All other lesions (or disease sites), including small lesions (longest diameter < 10 mm or pathological lymph nodes with ≥ 10 to < 15 mm short axis)
- Lesions considered truly non-measurable include the following: leptomeningeal disease, ascites, pleural/pericardial effusion, inflammatory breast disease, lymphangitic involvement of skin or lung, and abdominal masses/abdominal organomegaly identified by physical examination that is not measurable by reproducible imaging techniques

Tumor Response Evaluation

Baseline Documentation of Target and Nontarget Lesions

Target lesions

- When > 1 measurable lesion is present at baseline, all lesions up to a maximum of 5 lesions total (and a maximum of 2 lesions per organ) representative of all involved organs should be identified as target lesions
- It may be the case that, on occasion, the largest lesion does not lend itself to reproducible measurement, in which circumstance the next largest lesion that can be measured reproducibly should be selected

Nontarget lesions

- All other lesions (or disease sites), including pathological lymph nodes, should be identified as nontarget lesions
- It is possible to record multiple nontarget lesions involving the same organ as a single item (eg, "multiple enlarged pelvic lymph nodes" or "multiple liver metastases")

Evaluation of Target Lesions

Target lesions will be evaluated, and response recorded as defined in Table 47.

Table 47: Response Based on Evaluation of Target Lesions at Each Assessment

Complete response	Disappearance of all target lesions; if a pathologic lymph node, reduction in the shortest axis to < 10 mm[a]
Partial response[b]	≥ 30% decrease in the sum of the diameters of target lesions relative to the baseline sum diameters[c]
Stable disease[b,d]	Neither a sufficient reduction to qualify as a partial response nor a sufficient increase to qualify as progression[c]
Progressive disease[b]	≥ 20% increase in the sum diameters relative to the smallest sum diameters recorded (including the baseline sum diameters) in conjunction with an increase of at least 5 mm in that smallest sum diameters or the appearance of 1 or more new lesions[c,e]

[a] For each pathologic lymph node considered a target lesion, the node must have a short axis measuring < 10 mm to be considered as a complete response. In such cases, the sum diameters may not be zero (as a normal lymph node can have a short axis of < 10 mm).
[b] For each pathologic lymph node considered a target lesion, the measurement of the short axis of the node is to be included in the sum diameters when determining partial response, stable disease, and progression.
[c] In this study, the "baseline sum diameter" is calculated based on the lesion measurements obtained at screening.
[d] Duration of stable disease is measured from the date of the first dose of study treatment until criteria for progressive disease are met based on the smallest sum diameters recorded (including the baseline sum diameters).
[e] The finding of a new lesion should be unequivocal and not possibly attributable to a difference in imaging modality or scanning technique. Postbaseline, fluorodeoxyglucose positron emission tomography (FDG PET) may be useful in assessing new lesions apparent on computed tomography (CT) scan.

Evaluation of Nontarget Lesions

Nontarget lesions will be evaluated, and response recorded as defined in Table 48.

Table 48: Response Based on Evaluation of Nontarget Lesions at Each Assessment

Complete response	Disappearance of all nontarget lesions; all lymph nodes must be nonpathologic in size (ie, ≥ 10 mm on the short axis)
Not complete response or not progressive disease	Persistence of 1 or more nontarget lesions
Progressive disease	Unequivocal progression[a] of any existing nontarget lesion or the appearance of 1 or more new lesions[b]

[a] The patient should stop study treatment, even in the presence of a partial response or stable disease, based on an assessment of target lesions.
[b] The finding of a new lesion should be unequivocal and not possibly attributable to a difference in imaging modality or scanning technique. Postbaseline, fluorodeoxyglucose positron emission tomography (FDG PET) may be useful in assessing new lesions apparent on computed tomography (CT) scan.

New Lesions

The appearance of new malignant lesions denotes progressive disease; therefore, some comments on detection of new lesions are important. There are no specific criteria for the identification of new radiographic lesions; however, the finding of a new lesion should be unequivocal, ie, not attributable to differences in scanning technique, change in imaging modality, or findings thought to represent something other than tumor (eg, some "new" bone lesions may be simply healing or flare of pre-existing lesions). This is particularly important when the patient's baseline

lesions show PR or CR. For example, necrosis of a liver lesion may be reported on a CT scan report as a “new” cystic lesion, which it is not.

Evaluation of Overall Response

Overall response based on the evaluation of target and nontarget lesions will be determined as shown in Table 49.

Table 49: Evaluation of Overall Response at Each Assessment

Target Lesions	Nontarget Lesions	New Lesions	Overall Response
Complete response	Complete response	No	Complete response
Complete response	Not evaluable[a]	No	Partial response
Complete response	Not complete response/ non-progressive disease	No	Partial response
Partial response	Non-progressive disease and not evaluable[a]	No	Partial response
Stable disease	Non-progressive disease and not evaluable[a]	No	Stable disease
Not all evaluated	Non-progressive disease	No	Not evaluable
Progressive disease	Any	Yes or No	Progressive disease
Any	Progressive disease	Yes or No	Progressive disease
Any	Any	Yes	Progressive disease

[a] Not evaluable is defined as either when no or only a subset of lesion measurements are made at an assessment.

10.6. Appendix 6: Examples of Inhibitors for P450-Mediated Metabolism

FDA provides a list of substrates, inhibitors and inducers for drug development and drug interactions. Examples of p-gp inhibitors for transporters and clinical index inhibitors for P450-mediated metabolism are noted in the FDA guidance at: https://www.fda.gov/drugs/drug-interactions-labeling/drug-development-and-drug-interactions-table-substrates-inhibitors-and-inducers. Refer to this FDA website for the most updated lists or country-specific regulatory authority listing.

10.7. Appendix 7: Concomitant Medications Associated With Risk of QTc Interval Prolongation and/or Torsades de Pointes

Casdatifan has not demonstrated adverse effects on QT prolongation in studies to date; however, caution is advised when administering casdatifan with any drug that may prolong QT.

A vast number of medications prolong the QT interval. They are preferably classified based on the degree of QT prolongation they induce. This is specifically medication-dependent. For example, many commonly used medications, such as diphenhydramine and azithromycin, exhibit QT-prolonging effects. However, the degree of QT prolongation is not severe enough to warrant caution in patients who do not present underlying QTc prolongation. These medications bind to the human ether-related gene (hERG) channels and reduce electrical conduction through the potassium ion channels delaying repolarization of the conduction system of the heart.

Caution is advised when combining QT-prolonging medications or when using these medications in patients with electrolyte abnormalities. Table 50 below shows a list of potential medications that may prolong QT interval.

Consult the medical monitor prior to enrolling any patients on casdatifan or starting them on new medication while on study that you believe may prolong QT. Additionally, consultation with your local pharmacist is advised.

Table 50: Concomitant Medications Associated With Risk of QTc Interval Prolongation and/or Torsades de Pointes

	Drug Categories				
**Risk**	**Antiarrhythmic Drugs**	**Common Antibacterial and Antifungal Drugs**	**Prokinetic and Antiemetic Drugs**	**Antipsychotics**	**Antidepressants**
Known risk	Amiodarone<br>Disopyramide<br>Dofetilide<br>Dronedarone<br>Flecainide<br>Ibutilide<br>Procainamide<br>Quinidine<br>Sotalol	Moxifloxacin<br>Levofloxacin<br>Ciprofloxacin<br>Clarithromycin<br>Erythromycin<br>Azithromycin<br>Fluconazole<br>Pentamidine	Domperidone<br>Chlorpromazine<br>Ondansetron<br>Droperidol	Haloperidol<br>Mesoridazine<br>Thioridazine<br>Pimozide	Escitalopram<br>Citalopram
Possible risk	None	Telavancin<br>Telithromycin<br>Gemifloxacin<br>Norfloxacin<br>Ofloxacin	Dolasetron<br>Granisetron<br>Promethazine<br>Tropisetron	Lithium<br>Clozapine<br>Paliperidone<br>Risperidone<br>Promethazine<br>Perphenazine<br>Pimavanserin<br>Iloperidone<br>Aripiprazole<br>Asenapine	Clomipramine<br>Desipramine<br>Imipramine<br>Mirtazapine<br>Nortriptyline<br>Trimipramine<br>Venlafaxine

Table 50: Concomitant Medications Associated With Risk of QTc Interval Prolongation and/or Torsades de Pointes

Risk	Drug Categories				
	**Antiarrhythmic Drugs**	**Common Antibacterial and Antifungal Drugs**	**Prokinetic and Antiemetic Drugs**	**Antipsychotics**	**Antidepressants**
Conditional risk	Ivabradine	Amphotericin B Itraconazole<br>Ketoconazole<br>Metronidazole<br>Posaconazole<br>Voriconazole<br>Cotrimoxazole (avoid in congenital long QT syndrome)	Metoclopramide	Quetiapine<br>Olanzapine<br>Ziprasidone	Amitriptyline<br>Doxepin<br>Fluoxetine<br>Fluvoxamine<br>Paroxetine<br>Sertraline<br>Trazodone
Alternatives	None	Penicillin<br>Cephalosporins<br>Doxycycline<br>Anidulafungin	Aprepitant<br>Fosaprepitant<br>Palonosetron	Brexpiprazole	Desvenlafaxine<br>Bupropion (except in supratherapeutic dose)<br>Vortioxetine<br>Vilazodone<br>Levomilnacipran<br>Milnacipran

Notes: Known risk of Torsades de Pointes (TdP) - These drugs prolong the QT interval and are clearly associated with a known risk of TdP, even when taken as recommended.
Possible risk of TdP - These drugs can cause QT prolongation but lack evidence for a risk of TdP when taken as recommended.
Conditional risk of TdP - These drugs could cause TdP only under certain conditions, such as excessive dosing, electrolyte imbalance, and interacting with other drugs that can cause TdP.
Alternatives - Drugs that at this point have not been linked to clinically significant QTc prolongation.
See http://crediblemeds.org for an exhaustive list.
Ondansetron is only prohibited if administered as a single intravenous dose of > 16 mg. Oral administration is allowed.
Reference: Porta-Sánchez et al. 2017.

10.8. Appendix 8: International Metastatic Renal Cell Carcinoma Database Consortium (IMDC) Criteria

The IMDC Criteria was developed as a prognostic tool for patients with metastatic renal cell carcinoma (mRCC) who receive systemic therapy (International Metastatic Renal Cell Carcinoma Database Consortium 2024). The IMDC risk factors have been externally validated in the first-, second-, third-, fourth-line settings, with the use of tyrosine kinase inhibitors and nivolumab, and with non-clear cell renal cell carcinomas in the first-line setting. Risk classification into favorable-, intermediate-, or poor-risk groups is based on the IMDC risk factors described below.

**International Metastatic Renal Cell Carcinoma Database Consortium (IMDC) Criteria**	
**Risk Factors**	**Value**
Karnofsky Performance Status	< 80%
Time from any initial diagnosis (including localized renal cell carcinoma) to when first systemic therapy is started for metastatic disease	< 1 year
Hemoglobin	< Lower limit of normal
Neutrophils	> Upper limit of normal
Platelets	> Upper limit of normal
Corrected Calcium	> Upper limit of normal
**Risk Profile**	**Score**
Favorable	0 factors
Intermediate	1-2 factors
Poor	3+ factors

Note: Because normal ranges vary between laboratories, limits of normal set by the laboratory performing the tests will be used.
Reference: International Metastatic Renal Cell Carcinoma Database Consortium.

11. REFERENCES

Agarwal N, Brugarolas J, Ghatalia P, et al. Randomized phase II dose comparison LITESPARK-013 study of belzutifan in patients with advanced clear cell renal cell carcinoma. Ann Oncol. 2024;35(12):1148-1156. doi:10.1016/j.annonc.2024.08.2338.

Albiges L, Choueiri TK, Peltola K, et al. 42 Belzutifan versus everolimus for previously treated advanced clear cell renal cell carcinoma: subgroup analysis of the phase 3 LITESPARK-005 study. Oncologist. 2024;29(suppl 1):S3–S4. doi:10.1093/oncolo/oyae181.005.

American Thoracic Society (ATS) Committee on Proficiency Standards for Clinical Pulmonary Function Laboratories. ATS statement: guidelines for the six-minute walk test. Am J Respir Crit Care Med. 2022;166(1):111–117. doi:10.1164/ajrccm.166.1.at1102.

Atkins MB, Ghatalia P, Merchan JR, et al. Belzutifan in patients with advanced clear cell renal cell carcinoma (ccRCC): subgroup analysis of the phase 2 LITESPARK-013 study. J Clin Oncol. 2024;42(16 suppl):abstract 4534. doi:10.1200/JCO.2024.42.16_suppl.4534.

CABOMETYX (cabozantinib) US prescribing information. Exelixis, Inc ; 2025.

Castellano D, Pablo Maroto J, Benzaghou F, et al. Exposure-response modeling of cabozantinib in patients with renal cell carcinoma: implications for patient care. Cancer Treat Rev. 2020;89:102062. doi:10.1016/j.ctrv.2020.102062.

Centers for Disease Control and Prevention/National Center for Health Statistics. National Health and Nutrition Examination Survey (NHANES) questionnaires, datasets, and related documentation. Centers for Disease Control and Prevention. https://wwwn.cdc.gov/nchs/nhanes/.

Chiu DK-C, Zhang X, Cheng BY-L, et al. Tumor-derived erythropoietin acts as an immunosuppressive switch in cancer immunity. Science. 2025; 388(6745):eadr3026. doi:10.1126/science.adr3026.

Choueiri TK, Escudier B, Powles T, et al; METEOR Investigators. Cabozantinib versus everolimus in advanced renal-cell carcinoma. N Engl J Med. 2015;373(19):1814-1823. doi:10.1056/NEJMoa1510016.

Choueiri TK, Motzer RJ. Systemic therapy for metastatic renal-cell carcinoma. N Engl J Med. 2017;376(4):354-366. doi:10.1056/NEJMra1601333.

Choueiri TK, Hessel C, Halabi S, et al. Cabozantinib versus sunitinib as initial therapy for metastatic renal cell carcinoma of intermediate or poor risk (Alliance A031203 CABOSUN randomised trial): progression-free survival by independent review and overall survival update [published correction appears in Eur J Cancer. 2018;103:287]. Eur J Cancer. 2018;94:115-125. doi:10.1016/j.ejca.2018.02.012.

Choueiri TK, Bauer TM, Papadopoulos KP, et al. Inhibition of hypoxia-inducible factor-2α in renal cell carcinoma with belzutifan: a phase 1 trial and biomarker analysis. Nat Med. 2021a;27(5):802-805. doi:10.1038/s41591-021-01324-7.

Choueiri TK, Powles T, Burotto M, et al; CheckMate 9ER Investigators. Nivolumab plus cabozantinib versus sunitinib for advanced renal-cell carcinoma. N Engl J Med. 2021b;384(9):829-841. doi:10.1056/NEJMoa2026982.

Choueiri TK, McDermott DF, Merchan J, et al. Belzutifan plus cabozantinib for patients with advanced clear cell renal cell carcinoma previously treated with immunotherapy: an open-label, single-arm, phase 2 study. Lancet Oncol. 2023a;24(5):553-562. doi:10.1016/S1470-2045(23)00097-9.

Choueiri TK, Powles T, Albiges L, et al. Cabozantinib plus nivolumab and ipilimumab in renal-cell carcinoma. Comment Reply. N Engl J Med. 2023b;389(5):477-478. doi:10.1056/NEJMc2306786.

Choueiri TK, Powles T, Peltola K, et al. LITESPARK-005 Investigators. Belzutifan versus everolimus for advanced renal-cell carcinoma. N Engl J Med. 2024a;391(8):710-721. doi:10.1056/NEJMoa2313906.

Choueiri TK, Tomczak P, Park SH, et al. KEYNOTE-564 Investigators. Overall survival with adjuvant pembrolizumab in renal-cell carcinoma. N Engl J Med. 2024b;390(15):1359-1371. doi:10.1056/NEJMoa2312695.

Choueiri TK, Escudier B, McDermott DF, et al. Nivolumab plus ipilimumab vs sunitinib for first-line treatment of advanced renal cell carcinoma: Final analysis from the phase 3 CheckMate 214 trial. J Clin Oncol. 2025;43:4505. doi: 10.1200/JCO.2025.43.16_suppl.4505.

Cockcroft DW, Gault MH. Prediction of creatinine clearance from serum creatinine. Nephron. 1976;16(1):31-41. doi:10.1159/000180580.

Cybulska-Stopa B, Piejko K, Ostaszewski K, et al. Long-term clinical evidence of comparable efficacy and toxicity of nivolumab and pembrolizumab in advanced melanoma treatment. Melanoma Res. 2023;33(3):208-217. doi:0.1097/CMR.0000000000000885.

Eisenhauer EA, Therasse P, Bogaerts J, et al. New response evaluation criteria in solid tumours: revised RECIST guideline (version 1.1). Eur J Cancer. 2009;45(2):228-247. doi:10.1016/j.ejca.2008.10.026.

Hsieh JJ, Purdue MP, Signoretti S, et al. Renal cell carcinoma. Nat Rev Dis Primers. 2017;3:17009. doi:10.1038/nrdp.2017.9.

International Metastatic Renal Cell Carcinoma Database Consortium. IMDC Risk Calculator. Accessed 2024. https://www.imdconline.com/

Jonasch E, Donskov F, Iliopoulos O, et al; MK-6482-004 Investigators. Belzutifan for renal cell carcinoma in von Hippel-Lindau disease. N Engl J Med. 2021;385(22):2036-2046. doi:10.1056/NEJMoa2103425.

Kondo K, Klco J, Nakamura E, Lechpammer M, Kaelin WG Jr. Inhibition of HIF is necessary for tumor suppression by the von Hippel-Lindau protein. Cancer Cell. 2002;1(3):237–246. doi:10.1016/s1535-6108(02)00043-0.

Kondo K, Kim WY, Lechpammer M, Kaelin WG Jr. Inhibition of HIF2alpha is sufficient to suppress pVHL-defective tumor growth. PLoS Biol. 2003;1(3):E83. doi:10.1371/journal.pbio.0000083.

Lee D, Gittleman H, Weinstock C, et al. A U.S. Food and Drug Administration-pooled analysis of frontline combination treatment survival benefits by risk groups in metastatic renal cell carcinoma. Eur Urol. 2023;84(4):373-378. doi:10.1016/j.eururo.2023.05.030.

Miikkulainen P, Högel H, Seyednasrollah F, Rantanen K, Elo LL, Jaakkola PM. Hypoxia-inducible factor (HIF)-prolyl hydroxylase 3 (PHD_3) maintains high *HIF2A* mRNA levels in clear cell renal cell carcinoma. J Biol Chem. 2019;294(10):3760-3771. doi:10.1074/jbc.RA118.004902.

Motzer RJ, Escudier B, McDermott DF, et al; CheckMate 025 Investigators. Nivolumab versus everolimus in advanced renal-cell carcinoma. N Engl J Med. 2015a;373(19):1803-1813. doi:10.1056/nejmoa1510665.

Motzer RJ, Rini BI, McDermott DF, et al. Nivolumab for metastatic renal cell carcinoma: results of a randomized phase II trial. J Clin Oncol. 2015b;33(13):1430-1437. doi:10.1200/JCO.2014.59.0703.

Motzer RJ, Tannir NM, McDermott DF, et al; CheckMate 214 Investigators. Nivolumab plus ipilimumab versus sunitinib in advanced renal-cell carcinoma. N Engl J Med. 2018;378(14):1277-1290. doi:10.1056/nejmoa1712126.

Motzer RJ, Penkov K, Haanen J, et al. Avelumab plus axitinib versus sunitinib for advanced renal-cell carcinoma. N Engl J Med. 2019;380(12):1103-1115. doi:10.1056/NEJMoa1816047.

Motzer RJ, Escudier B, George S, et al. Nivolumab versus everolimus in patients with advanced renal cell carcinoma: updated results with long-term follow-up of the randomized, open-label, phase 3 CheckMate 025 trial. Cancer. 2020;126(18):4156-4167. doi:10.1002/cncr.33033.

Motzer R, Alekseev B, Rha SY, et al; CLEAR Trial Investigators. Lenvatinib plus pembrolizumab or everolimus for advanced renal cell carcinoma. N Engl J Med. 2021;384(14):1289-1300. doi:10.1056/NEJMoa2035716.

Motzer RJ, Porta C, Eto M, et al. Lenvatinib plus pembrolizumab versus sunitinib in first-line treatment of advanced renal cell carcinoma: final prespecified overall survival analysis of CLEAR, a Phase III study. J Clin Oncol. 2024;42(11):1222-1228. doi:10.1200/JCO.23.01569.

National Comprehensive Cancer Network (NCCN). NCCN Clinical Practice Guidelines in Oncology (NCCN Guidelines®) – Kidney Cancer. Version 2.2024; September 6, 2024. Accessed 2024. NCCN.org.

OPDIVO (nivolumab) injection US prescribing information. Bristol-Myers Squibb Company; 2025.

Padala SA, Barsouk A, Thandra KC, et al. Epidemiology of renal cell carcinoma. World J Oncol. 2020;11(3):79-87. doi:10.14740/wjon1279.

Pal SK, Albiges L, Tomczak P, et al. Atezolizumab plus cabozantinib versus cabozantinib monotherapy for patients with renal cell carcinoma after progression with previous immune checkpoint inhibitor treatment (CONTACT-03): a multicentre, randomised, open-label, phase 3 trial. Lancet. 2023;402(10397):185-195. doi:10.1016/S0140-6736(23)00922-4.

Porta-Sánchez A, Gilbert C, Spears D, et al. Prolongation induced by cancer therapies: a systematic review. J Amer Heart Assoc. 2017;6:e007724. doi:10.1161/JAHA.117.007724.

Rini BI, Hutson TE, Figlin RA, et al. Sunitinib in patients with metastatic renal cell carcinoma: clinical outcome according to International Metastatic Renal Cell Carcinoma Database

Consortium Risk Group. Clin Genitourin Cancer. 2018;16(4):298-304. doi:10.1016/j.clgc.2018.04.005.

Rini BI, Plimack ER, Stus V, et al; KEYNOTE-426 Investigators. Pembrolizumab plus axitinib versus sunitinib for advanced renal-cell carcinoma. N Engl J Med. 2019;380(12):1116-1127. doi:10.1056/NEJMoa1816714.

Rini BI, Brugarolas J, Atkins MB. Navigating and adapting care integrating immunotherapy, antiangiogenic therapy, and combinations in patients with advanced renal cell carcinoma. J Immunother Cancer. 2023;11(3):e006361. doi:10.1136/jitc-2022-006361.

Rini BI, Rodriguez CS, Albiges L, et al. LBA74 Final analysis of the phase III LITESPARK-005 study of belzutifan versus everolimus in participants (pts) with previously treated advanced clear cell renal cell carcinoma (ccRCC). Ann Oncol. 2024;35(suppl 2):S1262-S1263. doi:10.1016/j.annonc.2024.08.2317.

Sato Y, Yoshizato T, Shiraishi Y, et al. Integrated molecular analysis of clear-cell renal cell carcinoma. Nat Genet. 2013;45(8):860-867. doi:10.1038/ng.2699.

Siegel RL, Miller KD, Fuchs HE, Jemal A. Cancer statistics, 2021. CA Cancer J Clin. 2021;71(1):7-33. doi:10.3322/caac.21654.

Tannir NM, Agarwal N, Porta C, et al. Efficacy and safety of telaglenastat plus cabozantinib vs placebo plus cabozantinib in patients with advanced renal cell carcinoma: the CANTATA randomized clinical trial. JAMA Oncol. 2022;8(10):1411-1418. doi:10.1001/jamaoncol.2022.3511.

Tannir NM, Albigès L, McDermott DF, et al. Nivolumab plus ipilimumab versus sunitinib for first-line treatment of advanced renal cell carcinoma: extended 8-year follow-up results of efficacy and safety from the phase III CheckMate 214 trial. Ann Oncol. 2024;35(11):1026-1038. doi:10.1016/j.annonc.2024.07.727.

Tenold M, Ravi P, Kumar M, et al. Current approaches to the treatment of advanced or metastatic renal cell carcinoma. Am Soc Clin Oncol Educ Book. 2020;40:1-10. doi:10.1200/EDBK_279881.

US Food & Drug Administration (FDA). Clinical Considerations for Therapeutic Cancer Vaccines: Guidance for Industry. October 2011. Accessed 2022. https://www.fda.gov/regulatory-information/search-fda-guidance-documents/clinical-considerations-therapeutic-cancer-vaccines.

US Food & Drug Administration (FDA). Collection of Race and Ethnicity Data in Clinical Trials: Guidance for Industry and Food and Drug Administration Staff. October 26, 2016. Accessed 2022. https://www.fda.gov/regulatory-information/search-fda-guidance-documents/collection-race-and-ethnicity-data-clinical-trials.

WELIREG (belzutifan) US prescribing information. Merck Sharp & Dohme LLC; 2025.

Yang G, Shi R, Zhang Q. Hypoxia and oxygen-sensing signaling in gene regulation and cancer progression. Int J Mol Sci. 2020;21(21):8162. doi:10.3390/ijms21218162.

YERVOY (ipilimumab) US prescribing information. Bristol-Myers Squibb Company; 2025.

Yu Y, Yu Q, Zhang X. Allosteric inhibition of HIF-2α as a novel therapy for clear cell renal cell carcinoma. Drug Discov Today. 2019;24(12):2332-2340. doi:10.1016/j.drudis.2019.09.008.

Zarba M, Ferrier E, Wells C, et al. Systemic treatments in favorable and very favorable risk metastatic renal cell carcinoma (mRCC): real world evidence from the International mRCC Database Consortium (IMDC). J Clin Oncol. 2024;42(16 suppl):abstract 4514. doi:10.1200/JCO.2024.42.

Zhao X, Suryawanshi S, Hruska M, et al. Assessment of nivolumab benefit–risk profile of a 240-mg flat dose relative to a 3-mg/kg dosing regimen in patients with advanced tumors. Ann Oncol. 2017;28(8):2002-2008. doi:10.1093/annonc/mdx235.

Protocol ARC-20 Version 11.0 (Amendment 10)

Signature Page for VV-CLIN-000895 v14.0

Approval with eSig	PPD<br>|23-Dec-2025 02:39:01 GMT+0000

Signature Page for VV-CLIN-000895 v14.0
