# Supplementary material for: Casdatifan shows durable response linked to HIF-2α biology in kidney cancer
Source: Nature. 2026 Jul 1;656(8128):734–41. doi: 10.1038/s41586-026-10718-x (PMC13489952; doi:10.1038/s41586-026-10718-x)
Supplement: Supplementary file 2 [file 41586_2026_10718_MOESM2_ESM.pdf]

nature portfolio

Corresponding author(s): Toni K. Choueiri, MD

Last updated by author(s): May 6, 2026

Reporting Summary

Nature Portfolio wishes to improve the reproducibility of the work that we publish. This form provides structure for consistency and transparency in reporting. For further information on Nature Portfolio policies, see our Editorial Policies and the Editorial Policy Checklist.

Statistics

For all statistical analyses, confirm that the following items are present in the figure legend, table legend, main text, or Methods section.

n/a	Confirmed	
☐	☒	The exact sample size (*n*) for each experimental group/condition, given as a discrete number and unit of measurement
☐	☒	A statement on whether measurements were taken from distinct samples or whether the same sample was measured repeatedly
☐	☒	The statistical test(s) used AND whether they are one- or two-sided<br>*Only common tests should be described solely by name; describe more complex techniques in the Methods section.*
☐	☒	A description of all covariates tested
☐	☒	A description of any assumptions or corrections, such as tests of normality and adjustment for multiple comparisons
☐	☒	A full description of the statistical parameters including central tendency (e.g. means) or other basic estimates (e.g. regression coefficient) AND variation (e.g. standard deviation) or associated estimates of uncertainty (e.g. confidence intervals)
☐	☒	For null hypothesis testing, the test statistic (e.g. *F*, *t*, *r*) with confidence intervals, effect sizes, degrees of freedom and *P* value noted<br>*Give P values as exact values whenever suitable.*
☒	☐	For Bayesian analysis, information on the choice of priors and Markov chain Monte Carlo settings
☒	☐	For hierarchical and complex designs, identification of the appropriate level for tests and full reporting of outcomes
☒	☐	Estimates of effect sizes (e.g. Cohen's *d*, Pearson's *r*), indicating how they were calculated

Our web collection on statistics for biologists contains articles on many of the points above.

Software and code

Policy information about availability of computer code

Data collection	Clinical data were collected in a multicenter clinical trial conducted at academic hospitals and specialized cancer centers in the United States, Australia, and South Korea. Clinical data were captured at each protocol defined study visit as well as unscheduled visits and recorded in electronic case report forms (eCRFs).
Data analysis	SAS v9.4 was used for clinical data analyses.<br>For exploratory biomarker analyses, RNA-seq data quality control was performed using FASTQC v0.11.9. Reads were aligned to GRCh38 human reference genome (Ensembl v104) using the STAR aligner v2.6.1d, and quantification was performed using salmon v1.4.0 with GENCODE v38 annotations. Limma v3.64.1 with voom preprocessing was used for differential gene expression analysis. GSEA was performed on the ranking of t-statistics from the differential gene expression analyses using the Fast GSEA package v1.34.0. Gene set scores were calculated using ssGSEA implemented in the Gene Set Variation Analysis (GSVA) package v2.4.7. Raw data was processed using the nf-core sarek workflow v3.4.2 and additional custom Nextflow pipelines. Reads from FASTQ files were trimmed and split using fastP v0.23.4, and quality control was performed using MultiQC v1.25.1. Trimmed reads were aligned to GRCh38 using bwa-mem v0.7.17.post1188. Somatic variants were identified using three different variant callers: Mutect2 v4.5.0.0, Strelka v2.9.10 and VarScan v2.4.4. Variants were annotated using VEP v108. Clinical association of various biomarkers were analyzed using R v4.3.1. Kaplan-Meier survival analyses for HIF-2α IHC staining ≥25% or <25% were conducted with log-rank P values calculated using the survival v3.8-3 and survminer v0.5.0 R packages. Visualization of the differences between defined groups was performed using ggplot2 v4.0.2 and base R plotting functions.

For manuscripts utilizing custom algorithms or software that are central to the research but not yet described in published literature, software must be made available to editors and reviewers. We strongly encourage code deposition in a community repository (e.g. GitHub). See the Nature Portfolio guidelines for submitting code & software for further information.

Data

Policy information about availability of data

All manuscripts must include a data availability statement. This statement should provide the following information, where applicable:

- Accession codes, unique identifiers, or web links for publicly available datasets
- A description of any restrictions on data availability
- For clinical datasets or third party data, please ensure that the statement adheres to our policy

Arcus Biosciences is committed to sharing clinical trial data with external qualified scientific researchers in the interest of advancing public health. Arcus will provide access to individual de-identified participant data and related study documents (protocols, statistical analysis plans and clinical study reports) upon request from qualified researchers, and subject to certain criteria, conditions, and exceptions. For information on the process or to submit a request, visit https://trials.arcusbio.com/our-transparency-policy. The GRCh38 human reference genome is available from genome.usc.edu. RNA expression and somatic mutation data from IMmotion151 were generated by Genentech/gRED and used under agreement with Genentech from the European Genome Archive: https://ega-archive.org/studies/EGAS00001004353. Expression data from the Javelin101 study are publicly available (Motzer et al., Nature Med, 2020).
The custom codes used in this study contain confidential information and are therefore not publicly available. Code may be made available upon reasonable request, subject to appropriate agreements. For information on the process or to submit a request, visit https://trials.arcusbio.com/our-transparency-policy.

Research involving human participants, their data, or biological material

Policy information about studies with human participants or human data. See also policy information about sex, gender (identity/presentation), and sexual orientation and race, ethnicity and racism.

Reporting on sex and gender	Sex was recorded as a binary variable based on self-reported biological characteristics; gender identity was not collected. Analyses were not stratified by sex. Patient sex (female and male) is summarized in Table 1 and Extended Data Table 1.
Reporting on race, ethnicity, or other socially relevant groupings	Race and/or ethnicity were classified as reported by the patient.
Population characteristics	Patients were aged ≥ 18 years with histologically confirmed metastatic ccRCC, received prior treatment in the locally advanced or metastatic setting with an anti–PD-(L)1 inhibitor and a VEGFR-TKI, were HIF-2α inhibitor naive, had a creatinine clearance of ≥40 mL/min and had ≥1 measurable tumor lesion per RECIST v1.1. Patients must also have had an ECOG performance status of 0 or 1 and adequate organ and marrow function. Complete eligibility criteria are described in Supplementary Tables 1 and 2.
Recruitment	The ARC-20 study was conducted at academic hospitals and specialized cancer centers in the United States, Australia and the South Korea. Participants were recruited by investigators at participating study sites. Recruitment bias is not expected to be greater than that observed in clinical trials of similar phase and size and is not anticipated to materially affect study results. This study may be subject to self-selection bias, as participation was limited to patients who met protocol defined eligibility criteria and consented to enrollment in a clinical trial setting. As such, the study population may not fully represent the broader population of patients with kidney cancer encountered in routine clinical practice.<br>Additional sources of potential bias include the single-arm, early-phase nature of the trial. Without a randomized comparator, estimates of antitumor efficacy may be influenced by unmeasured confounders, including variable disease trajectories, differences in prior treatment responsiveness and the natural heterogeneity of refractory ccRCC. Biomarker analyses were exploratory and conducted in subsets of patients with available samples, which may introduce selection bias and limit generalizability. These factors were considered when interpreting the results.
Ethics oversight	Before the study was initiated, an institutional review board or independent ethics committee at each study site approved the protocol and other relevant study materials. A central institutional review board supported multiple sites. A full list of ethics committee names is provided below.<br>All patients or their legally authorized representatives provided written informed consent before enrollment; patients were not compensated monetarily for their participation in this trial. This study was conducted in full accordance with the Declaration of Helsinki, Council for International Organizations of Medical Sciences, the International Council for Harmonisation and all regional laws and regulations.<br><br>Ethics committees:<br>Macquarie University Human Research Ethics Committee<br>Bellberry Human Resource Ethics Committee<br>Eastern Health Office of Research and Ethics<br>Mount Sinai School of Medicine IRB #1 - Board B<br>Salus IRB<br>Advarra<br>The University of Louisville Institutional Review Board<br>Castle IRB<br>Dana-Farber Cancer Institute Institutional Review Board<br>Vanderbilt University Medical Center<br>Ochsner Clinic Foundation Institutional Review Board<br>University of Miami Institutional Review Board<br>The UCLA Institutional Review Board<br>Johns Hopkins Medicine Institutional Review Board

Cleveland Clinic Institutional Review Board
University Hospitals Cleveland Medical Center
Memorial Sloan Kettering Cancer Center IRB
Drug Research Ethics Committee of HM Hospitales
IRB of Samsung Medical Center
Seoul National University Bundang Hospital Institutional Review Board
IRB of Asan Medical Center
Yonsei University Health System, Severance Hospital Institutional Review Board

Note that full information on the approval of the study protocol must also be provided in the manuscript.

Field-specific reporting

Please select the one below that is the best fit for your research. If you are not sure, read the appropriate sections before making your selection.

☒ Life sciences ☐ Behavioural & social sciences ☐ Ecological, evolutionary & environmental sciences

For a reference copy of the document with all sections, see nature.com/documents/nr-reporting-summary-flat.pdf

Life sciences study design

All studies must disclose on these points even when the disclosure is negative.

Sample size	The dose-escalation sample size was determined using a rule-based 3 + 3 escalation method targeting a DLT threshold of 33%; 25 patients were enrolled. The planned sample size for dose expansion was approximately 30 patients per cohort, which was selected to obtain a preliminary assessment of antitumor activity based on the ORR. With this sample size, the lower bound of the Clopper-Pearson 90% CI would be expected to exclude historical control response rates with differences on the order of 15% to 20%. Across cohorts, a total of 127 patients were enrolled in the dose-expansion stage.
Data exclusions	Inclusion/exclusion criteria for the ARC-20 study are described in the methods and in Supplementary Tables 2 and 3. No patients were excluded from the safety analyses. Patients were excluded from the efficacy analyses (n=6) if they did not meet the following prespecified criteria: received ≥1 dose of study treatment and either had ≥1 post-baseline efficacy assessment or discontinued study treatment due to progressive disease or death. Patients with major protocol deviations which may impact interpretation of efficacy endpoints were also excluded from the efficacy analyses. sEPO data collected within 28 days post EPO-stimulating agents and within 4 days after a dose hold or dose reduction of less than half the intended dose were excluded from data analysis.
Replication	No replication was performed as this was a clinical trial.
Randomization	This study was not a randomized trial.
Blinding	This study was an open-label trial; therefore, patients, the sponsor, and the investigator were not blinded to the study treatment administered.<br>Covariates were not formally controlled for in the ARC-20 study because it is an early phase, single arm clinical trial primarily designed to evaluate safety, tolerability, and preliminary clinical activity of casdatifan in patients with kidney cancer.<br><br>In the exploratory biomarkers analyses, univariable tests were performed and reported for all analyses with the exception of the Cox regression modeling that was performed to assess the relationship between tEPO RNAseq and PFS. For this analysis, sex, age (as a categorical variable; 18–64 years versus ≥65 years), IMDC risk (intermediate/poor vs favorable) and ECOG categories (0 versus 1) were used as demographic covariables during modeling.

Reporting for specific materials, systems and methods

We require information from authors about some types of materials, experimental systems and methods used in many studies. Here, indicate whether each material, system or method listed is relevant to your study. If you are not sure if a list item applies to your research, read the appropriate section before selecting a response.

Materials & experimental systems

n/a	Involved in the study	
☐	☒	Antibodies
☒	☐	Eukaryotic cell lines
☒	☐	Palaeontology and archaeology
☒	☐	Animals and other organisms
☐	☒	Clinical data
☒	☐	Dual use research of concern
☒	☐	Plants

Methods

n/a	Involved in the study	
☒	☐	ChIP-seq
☒	☐	Flow cytometry
☒	☐	MRI-based neuroimaging

Antibodies

Antibodies used	Rabbit anti-human HIF-2α antibody (1:100; Cat#59973, Cell Signaling Technologies, Boston MA); isotype antibody (1:400, Cat#3900S, Cell Signaling Technologies)
Validation	For the HIF-2α IHC assay, antibody specificity was validated using HIF-2α–engineered cell lines, Western blotting, peptide competition assays, and a multi-organ normal human tissue array. According to the manufacturer, the rabbit (DA1E) monoclonal antibody IgG isotype control is not directed against any known antigen. This antibody functions as an isotype control for rabbit IgG antibodies. This validation is supported by the relevant citations on the manufacturer's website, https://www.cellsignal.com/products/primary-antibodies/rabbit-da1e-monoclonal-antibody-igg-isotype-control/3900.

Clinical data

Policy information about clinical studies

All manuscripts should comply with the ICMJE guidelines for publication of clinical research and a completed CONSORT checklist must be included with all submissions.

Clinical trial registration	NCT05536141
Study protocol	The protocol is provided as a supplementary file.
Data collection	The ARC-20 study was registered on September 7, 2022, with clinicaltrials.gov. Data were collected in a multicenter clinical trial conducted at academic hospitals and specialized cancer centers in the United States, Australia and South Korea. Clinical data were collected by investigators and recorded in eCRFs. The data cutoff date is August 15, 2025.
Outcomes	The primary outcome of safety and tolerability was assessed through ongoing investigator evaluation of treatment emergent adverse events, including type, incidence, severity, and seriousness, graded according to the National Cancer Institute Common Terminology Criteria for Adverse Events (CTCAE) version 5.0. Safety assessments were conducted continuously from the first dose of study treatment until 30 days after the last dose or initiation of new systemic anticancer therapy, whichever occurred first. Adverse events were coded using the Medical Dictionary for Regulatory Activities (MedDRA), version 25.0. The secondary outcome of clinical activity was assessed by investigator determined objective response rate according to RECIST v1.1, based on radiologic tumor assessments performed at protocol specified time points. Exploratory outcomes included progression free survival, assessed according to RECIST v1.1, and biomarker analyses, which were conducted in available patient samples.

Plants

Seed stocks	n/a
Novel plant genotypes	n/a
Authentication	n/a
