# Supplementary material for: Casdatifan shows durable response linked to HIF-2α biology in kidney cancer
Source: Nature. 2026 Jul 1;656(8128):734–41. doi: 10.1038/s41586-026-10718-x (PMC13489952; doi:10.1038/s41586-026-10718-x)
Supplement: Supplementary file 3 [file 41586_2026_10718_MOESM3_ESM.pdf]

nature portfolio

Peer Review File

Casdatifan shows durable response linked to HIF-2α biology in kidney cancer

Corresponding Author: Professor Toni Choueiri

Any redactions in this file are there to maintain patient confidentiality, the confidentiality of unpublished data, or to remove third-party material.

This file contains all reviewer reports in order by version, followed by all author rebuttals in order by version.

Version 0:

Reviewer comments:

Referee #1

(Remarks to the Author)
Choueiri et al provide data for casdatifan, reporting (to this reviewer's knowledge) the highest RR seen to date with a HIF2 inhibitor and also the first putative biomarker (HIF2 RNA) of response. This is a highly significant paper to the field. The following comments are offered for the authors consideration.

Major Comments:

1. In Table 1, would offer more substantial breakdown of prior systemic therapy in the dose expansion phase.

Minor Comments:

1. The response rate observed in this study is significantly higher than that observed with belzutifan (35%; versus a ~20% response rate with belzutifan). Can the authors speculate as to why the response rate seen is significantly higher, given that both function via allosteric binding? There is brief comment around PK parameters but more rationale could be provided.
2. In Figure 2D, can the authors provide scaling for the provided micrographs?
3. In Figure 2F, can the authors provide rationale for the 25% cutoff used for HIF-2a staining? Was this derived from recursive partitioning?
4. In Figure 4C, it is unclear what the Forrest plots are articulating in terms of relative benefit. Would consider removing.

Referee #2

(Remarks to the Author)
This is a well written, clinically significant publication exploring the safety, activity and pharmacodynamic biomarkers of the dose escalation and dose expansion cohorts of the ARC-20 phase 1 study of casdatifan.
The authors do a great job summarizing the patient population, safety results and efficacy results of both cohorts. The inclusion of which cases of anemia led to blood transfusions and the use of erythropoietin-stimulating products and how many cases of hypoxia led to the need of oxygen supplementation as well as the time to recovery for hypoxia is especially commendable.
There is also a good amount of thought to the pharmacokinetics of casdatifan and the authors explore the relationship between casdatifan dose and response, showing that there is no relationship between pk and resistance to casdatifan.
The hypothesis of the study hinges on the ability of casdatifan to achieve a superior shutdown of HIF2A to belzutifan. To prove this point the authors focus in one key PD biomarker, sEPO, that it is reported to be inhibited only by 70% by belzutifan due to saturable absorption. Reduction of sEPO in this cohort is >80% for all tested doses and there was a higher likelihood of response in patients that achieve more profound reductions in sEPO.
The authors show how patients with high baseline levels of tumor EPO expression had higher EPO reduction as the consequence of casdatifan and that EPO was being produced in the cancer cells and not in stromal cells, according to what is expected if driven by HIF2A overexpression. Then they showed correlation between EPO and HIF staining.

Our conclusion is that this is a really strong paper, and we want to recommend mostly minor changes/aclarations

Major comments:
Reduction of sEPO in different doses of casdatifan is not reported. How does the RP3D compare with other doses in terms of sEPO reduction. Is there a dose-relationship?
The authors analyze the correlation between VHL mutation and HIF2/EPO, and find no correlation but VHL function can be lost due to biallelic loss without any mutation being present. A further analysis testing the correlation of biallelic VHL loss + inactivating mutations with HIF2/EPO is recommended.
Presence of a (OxPhos) signature is correlated with higher resistance to casdatifan, while this might represent patients in which HIF2 is not overexpressed, there is evidence that the lack of VHL can result in abnormal expression of HF1A in patients with ccRC and this can mediate resistance to immunotherapy. We suggest an analysis of the potential role of HIF1A as resistance mechanism to casdatifan.

https://jitc.bmj.com/content/12/2/e008226

Minor comments:
Treatment with belzutifan was an exclusion cqriteria in the expansion cohorts, but authors should clarify if any patient in the escalation phase received previous HIF2 inhibition.
The inclusion of a CONSORT-DEFINE checklist would have helped review the completion of all the recommended elements.
The authors could have discussed if a dose-escalation strategy focused on optimizing the reduction of sEPO could be a beneficial strategy to optimize tumor responses.

Presence of a (OxPhos) signature is correlated with higher resistance to casdatifan, while this might represent patients in which HIF2 is not overexpressed, there is evidence that the lack of VHL can result in abnormal expression of HF1A in patients with ccRCC and this can mediate resistance to immunotherapy. We suggest a analysis of the potential role of HIF1A as resistance mechanism to casdatifan.

https://jitc.bmj.com/content/12/2/e008226

There is a subset of patients with ccRC that develop secondary inactivating mutations in HIF1A, while this results in a worse prognosis, they would also be the ones in wich the HIF2A signature would be more clear and could potentially explain the trend towards a more profound benefit of casdatifan in patients with worse IMDC score.

https://www.nature.com/articles/s41467-020-17873-3

Referee #3

(Remarks to the Author)
I co-reviewed this manuscript with one of the reviewers who provided the listed reports.

Referee #4

(Remarks to the Author)
Choueiri et al have compiled the results of the ARC-20 phase 2 trial of casdatifan in refractory clear cell RCC. This is an impactful study demonstrating that casdatifan produces a deep on-target effect with encouraging efficacy in a heavily pretreated population enriched for intermediate- and poor-risk RCC. Antitumor activity (ORR 31–35%, DCR 81–84%) appears clinically meaningful and responses appear durable. Toxicities were consistent with the expected class effects of HIF-2α inhibition and overall manageable, with no treatment-related deaths reported, although anemia and hypoxia remain clinically significant.

There is encouraging biomarker analysis of erythropoietin reduction correlating with clinical benefit, with higher baseline tumor EPO mRNA expression associated with response. Transcriptomic GSEA further demonstrates that a HIF-2α gene signature is associated with longer PFS with casdatifan but shorter PFS with non–HIF-2α regimens, supporting HIF-2α inhibition as a rational therapeutic strategy in biomarker-selected patients.

Overall the analysis was comprehensive and manuscript is well written, the figures are clear, and the data is well presented.

Several considerations to strengthen the manuscript:
- In discussion section, consider expanding on clinical positioning of casdatifan/where HIF2a inhibitors fits in the current treatment landscape- intended line of therapy (earlier v later), monotherapy v. combination considerations, sequencing strategies
- Biomarker expansion: additional circulating biomarkers (e.g., PTHrP) and validation of current findings in larger datasets would strengthen the translational impact. Are robust correlatives incorporated into the larger, ongoing studies? Particularly, authors mentioned higher expression of the Hallmark oxidative phosphorylation signature is associated with disease progression. In discussion section, consider expanding on this/potential mechanisms of resistance/other biomarkers of resistance that can be considered in future trials
- The limited sample size restricts precision of efficacy estimate - please note that ongoing studies, including PEAK1, will be

important for validation
- Follow up for durability endpoints (PFS and DoR) remains immature, longer follow up will help better define durability of benefit in responding populations - can the authors add whether further follow up is ongoing?

Minor comment:
- Would add more detail in the manuscript title to differentiate this from future pubs of casdatifan in RCC - suggest "Clinical and biomarker findings from a phase 2 trial of casdatifan in several cohorts of clear cell renal cell carcinoma" or something like this

Referee #5

(Remarks to the Author)
I co-reviewed this manuscript with one of the reviewers who provided the listed reports.

Version 1:

Reviewer comments:

Referee #1

(Remarks to the Author)
The authors have satisfactorily addressed my queries.

Referee #2

(Remarks to the Author)
The authors have addressed all the points clearly and thoroughly, and the changes have improved the paper.
In my opinion, the manuscript is now suitable for publication in its current form.

Referee #3

(Remarks to the Author)
I co-reviewed this manuscript with one of the reviewers who provided the listed reports.

Referee #4

(Remarks to the Author)
The authors have addressed all reviewer comments in a satisfactory manner.

Referee #5

(Remarks to the Author)
I co-reviewed this manuscript with one of the reviewers who provided the listed reports.

Open Access This Peer Review File is licensed under a Creative Commons Attribution 4.0 International License, which permits use, sharing, adaptation, distribution and reproduction in any medium or format, as long as you give appropriate credit to the original author(s) and the source, provide a link to the Creative Commons license, and indicate if changes were made.
In cases where reviewers are anonymous, credit should be given to 'Anonymous Referee' and the source.
The images or other third party material in this Peer Review File are included in the article's Creative Commons license, unless indicated otherwise in a credit line to the material. If material is not included in the article's Creative Commons license and your intended use is not permitted by statutory regulation or exceeds the permitted use, you will need to obtain permission directly from the copyright holder.
To view a copy of this license, visit https://creativecommons.org/licenses/by/4.0/

Referee #1 (Remarks to the Author): Choueiri et al provide data for casdatifan, reporting (to this reviewer's knowledge) the highest RR seen to date with a HIF2 inhibitor and also the first putative biomarker (HIF2 RNA) of response. This is a highly significant paper to the field. The following comments are offered for the authors consideration.

> **Author response:** We thank the reviewer for the positive feedback and the opportunity to strengthen our manuscript.

Major Comments:

1. In Table 1, would offer more substantial breakdown of prior systemic therapy in the dose expansion phase.

> **Author response:** We have updated Table 1 to include the proportion of patients who received 1, 2, 3, or >3 prior systemic anticancer therapies (across all treatment settings).

Minor Comments:

1. The response rate observed in this study is significantly higher than that observed with belzutifan (35%; versus a ~20% response rate with belzutifan). Can the authors speculate as to why the response rate seen is significantly higher, given that both function via allosteric binding? There is brief comment around PK parameters but more rationale could be provided.

> **Author response:** We hypothesize that casdatifan may be associated with a higher objective response rate (ORR) than belzutifan due to differences in both pharmacokinetic and pharmacodynamic properties. Unlike belzutifan, which demonstrates saturable absorption and limited exposure escalation at higher doses, casdatifan exhibits dose-proportional pharmacokinetics. This may translate into more complete HIF-2α inhibition, as suggested by the greater suppression of circulating erythropoietin levels observed with casdatifan (>80%) compared with belzutifan (~65%)[1-3]. Collectively, these findings raise the possibility that casdatifan achieves deeper and more sustained target inhibition, which may underlie the observed clinical activity and higher ORR. We have revised the Introduction section to make this point clearer:

Introduction section, page 5, paragraph 3: Casdatifan is a potent, ~~orally bioavailable,~~ selective, orally bioavailable HIF-2α inhibitor that was designed to enhance tissue penetration, reflected in its improved ~~has a potentially improved~~ pharmacodynamic profile relative to other members of the therapeutic class. ~~In preclinical studies, casdatifan inhibited transcriptional activity of HIF-2α and reduced tumor burden in ccRCC xenograft models.~~ In the phase 1 ARC-14 study, casdatifan exhibited manageable toxicity and dose-proportional and time-invariant pharmacokinetics across doses of 3–100 mg in healthy participants. Importantly, ARC-14 findings indicate that higher doses of casdatifan lead to deeper suppression of serum erythropoietin (sEPO), a well-established pharmacodynamic biomarker of HIF-2α inhibition used to assess on-target activity. Pharmacokinetic and pharmacodynamic modeling predicted that casdatifan at a dose of 20 mg once daily (QD) would produce more than 70% reduction in erythropoietin (EPO) levels from baseline, which is higher than the effect observed with the approved dose of belzutifan (120 mg QD). Notably, casdatifan doses above 20 mg QD showed mean maximal reductions in EPO levels of up to 85% in ARC-14. ~~Based on these data, we hypothesize that deeper inhibition of HIF-2α with higher casdatifan doses may translate into improved clinical activity in patients with refractory ccRCC.~~ In summary, casdatifan achieves stronger and more consistent HIF-2α inhibition in tumors.

2. In Figure 2D, can the authors provide scaling for the provided micrographs?

Author response: We believe the reviewer is referring to Figure 3d rather than Figure 2d. Figure 3d has been updated to show the length of the scale bar (50 μm) for the micrographs.

3. In Figure 2F, can the authors provide rationale for the 25% cutoff used for HIF-2a staining? Was this derived from recursive partitioning?

Author response: The 25% cutoff was determined from a natural breakpoint observed as the local minimum of a bimodal distribution in the HIF-2α staining (see **Fig. A** below) and not from recursive partitioning. This approach allowed us to distinguish between two distinct subpopulations.

Fig. A: Histogram of HIF-2α IHC tumor percent positive values. The solid vertical line represents 25% tumor positivity (cutoff used for the study), whereas the dotted vertical line represents the median.

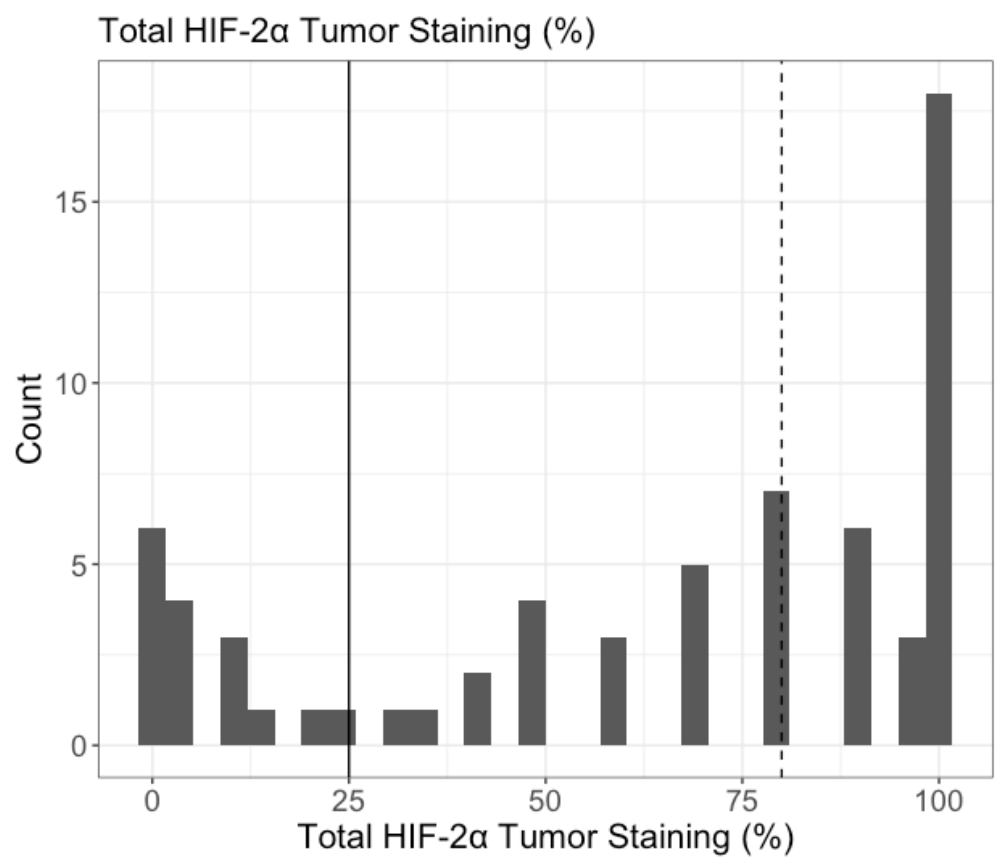

Not only was this cutoff based on staining distribution, but it was also associated with differences in expression of relevant biological gene sets (Courtney HIF-2α, Hallmark EMT and Hallmark OxPhos) that we observed in the RNA-seq data, as shown in **Fig. 4, panel b** (included below for reference). This cutoff also identified patients with longer PFS (manuscript **Fig. 2, panel f**, shown below for reference).

Manuscript Fig. 2, panels b and f.

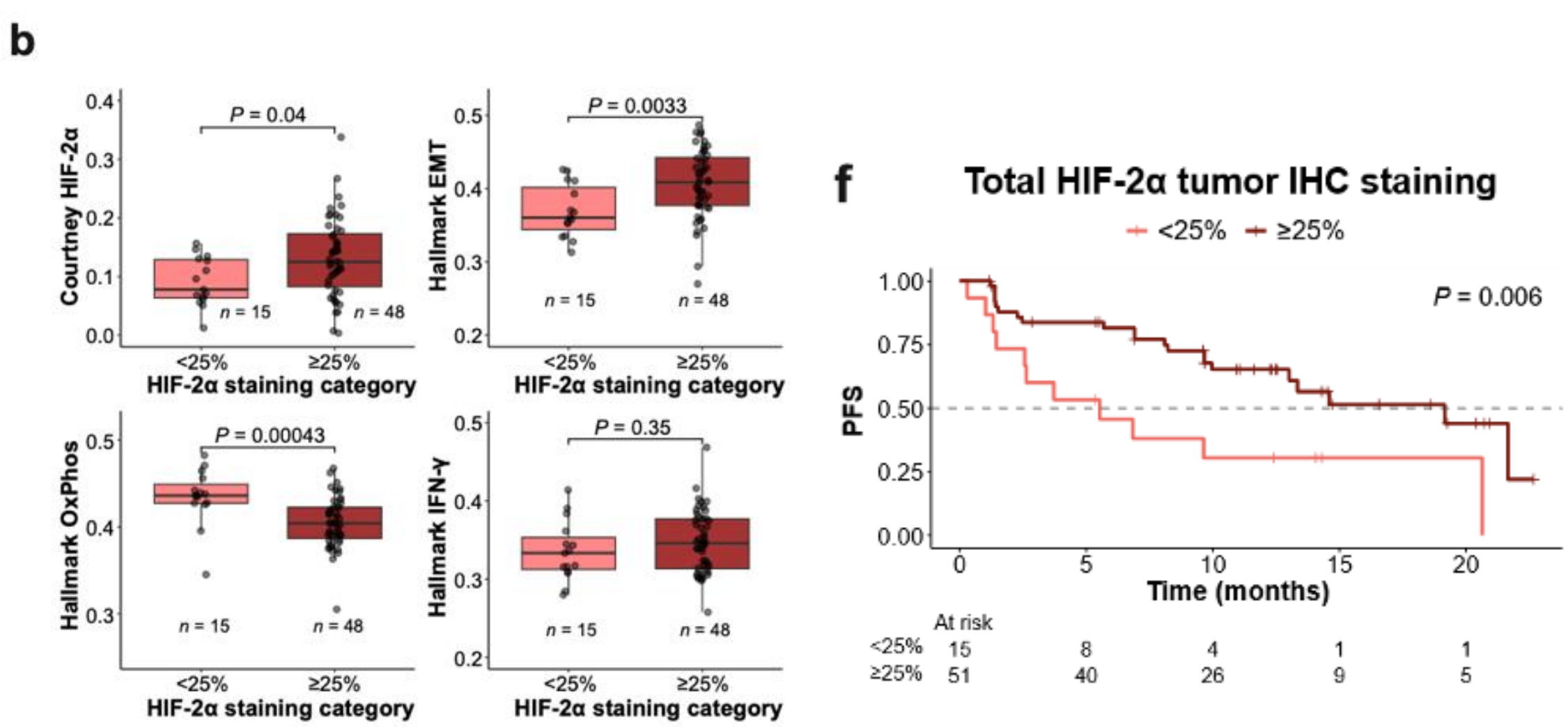

The manuscript currently provides a rationale for the selected cutoff in the Results section (page 12, paragraph 4): “Of patients with both HIF-2α IHC and EPO mRNA ISH available, we observed that those with ≥25% tumor HIF-2α IHC staining (cutoff selected based on a natural breakpoint distinguishing low vs high staining) had significantly higher levels of tEPO mRNA in tumor cells.”

4. In Figure 4C, it is unclear what the Forrest plots are articulating in terms of relative benefit. Would consider removing.

Author response: We thank the reviewer for their important observation on figure clarity. We have proposed changes to the figure and added Kaplan-Meier curves to better illustrate our results (see revisions to Figure 4c and legend, below). Briefly, this figure shows that patients with a high HIF-2α signature perform poorly with the single agent anti-VEGF sunitinib and with the anti-VEGF–containing regimens of bevacizumab plus atezolizumab and axitinib plus avelumab in the first-line ccRCC setting. Conversely, patients with high HIF-2α signatures derive PFS benefit from the single agent HIF-2α inhibitor casdatifan, as shown in the new figure below (**Extended Data Figure 5**; study design figure has been moved to the supplement to make space). In contrast, other signatures, such as OxPhos and IFN-γ signaling, do not show this pattern. Taken together, this supports the hypothesis that HIF-2α inhibition by casdatifan may address a negatively prognostic biology under current standard-of-care treatment modalities in first-line ccRCC (VEGF inhibition and anti–PD-[L]1), representing an unmet medical need that may be addressed by unique combination treatments containing casdatifan.

[Revised legend] Fig. 4. HIF-2α–dependent ccRCC~~s~~ <u>tumors</u> underperform on current therapies, supporting exploration of casdatifan-based combination strategies. a, NES of gene sets significantly enriched in patients with rapid ~~progressive disease~~ <u>PD</u> (positive scores) versus patients with disease control (negative scores). **b**, Selected gene set ssGSEA scores in patients with ≥25% HIF-2α IHC staining versus patients with <25% IHC staining. *P* value by Wilcoxon ranked-sum test is shown; **c**, Forest plot showing<u> HRs and 95% CIs o</u>f univariable Cox proportional hazard model comparing PFS of patients with high vs low ssGSEA scores (by median) of select gene <u>sets across clinical trials of different treatments: ARC-20 (casdatifan), IMmotion151 (sunitinib [*n* = 416] and atezolizumab plus bevacizumab [*n* = 407] arms) and JAVELIN</u>

Renal 101 (sunitinib [*n* = 372] and avelumab plus axitinib [*n* = 354] arms). Blue indicates that a higher signature score is associated with lower risk of progression (longer PFS). Red indicates that a higher signature score is associated with higher risk of progression (shorter PFS). 1L, first line; atezo, atezolizumab; avelu, avelumab; axi, axitinib; bev, bevacizumab; ccRCC, clear cell renal cell carcinoma; GSEA, gene set enrichment analysis; HIF, hypoxia-inducible factor; HR, hazard ratio; IFN, interferon; IHC, immunohistochemistry; IO, immunotherapy; NES, normalized enrichment score; ns, not significant; OxPhos, oxidative phosphorylation; PD, progressive disease; PFS, progression-free survival; ssGSEA, single-sample GSEA; VEGF, vascular endothelial growth factor.

[Revised] Fig. 4, panel c

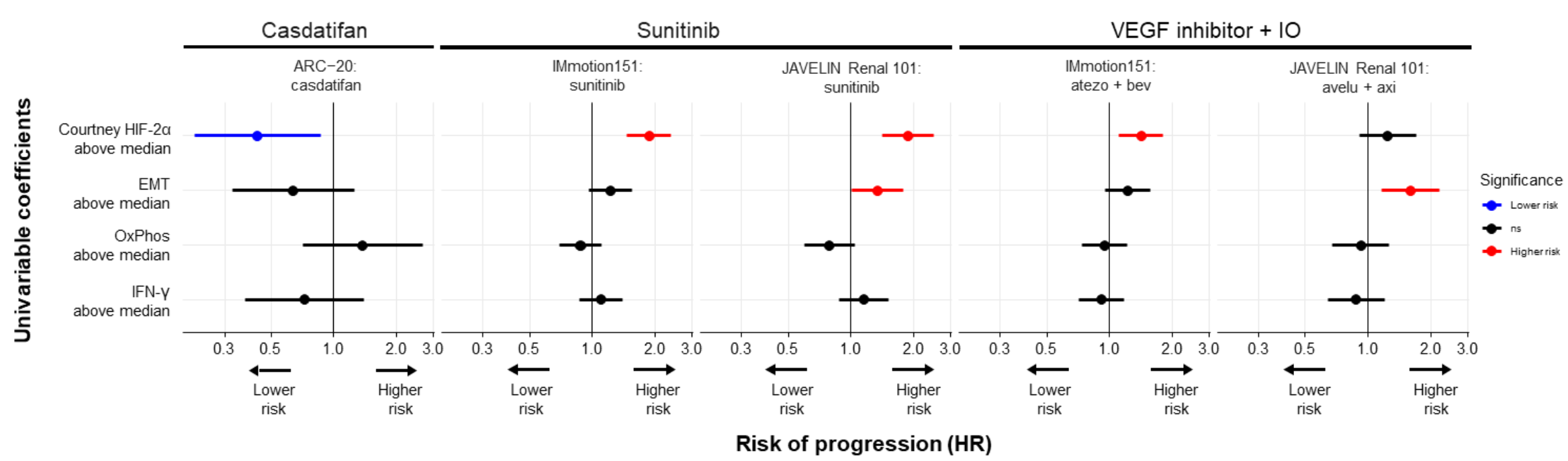

[New] Extended Data Fig. 5 | Kaplan-Meier PFS probability analysis by Courtney HIF-2α signature expression in ARC-20 and IM151. *P* values were calculated using log-rank test. atezo, atezolizumab; bev, bevacizumab; HIF, hypoxia-inducible factor; IM151, IMmotion151; PFS, progression-free survival; sig, signature.

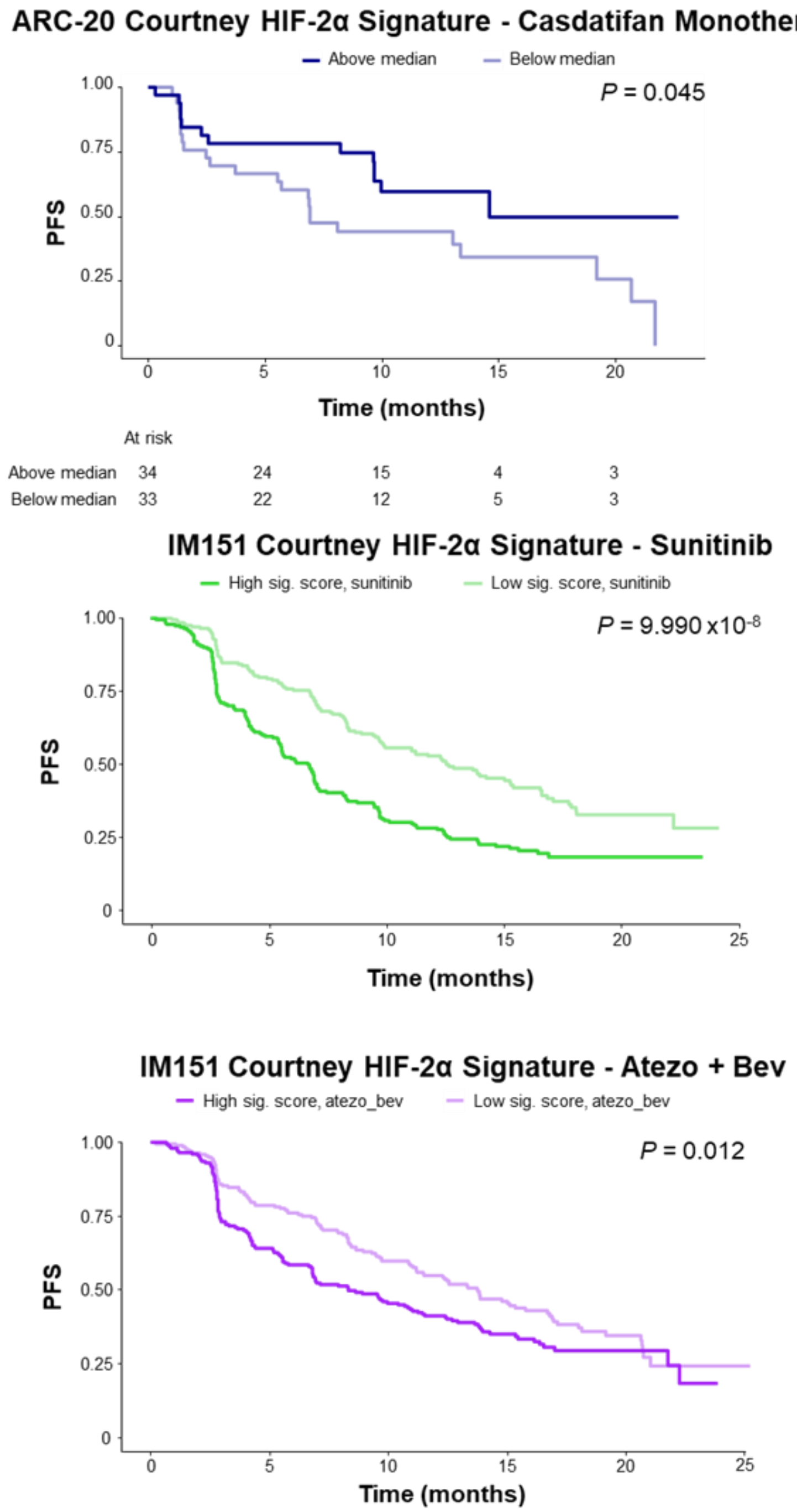

Referee #2 (Remarks to the Author): This is a well written, clinically significant publication exploring the safety, activity and pharmacodynamic biomarkers of the dose escalation and dose expansion cohorts of the ARC-20 phase 1 study of casdatifan. The authors do a great job summarizing the patient population, safety results and efficacy results of both cohorts. The inclusion of which cases of anemia led to blood transfusions and the use of erythropoietin-stimulating products and how many cases of hypoxia led to the need of oxygen supplementation as well as the time to recovery for hypoxia is especially commendable. There is also a good amount of thought to the pharmacokinetics of casdatifan and the authors explore the relationship between casdatifan dose and response, showing that there is no relationship between pk and resistance to casdatifan. The hypothesis of the study hinges on the ability of casdatifan to achieve a superior shutdown of HIF2A to belzutifan. To prove this point the authors focus in one key PD biomarker, sEPO, that it is reported to be inhibited only by 70% by belzutifan due to saturable absorption. Reduction of sEPO in this cohort is >80% for all tested doses and there was a higher likelihood of response in patients that achieve more profound reductions in sEPO. The authors show how patients with high baseline levels of tumor EPO expression had higher EPO reduction as the consequence of casdatifan and that EPO was being produced in the cancer cells and not in stromal cells, according to what is expected if driven by HIF2A overexpression. Then they showed correlation between EPO and HIF staining. Our conclusion is that this is a really strong paper, and we want to recommend mostly minor changes/aclarations

> **Author response:** Thank you for the feedback and the opportunity to revise our paper. We provide responses to your comments below.

Major comments:

1. Reduction of sEPO in different doses of casdatifan is not reported. How does the RP3D compare with other doses in terms of sEPO reduction. Is there a dose-relationship?

> **Author response:** As mentioned above, in the ARC-14 study, pharmacokinetic and pharmacodynamic modeling predicted that casdatifan at a dose of 20 mg QD would produce more than a 70% reduction in EPO levels from baseline, which is higher than the effect observed with the approved dose of belzutifan (120 mg QD)[2]. In the ARC-20 study, best median sEPO reduction was more than 80% across casdatifan doses (50 mg BID, 50 mg QD, 100 mg QD, 150 mg QD and 200 mg QD) in the biomarker-evaluable

monotherapy population, which includes the RP3D of 100 mg QD (see figure below, which has been added to the manuscript as **Extended Data Fig. 4**).

Extended Data Fig. 4 | Best sEPO reduction across all doses of casdatifan monotherapy in the ARC-20 ccRCC biomarker-evaluable population. BID, twice daily; ccRCC, clear cell renal cell carcinoma; QD, once daily; sEPO, serum erythropoietin.

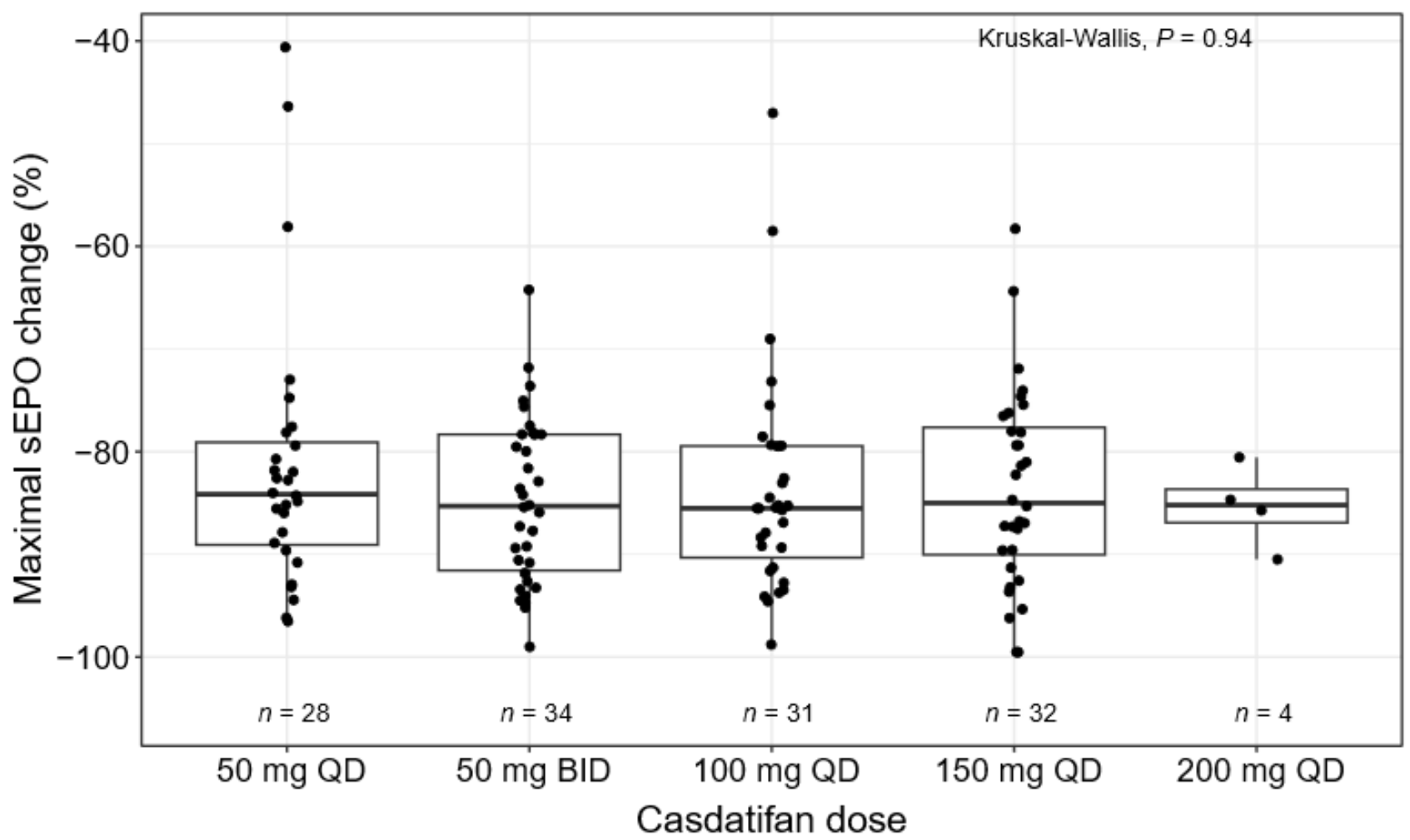

We have revised the manuscript text as follows:

> **Results section, page 11, paragraph 1:** Notably, maximal sEPO reduction was comparable across casdatifan daily doses ranging from 50 mg to 200 mg (**Extended Data Fig. 4**).

2. The authors analyze the correlation between *VHL* mutation and HIF2/EPO, and find no correlation but *VHL* function can be lost due to biallelic loss without any mutation being present. A further analysis testing the correlation of biallelic *VHL* loss + inactivating mutations with HIF2/EPO is recommended.

Author response: We thank the reviewers for highlighting the importance of assessing functional *VHL* loss. Using whole-exome sequencing data, we evaluated biallelic *VHL* inactivation through an integrated analysis of single-nucleotide variants and copy-number alterations. We did not observe a significant association between detectable biallelic *VHL* loss and HIF-2α staining, sEPO levels, or clinical outcomes (see **Fig. B**

below). We acknowledge that whole-exome sequencing may not fully capture all mechanisms of *VHL* inactivation, such as epigenetic silencing, which may contribute to the lack of observed association.

Genomic *VHL* status alone may not fully reflect downstream HIF-2α pathway activity in ccRCC. We also conducted experiments to assess potential connections between detectable biallelic *VHL* loss and HIF-2α staining, sEPO changes, or clinical outcomes; no associations were observed (**Fig. B**). Notably, *VHL* mutations were similarly not associated with clinical outcome measures in LITESPARK-013 with belzutifan[4].

Fig. B: *VHL* biallelic genetic loss is not associated with clinical outcomes, total HIF-2α tumor IHC staining, or best percent EPO change.

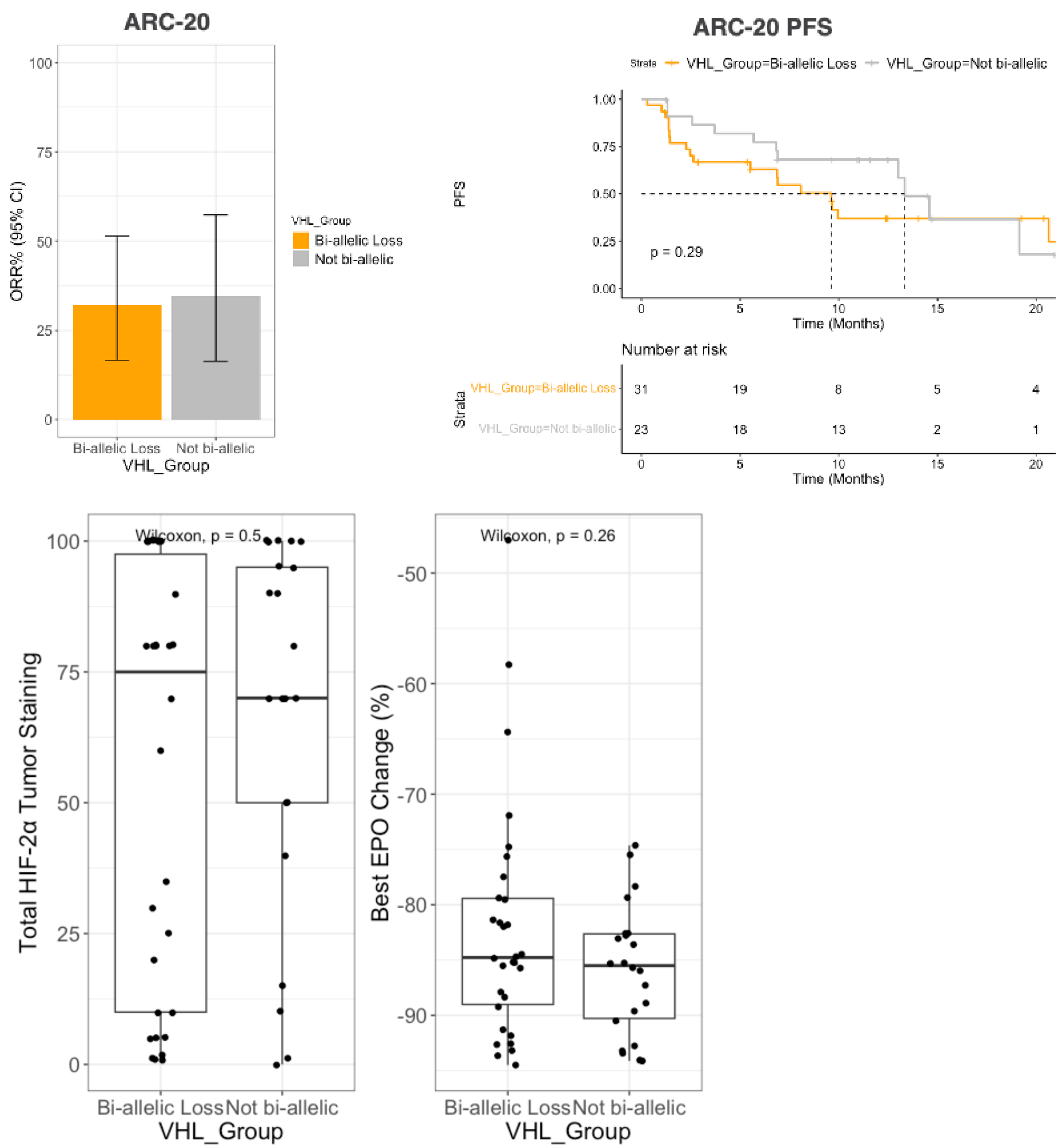

As mentioned, *VHL* may also be silenced epigenetically, but epigenetic data are not available for these samples. To further investigate other potential mechanisms of *VHL* silencing, we hypothesized that both genetic and/or epigenetic loss of *VHL* could be captured at the transcriptional level using transcriptomics data. We derived a *VHL* mutation signature score using data from the IMmotion151 clinical trial dataset. Briefly,

we performed differential gene expression analysis in ccRCC from the IMmotion151 dataset (sarcomatoid tumors were excluded) and identified 184 genes with higher expression in patients with *VHL* mutations. This set of genes was validated in the IMmotion150 dataset by calculating an ssGSEA score for each patient and comparing patients with *VHL* mutations vs patients with wild-type (WT) *VHL*. We then applied the *VHL* mutation signature score to data from patients with ccRCC in The Cancer Genome Atlas-Kidney Renal Clear Cell Carcinoma (TCGA-KIRC) dataset and found that *VHL* mutation signature scores were significantly higher (P = 3.1 x 10^{-14}) in tumors with *VHL* mutation and copy number loss compared with tumors with WT *VHL* or reported *VHL* amplifications (**Fig. C**).

Fig. C: Higher *VHL* mutation signature scores in tumors with altered *VHL* compared with tumors with WT *VHL* with neutral copy number change.

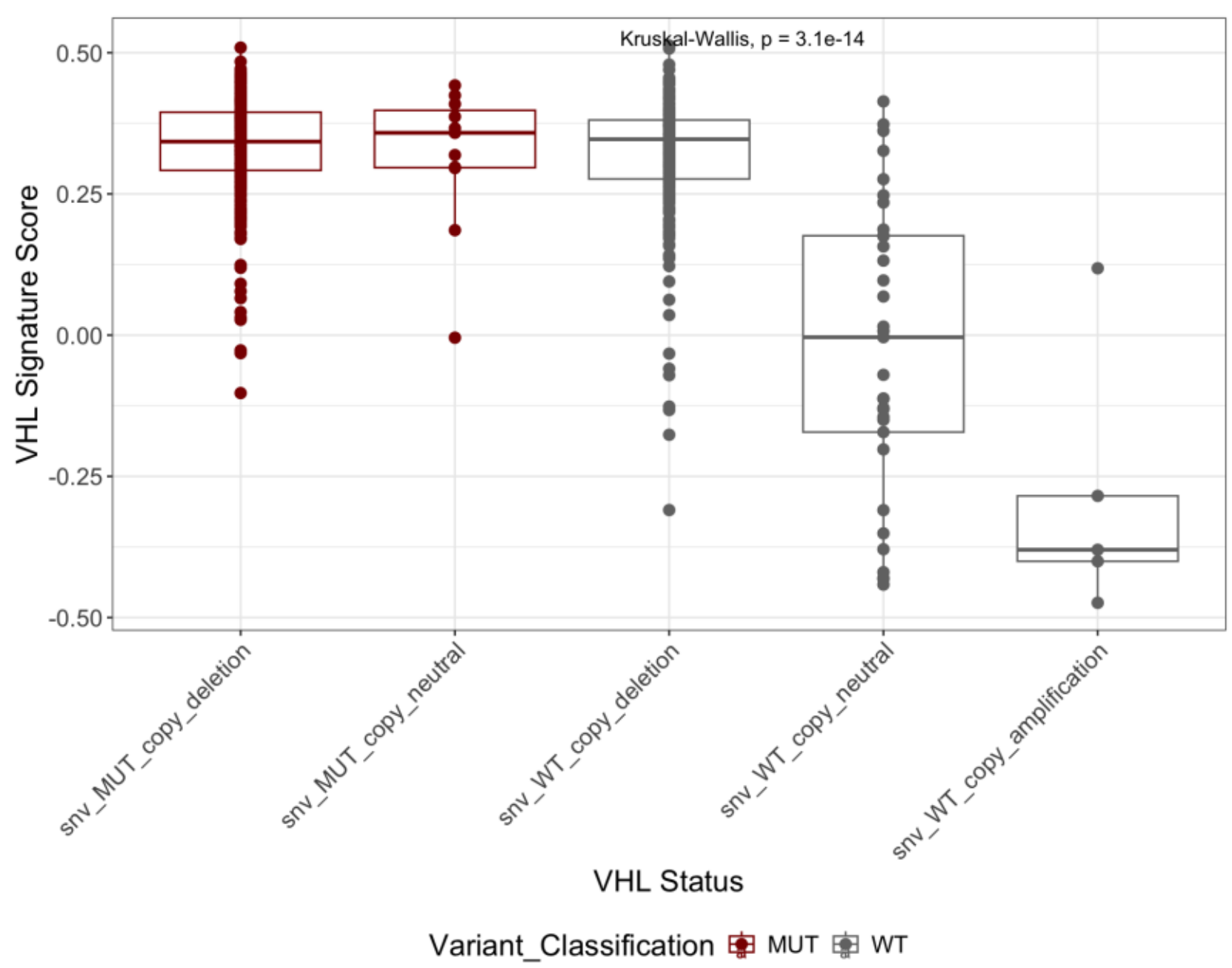

We further analyzed data from patients with paired methylation measurements in the TCGA-KIRC dataset. Patients with WT *VHL* classification, who frequently presented higher levels of methylation at most promoter-associated probes, also tended to express higher levels of the *VHL* mutation signature score despite no apparent mutation to the *VHL* gene. This suggests that patients with apparent WT *VHL* can undergo *VHL* disruption through alternative mechanisms, captured through derived *VHL* mutational signature levels (**Fig. D**).

Fig. D: *VHL* mutation signature score by *VHL* promoter methylation and mutation status in patients with ccRCC from the TCGA-KIRC dataset. Patients with WT *VHL* classification typically demonstrated higher levels of methylation at probes within the *VHL* promoter region compared with patients with mutant *VHL*, accompanied by similarly high levels of *VHL* mutation signature score.

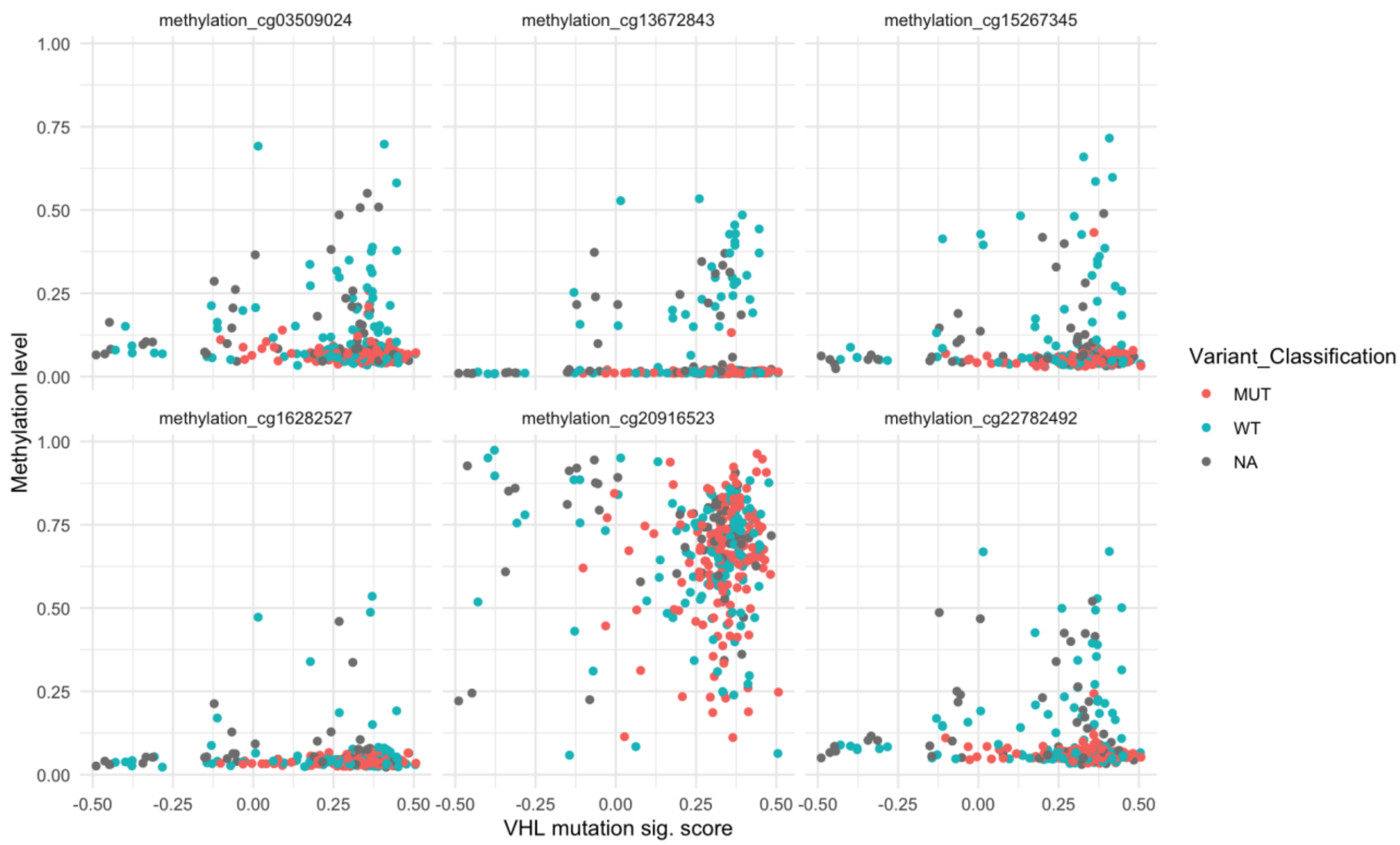

We then investigated the same question for patients in the ARC-20 monotherapy cohort who had tumors with available data. We found that *VHL* mutation signature scores were comparable between tumors with apparent WT *VHL* and tumors with *VHL* mutation (**Fig. E**), which suggests that other forms of *VHL* silencing may be occurring in a substantial subset of the apparent WT *VHL* tumors.

Fig. E: Distribution of *VHL* mutation signature scores in tumors with mutant or apparently WT *VHL*. Vertical ticks represent samples.

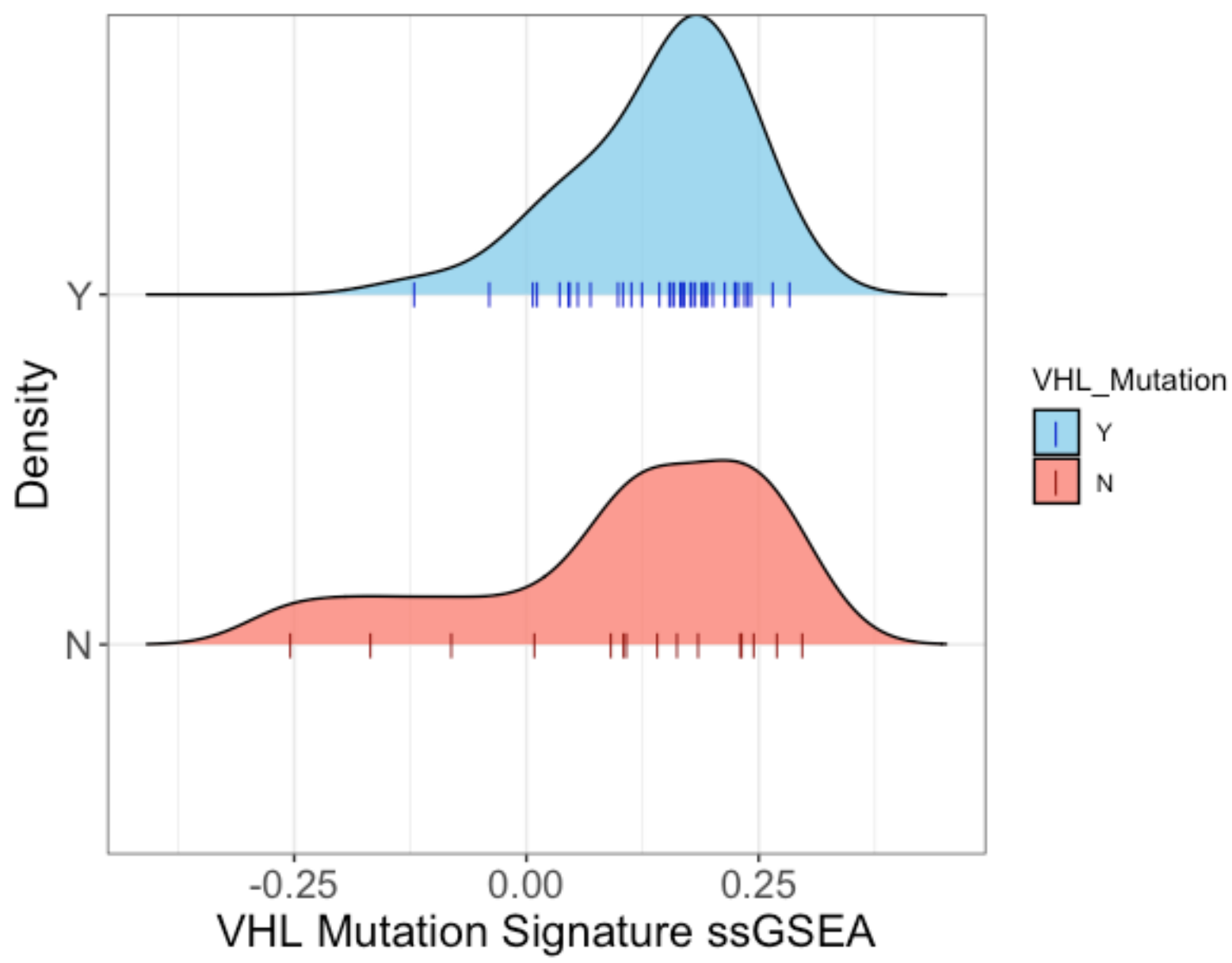

3. Presence of a (OxPhos) signature is correlated with higher resistance to casdatifan, while this might represent patients in which HIF2 is not overexpressed, there is evidence that the lack of *VHL* can result in abnormal expression of HF1A in patients with ccRCC and this can mediate resistance to immunotherapy. We suggest an analysis of the potential role of HIF1A as resistance mechanism to casdatifan. https://jitc.bmj.com/content/12/2/e008226

Author response: In the ARC-20 study, we evaluated HIF-1α protein levels using a proprietary HIF-1α IHC assay in the subset of patients treated with casdatifan who had available tumor IHC data. We observed no association between HIF-1α staining and primary progressive disease rate (**Fig. F, left panel**). We also explored the association between total HIF-1α tumor staining and PFS using two different cutoffs: ≥25% (similar to the HIF-2α analysis) and ≥ median (≥60%), which was also the natural breakpoint of a

bimodal distribution observed for HIF-1α staining. No association was observed between HIF-1α staining and PFS at either cutoff (**Fig. F, right panel**).

Fig. F: No association between HIF-1α IHC staining and progressive disease rate or PFS (≥ median cutoff shown; results were similar with the ≥25% cutoff).

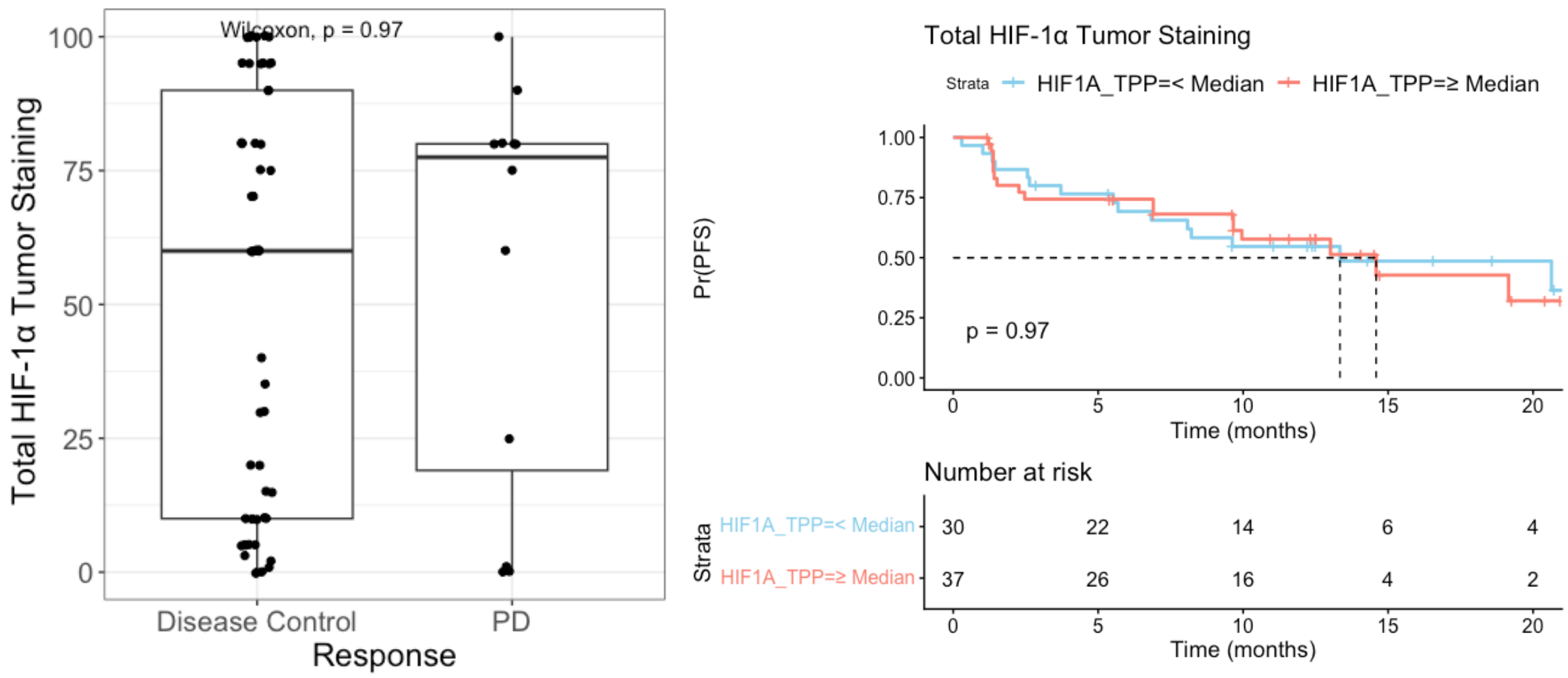

We revised the manuscript to summarize these findings.

> **Results section, page 13, paragraph 1:** Notably, patients with ≥25% tumor HIF-2α IHC staining had significantly longer PFS compared with those with <25% tumor HIF-2α IHC staining (*P* = 0.006; **Fig. 3f**). In addition, we investigated the relationship between HIF-1α IHC staining in a similar manner and observed no association between HIF-1α expression and primary PD rate or PFS (data not shown).

Minor comments:

1. Treatment with belzutifan was an exclusion criteria in the expansion cohorts, but authors should clarify if any patient in the escalation phase received previous HIF2 inhibition.

Author response: We revised the Results section to clarify that patients in the dose-escalation stage did not previously receive treatment with a HIF-2α inhibitor, except for one patient who received prior belzutifan treatment.

Results section, page 7, paragraph 1: None of the patients had previously received treatment with a HIF-2α inhibitor, except for one patient in the 50 mg twice daily (BID) cohort who had received prior belzutifan treatment for 6 months as a fifth-line treatment (discontinued because of progressive disease [PD]).

2. The inclusion of a CONSORT-DEFINE checklist would have helped review the completion of all the recommended elements.

Author response: We have reviewed the CONSORT-DEFINE checklist to ensure that all applicable items are addressed in the manuscript. The completed checklist has been included with the submission files. Corresponding manuscript revisions made to align with the checklist are outlined below. These revisions address checklist items related to study rationale, additional methodological details and reporting of previous presentations.

Methods, pages 31–35: ARC-20 was designed to establish the safety and biologically active dose of casdatifan and to directly link the depth of HIF-2α inhibition to pharmacodynamic effects, tumor biology and clinical benefit in refractory metastatic ccRCC.

Safety decisions were guided by a dose-escalation committee (DEC) and a safety review committee (SRC). The DEC reviewed cohort-level safety and DLT data to guide dose escalation and dose clearance for expansion, while the SRC evaluated safety during the dose-expansion stage. The DEC and SRC were not independent of the study sponsor. Final decisions regarding continuation or termination of the trial or its components were made under sponsor governance and were informed by committee recommendations. Access to interim information was restricted to authorized study personnel and committee members and was kept confidential. Further details, including composition of the DEC and SRC, are provided in the study protocol (available as supplementary material).

Preliminary findings from ARC-20 were previously presented at the EORTC-NCI-AACR Symposium, October 23–25, 2024, Barcelona, Spain; American Society of Clinical Oncology Genitourinary Cancers Symposium, May 30–June 3, 2025, Chicago, Illinois; Kidney Cancer Research Summit, July 17–18, 2025, Boston, Massachusetts; and American Society of Clinical Oncology Genitourinary Cancers Symposium, February 26–28, 2026, San Francisco, California.

The starting dose of 20 mg QD was selected based on the totality of nonclinical data and findings from the first-in-human study of casdatifan in healthy participants (ARC-14), which suggested manageable toxicity at this dose. The dose range of 20–200 mg QD was selected to enable evaluation of a broad range of exposures and to inform clinical development. Patients were enrolled on a rolling basis in sets of three. After three patients in a set were dosed, enrollment was paused until the 3-week treatment cycle was completed. Dosing then resumed by adding another set of three to the same dose level or by escalating to the next dose level according to the 3 + 3 design. The maximum tolerated dose corresponded to the highest dose at which the DLT incidence was below 33%. Further details on methodology for the dose-escalation stage are reported in Supplementary Table 2. Specific criteria for dose discontinuation, dose modification and dosing delays are provided in the study protocol (available as supplementary material).

Dose-expansion cohorts were permitted to initiate before completion of dose escalation, provided the dose had cleared DLT evaluation.

Specific criteria for dose discontinuation, dose modification and dosing delays were prespecified in the study protocol (available as supplementary material).

If any individual expansion dose demonstrated preliminary signs of efficacy and an acceptable safety profile as determined by the SRC, an additional 30 patients could enroll into the cohort.

Methods, pages 39: The biomarker-evaluable population included patients with ccRCC in the dose-escalation and dose-expansion monotherapy cohorts who received daily doses of casdatifan from 50 mg to 200 mg and had available, analyzable serum or tissue biomarker data. The biomarker-evaluable population also included two patients who experienced non-evaluable confirmed best overall response due to PD before their first on-treatment scan. These two patients were classified as experiencing PD for biomarker analyses.

Supplementary Table 2: Evaluation of additional dose levels was allowed based on emerging data on safety and/or pharmacokinetics but could not exceed 200 mg QD or a 100% dose increase from the previous cohort

For patients who may derive clinical benefit at a dose level lower than the highest dose that is deemed safe and tolerable, intra-patient dose escalation was allowed after discussion with the sponsor's medical monitor if:

- Escalation to the next dose level that has been deemed safe was at least 1 dose level below the actively enrolling dose
- Patients had signs of clinical benefit (eg, stable disease with some tumor shrinkage, improvement of symptoms, an objective response)
- Patients tolerated their current dose level (eg, did not experience a DLT) and had only toxicities considered grade ≤2 during the prior treatment cycle

3. The authors could have discussed if a dose-escalation strategy focused on optimizing the reduction of sEPO could be a beneficial strategy to optimize tumor responses.

Author response: We appreciate this comment from the reviewer; however, we note that the casdatifan 100 mg daily dose has been chosen for further investigation in the ongoing phase 3 PEAK-1 trial based on a totality of evidence, including safety and efficacy. Although pharmacokinetics are an important component of understanding

casdatifan's clinical benefits, sEPO reduction was comparable across daily casdatifan doses ranging from 50 mg to 200 mg (data now shown in **Extended Data Fig. 4**), as mentioned above in our response to **Referee #2, query 1**. In addition, the safety profile of casdatifan is generally consistent across daily doses of 50 mg to 100 mg[5]. Based on the totality of pharmacokinetic, pharmacodynamic, safety and efficacy data, we believe that casdatifan at a dose of 100 mg QD is the most appropriate initiation dose for patients to achieve optimal clinical outcomes.

4. Presence of a (OxPhos) signature is correlated with higher resistance to casdatifan, while this might represent patients in which HIF2 is not overexpressed, there is evidence that the lack of *VHL* can result in abnormal expression of HIF1A in patients with ccRCC and this can mediate resistance to immunotherapy. We suggest a analysis of the potential role of HIF1A as resistance mechanism to casdatifan. https://jitc.bmj.com/content/12/2/e008226

Author response: As indicated above in our response to **Referee #2, query 3**, we evaluated HIF-1α protein levels using a proprietary HIF-1α IHC assay in the subset of patients in ARC-20 treated with casdatifan who had available tumor IHC data. In patients with available HIF-1α IHC data, we observed no association between HIF-1α staining and primary progressive disease rate or PFS in patients treated with casdatifan.

5. There is a subset of patients with ccRCC that develop secondary inactivating mutations in HIF1A, while this results in a worse prognosis, they would also be the ones in which the HIF2A signature would be more clear and could potentially explain the trend towards a more profound benefit of casdatifan in patients with worse IMDC score.
https://www.nature.com/articles/s41467-020-17873-3

Author response: We appreciate the reviewer's comment and acknowledge the hypothesis that secondary inactivating mutations in HIF-1α could potentially contribute to heterogeneity in clinical benefit observed with HIF-2α inhibition. In the ARC-20 study, the number of paired pre- and post-treatment tumor biopsies was limited (n = 2), precluding effective evaluation of the potential association of *HIF1A* mutations with the clinical benefit of casdatifan. Accordingly, this hypothesis could not be directly tested in ARC-20; however, this is an interesting research question that warrants investigation in future studies incorporating longitudinal tumor sampling and integrated molecular profiling.

Referee #3 (Remarks to the Author): I co-reviewed this manuscript with one of the reviewers who provided the listed reports.

> **Author response:** Thank you for reviewing this paper. We appreciate the opportunity to revise and resubmit our manuscript.

Referee #4 (Remarks to the Author): Choueiri et al have compiled the results of the ARC-20 phase 2 trial of casdatifan in refractory clear cell RCC. This is an impactful study demonstrating that casdatifan produces a deep on-target effect with encouraging efficacy in a heavily pretreated population enriched for intermediate- and poor-risk RCC. Antitumor activity (ORR 31–35%, DCR 81–84%) appears clinically meaningful and responses appear durable. Toxicities were consistent with the expected class effects of HIF-2α inhibition and overall manageable, with no treatment-related deaths reported, although anemia and hypoxia remain clinically significant. There is encouraging biomarker analysis of erythropoietin reduction correlating with clinical benefit, with higher baseline tumor EPO mRNA expression associated with response. Transcriptomic GSEA further demonstrates that a HIF-2α gene signature is associated with longer PFS with casdatifan but shorter PFS with non–HIF-2α regimens, supporting HIF-2α inhibition as a rational therapeutic strategy in biomarker-selected patients. Overall the analysis was comprehensive and manuscript is well written, the figures are clear, and the data is well presented.

> **Author response:** We appreciate the reviewer's feedback and the opportunity to strengthen our paper.

Several considerations to strengthen the manuscript:

1. In discussion section, consider expanding on clinical positioning of casdatifan/where HIF2a inhibitors fits in the current treatment landscape- intended line of therapy (earlier v later), monotherapy v. combination considerations, sequencing strategies

> **Author response:** We agree that the Discussion section should better contextualize casdatifan within the evolving ccRCC treatment landscape. We have revised the manuscript to note that, based on the FDA approval of belzutifan and clinical practice guidelines, HIF-2α inhibition is a therapeutic option for advanced ccRCC after treatment with a PD-(L)1 inhibitor and a VEGFR-TKI[3,6]. We have added a recent biomarker analysis of belzutifan[7] to provide additional context for interpreting these data. We also clarify that, although the present monotherapy data for casdatifan are encouraging, additional studies are needed to validate findings and inform its place in therapy. Overall, based on the ongoing clinical development program, the most likely near-term clinical positioning of casdatifan appears to be in combination-based strategies rather than as stand-alone therapy. These revisions are shown below:

Discussion section, page 17, paragraph 3: To our knowledge, this is one of the first systematic and comprehensive ~~study~~ studies linking patient-level tumor biology and clinical activity for a HIF-2α inhibitor... Consistent with observations in the periphery, baseline tEPO mRNA expression was higher in patients who experienced better outcomes from casdatifan ~~disease control, and higher tEPO expression was associated with longer PFS~~. Patients with higher levels of tumor HIF-2α protein expressed higher levels of tEPO as measured by ISH and experienced longer PFS, linking sEPO, tEPO and HIF-2α biology to clinical benefit with casdatifan. This is consistent with results from a biomarker analysis of LITESPARK-013, in which a high HIF-2α tumor positivity score was associated with improved PFS with belzutifan treatment.

Discussion section, page 18, paragraph 3: Currently, HIF-2α inhibition is a therapeutic option for patients with advanced ccRCC after treatment with a PD-(L)1 inhibitor and a VEGFR-TKI. The antitumor activity of casdatifan in this setting appears to be improved compared with prior HIF-2α inhibitors, although additional studies are needed to validate findings and inform casdatifan's place in the current treatment landscape. The phase 3 randomized PEAK-1 study (NCT07011719) is evaluating casdatifan plus cabozantinib in previously treated advanced ccRCC. Based on the ongoing clinical development program, casdatifan is likely to be positioned in combination with current standard of care rather than as monotherapy.

2. Biomarker expansion: additional circulating biomarkers (e.g., PTHrP) and validation of current findings in larger datasets would strengthen the translational impact. Are robust correlatives incorporated into the larger, ongoing studies? Particularly, authors mentioned higher expression of the Hallmark oxidative phosphorylation signature is associated with disease progression. In discussion section, consider expanding on this/potential mechanisms of resistance/other biomarkers of resistance that can be considered in future trials

Author response: We appreciate this insight from the Reviewer. In ongoing clinical trials with larger study populations, we are continuing to collect and assess sEPO measurements, which will offer an opportunity to validate the findings described here. Notably, the current analysis is restricted to monotherapy patients, whereas the ongoing

studies—including the phase 3 PEAK-1 study (NCT07011719)—will evaluate combination regimens.

We revised the Discussion section to highlight areas for future research:

> **Discussion section, page 19, paragraph 2:** Future studies (eg, PEAK-1) will validate these findings in a larger study population and investigate connections between exploratory biomarkers and clinical outcomes.

3. The limited sample size restricts precision of efficacy estimate - please note that ongoing studies, including PEAK1, will be important for validation

Author response: We have noted in the Discussion section that future studies will validate our findings:

> **Discussion section, page 19, paragraph 2:** Future studies (eg, PEAK-1) will validate these findings in a larger study population...

4. Follow up for durability endpoints (PFS and DoR) remains immature, longer follow up will help better define durability of benefit in responding populations - can the authors add whether further follow up is ongoing?

Author response: Yes, the ARC-20 study is ongoing. We have clarified in the Discussion section that longer follow-up will provide additional insight into the clinical benefit of casdatifan:

> **Discussion section, page 19, paragraph 2:** The ARC-20 study is ongoing, and extended follow-up will provide additional insight into the durability of clinical benefit among responders and the activity of casdatifan in combination with other therapies.

Minor comment:

1. Would add more detail in the manuscript title to differentiate this from future pubs of casdatifan in RCC - suggest "Clinical and biomarker findings from a phase 2 trial of casdatifan in several cohorts of clear cell renal cell carcinoma" or something like this

> **Author response:** Thank you for the suggestion. We revised the title to better differentiate the manuscript from future casdatifan publications. The title now reads: Casdatifan in kidney cancer: clinical and biomarker findings from the ARC-20 study.

Referee #5 (Remarks to the Author): I co-reviewed this manuscript with one of the reviewers who provided the listed reports.

> **Author response:** Thank you for your review. We appreciate the opportunity to improve our manuscript.

References

1. Marathe, D. D. et al. Exposure-response analyses for belzutifan to inform dosing considerations and labeling. *J Clin Pharmacol* **64**, 1246–1258 (2024).

2. Ghasemi, M. et al. Pharmacokinetics, pharmacodynamics and safety of casdatifan, a novel hypoxia-inducible factor-2α inhibitor, in healthy participants. *Br J Clin Pharmacol* **91**, 3366–3377 (2025).

3. Welireg (belzutifan). Prescribing information. Merck & Co., Inc.; 2025. Accessed October 19, 2025. https://www.merck.com/product/usa/pi_circulars/w/welireg/welireg_pi.pdf.

4. Agarwal, N. et al. Biomarker analysis from the phase 2 LITESPARK-013 study of 2 different belzutifan (bel) doses in participants (pts) with advanced clear cell renal cell carcinoma (ccRCC). *J Clin Oncol* **44**, 545-545 (2026).

5. Choueiri, T. K. et al. Casdatifan (Cas) monotherapy in patients (pts) with previously treated clear cell renal cell carcinoma (ccRCC): Safety, efficacy and subgroup analysis across multiple doses from ARC-20, a phase 1 open-label study. *J Clin Oncol* **43**, 441–441 (2025).

6. Powles, T. et al. Renal cell carcinoma: ESMO Clinical Practice Guideline for diagnosis, treatment and follow-up. *Ann Oncol* **35**, 692-706 (2024).

7. Agarwal, N. et al. Randomized phase II dose comparison LITESPARK-013 study of belzutifan in patients with advanced clear cell renal cell carcinoma. *Ann Oncol* **35**, 1148–1156 (2024).
